# Synonymous variants that disrupt messenger RNA structure are significantly constrained in the human population

**DOI:** 10.1093/gigascience/giab023

**Published:** 2021-04-05

**Authors:** Jeffrey B S Gaither, Grant E Lammi, James L Li, David M Gordon, Harkness C Kuck, Benjamin J Kelly, James R Fitch, Peter White

**Affiliations:** Computational Genomics Group, The Institute for Genomic Medicine, Nationwide Children's Hospital, 575 Children's Crossroad, Columbus, OH 43215, USA; Computational Genomics Group, The Institute for Genomic Medicine, Nationwide Children's Hospital, 575 Children's Crossroad, Columbus, OH 43215, USA; Computational Genomics Group, The Institute for Genomic Medicine, Nationwide Children's Hospital, 575 Children's Crossroad, Columbus, OH 43215, USA; Computational Genomics Group, The Institute for Genomic Medicine, Nationwide Children's Hospital, 575 Children's Crossroad, Columbus, OH 43215, USA; Computational Genomics Group, The Institute for Genomic Medicine, Nationwide Children's Hospital, 575 Children's Crossroad, Columbus, OH 43215, USA; Computational Genomics Group, The Institute for Genomic Medicine, Nationwide Children's Hospital, 575 Children's Crossroad, Columbus, OH 43215, USA; Computational Genomics Group, The Institute for Genomic Medicine, Nationwide Children's Hospital, 575 Children's Crossroad, Columbus, OH 43215, USA; Computational Genomics Group, The Institute for Genomic Medicine, Nationwide Children's Hospital, 575 Children's Crossroad, Columbus, OH 43215, USA; Department of Pediatrics, College of Medicine, The Ohio State University, 370 W. 9th Avenue, Columbus, OH 43210, USA

**Keywords:** synonymous variant, RNA structure, mRNA stability, genetic disease, Apache Spark, genomics

## Abstract

**Background:**

The role of synonymous single-nucleotide variants in human health and disease is poorly understood, yet evidence suggests that this class of “silent” genetic variation plays multiple regulatory roles in both transcription and translation. One mechanism by which synonymous codons direct and modulate the translational process is through alteration of the elaborate structure formed by single-stranded mRNA molecules. While tools to computationally predict the effect of non-synonymous variants on protein structure are plentiful, analogous tools to systematically assess how synonymous variants might disrupt mRNA structure are lacking.

**Results:**

We developed novel software using a parallel processing framework for large-scale generation of secondary RNA structures and folding statistics for the transcriptome of any species. Focusing our analysis on the human transcriptome, we calculated 5 billion RNA-folding statistics for 469 million single-nucleotide variants in 45,800 transcripts. By considering the impact of all possible synonymous variants globally, we discover that synonymous variants predicted to disrupt mRNA structure have significantly lower rates of incidence in the human population.

**Conclusions:**

These findings support the hypothesis that synonymous variants may play a role in genetic disorders due to their effects on mRNA structure. To evaluate the potential pathogenic impact of synonymous variants, we provide RNA stability, edge distance, and diversity metrics for every nucleotide in the human transcriptome and introduce a “Structural Predictivity Index” (SPI) to quantify structural constraint operating on any synonymous variant. Because no single RNA-folding metric can capture the diversity of mechanisms by which a variant could alter secondary mRNA structure, we generated a SUmmarized RNA Folding (SURF) metric to provide a single measurement to predict the impact of secondary structure altering variants in human genetic studies.

## Background

Accurate molecular genetic diagnosis of a rare disease is essential for patient care [1], yet today's best molecular tests and analysis strategies leave 60–75% of patients without a diagnosis [2–6]. Current clinical practice for sequence variant interpretation focuses primarily on missense, nonsense, or canonical splice variants [7], with numerous computational methods for prediction of the impact of non-synonymous single-nucleotide variants (nsSNVs) on protein function [8]. By contrast, we have limited knowledge in regard to the role that synonymous single-nucleotide variants (sSNVs) may have in health and disease. These variants modify the codon in a transcript but leave the protein unchanged, and for years were erroneously considered to be “silent.” However, the past 2 decades have seen a growing understanding that synonymous codons serve vital regulatory functions [9–12].

**Figure 1:**
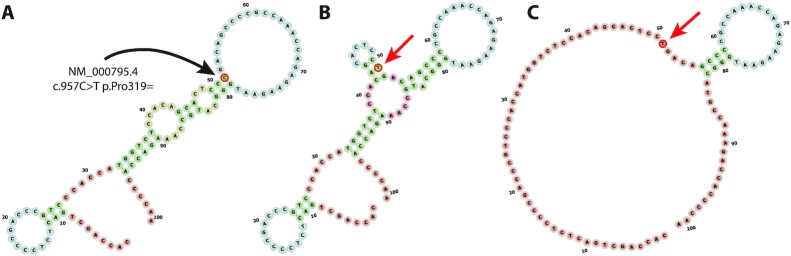
A synonymous variant introduces a marked change in local minimum free energy of the mRNA secondary structures in the *DRD2* gene. Using a known synonymous variant of pharmacogenomic significance in the dopamine receptor, DRD2 ( NM_000795.4: c.957C>T (p.Pro319 = )), this figure demonstrates how the 101-bp window used in our analysis captures the variant's impact on RNA secondary structure. Wild-type (**A**) and mutant (**B** and **C**) sequences (RefSeq transcript NM_000795.4, coding positions 907–1008) are identical except for a synonymous C→T mutation at position 51 (major “C” allele is indicated by the black arrow; minor “T” allele is indicated by the red arrow). **(A)** Wild-type optimal and centroid structures (which coincide) demonstrate a relatively stable secondary structure with a minimum free energy of −12.5 kcal/mol. In the ensemble of possible structures arising from the sSNV at position 51, there is a significant reduction in stability of the molecule in terms of both the (**B**) mutant optimal structure (−11.5 kcal/mol) and **(C)** mutant centroid structure (−5.1 kcal/mol). The synonymous variant results in a less stable mRNA molecule, which laboratory studies demonstrate reduces the half-life of the transcript, ultimately reducing protein expression of the dopamine receptor, DRD2. Nucleotides are colored according to the type of structure in which they occur: green: stems (canonical helices); red: multiloops (junctions); yellow: internal loops; blue: hairpin loops; orange: 5′ and 3′ unpaired region.

One of the principal levers by which synonymous codons direct the translational process is through messenger RNA (mRNA) structure. Unlike DNA, an mRNA molecule is single stranded and therefore capable of forming complex configurations largely by base-pairing with itself, yielding the “secondary structure," which further folds through covalent attractions to form the “tertiary structure" (Fig. 1) [13]. The secondary structure has proven to be essential for understanding the regulatory functions of RNAs, and sophisticated methods exist to predict the ensemble of possible structures that a given mRNA strand can adopt [14]. An important physical property of an RNA structure is its stability, which is defined as the extent to which an RNA molecule retains its structural integrity. RNA stability is largely a function of G+C content of the molecule in question, although most of the energy comes from the stacking-energy of the G = C pairs rather than the pairs individually [15].

Studies first published in 1999 indicated that stable mRNA secondary structures are selected for in key genomic regions across all kingdoms of life [16–19]. Stable RNA has a longer functional half-life, being more resistant to degradation or base-catalyzed hydrolysis, and stronger coding structures can endure more rounds of translation, ultimately resulting in more protein [17, 20–25]. Repeated translation destabilizes an RNA, weakening the brakes on ribosomal translational speed and producing collisions that trigger decay pathways [26–31]. There are however cases where weak structure is more desirable, most notably in the 5′ untranslated regions (UTRs) and around the start codon, to make it easier to commence translation [17, 32–37]. Diminished stability in stress response genes may also permit a more dynamic response of the cell to stress [33]. The stability of an mRNA transcript can also determine the speed of translation [16, 18, 19, 29, 38, 39] and vitally facilitate or prevent microRNAs and RNA-binding proteins from attaching to specific structural motifs [40–44]. Studies have also strongly linked mRNA structure to protein conformation and function, with synonymous codons acting as a subliminal code for the protein-folding process [12, 29, 45–50]. Given all these mechanisms, when synonymous variants are ignored, we are almost certainly missing novel plausible explanations for genetic disease.

The growing understanding of the importance of RNA structure has inspired a rich literature of *in silico* secondary structure prediction methods. One culminating study looked at predicted structures across the genomes of 17 vertebrates and found 516,000 structurally conserved elements across species, with the most conserved structures lying in coding regions [51]. An analogous work focusing on 23 drosophilids and 4 other insect species found 345,000 structurally conserved elements [52], and recently a study on the whole Tree of Life found comparable conservation [53]. As we have done in the present work, all 3 of these previous studies used the ViennaRNA package [14] (or tools built to utilize it, such as CMFinder [54] and RNAz [55]). In an alternative approach, 1 group trained a machine-learning algorithm called RNAsnap on both single and multiple-aligned sequences to predict solvent accessibility in protein-bound RNA tertiary structures [56]. Anticipating our study, the authors found decreased minor allele frequencies (MAFs) in the 1000 Genomes database [57] at structurally significant positions (Supplementary Fig. S7 shows that the pattern of constraint observed with *P*(MAF) > 0 is maintained when using the log(MAF) statistic used by Yang et al. [56]). In a study similar to ours but more limited in scope, the authors compared wild-type and mutated predicted structures to identify “RiboSNitches” or structurally disruptive SNVs in 5′ UTRs [58, 59]. However, the authors were limited by the computational cost of computing folding statistics for every SNV of interest.

Despite the widespread scientific interest in mRNA structure, its role in human health and disease remains poorly comprehended, and relatively few pathogenic synonymous variants affecting mRNA folding have been described [20, 21, 23, 25]. A structure-altering sSNV in the dopamine receptor DRD2 inhibited protein synthesis and accelerated mRNA degradation [60]. An sSNV in the *COMT* gene, implicated in cognitive impairment and pain sensitivity, was shown *in vitro* to constrain enzymatic activity and protein expression [61]. An sSNV in the *OPTC* gene of a patient with glaucoma resulted in decreased protein expression *in vivo* [62]. In patients with cystic fibrosis, an sSNV in *CFTR* was linked to decreased expression [63], and an mRNA-secondary-structure–altering silent codon change contributed to *CFTR* dysfunction by altering the dynamics of translation, leading to protein misfolding [22, 24]. Two sSNVs in *NKX2-5*, identified in patients with congenital heart disease, decreased the mRNA's transactivation potential [64]. In hemophilia B, an sSNV in the factor IX gene affected the transcript's secondary structure and reduced extracellular protein levels [65], and both synonymous and nonsynonymous variants were shown more likely be deleterious when occurring in stable regions of *F8* and *DMD* mRNAs [66]. Our understanding of the role of synonymous variants in cancer is rapidly expanding, with recent studies demonstrating that they may act as drivers of the disease [67–69], altering the function of oncogenes such as *RET* [70] and *KRAS*[71].

While there are numerous methods to predict the impact of amino acid altering and regulatory variation, relatively few approaches have been developed to identify functional sSNVs. Of the 5 synonymous variant metrics we found in the literature, only 2 use RNA-folding statistics—SiLVA [72] and DDIG-SN [73]—and in each case the authors emphasize that the structural features make almost no difference to the model. These scores primarily excel at identifying splicing defects, and the same is true for other synonymous scores such as IDSV [74], regSNPs-splicing [75], and Syntool [76]. There are, in contrast, tools that measure disruptions of RNA folding, albeit not exclusively in synonymous variants—the 3 most prominent are the webservers RNAsnp [77], SNPfold [59], and MutaRNA [78]. These 3 webservers perform largely the same task, comparing predicted wild-type and mutated structures and returning the change in base-pairing probabilities and or/visualizations of the structures themselves. However, the 3 tools are limited to the assessment of a single variant, requiring an on-the-fly calculation for every SNV under consideration, making them unsuitable for scoring sSNVs in the 4–6 million variants typically identified from genome sequencing of a single individual. To the best of our knowledge there are no precalculated transcriptome-wide scores well equipped to model sSNVs that specifically alter RNA structure.

Given the established importance of RNA structure, we hypothesize that there may be many more as yet to be identified sSNVs that can provoke genetic disorders through their disruption of RNA structural elements. As such, the aims of this study were the creation of RNA-structural metrics for every possible single-nucleotide variant (SNV) and to evaluate whether structure-disrupting sSNVs are constrained in the human population. Through developing methods to predict whether an SNV is “structurally pathogenic,” we hope to drive the discovery of novel genetic etiologies in both monogenic genetic disorders and more complex human disease.

## Data Description

### Raw dataset

To obtain all human mRNA transcripts we downloaded the NCBI RefSeq Release 81 from an online repository [79]. Transcript sequences corresponded to human reference genome build GRCh38.

### Massively parallel generation of RNA stability metrics

**Figure 2:**
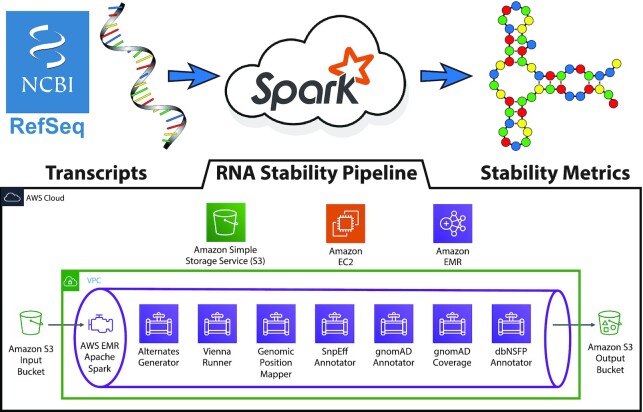
Graphical depiction of computational workflow used to generate ViennaRNA-folding metrics for the entire transcriptome. The entire analysis workflow was parallelized using Apache Spark and the Amazon Elastic Map Reduce (EMR) service, generating 5 billion ViennaRNA metrics over the course of 2 days. Using a custom pipeline developed for the process that was executed across 47 Amazon Elastic Cloud Compute (EC2) spot instances, input data were retrieved from an Amazon Simple Storage Solution (S3) bucket and processed through the pipeline consisting of 8 steps. We first obtained the 101-base sequence centered around an SNV in a transcript and generated 3 alternate sequences (with the ALT rather than the REF at position 51) (Step 1). We next applied ViennaRNA modules to sequence to obtain structural metrics (Step 2). Results were then mapped to chromosomal coordinates (Step 3) and annotated with SnpEff to identify splice variants (Step 4), annotated with gnomAD population frequencies (Step 5) and coverage information (Step 6), and finally annotated with metrics from dbNSFP (Step 7). Final dataset was written to Amazon S3 in Parquet columnar file format for further analysis and interpretation.

To assess the impact of synonymous mutations on mRNA structure, we carried out a genome-wide computation in which folding statistics were calculated for every possible variant in the human transcriptome (RefSeq Release 81, GRCh38). For each position in all transcripts, we built a 101-base window centered around the reference and 3 alternate sequences with the alternate allele substituted at the 51st position. We applied the ViennaRNA software package to the wild-type and mutated sequences to obtain 10 folding metrics quantifying the structural disruption caused by all 3 possible SNVs at the position (see Supplementary Table S1 for metric details). Computing this dataset of structural predictions for nearly half a billion SNVs was truly a “big data” computational task. We relied heavily on the parallelizability of the Apache Spark framework and custom wrappers that adapted the ViennaRNA software package to run within the Hadoop framework (Fig. 2). Details of the calculation and subsequent assignment of variants into classes are given in Methods.

Of the 10 mRNA-structural metrics output by our Vienna implementation, we adopted 3 as central to our analysis: Δ minimum free energy (ΔMFE), centroid edge distance (CED), and Δ centroid distance (ΔCD). The metric ΔMFE measures the change in mRNA free energy or “stability” caused by the sSNV, while CED gives the number of base pairs that vary between the mutant and wild-type centroid structures. The metric ΔCD measures the sSNV's effect on the diversity of the mRNA's structural ensemble, which is the collection of various structures that a given sequence can exhibit. Distributions of these metrics, along with the other 7 mRNA-structural metrics output by our RNA structure pipeline, are presented in Supplementary Fig. S1.

To test whether certain sSNVs are under constraint due to their effect on mRNA structure, we used population frequencies from the Genome Aggregation Database (gnomAD) containing aggregate genome and exome sequencing data from a total of 201,904 unrelated human individuals (gnomAD v2.1 dataset contains data from 125,748 exomes mapped to the GRCh37/hg19 reference sequence and lifted over the GRCh38; the gnomAD v3.1 dataset contains 76,156 whole genomes [and no exomes], all mapped to the GRCh38 reference sequence) [80]. Our expectation was that SNVs with disruptive structural properties would be found less frequently in human populations. We defined a variant to be constrained if it was absent from both gnomAD v2.1 and v3.1 datasets and unconstrained if it had a MAF > 0 in either set, a strategy similar to that used by other groups [81, 82].

## Analysis

### Global constraint to maintain stability

**Figure 3:**
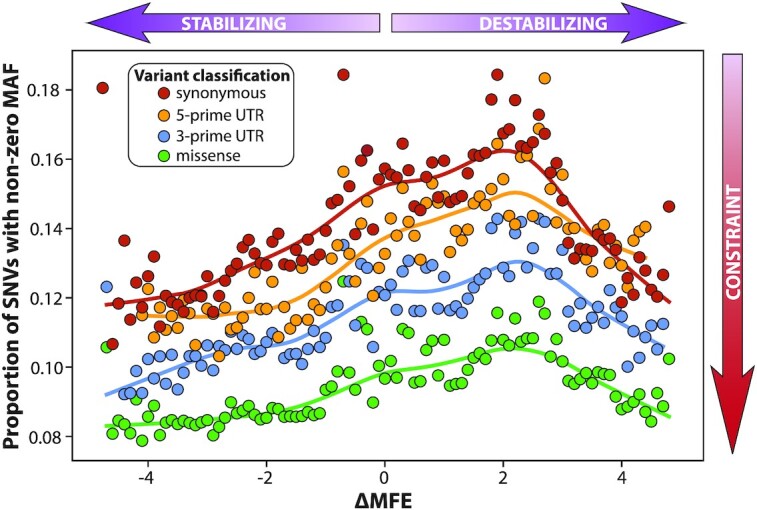
Exonic SNVs predicted to affect mRNA structure are constrained in the human population. Population frequency of SNVs was plotted against predicted impact on mRNA structure. Circles show proportion of SNVs with nonzero gnomAD exonic frequency at each value of the RNA stability metric ΔMFE. The bell-shaped pattern of constraint was observed across all classes of SNVs, with constraint appearing to be greatest in sSNVs (red), followed by SNVs in the 5′ UTR (orange), then SNVs in the 3′ UTR (blue), and finally nsSNVs (green). Values of ΔMFE with <2,000 (synonymous), 200 (UTRs), or 5,000 (missense) positive-MAF sSNVs are excluded. Only SNVs passing all filters for both WGS and WES data are represented (see Methods for details).

Our study reveals a striking connection between a given SNV's impact on mRNA structure and its frequency in the gnomAD database. This central finding is summarized in Fig. 3, which depicts the proportion of SNVs with gnomAD MAF > 0 at every value of our stability-metric ΔMFE. All 4 variant classifications—synonymous, 5′ UTR, 3′ UTR, and missense—show a bi-directional constraint to maintain the wild-type mRNA structure. When the SNV either weakens the mRNA structure (high ΔMFE) or strengthens it (low ΔMFE) the SNV is depleted in the population roughly in proportion to the level of disruption. While this pattern of constraint was observed across all 4 variant classes, Fig. 3 indicates that it is strongest for synonymous variants..

**Figure 4:**
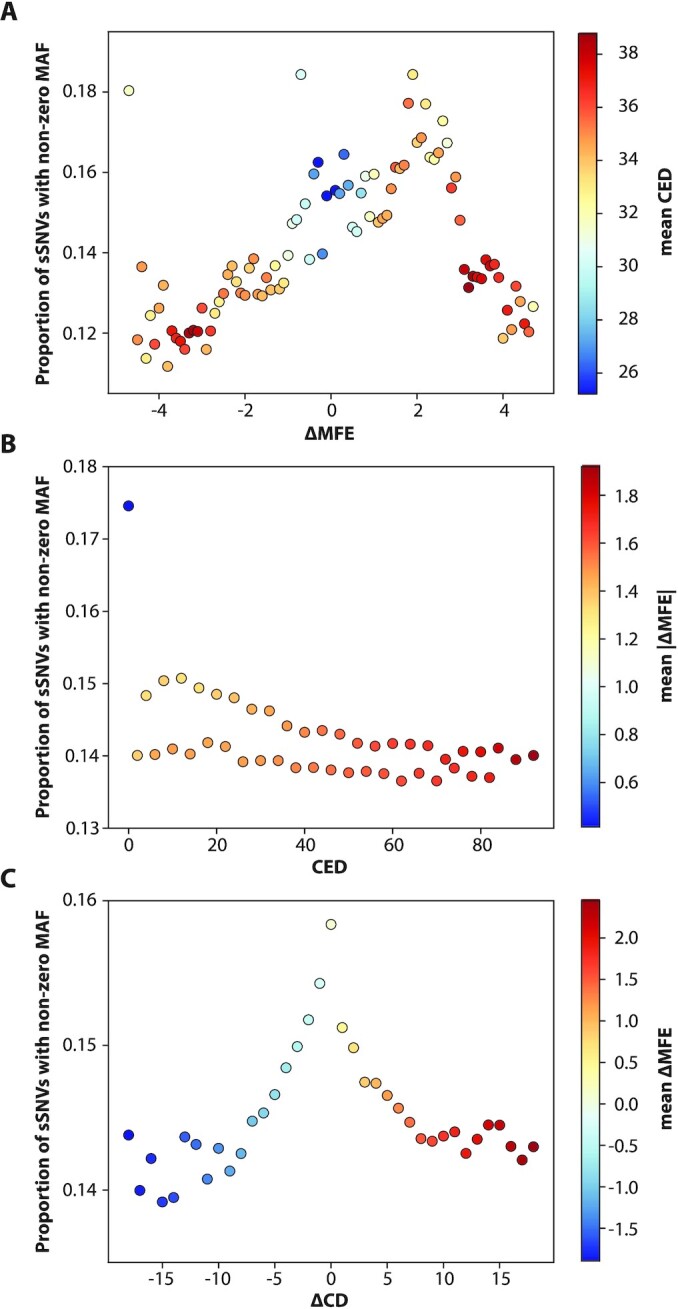
Synonymous variants predicted to affect mRNA structure are constrained in the human population. Population frequency of sSNVs was plotted against the predicted impact on mRNA structure. Synonymous variants that disrupt structure tend to be absent from the gnomAD database, while those with limited impact on structure appear at least once in the gnomAD database. **(A)** Proportion of sSNVs with nonzero gnomAD frequency at each value of the RNA stability metric ΔMFE. Color represents average CED value, to highlight the relationship between minimum free energy and edit distance. **(B)** Analogous plot for metric CED measuring edge differences between mutant/wild-type centroid structures. Color represents |ΔMFE|, measuring absolute change in stability. **(C)** Analogous plot for diversity-metric ΔCD measuring change in structural ensemble diversity due to sSNV. Color is by ΔMFE measuring change in stability. Metric values with <2,500 (ΔMFE), 7,500 (CED), or 3,500 (ΔCD) positive-MAF sSNVs excluded.

Figure 4 summarizes constraint in the synonymous case, showing the relationship of our 3 main structural metrics with gnomAD frequency. Figure 4A recapitulates the pattern of green circles in Fig. 3, revealing that disrupting mRNA stability decreases the chance of a synonymous SNV's appearing in human mRNA transcripts. The global peak at ΔMFE = 2 reflects the dominant contribution of CpG transitions, which tend to be destabilizing—see Analysis: CpG transitions have constraint against destabilization of their mRNA structures. The effect of removing or creating new base-pairings, quantified by the metric CED, is shown in Fig. 4B (see Supplementary Fig. S2 for an illustration of how CED is calculated). This figure validates our basic hypothesis that structurally disruptive sSNVs should appear less frequently in the population. We see that sSNVs that leave the centroid structure unchanged (i.e., CED = 0) are ∼15% more common than those sSNVs predicted to alter it, and SNVs with large CED values are constrained in proportion. Our third metric ΔCD measures change in the diversity of the mRNA ensemble (i.e., collection of all the structures formed by millions of *in vivo* mRNAs) and is shown in Fig. 4C. This figure illustrates that changes in diversity—towards either more or less—are also constrained in gnomAD. The symmetry in depletion between over- and under-diversifying sSNVs is surprisingly regular. Analogous plots for the remaining 7 structural metrics can be viewed in Supplementary Fig. S3.

The color coding in Fig. 4 illuminates the relationship between the 3 structural metrics. Changes in stability are correlated with changes in base-pairing and vice versa, as demonstrated by the red values at the extremes of each distribution. Fig. 4C depicts a clear relationship between diversity and stability, with those sSNVs that diversify the ensemble (high ΔCD) also tending to weaken it (red). This diversity-instability relationship is intuitive, as a destabilizing mutation “frees up” portions of the mRNA to assume new shapes.

### Variation of constraint with REF>ALT context

We next set out to determine whether the constraint demonstrated in Fig. 4 holds uniformly for all synonymous nucleotides or whether it varies in different REF>ALT contexts. We would expect the latter because the bases C and G form much stronger structural bonds than do A and T. To probe this question we divide our sSNVs into 14 classes (Table 1): 12 classes based on their reference and alternate mRNA alleles (e.g., A>C, C>G, T>C) and 2 additional classes based on potential loss of methylated cytosine (CpG>TpG or CpG>CpA, the latter of which results from a deamination on an antisense strand). For consistency and clarity, we treat thymine as an mRNA base, even though it is actually replaced by uracil in mRNA. Then within each REF>ALT context we reconstruct the 3 plots of Fig. 4 and also perform weighted linear (or quadratic, for ΔCD) regressions between the 3 different stability metrics and the probability that the gnomAD MAF > 0 (see Methods for details and Supplementary Table S2 for full regression statistics).

**Table 1:** Structural metrics correlate with gnomAD frequency in most REF>ALT contexts

**Context**	**Constrained against**	***R* ^2^**	***P*-value**	**Mediator**	**Proportion of variance explained by mediator**
**ΔMFE—Structural stability constraint**					
CpG>CpA	Weaker structure	0.683	5.23e−69	−CpG content	0.769
CpG>TpG	Weaker structure	0.482	2.43e−45	−CpG content	0.746
C>G	Weaker structure	0.154	1.72e−29	+Trailing G	0.156
G>T	Weaker structure	0.136	4.02e−22	+Leading C	0.317
C>T	Weaker structure	0.125	1.67e−20	−Leading G	0.134
T>C	Both	0.117*	5.97e−18*	+Leading A	0.343
C>A	Weaker structure	0.087	1.73e−16	+Trailing G	0.332
G>A	Stronger structure	0.035	1.64e−06	−Trailing A	0.241
A>G	Stronger structure	0.030	1.11e−05	+Trailing T	0.335
G>C	Weaker structure	0.018	0.000286	+Leading C	0.227
A>C	Stronger structure	0.011	0.00296	+Leading C	0.064
**CED—Base-pairing constraint**					
CpG>CpA	Base-pair alteration	0.606	5.56e−15	−CpG content	0.787
C>A	Base-pair retention	0.396	2.51e−09	+CodonBase2 = G	0.506
G>A	Base-pair retention	0.352	3.2e−08	−Trailing A	0.607
CpG>TpG	Both	0.388*	3.79e−08*	−CpG content	0.563
T>C	Base-pair alteration	0.240	8.33e−06	+CodonBase2 = A	0.666
C>T	Base-pair alteration	0.196	9.8e−05	+C content	0.414
G>C	Base-pair alteration	0.161	0.000444	+Leading C	0.386
**ΔCD—Diversity Constraint**					
CpG>CpA	Diversity changes	0.650	1.57e−14	−CpG content	0.849
G>A	Diversity maintenance	0.482	1.07e−09	−Trailing A	0.621
CpG>TpG	Diversity changes	0.336	3.81e−06	+A content	0.418
C>A	Diversity maintenance	0.278	6.71e−06	+Trailing G	0.443

Correlation between structural metrics ΔMFE, CED, and integer-rounded ΔCD on the one hand, and the quantity *P*(MAF > 0) on the other, over all sSNVs in a given context. The *R*^2^ and *P*-values are obtained from a weighted least-squares linear regression, with the *P*-value corresponding to the linear coefficient; a quadratic regression was also performed, but only the *P*-value was retained as denoted by an asterisk. Only context-metric pairs with *P* < 0.005 are included. “Normalized slope” was obtained by dividing slope of regression line by average *P*(MAF > 0) in the context and then multiplying by range covered by metric in its central 90% of sSNVs. “Mediator” is raw sequence variable that explains largest proportion of structural trend in this context, with sign adjusted to correlate positively with gnomAD frequency. “Mediator *R*^2^” gives proportion of variance explained by the mediator (see Mediator variables in Results for details).

We observe that constraint for mRNA structure is highly dependent on mutational context (Table 1). Some REF>ALT contexts show constraint in 1 direction only (e.g., against weakening of their structures), while other contexts show no significant constraint at all. The metric ΔMFE, which measures changes to mRNA energy or stability, shows a striking context dependence (Table 1). All significant REF>ALT changes are constrained unidirectionally, with 1 direction showing a depletion in population frequencies while the other shows an enrichment (the direction of constraint is obtained by a weighted linear regression; see Methods for details). In line with our understanding of the structural biochemistry of RNA folding, mutations from “strong” REFs (C and G, so called because they form strong Watson-Crick bonds) to “weak” ALTs A and T are constrained against high values of ΔMFE, i.e., against the weakening of structure. Conversely, mutations from weak to strong nucleotides are constrained against the strengthening of structure (low ΔMFE). The exception to this rule is the context G>A (see section Constraint for mRNA stability in non-CpG-transitional contexts).

Evaluation of the base-pair metric CED demonstrates that some contexts are constrained against large changes in mRNA base-pairing, while in others, SNVs altering base pairs are actually enriched (Table 1). This result reflects the fact that in some contexts small base-pairing changes are enriched over *no* base-pairing changes. In keeping with our main hypothesis, large changes of base-pairing are still uniformly constrained. As was the case with ΔMFE, we again observe that the context G>A is the exception.

Finally, the bottom section of Table 1 shows mutational contexts that exhibit significant constraint against changes to ensemble diversity as measured by ΔCD. We see that only a few contexts exhibit this constraint. In the 2 CpG-transitional contexts, the bell-shaped pattern of Fig. 4C is faithfully reproduced, with both decreases and increases to ensemble diversity being equally harmful. However, the context G>A is enriched for changes in diversity—this context is strangely aberrant when assessed with all 3 metrics.

### CpG transitions have constraint against destabilization of their mRNA structures

The data in Table 1 show that our observed constraint for mRNA structure is greatest in the case of CpG transitions. Because these variants (and their suppression) are crucial to the story of mRNA stability, it is important to have an appreciation of their role in a biochemical context. The dinucleotide CG (usually denoted CpG to distinguish this linear sequence from the CG base-pairing of cytosine and guanine) is capable of becoming methylated and then mutating by a process called “deamination” into a TG dinucleotide; deaminations are also possible in unmethylated CpGs, but these result in a uracil that is quickly identified as a foreign base and repaired. In mammals 70–80% of CpGs are methylated, which makes a CpG transition ∼4× more common than any other mutation type among mammals (see Supplementary Table S3) [83]. The nucleotides C and G also form foundational bonds in mRNA secondary structures. Most of the energy of an mRNA structure lies in its “stacks” of nucleotides, with the average energy of a C-G pair in a stack ∼65% stronger than that of any other base-pairing [84].

**Figure 5:**
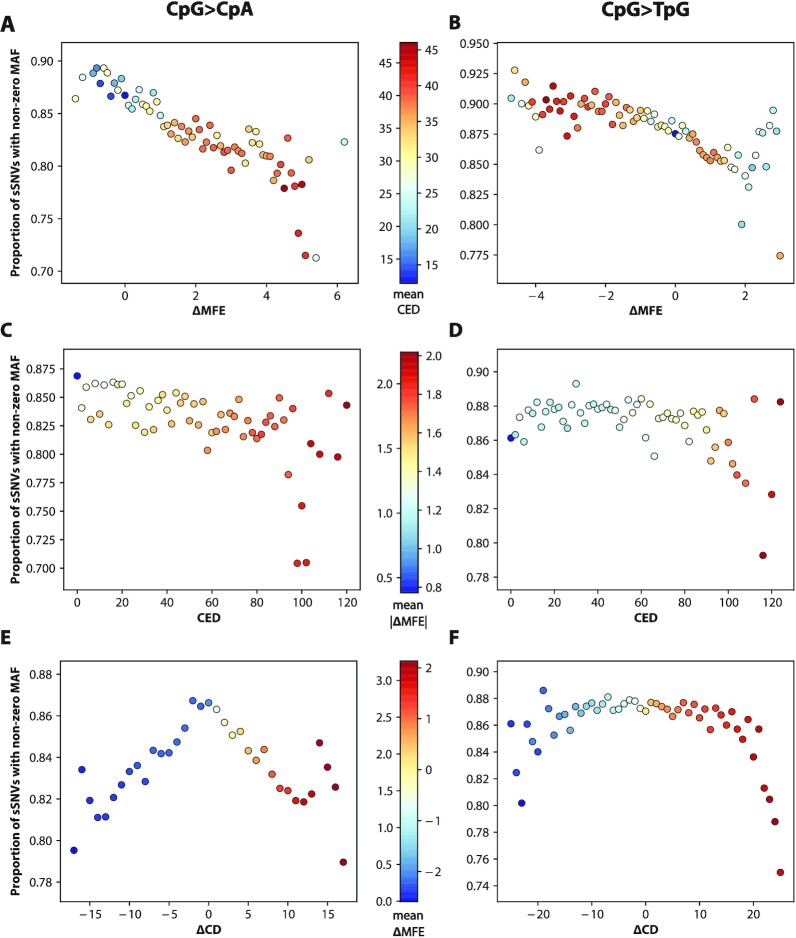
Synonymous CpG transitions are markedly constrained against destabilization of their mRNA structures. Population frequency of sSNV vs effect on mRNA structure in synonymous CpG transitions was examined. Proportion of synonymous CpG transitions with nonzero MAF at each value of ΔMFE were determined for **(A)** CpG>CpA and **(B)** CpG>TpG synonymous mutations. ΔMFE values with <75 nonzero-MAF sSNVs are excluded. Color gives average CED in each context, ranging from 15 (blue) to 50 (red). Similarly, proportion of synonymous CpG transitions with nonzero MAF at each value of CED were determined for **(C)** CpG>CpA sSNVs and **(D)** CpG>TpG sSNVs. Color represents average ΔMFE and ranges from −0.8 (blue) to 1.85 (red). CED values with <40 (CpG>CpA) or 75 (CpG>TpG) nonzero-MAF sSNVs are excluded. Finally, proportions of synonymous CpG transitions with nonzero MAF at each value of ΔCD (after rounding to nearest integer) were determined for **(E)** CpG>CpA and **(F)** CpG>TpG sSNVs. Color represents average ΔMFE and ranges from −3 (blue) to 4 (red). Rounded ΔCD values with <250 (CpG>CpA) or 20 (CpG>TpG) nonzero-MAF sSNVs are excluded.

We find strong evidence that CpG transitions are constrained against weakening of their mRNA structures. This striking trend is largely explained (in a statistical sense) by CpG content, i.e., number of CpG dinucleotides in the vicinity (see “Proportion of variance explained by Mediator” in Table 1). Figure 5 shows the populational constraint for our 3 main metrics in CpG-transitional contexts. Most strikingly, we find that synonymous CpG>CpA and CpG>TpG mutations both show a steady constraint against weakening of mRNA structure (high ΔMFE) (Fig. 5A and B). Fascinatingly, both contexts exhibit a cluster of outliers in the most destructive (i.e., most destabilizing) region, suggestive of extreme constraint borne of significant structural disruption.

The behavior of the edge metric CED in these contexts is also clear-cut. In Fig. 5C and D we see a clear constraint against mutations with high CED values, and the red coloring shows that such changes are, on average, destabilizing. We also observe a depletion at CED = 0 in the CpG>TpG case; this is responsible for the bidirectional constraint reported in Table 1. Finally, Fig. 5E and F show that the basic pattern of constraint for diversity in Fig. 4C is reproduced and is essentially unchanged for both types of CpG transition. The coloring of Fig. 5 indicates that mutations CpG>CpA are more weakening on average than their CpG>TpG counterparts, despite being largely produced by the same biochemical mechanism (a CpG>TpG deamination on either the sense or anti-sense strand). We speculate on this disparity in the Discussion.

### Constraint for mRNA stability in non-CpG-transitional contexts

We observe a constraint for mRNA structure in most REF>ALT contexts (as indicated by Table 1). We can classify the remaining contexts on the basis of whether they are constrained against weakening or strengthening of their structures (as reported in the top section of Table 1). Supplementary Fig. S4 shows plots of contexts where ΔMFE and gnomAD frequency are negatively correlated, i.e., where structure-weakening sSNVs are under constraint. Notably, all these contexts are strong>weak (or strong>strong in the case of C<>G), consistent with the principle that 1 purpose of such nucleotides is to maintain stability. In Supplementary Fig. S5 we show the contexts where ΔMFE and gnomAD frequency vary positively, which amounts to constraint against structure-strengthening sSNVs. Correspondingly, we note that 2 out of 3 of these contexts are weak>strong (and the third is the consistently aberrant context G>A).

### Mediator variables

In Table 1 we provide a “Mediator” variable for the connection between our RNA-folding metrics and gnomAD frequencies in each mutational context. The name “Mediator” signifies that the variable explains some of the connection between the structural metric and gnomAD (details on how the Mediator and percent variance explained are calculated are given in Methods.) These Mediators can explain large portions of the trends in Fig. 5 and Supplementary Figs S4 and S5. The striking trend between ΔMFE and gnomAD frequency in CpG-transitional contexts, for example, is largely driven by the local CpG content. CpG content is also the most powerful feature for CED and ΔCD in these contexts, with high CpG content consistently correlating with depletion. A plausible inference is that an abundance of CpGs signifies important mRNA structure whose disruption could be harmful.

In non-CpG-transitional contexts, the Mediator almost always proves to be a nucleotide upstream or downstream of the sSNV. In the context C>A we can recover 28% of the relationship between ΔMFE and gnomAD frequency simply by looking at whether the C is followed by a G. The power of CpG dinucleotides in recovering our structural trends emphasizes the effect of these dinucleotides on mRNA structure.

### Global quantification of mRNA constraint

Our analysis shows that variants predicted to disrupt mRNA secondary structures are constrained in the population. However, the complexity of mRNA structure means that focusing on 1 single metric will surely lead to loss of information. To overcome this potential limitation of our RNA-folding metrics, we set out to devise a more comprehensive method for predicting possible pathogenicity due to mRNA structure. Our strategy is to consider the additional statistical power bestowed by mRNA structure. In each context from Table 1 we use RNA-sequence features (such as nearby bases and transcript position) to construct 2 separate models to estimate the probability that an sSNV will appear in gnomAD: an “active” model that incorporates our mRNA-structural metrics (*P_s_*) and a null model that only uses sequence features (*P_n_*). These models give us 2 separate estimates for the quantity *P*(MAF > 0). Then we define the Structural Predictivity Index (SPI) to be the log-quotient of the 2 probabilities:

\begin{equation*} {\mathrm{SPI\,\,}} = {\mathrm{\,\,lo}}{{\mathrm{g}}_{10}}\left( {\frac{{{P_s}}}{{{P_n}}}} \right). \end{equation*}

**Figure 6:**
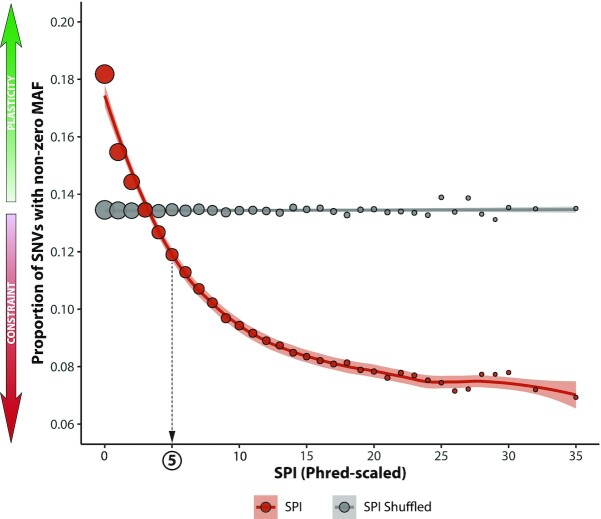
SPI score correlates with constraint in synonymous variants. Variants are grouped by Phred-scaled SPI integer values into 33 bins, with the number of sSNVs per bin ranging from ∼1,000,000 (large circles) to ∼5,000 SNVs (small circles). The corresponding value of *P*(MAF > 0) was plotted against the Phred-scaled SPI score of each bin (red circles) and fitted with a smoothed loess curve (red line). A clear correlation between global constraint and increasing score can be observed, with all scores ≥5 (our suggested minimum cut-off, dashed arrow) demonstrating constraint in *P*(MAF > 0) below that of the average seen in sSNVs globally (grey line). To assess the power of this correlation as compared to random chance, SPI scores were randomly shuffled and the MAF distribution of the shuffled SPI scores plotted (grey circles). Across all Phred-scaled SPI bins, the *P*(MAF > 0) for the shuffled data remains at or close to the expected global average of 13.8%, calculated for all 17 million sSNVs that had sufficient coverage in gnomAD to determine MAF. This clearly demonstrates that sSNVs' high Phred-scaled SPI scores are constrained (red arrow), while those with a low score demonstrate greater plasticity (green arrow), with an increased probability of MAF > 0. Shaded area represents 90th percentile confidence intervals for both SPI (red) and shuffled SPI (grey).

The metric SPI thus measures the predictive power bestowed by mRNA-structural variables. When it varies from 0, mRNA-structural metrics yield new insight about an SNV's potential to play a functional role in mRNA secondary structure. The variation of gnomAD MAF with respect to SPI can be seen in Fig. 6. We observe uniform constraint in SPI, validating the structural score ${P_s}$: when ${P_s}$ is relatively low, SNVs are depleted; when it is relatively high, SNVs are enriched. This global relationship between SPI and constraint is also evident across all 14 sequence contexts (Supplementary Fig. S6). We show the power of SPI in each sequence context (given by its area under the curve in predicting whether gnomAD nonzero frequency is >0) in Supplementary Table S4.

**Figure 7:**
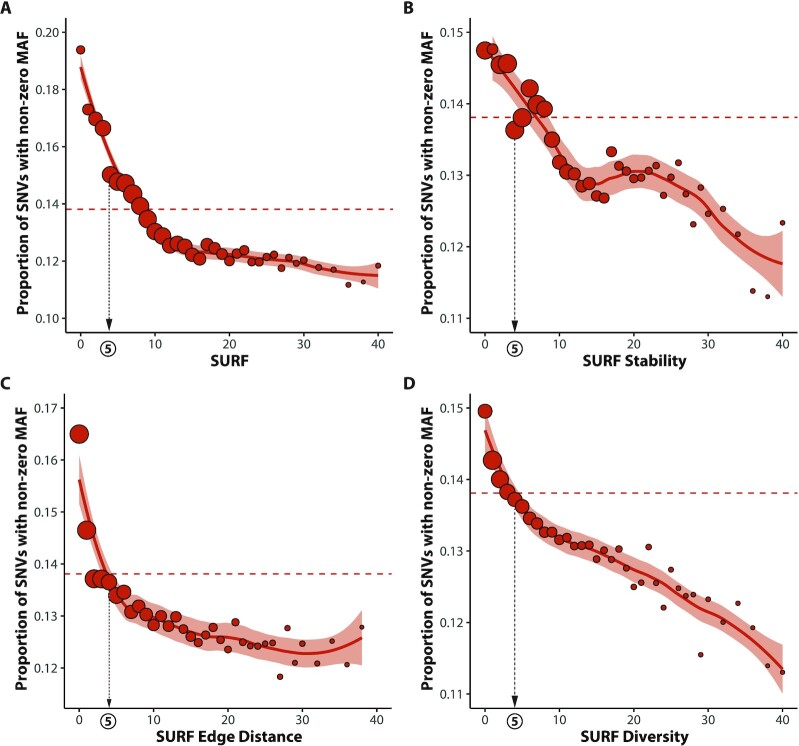
SUmmarized RNA Folding (SURF) metrics correlate with constraint in synonymous variants. SPI and each of the 10 RNA-folding metrics were percentile ranked and Phred-scaled [−10 × log_10_(rank)], such that the larger the Phred-scaled value the greater the predicted change in RNA structure. For each SNV in our dataset, the maximum Phred score was determined across **(A)** all 11 metrics—SURF, **(B)** the 4 stability metrics (ΔMFE, ΔCFE, ΔMEAFE, and ΔEFE)—SURF Stability, **(C)** the 4 edge distance metrics (MFEED, CED, MEAED, and EED)—SURF Edit Distance, or **(D)**—the 2 diversity metrics (ΔCD and ΔEND)—SURF Diversity. For each plot, variants are grouped by integer values into 36 bins (ranging from 0 to 40, i.e., the 99.99th percentile). The corresponding value of *P*(MAF > 0) was plotted against the SURF metric for each bin (red circles) and fitted with a smoothed loess curve (red line). Shaded area represents 90th percentile confidence intervals for the given summary metric. Dashed red line indicates the average *P*(MAF > 0) value of 13.8% seen in sSNVs globally. The dashed arrow indicates our suggested minimum cut-off of 5 for any given metric. Across all 4 summarized metrics, a clear correlation between global constraint and increasing score can be observed.

Finally, to simplify use of our RNA stability dataset we calculated SUmmarized RNA Folding (SURF) metrics. For each of the 10 RNA-folding metrics and SPI, scores were percentile ranked and Phred-scaled [$- 10\,\, \times \,\,{\mathrm{lo}}{{\mathrm{g}}_{10}}( {\mathrm{Percentile}\,\,{\mathrm{Rank}}} )$], such that the larger the Phred-scaled value the greater the predicted change in RNA structure. For each SNV in our dataset, the maximum Phred score was calculated either across all 11 metrics (SURF), across the 4 stability metrics (SURF Stability), across the 4 edge distance metrics (SURF Edit Distance), or across the 2 diversity metrics (SURF Diversity). Across all 4 summarized metrics, a clear correlation between global constraint and increasing score can be observed (Fig. 7).

### Clinical examples of structural pathogenicity

The literature reveals only a few examples of synonymous SNVs unequivocally shown to be pathogenic through their effects on mRNA structure. These sSNVs, with accompanying values of our 3 ViennaRNA metrics, SPI, and SURF, are listed in Table 2. This set of known pathogenic sSNVs show a clear enrichment for our structural metrics, with each exhibiting a value of ΔMFE, CED, ΔCD, or SPI that is in the third quartile of distribution for the given score. All 9 SNVs had a damaging SURF score, ranging from 9.5 to 18.6 (the 89th to 99th percentile). For example, 1 pathogenic sSNV in *NKX2–5* (rs2277923), linked to congenital heart disease, has a SURF score in the 90th percentile [64]. It should be noted that none of these clinical sSNVs qualifies as a truly exceptional outlier for any of our ViennaRNA metrics or SPI; while all have SURF scores above the 89th percentile, none exceed the 99th percentile (see Discussion for suggested score cut-off values).

**Table 2:** Known sSNVs clinically implicated for structural pathogenicity are successfully predicted to be pathogenic by our structural metrics.

					**Phred score[raw value] (percentile value)**			
**Gene**	**Condition**	**SNP (hg38 / GRCh38)**	**Context**	**SURF**	**SPI**	**ΔMFE**	**CED**	**ΔCD**
*COMT*	Pain Sensitivity	rs4633NC_000022.11:g.19962712C>T M_000754.3:c.186C>T NP_000745.1:p.His62=	CpG>TpG	9.7(CED)	3.8 [0.28] (58%)	2.9[-0.5](31%)	9.7[66](89%)	4.6[-4.0](19%)
*COMT*	Pain Sensitivity	rs4818NC_000022.11:g.19963684C>G NM_000754.3:c.408C>G NP_000745.1:p.Leu136=	C>G	10.1(ΔMFE)	4.0[-0.16](60%)	10.1[-3.0](6%)	5.0[38](68%)	7.4[-7.0](10%)
*DRD2*	Schizophrenia, substance abuse	rs6277 NC_000011.10:g.113412737G>A NM_000795.4:c.957C>T NP_000786.1:p.Pro319=	CpG>TpG	18.6(ΔMEAFE)	3.1[0.35](51%)	4.0[1.0](75%)	8.4[60](86%)	9.8[9.5](94%)
*F2*	Thrombosis	rs72554028 NC_000011.10:g.46739363C>T NM_000506.4:c.1824C>T NP_000497.1:p.Arg608=	C>T	15.9(MFEED)	1.4[0.61](28%)	5.9[-1.7](15%)	11.2[72](92%)	5.1[-4.5](17%)
*KRAS*	Cancer	NA NC_000012.12:g.25245355T>G NM_033360.3:c.30A>C NP_203524.1:p.Gly10=	A>C	10.2(EED)	2.8[0.11](48%)	0.5[0](49%)	5.2[40](70%)	3.3[2.5](74%)
*NKX2-5*	Congenital heart disease	rs72554028 NC_000005.10:g.173233001C>T NM_004387.4:c.543G>A NP_004378.1:p.Gln181	G>A	12.6(ΔEFE)	4.0[-0.18](60%)	11.5[3.5](96%)	1.1[4](22%)	0.2[0.0](50%)
*NKX2-5*	Congenital heart disease	rs2277923 NC_000005.10:g.173235021T>C NM_004387.3:c.63A>G NP_004378.1:p.Glu21=	A>G	9.8(ΔEND)	1.9[0.41](36%)	0.5[0.0](49%)	2.9[20](49%)	9.3[9.0](94%)
OPTC	Primary open angle glaucoma	rs559635109 NC_000001.11:g.203498796C>T NM_014359.3:c.486C>T NP_055174.1:p.Phe162=	C>T	9.5(ΔCFE)	5.7[-0.57](73%)	0.5[0.0](49%)	4.3[32](63%)	3.3[2.5](74%)
TP53	Cancer	rs748527030 NC_000017.11:7676528:T>C NM_000546.5:c.66A>G NP_000537.3:p.Leu22=	A>G	13.1(ΔCD)	0.1[1.95](2%)	2.2[-0.2](36%)	2.9[20](49%)	13.1[12.5](97%)

dbSNP RS number and standardized SNV annotations are provided, along with the gene’s official symbol and disease the sSNV has been associated with. SURF scores are shown, along with the metric that produced that score (i.e., for the first sSNV in the table, the highest Phred-scaled value across all 11 metrics was of 9.7, observed with the CED metric). PPhred scores for SPI, ΔMFE, CED and ΔCD are also provided, along with the metrics raw value [middle] and percentile value (bottom). For all scores, the greater the Phred-scaled value, the greater the predicted change to the RNA structure. All clinically implicated sSNVs were predicted to be damaging according to SURF score, and had one or more individual stability metrics with a score greater than 5 (our suggested minimum cutoff, representing the 3rd quartile for the metric).

## Discussion

We developed novel software to enable efficient generation of billions of RNA-folding metrics for any species. This software allowed us to calculate RNA-folding metrics for every base in the human transcriptome (∼0.5 billion SNVs). The RNA stability scores generated by this approach enable global assessment of synonymous variants and their potential role in human health and disease. We focused our analysis on the ∼21 million synonymous variants found in the transcriptome, avoiding those sSNVs that could affect canonical splice sites and confound our analysis. Our study revealed that there is significant selection against sSNVs predicted to disrupt the given transcript's local mRNA secondary structure, supporting our hypothesis that RNA structure itself plays a critical role in human health and disease.

Multiple arguments support a true causal relationship behind RNA stability and the observed correlation with constraint in the human population. First, we tested our hypothesis using 3 qualitatively distinct measures of structural disruption: change in stability (ΔMFE), change in base-pairing (CED), and change in ensemble diversity (ΔCD). All 3 metrics showed that SNVs that alter mRNA structure are constrained in human populations.

Second, our study revealed some patterns that can be elegantly explained in terms of mRNA structure. We showed that strong>weak mutations such as C>A are only depleted when they weaken mRNA structure, while weak>strong mutations are only depleted when they strengthen it. We also found that sSNVs with extreme ΔMFE and CED values are constrained even beyond the general trends (Fig. 5), suggesting that this severe disruption is more-than-linearly unviable. Furthermore, Fig. 4B highlights a pattern in CED values that alternates between high and low on successive values (CED can only take on even values because the destruction/creation of a base pair always requires 2 edits): the sSNVs with CED values that were multiples of 4 (4, 8, 12…) were shown to be enriched over those that were only multiples of 2 (2, 6, 10…). Such CED values are required if the total number of base pairs is to be conserved, supporting that the constraint is needed to maintain overall base-pairing.

Third, the structural constraint that we observe is not just restricted in Watson-Crick base pairs, but also in nucleotides where wobble base-pairing occurs. Wobble base-pairing takes place between 2 nucleotides such as guanine-uracil (G-U) that are not canonical Watson-Crick base pairs but have comparable thermodynamic stabilities. We observed bidirectional constraint for ΔMFE in the context T>C, viewable in Supplementary Fig. S5. We conjecture that the dual constraint in this context might be due to guanine's unique ability to wobble base-pair. Thus, the dual constraint from mutations T>C could be related to the transformation of T = G wobble base pairs into stronger C = G Watson-Crick base pairs.

Finally, our SPI, created specifically to control for all confounding factors, demonstrates a clear relationship between mRNA structure and constraint. When structural metrics decrease the model score, the gnomAD MAF is lower, whereas when structural metrics increase the model score, gnomAD MAF is higher (Fig. 6). This strongly suggests that our trends are direct and causal. This “proof of non-spuriousness” justifies our decision to regard sequence variables that contribute to mRNA structure—such as adjacent nucleotides and GC/CpG content—as Mediators (Table 1).

That many of the Mediators are adjacent nucleotides—“leading C,” “trailing G,” and so on—suggests that the reference and Mediator are set next to one another in a stable “stack,” such stacks being the principal feature of mRNA structures. Our data show that these stacks are more likely to be enriched for mutations, not depleted; suggesting that a strong structure has more tolerance to be destabilized, whereas a weak structure cannot. The trend operates in the other direction too, with weak features like “leading A” and “trailing T” featuring mainly in W (A or T) > S (G or C) contexts—as if the less existing structure, the less the danger of being overstabilized. Relatedly, several of the Mediators simply create a CpG—in view of the hyper-mutability and structural sturdiness of CpG dinucleotides, it seems inevitable that they should explain some of our trends. However, CpGs do not explain the appearance of Mediator As and Ts in the W>S contexts, nor do they account for the bidirectional constraint that we observe in ΔMFE and ΔCD in Figs 3 and 4. Regardless, in view of the deep connections linking CpG status and all the other Mediators to both mutability and RNA structure, an ensemble approach such as SPI is perhaps the best way of isolating the structural contribution of any given SNV.

### Successful identification of structurally disruptive sSNVs in known pathogenic synonymous variants

Over the past decade numerous studies have demonstrated that synonymous variants play essential molecular roles in regulating both mRNA structure and processing, including regulation of protein expression, folding, and function [reviewed in 9, 85, 86]. However, the potential for pathogenic synonymous variants that affect RNA folding in human genetic disease is not universally appreciated and this class of genetic variation is widely ignored in the practice of clinical variant interpretation. Current American College of Medical Genetics guidelines for the assessment of clinically relevant genetic variants focus primarily on missense, nonsense, or canonical splice variants and suggest that synonymous “silent” variants should be classified as likely benign if the nucleotide position is not conserved and they are not implicated by splicing assessment tools [7].

The variant assessment community has numerous computational tools to systematically assess the pathogenicity of amino acid–altering nsSNVs. These algorithms are primarily based upon the high conservation of protein sequences and as such are not equipped to assess pathogenicity in synonymous variants, which are under different constraints [87]. Given the scarcity of RNA structure–specific tools that would aid in the simultaneous assessment of both nsSNVs and functional sSNVs in a given patient's genome, we are almost certainly missing novel disease etiologies that have their molecular underpinnings in pathological alterations to mRNA structure.

One of the primary goals of the present study was to address this critical need by creating metrics to enable systematic assessment of all sSNVs in a given individual's genome. While our structural metrics and SPI are not the first attempt to quantify pathogenicity due to mRNA-structural distortion, current methods are limited in their application for genome-wide variant assessment. For example, the RNAsnp Web Server predicts the change in optimal mRNA structure and base-pairing probabilities due to an SNV [77], and the command line tool remuRNA calculates the relative entropy between the mutant and wild-type mRNA-structural ensembles [88]. However, while these tools predict disruptions to mRNA structure, they do not attempt to predict pathogenicity and must be executed manually on each variant of interest.

Both RNAsnp and remuRNA were recently used to create a database of synonymous mutations in cancer (SynMICdb), using data from COSMIC across 88 tumor types [71]. For constitutional genetic disease, a related resource is the Database of Deleterious Synonymous Mutation (dbDSM), which manually curates sSNVs reported to be pathogenic in the literature and in databases like ClinVar [89]. These resources represent an important step towards evaluating sSNVs in disease. However, outside of those synonymous variants known to affect splicing, relatively few sSNVs have well-supported evidence of their pathogenicity. As such, to evaluate our metrics, we focused on a set of 9 sSNVs that we believe the authors unequivocally demonstrated to be pathogenic through their effects on mRNA structure (Table 2). This dataset included 1 variant in *OPTC* associated with glaucoma [62], 2 variants in *NKX2-5* associated with congenital heart defects [64], 1 variant in *DRD2* associated with post-traumatic stress disorder [60], 2 variants in *COMT* associated with pain sensitivity [61], 1 variant in *F2* (prothrombin) associated with thrombosis [90], and 2 variants linked to cancer in *KRAS* [71] and *TP53* [91].

All 9 sSNVs demonstrated definite enrichment for our structural metrics, by stability, edge distance, diversity, or SPI, with the summary metric, SURF, having values in the 90th percentile range for all 9 sSNVs. For example, the synonymous variant in *F2* (NM_000506.4: c.1824C>T; p.Arg608=) had a SURF score in the 97th percentile (driven by a high MFEED value), indicating that the variant introduced a high number of base-pair changes in the *F2* mRNA. Moreover, the negative ΔMFE and ΔCD values that we report for this variant indicate that it results in a more stable mRNA with reduced diversity in the structural ensemble. This fits with the observations of Pruner et al. [90], who demonstrated that the variant increased *F2* mRNA levels, carriers of the variant had increased concentrations of F2 in plasma, and the frequency of the variant was significantly higher in patients with venous thromboembolism and cerebrovascular insult.

Notably, none of these clinically relevant sSNVs qualifies as a truly exceptional outlier for any of our ViennaRNA metrics or SPI, with all percentiles being <99. It is plausible that such extreme outliers are not biologically tenable, making them less likely to appear in the human population. Another possibility is that these sSNVs occupy important regulatory positions and that an sSNV deleterious to mRNA secondary structure may exhibit pathogenicity when it distorts structure in a key region of the transcript. At any rate, the moderateness of our structural metrics in putative SNVs indicates that a 70th-percentile cut-off (Phred value ≥ 5) for pathogenicity would be reasonable.

### Molecular mechanisms underlying constraint of sSNVs

Synonymous variants that affect mRNA secondary structure could confer pathogenicity in numerous ways. Foremost of these mechanisms is that an unstable RNA has a shorter functional half-life and so produces less overall protein [20, 22, 24]. RNA structure modulates the movement of the ribosome along the mRNA molecule, dictating the length of pauses in ribosomal elongation and translocation, both critical for appropriate protein folding and ensuring that a safe distance is maintained between adjacent ribosomes [31]. Stronger structures may snap quickly back together after translation, reducing the possible time window for ribosomal collisions [27], while weaker secondary structures may disappear between ribosomes operating close to one another [92], demonstrating how precisely ribosomal positioning can be regulated through the folding of RNA. Ribosomal collisions essentially end the RNA's life, activating the no-go decay (NGD) pathway, and are also known to cause frame-shifts [26, 30, 92, 93]. In support of all these hypotheses, we note that the majority of our observed constraint is to preserve stability.

Another potential consequence of RNA misfolding is that a more stable mRNA may not be able to initiate translation, also resulting in lower protein levels [16, 18, 29, 39]. Nearly all species exhibit a reduction in mRNA stability near the start codon; however, for mammals and birds this trend in mainly seen in GC-rich genes [17]. Some studies suggest that by making the mRNA structure too difficult, or too easy, for the ribosome to process, synonymous codons can act to promote or frustrate proper protein folding [49]. RNA stability limits the growth rate of the peptide chain and thereby provides time for the core of the protein to establish itself [94, 95]. These findings emphasize the centrality of mRNA structure in regulation of ribosomal speed.

sSNVs also play roles in other processes that could affect our observations. While the stability of an mRNA transcript can determine how quickly it is translated [19, 29, 38], protein synthesis is regulated by both the abundance [96] and recruitment of transfer RNAs (tRNAs) through synonymous codon utilization (codon bias) [97–99]. However, there are 2 reasons we expect codon optimality to be a secondary factor in our study. First, we do not observe a depletion in mutations from optimal to suboptimal codons (see Supplementary Fig. S8). Second, the optimal reference codons tend to be those ending in G or C, so our REF>ALT contexts should largely account for changes in codon optimality. This assumption is consistent with an earlier study that clearly separated the 2 factors’ contributions to gene expression [27]. Yet it is worth remarking that optimal to suboptimal mutations (i.e., G/C to A/T) do show sharper constraint throughout our work. Regardless, to give proper weight to tRNA we include the tRNA Adaptivity Index (tAI), a measure of codon optimality, in our null model for SPI [100]. Our understanding of the role of bicodon bias in human disease is limited, yet pairing of consecutive codons is another mechanism by which the translational process is regulated [12, 47].

Finally, it is important to consider the essential role of synonymous codons in RNA splicing. While we took care to exclude sSNVs affecting the canonical splice sites from our constraint analysis, exonic variants beyond the canonical splice site can disrupt splice enhancers [101], or they may also activate cryptic splice sites, leading to loss of coding sequence (CDS) [102]. Given the diversity of molecular roles that synonymous codons have, it will be important for future studies to create scores that would allow assessment of sSNV pathogenicity through any these possible mechanisms.

## Potential Implications

We have shown that sSNVs that disrupt mRNA structure are significantly constrained in the human population, thereby supporting a growing understanding that previously assumed that “silent” polymorphisms actually play important roles in regulation of gene expression and protein function. We have demonstrated that this connection is rich, complex, and biologically intuitive. Given that there are multiple mechanisms by which sSNVs influence biological function, we are almost certainly missing undiscovered disease etiologies when these variants are ignored.

In addition to providing the community with a dataset of 10 ViennaRNA structural metrics for every known variant, our SPI represents a comprehensive method for predicting possible pathogenicity due specifically to changes in mRNA secondary structure. Because no single metric is capable of capturing all aspects by which a variant can alter structure, our summary metric SURF provides a single measurement to predict the impact of mRNA-structural variables in human genetic studies. We hope that these metrics will be used to accurately assess and prioritize an underrepresented class of genetic variation that may be playing a significant and as-yet-to-be-realized role in human health and disease.

## Methods

### RNA structure prediction process

Global assessment of sSNVs is truly a big data problem because it requires generation and evaluation of several raw values for each of hundreds of millions of positions within the genome. To address this challenge and successfully predict the mRNA-structural effects of every possible sSNV, we developed novel software built upon the Apache Spark framework (Fig.2). Apache Spark is a distributed, open source compute engine that drastically reduces the bottleneck of disk I/O by processing its data in memory whenever possible [103]. This leads to a 100× increase in speed and allows for more flexible software design than can be achieved in the traditional Hadoop MapReduce paradigm. Spark is well suited to address many of the challenges faced in analyzing big genomics data in a highly scalable manner, and adoption is growing steadily, with applications such as SparkSeq [104] for general processing, SparkBWA [105] for alignment, and VariantSpark for variant clustering [106]. By developing a solution within this framework, we eliminate significant computational hurdles standing in the way of large-scale analysis of sSNVs.

We used the NCBI RefSeq database (Release 81, GRCh38) as the source for all known human coding transcript sequences. At each position within a given transcript, 4 sequence windows of 101 bases were built, differing only in their central nucleotide, which was set to the reference nucleotide or 1 of the 3 possible alternate bases. If the nucleotide lay within 50 bases of the transcript boundary, the window was simply taken to be the leading/trailing 101 nucleotides of the transcript. Using Apache Spark in the Amazon Web Services (AWS) Elastic Map Reduce (EMR) service, we developed a massively parallel implementation of the ViennaRNA Package to analyze the 4 possible sequences. ViennaRNA is a secondary structure prediction package that has been extensively used and continuously developed for nearly 25 years, and uses the standard partition-function paradigm of RNA structural prediction [107].

Our Spark implementation of Vienna enabled us to examine changes in mRNA folding that result from any given polymorphism and thereby obtain 10 metrics that quantified the SNV's effect on mRNA secondary structure (see Supplementary Table S1). First, we used RNAfold to obtain predicted free energies for both mutant and wild-type sequences, which we compared directly to obtain 4 metrics describing the sSNV's effect on mRNA stability (ΔMFE, ΔCFE, ΔEFE, and ΔMEAFE). Next, we fed the predicted structures from RNAfold into the ViennaRNA programs RNApdist and RNAdistance to obtain 6 additional metrics quantifying the change in base-pairing (CED, MFEED, EED, MEAD) and ensemble diversity (ΔCD, ΔEND) due to each SNV. (See the documentation of [14] for detailed descriptions of these concepts.) We performed this procedure for all 470 million possible SNVs in 45,800 transcripts. After building our fasta files, we were able to run the whole computation in <24 hours using 51 c4.8xlarge AWS EMR computing nodes.

### Classification of variants

A common difficulty in variant classification is that an SNV may have different effects in different transcripts. To address this challenge, we annotated every SNV using the program snpEff [108], whose source code was modified to allow record-by-record calling via Spark. This snpEff analysis produced multiple annotations including the effect and location of the variant, e.g., missense, synonymous, canonical splice site, and so forth. To validate these snpEff predictions we also manually predicted the coding effect of each SNV using start and stop codon information from RefSeq [109]. The small number of sSNVs where our predicted biotype disagreed with snpEff's were discarded. After computing variant effect and location, we assigned each SNV a classification based on the most deleterious role it played in any transcript. In decreasing order of deleteriousness, these roles were start loss, stop gain, start gain, stop loss, missense, synonymous, 5′ UTR, 3′ UTR.

Having completed the annotation process we had a total of 470,606,772 SNVs in all known transcripts. Because exonic locations can share the same genomic coordinates for multiple transcripts, we next collapsed the data to 184,810,596 unique chromosome positions, assigning each variant a canonical transcript. Canonical transcripts were selected by (i) representation in the Matched Annotation from NCBI and EMBL-EBI (MANE) database (v0.9); or if the given gene was not in MANE, we chose either (ii) the transcript with the longest CDS or, when CDS length was the same across multiple transcripts for a given gene, (iii) the longest transcript. After filtering out variants implicated in splicing or lacking annotations needed in future steps, we obtained a dataset of 22.9 million synonymous variants, 70 million missense variants, 73 million variants in the 3′ UTR, and 13 million variants in the 5′ UTR. See Fig. 2 for a summary of our computational pipeline and Supplementary Table S5 for a record of the number of SNVs filtered at each stage.

### Determination of population minor allele frequencies

To measure constraint operating on an SNV we used population frequencies obtained from the gnomAD database. We combined both the exome variant calls from release v2.1.1 (originally mapped to GRCh37 and lifted over to GRCh38 coordinates by the gnomAD group) and genome sequencing variant calls from v3.1 (mapped and called using GRCh38). Quality filtering was applied using gnomAD recommendations, removing ∼1 million SNVs that failed random forest filtering (thresholds of 0.055 for gnomAD 2.1.1 exome data) and removing ∼3,000 SNVs with an inbreeding coefficient < 0.3. Approximately 22,000 were filtered out with a MAF ≥ 0.5 (indicative of sites where the reference allele represented a minor allele in the population). Finally, because the majority (∼90%) of SNVs have a gnomAD frequency 0, it was important to identify SNVs marked zero purely through a lack of coverage. To achieve this, we flagged and removed all sSNVs where <70% of samples had ≥20× coverage. Approximately 7.6 million SNVs failed these quality and coverage metrics, leaving a core dataset of 21.4 million synonymous variants, 68 million missense variants, 69 million variants in the 3′ UTR, and 12 million variants in the 5′ UTR (Supplementary Table S5). When combining the gnomAD data from whole-genome sequencing (WGS) and whole-exome sequencing (WES) sets, we used only those SNVs that passed all our filters in both sets. An SNV with MAF > 0 in only 1 of the sets was considered to have MAF > 0 in the joint set.

### Further variant annotations and data partitioning

We estimated the local nucleotide content around each sSNV by dividing each transcript into windows of 40 bases and in each window calculated the proportion of A's, C's, G's, T's, CpG's, and AT's in the surrounding 3 windows; these annotations were used in constructing SPI and identifying Mediator variables. Finally, we joined multiple additional annotations (including conservation metrics such as PhyloP) from the dbNSFP dataset [110]. Again, this heavy task was greatly facilitated by our Spark framework.

We carried out most of the analysis separately on subsets of data defined by a common mRNA reference and alternate allele, e.g., those sSNVs of form C>A. The reference and alternate alleles exert such a huge influence on gnomAD frequency that the best solution seemed to be to control for them explicitly. The number of sSNVs in each context and the proportion appearing in gnomAD are given in Supplementary Table S3.

### Identification of significant contexts

Table 1, which describes the correlation between our structural metrics and gnomAD frequency in each REF>ALT context, is an abbreviated version of the more complete description given in Supplementary Table S2. In each context we ran linear and quadratic regressions between our structural metric and the value *P*(MAF > 0) at each value of the metric, weighted by the number of sSNVs for which the metric attained that value. An asterisk denotes that quadratic *R*^2^ and *P*-values are reported instead of linear; this was done if quadratic pseudo-*R*^2^ exceeded the linear by a factor of ≥5. The normalized slope was computed by dividing the slope of the regression line by the average *P*(MAF > 0) in the context and then multiplying by the range covered by the metric in its central 90% of sSNVs. The “Constrained Against” field simply states whether the normalized slope (or the quadratic coefficient, in quadratic cases) is positive or negative.

### Mediator variables

Mediator variables (so called because they explain some of the connection between our mRNA-structural metrics and gnomAD frequency) are given in Table 1. They were chosen to be the sequence feature that explained the greatest portion of the connection between a structural metric (e.g., ΔMFE) and the proportion of nucleotides with MAF > 0 in a context. Possible Mediator variables that we considered were local nucleotide content and the specific nucleotides upstream/downstream of the sSNV.

To compute the proportion of correlation between a structural metric (e.g., ΔMFE) and MAF that is explained by a sequence feature such as CpG content in a particular REF-ALT context, we first built a simple logistic regression model to estimate the quantity $P({\mathrm{MAF}} > 0{\mathrm{\,\,}}|{\mathrm{\,\,CpG\,\,content}}$). We then plug the resulting estimate $\,\,{{P}_{{\bf{est}}}}({\mathrm{MAF}} > 0{\mathrm{\,\,}}|{\mathrm{\,\,CpG\,\,content}}$) into the expression

\begin{eqnarray*}
&&\,\,{{V}_{{\mathrm{CpG\,\,content}}}} = \mathop \sum \nolimits_x {{n}_{x}}\,\, \times \,\,{\left\{ {{E}\,\,[ {\,\,{{P}_{{\mathrm{est}}}}({\mathrm{MAF}} > 0{\mathrm{\,\,}}|{\mathrm{\,\,CpG\,\,content}}} )\,\,|\,\,}\right.}\\ &&\qquad \, {\left.{{\mathrm{\Delta MFE}}
= {x}]\,\,-{P}({\mathrm{MAF}} > 0{\mathrm{\,\,}}|\,\,{\mathrm{\Delta MFE}} = {x})} \right\}^2}\,\,\,\,, \end{eqnarray*}

where the sum is over all values of ΔMFE and $n_x$ is number of sSNVs in the context with ΔMFE = *x*. Comparing this quantity ${{V}_{{\mathrm{CpG\,\,content}}}}$ to the null variance

\begin{equation*} \,\,{{V}_{{\mathrm{null}}}} = \mathop \sum \nolimits_x {{n}_{x}}\,\, \times \,\,{\left[ {\,\,{P}({\mathrm{MAF}} > 0)-{P}({\mathrm{MAF}} > 0{\mathrm{\,\,}}|\,\,{\mathrm{\Delta MFE}} = {\mathrm{x}})} \right]^2}\,\,\,\, \end{equation*}

allows us to compute the proportion of the variation explained by CpG content:

\begin{equation*} \,\,{R}_{{\mathrm{CpG\,\,content}}}^2 = \,\,1 - \frac{{{{V}_{{\mathrm{CpG\,\,content}}}}}}{{{{V}_{{\mathrm{null}}}}}}. \end{equation*}

The “Mediator” for a given structural metric in a given context is chosen as the variable with the highest *R*^2^_._ Finally, the correlation between the Mediator and the event that MAF > 0 was checked, and the Mediator given a sign (+/-) so that it correlated positively with MAF > 0.

### Construction of SPI

To construct SPI scores we built 2 separate models over each of our 14 contexts to predict the event MAF > 0. The “null” model used multiple natural features—the 9 nucleotides in the SNV's home and adjacent codons, the proportion of A/C/G/T/CpG/AT's in the surrounding 120 nucleotides, the sSNV's position in its codon, its transcript and the transcript's length, and the tAI (obtained from a supplement of [111] from [112]) of the wild-type and mutant codons. The second, “active” model used all these features plus our 10 ViennaRNA metrics and the binding statuses of the reference and alternate bases in the predicted MFE structures generated by Vienna.

Both sets of variables were then used to predict whether MAF > 0 using a weighted general linear model as implemented in the LogisticRegression module of the Python scikit-learn package [113]. We then defined the SPI score for an sSNV to be the base-10 logarithm of the active model's predicted *P*(MAF > 0) divided by the null model's predicted *P*(MAF > 0). Context-wise plots for SPI are given in Supplementary Fig. S6.

We trained our SPIs using a 5-fold cross-validation in each SNV context, with the final assigned prediction being the average of all 5 predicted probabilities for a variant. When training SPI we used 6 separate schemes for partitioning the gnomAD data: WGS only, WES only, their union but throw away SNVs present in only 1 dataset, the union but count such SNVs as having MAF > 0, and analogously for intersections. Then in each SNV context we use the SPI score that yields the highest area under the curve. We also tried 3 different model styles for computing the raw predictions that comprise SPI—general logistic as implemented in Python's sklearn LogisticRegression module, random forest as implemented in sklearn's RandomForestClassifier, and gradient-boosted trees as implemented in the extreme gradient boosting Python package XGBoost [114]. The performance of each SPI “flavor” is given in Supplementary Table S4. We settled on the general logistic model, owing to its simplicity and also owing to the generally poor performance of the 2 tree-based models. SPI scores were *z*-score normalized (subtracted the mean and divided by the s.d.) and percentile ranked within each context. Finally, these context-specific percentile rankings were converted to a Phred-scaled score [$- 10{\mathrm{\,\,}} \times {\mathrm{\,\,}}\mathrm{lo}{\mathrm{g}_{10}}( {1 - {\mathrm{\,\,SPI\,\,Context\,\,Percentile}}} )$] prior to building Fig. 6.

### Construction of SURF

To construct our final SUmmarized RNA Folding (SURF) metrics (Fig. 7), each of the 10 RNA-folding metrics was percentile ranked and Phred-scaled, such that the larger the Phred-scaled value the greater the predicted change in RNA structure. For scores measuring a Δ in the given metric, negative stability and diversity values were ranked separately from positive values, using the formula $-10{\mathrm{\,\,}} \times {\mathrm{\,\,}}\mathrm{lo}{\mathrm{g}_{10}}( {1 - {\mathrm{Percentile\,\,Rank}}} )$. For edge distance, positive stability and positive diversity metrics results were Phred-scaled using the same formula. Finally, any Phred score >50 (i.e., a metric in the 99.999th percentile or above) was set to a value of 50, resulting in all Phred-scaled scores ranging from 0 to 50. For each SNV in our dataset, maximum Phred score was determined across the 4 stability metrics (ΔMFE, ΔCFE, ΔMEAFE, and ΔEFE) to generate the SURF Stability score, across the 4 edge distance metrics (CED, MFEED, EED, and MEAED) to generate SURF Edit Distance score, or across the 2 diversity metrics (ΔCD and ΔEND) to generate the SURF Diversity score. Finally, the single summary metric, SURF, was generated by choosing the maximum Phred score across any of the 10 RNA stability metrics and SPI.

## Availability of Source Code and Requirements

Project name: rna-stability

Project home page: https://github.com/nch-igm/rna-stability

Operating system: Linux

Programming language: Scala

Other requirements: Apache Spark 2.4+

License: FreeBSD

Biotools ID: bio.tools/rna-stability

RRID: https://scicrunch.org/scicrunch/Resources/record/nlx_144509-1/SCR_019259/resolver

## Data Availability

The software we developed and structural scores are available on GitHub [115] and via the *GigaScience* database GigaDB [116].

## Additional Files

**Supplementary Table S1**. Vienna RNA metrics

**Supplementary Table S2**. Constraint across sequence contexts

**Supplementary Table S3**. sSNV contexts across the human transcriptome

**Supplementary Table S4**. Modeling structural constraint with SPI score

**Supplementary Table S5**. Data pre-processing steps

**Supplementary Figure S1**. Distribution of structural metrics

**Supplementary Figure S2**. Calculation of edit distance

**Supplementary Figure S3**.Structural metrics over all synonymous SNVs

**Supplementary Figure S4**. Structural metrics in contexts constrained against destabilization

**Supplementary Figure S5**. Structural metrics in contexts constrained against overstabilization

**Supplementary Figure S6**. Sequence context and SPI

**Supplementary Figure S7**. Structural metrics vs log(MAF)

**Supplementary Figure S8**. Change in codon optimality vs mutation rate

## Abbreviations

AWS: Amazon Web Services; CDS: coding sequence; CED: centroid edit distance; COSMIC: Catalogue of Somatic Mutations in Cancer; ΔCD: Delta centroid distance; ΔMFE: Delta minimum free energy; gnomAD: Genome Aggregation Database; MAF: minor allele frequency; mRNA: messenger RNA; NCBI: National Center for Biotechnology Information; nsSNV: non-synonymous single-nucleotide variant; SNP: single-nucleotide polymorphism; SNV: single-nucleotide variant; SPI: Structural Predictivity Index; sSNV: synonymous single-nucleotide variant; SURF: SUmmarized RNA Folding; tAI: tRNA Adaptivity Index; tRNA: transfer RNA; UTR: untranslated region; WES: whole-exome sequencing; WGS: whole-genome sequencing.

## Competing Interests

The authors declare that they have no competing interests.

## Funding

Research reported in this publication was supported by the National Heart, Lung, and Blood Institute of the National Institutes of Health under award No. R01HL109758. We also thank the Nationwide Children's Foundation and The Abigail Wexner Research Institute at Nationwide Children's Hospital for generously supporting this body of work. J.L.L. was supported by the Pelotonia Fellowship for Undergraduate Research through The Ohio State University Comprehensive Cancer Society. These funding bodies had no role in the design of the study; no role in the collection, analysis, and interpretation of data; and no role in writing the manuscript.

## Authors’ Contributions

J.B.S.G., J.L.L., and P.W. developed methodology and performed data analysis and results interpretation. G.E.L. developed AWS Spark ViennaRNA pipeline and developed variant annotation tools. G.E.L. generated folding metrics. J.B.S.G. developed Structural Predictivity Index (SPI). D.M.G., H.C.K., B.J.K., and J.R.F assisted with data analysis, interpretation of results, and development of variant annotation tools. J.B.S.G, G.E.L., and P.W. prepared figures. All authors contributed to the preparation and editing of the final manuscript.

## Supplementary Material

giab023_GIGA-D-20-00178_Original_Submission

giab023_GIGA-D-20-00178_Revision_1

giab023_GIGA-D-20-00178_Revision_2

giab023_Response_to_Reviewer_Comments_Original_Submission

giab023_Response_to_Reviewer_Comments_Revision_1

giab023_Reviewer_1_Report_Original_Submission Yaoqi Zhou -- 11/5/2020 Reviewed

giab023_Reviewer_1_Report_Revision_1 Yaoqi Zhou -- 2/12/2021 Reviewed

giab023_Reviewer_2_Report_Original_Submission Anton A Komar, PhD -- 11/18/2020 Reviewed

giab023_Reviewer_2_Report_Revision_1 Anton A Komar, PhD -- 2/19/2021 Reviewed

giab023_Supplemental_File
