# Supplementary material for: Synonymous variants that disrupt messenger RNA structure are significantly constrained in the human population
Source: Gigascience. 2021 Apr 5;10(4):giab023. doi: 10.1093/gigascience/giab023 (PMC8023685; doi:10.1093/gigascience/giab023)
Supplement: giab023_GIGA-D-20-00178_Original_Submission [file giab023_giga-d-20-00178_original_submission.pdf]

GigaScience

Synonymous Variants that Disrupt mRNA Structure are Significantly Constrained in the Human Population

--Manuscript Draft--

**Manuscript Number:**	GIGA-D-20-00178
**Full Title:**	Synonymous Variants that Disrupt mRNA Structure are Significantly Constrained in the Human Population
**Article Type:**	Research
**Funding Information:**	
**Abstract:**	Background<br><br>The role of synonymous single nucleotide variants in human health and disease is poorly understood, yet there is a growing body of evidence to suggest that this class of “silent” genetic variation plays multiple regulatory roles in both transcription and translation. One mechanism by which synonymous codons direct and modulate the translational process is through alteration of the elaborate structure formed by single-stranded mRNA molecules. While tools to computationally predict the impact of non-synonymous variants on protein structure are plentiful, analogous tools to systematically assess how synonymous variants might disrupt mRNA structure are lacking.<br><br>Results<br><br>To address this need, we developed novel software using a parallel processing framework for large-scale generation of secondary RNA structures and folding statistics for the transcriptome of any species. Focusing our analysis on the human transcriptome, we calculated 5 billion RNA folding statistics for 469 million single nucleotide variants in 45,800 transcripts. By considering the impact of all possible synonymous variants globally, we discover that synonymous variants predicted to disrupt mRNA structure have significantly lower rates of incidence in the human population.<br><br>Conclusions<br><br>These findings support the hypothesis that synonymous variants may play a role in genetic disorders due to their effects on mRNA structure. Given that the community lacks tools to evaluate the potential pathogenic impact of synonymous variants, we provide RNA stability, edge distance and diversity metrics for every nucleotide in the human transcriptome and introduce a “Structural Predictivity Index” (SPI) to quantify structural constraint operating on any synonymous variant.
**Corresponding Author:**	Peter White, Ph.D.<br>Nationwide Children's Hospital<br>Columbus, OH UNITED STATES
**Corresponding Author Secondary Information:**	
**Corresponding Author's Institution:**	Nationwide Children's Hospital
**Corresponding Author's Secondary Institution:**	
**First Author:**	Jeffrey B.S. Gaither, Ph.D.
**First Author Secondary Information:**	
**Order of Authors:**	Jeffrey B.S. Gaither, Ph.D.
	Grant E. Lammi
	James L. Li

Powered by Editorial Manager® and ProduXion Manager® from Aries Systems Corporation

	David M. Gordon
	Harkness C. Kuck
	Benjamin J. Kelly
	James R. Fitch
	Peter White, Ph.D.
**Order of Authors Secondary Information:**	
**Additional Information:**	
**Question**	**Response**
Are you submitting this manuscript to a special series or article collection?	No
**Experimental design and statistics**<br><br>Full details of the experimental design and statistical methods used should be given in the Methods section, as detailed in our Minimum Standards Reporting Checklist. Information essential to interpreting the data presented should be made available in the figure legends.<br><br>Have you included all the information requested in your manuscript?	Yes
**Resources**<br><br>A description of all resources used, including antibodies, cell lines, animals and software tools, with enough information to allow them to be uniquely identified, should be included in the Methods section. Authors are strongly encouraged to cite Research Resource Identifiers (RRIDs) for antibodies, model organisms and tools, where possible.<br><br>Have you included the information requested as detailed in our Minimum Standards Reporting Checklist?	Yes
**Availability of data and materials**<br><br>All datasets and code on which the conclusions of the paper rely must be	Yes

either included in your submission or deposited in publicly available repositories (where available and ethically appropriate), referencing such data using a unique identifier in the references and in the “Availability of Data and Materials” section of your manuscript.<br><br>Have you have met the above requirement as detailed in our Minimum Standards Reporting Checklist?	

Powered by Editorial Manager® and ProduXion Manager® from Aries Systems Corporation

Main manuscript, tables and figure legends
Click here to download Manuscript RNA_stability_manuscript_GS.docx

Synonymous Variants that Disrupt mRNA Structure are Significantly Constrained in the Human Population

Jeffrey B.S. Gaither[1], Grant E. Lammi[1], James L. Li[1], David M. Gordon[1], Harkness C. Kuck[1], Benjamin J. Kelly[1], James R. Fitch[1] and Peter White[1,2,*]

[1] Computational Genomics Group, The Institute for Genomic Medicine, Nationwide Children's Hospital, Columbus, Ohio, USA

[2] Department of Pediatrics, College of Medicine, The Ohio State University, Columbus, Ohio, USA

* To whom correspondence should be addressed. Tel: +1 (614) 355-2671; Fax: +1 (614) 355-6833; Email: peter.white@nationwidechildrens.org

Mailing address:

Prof. Peter White, PhD
The Institute for Genomic Medicine
Nationwide Children's Hospital
575 Children's Crossroad
Columbus, OH 43215. USA

Running title: RNA Structural Constraint and Synonymous Variants

ABSTRACT

Background: The role of synonymous single nucleotide variants in human health and disease is poorly understood, yet there is a growing body of evidence to suggest that this class of "silent" genetic variation plays multiple regulatory roles in both transcription and translation. One mechanism by which synonymous codons direct and modulate the translational process is through alteration of the elaborate structure formed by single-stranded mRNA molecules. While tools to computationally predict the impact of non-synonymous variants on protein structure are plentiful, analogous tools to systematically assess how synonymous variants might disrupt mRNA structure are lacking.

Results: To address this need, we developed novel software using a parallel processing framework for large-scale generation of secondary RNA structures and folding statistics for the transcriptome of any species. Focusing our analysis on the human transcriptome, we calculated 5 billion RNA folding statistics for 469 million single nucleotide variants in 45,800 transcripts. By considering the impact of all possible synonymous variants globally, we discover that synonymous variants predicted to disrupt mRNA structure have significantly lower rates of incidence in the human population.

Conclusions: These findings support the hypothesis that synonymous variants may play a role in genetic disorders due to their effects on mRNA structure. Given that the community lacks tools to evaluate the potential pathogenic impact of synonymous variants, we provide RNA stability, edge distance and diversity metrics for every nucleotide in the human transcriptome and introduce a "Structural Predictivity Index" (SPI) to quantify structural constraint operating on any synonymous variant.

Keywords: synonymous variant, RNA structure, mRNA stability, genetic disease, Apache Spark, genomics

BACKGROUND

Accurate molecular genetic diagnosis of a rare disease is essential for patient care [1], yet today's best molecular tests and analysis strategies leave 60-75% of patients undiagnosed [2-6]. Current clinical practice for sequence variant interpretation focuses primarily on missense, nonsense or canonical splice variants [7], with numerous computational methods for prediction of the impact of non-synonymous single-nucleotide variants (**nsSNVs**) on protein function [8]. By contrast, we have limited knowledge in regard to the role that synonymous variants (**sSNVs**) may have in health and disease. These variants modify the codon in a transcript but leave the protein unchanged, and for years were erroneously considered to be "silent." However, the past two decades have seen a growing understanding that synonymous codons serve vital regulatory functions [9-12].

One of the principal levers by which synonymous codons direct the translational process is through mRNA structure. Unlike DNA, a messenger RNA (**mRNA**) molecule is single-stranded and therefore capable of forming complex configurations by base-pairing with itself, yielding the *secondary structure*, which further folds through covalent attractions to form the *tertiary structure* (**FIGURE 1**) [13]. The secondary structure has proven to be essential for understanding the regulatory functions of RNAs, and sophisticated methods exist to predict the ensemble of possible structures a given mRNA strand can adopt [14].

Studies first published in 1999 indicated that stable mRNA secondary structures are selected for in key genomic regions across all kingdoms of life [15-18]. Less stable RNA molecules may be more rapidly degraded resulting in lower protein levels [19-24]. The stability of an mRNA transcript also affects translational initiation and can determine the speed of translation [15-17, 25-27]. Recent studies have also strongly linked mRNA structure to protein conformation and function, with synonymous codons acting as a subliminal code for the protein folding process [12, 26, 28-30]. mRNA structure can also facilitate or prevent miRNAs and RNA-binding proteins from attaching to specific structural motifs [31-34]. Given all these mechanisms, when synonymous variants are ignored, we are almost certainly missing novel plausible explanations for genetic disease.

The role of mRNA structure in human health and disease, however, is poorly comprehended and relatively few pathogenic variants impacting mRNA folding have been described [19, 20, 22, 24]. A structure-altering sSNV in the dopamine receptor DRD2 inhibited protein synthesis and accelerated mRNA degradation [35]. A sSNV in

the *COMT* gene, implicated in cognitive impairment and pain sensitivity, was shown *in vitro* to constrain enzymatic activity and protein expression [36]. A sSNV in the *OPTC* gene of a glaucoma patient resulted in decreased protein expression *in vivo* [37]. In cystic fibrosis patients, a sSNV in *CFTR* was linked to decreased expression [38], and an mRNA-secondary-structure-altering silent codon change contributed to CFTR dysfunction by altering the dynamics of translation leading to protein misfolding [21, 23]. Two sSNVs in *NKX2-5*, identified in patients with congenital heart disease, decreased the mRNA's transactivation potential [39]. In hemophilia B, a sSNV in the factor IX gene impacted the transcript's secondary structure and reduced extracellular protein levels [40], and both synonymous and nonsynonymous variants were shown more likely be deleterious when occurring in a stable regions of *F8* and *DMD* mRNAs [41]. Our understanding of the role of synonymous variants in cancer is rapidly expanding, with recent studies demonstrating that they may act as drivers of the disease [42-44], altering the function of oncogenes such as RET [45] and KRAS [46].

We hypothesize that these reported instances of mRNA structure playing a role in disease represent only the tip of the iceberg. As such, the aims of this study were the creation of RNA-structural metrics for every possible single nucleotide variant (**SNV**) and to evaluate whether structure-disrupting SNVs are constrained in the human population. Through developing methods to predict whether a SNV is "structurally pathogenic," we hope to drive the discovery of novel genetic etiologies in both monogenic genetic disorders and more complex human disease.

DATA DESCRIPTION

Raw Dataset

To obtain all human mRNA transcripts we downloaded the NCBI RefSeq Release 81 from an online repository (ftp://ftp.ncbi.nlm.nih.gov/refseq/H_sapiens/mRNA_Prot/). Transcript sequences corresponded to human reference genome build GRCh38.

Massively parallel generation of RNA stability metrics

To assess the impact of synonymous mutations on mRNA structure, we carried out a genome-wide computation in which folding statistics were calculated for every possible variant in the human transcriptome

(RefSeq Release 81, GRCh38). For each position in all transcripts we built a 101-base window centered around the reference and three alternate sequences with the alternate allele substituted at the 51st position. We applied the ViennaRNA software package to the wildtype and mutated sequences to obtain 10 folding metrics quantifying the structural disruption caused by all three possible SNVs at the position (see **SUPPLEMENTARY DATA TABLE 1** for metric details). Computing this dataset of structural predictions for nearly half a billion SNVs was truly a "big data" computational task. We relied heavily on the parallelizability of the Apache Spark framework and custom wrappers which adapted the ViennaRNA software package to run within the Hadoop framework (**FIGURE 2**). Details of the calculation and subsequent assignment of variants into classes are given in **METHODS**.

Of the 10 mRNA-structural metrics output by our Vienna implementation, we adopted three as central to our analysis: dMFE, CFEED, and dCD. The metric dMFE (delta Minimum Free Energy) measures the change in mRNA free energy or "stability" caused by the sSNV, while CFEED (Centroid Free Energy Edit Distance) gives the number of base pairs that vary between the mutant and wildtype structures. The metric dCD (delta Centroid Distance) measures the sSNV's effect on the diversity of the mRNA's structural ensemble, which is the collection of various structures that a given sequence can exhibit. Distributions of these metrics, along with the other 7 mRNA-structural metrics output by our RNA structure pipeline are presented in **SUPPLEMENTARY DATA FIGURE 1**.

To test whether certain sSNVs are under constraint due to their effect on mRNA structure, we utilized population frequencies from the Genome Aggregation Database (gnomAD) containing aggregate genome and exome sequencing data from a total of 138,632 unrelated human individuals [47]. Our expectation was that SNVs with disruptive structural properties would be found less frequently in human populations. We defined a variant to be constrained if it was absent from gnomAD, and un-constrained if it had a gnomAD exon MAF > 0, a strategy similar to that employed by other groups [48, 49].

ANALYSIS

Global constraint to maintain stability

Our study reveals a striking connection between a given SNV's impact on mRNA structure and its frequency in the gnomAD database. This central finding is summarized in **FIGURE 3**, which depicts the proportion of SNVs with gnomAD MAF>0 at every value of our stability-metric dMFE. All four variant classifications – synonymous, 5′-UTR, 3′-UTR and missense – show a bi-directional constraint to maintain the wild-type mRNA structure. When the SNV either weakens the mRNA structure (high dMFE) or strengthens it (low dMFE) the SNV is depleted in the population roughly in proportion to the level of disruption. While this pattern of constraint was observed across all four variant classes, **FIGURE 3** indicates that it is strongest for synonymous variants.

FIGURE 4 summarizes constraint in the synonymous case, showing the relationship of our three main structural metrics with gnomAD frequency. **FIGURE 4A** recapitulates the pattern of green circles in **FIGURE 3**, revealing that disrupting mRNA stability decreases the chance of a synonymous SNV's appearing in human mRNA transcripts. The effect of removing or creating new base-pairings, quantified by the metric CFEED, is shown in **FIGURE 4B** (see **SUPPLEMENTARY DATA FIGURE 2** for an illustration of how CFEED is calculated). This figure validates our basic hypothesis that structurally disruptive sSNVs should appear less frequently in the population. We see that sSNVs which leave the centroid structure unchanged (i.e. CFEED=0) are roughly 15% more common than those sSNVs predicted to alter it, and SNVs with large CFEED values are constrained in proportion. Our third metric dCD measures change in the diversity of the mRNA ensemble (that is, the collection of all the structures formed by millions of *in vivo* mRNAs) and is shown in **FIGURE 4C**. This figure illustrates that changes in diversity – either towards more or less – are also constrained in gnomAD. The symmetry in depletion between over- and under-diversifying sSNVs is surprisingly regular.

The color-coding in **FIGURE 4** illuminates the relationship between the three structural metrics. Changes in stability are correlated with changes in base-pairing and vice-versa, as demonstrated by the red values at the extremes of each distribution. **FIGURE 4C** depicts a clear relationship between diversity and stability, with those sSNVs that diversify the ensemble (high dCD) also tending to weaken it (red). This diversity-instability relationship is intuitive, as a destabilizing mutation "frees up" portions of the mRNA to assume new shapes.

Variation of constraint with REF>ALT context

We next set out to determine if the constraint demonstrated in **FIGURE 4** holds uniformly for all synonymous nucleotides or whether it varies in different REF>ALT contexts. We would expect the latter as the bases C and G form much stronger structural bonds than do A and T. To probe this question we divide our sSNVs into 14 classes (**TABLE 1):** 12 classes based on their reference and alternate mRNA alleles (e.g. A>C, C>G, T>C, etc.) and 2 additional classes based on potential loss of methylated cytosine (CpG>TpG or CpG>CpA, the latter of which results from a deamination on an antisense strand). For consistency and clarity, we treat thymine as an mRNA base, even though it is actually replaced by uracil in mRNA. Then within each REF>ALT context we reconstruct the three plots of **FIGURE 4** and also perform weighted linear (or quadratic, for dCD) regressions between the three different stability metrics and the probability that the gnomAD minor allele frequency is > 0 (see **METHODS** for details and **SUPPLEMENTARY DATA TABLE 2** for full regression statistics).

We observe that constraint for mRNA structure is highly dependent on mutational context (**TABLE 1**). Some REF>ALT contexts show constraint in one direction only (e.g. against weakening of their structures), while other contexts show no significant constraint at all. The metric dMFE, which measures changes to mRNA energy or stability, shows a striking context-dependence (**TABLE 1A).** All significant REF>ALT changes are constrained *unidirectionally*, with one direction showing a depletion in population frequencies, while the other shows an enrichment (the direction of constraint is obtained by a weighted linear regression; see **METHODS** for details). In line with our understanding of structural biochemistry of RNA folding, mutations from “strong” REFs (C and G, so called because they form strong Watson-Crick bonds) to “weak” ALTs A and T are constrained against high values of dMFE. i.e. against the weakening of structure. Conversely, mutations from “weak” to “strong” nucleotides are constrained against the strengthening of structure (low dMFE). The exception to this rule is the context G>A (see *Constraint for mRNA stability in non-CpG-transitional contexts*).

Evaluation of the base-pair metric CFEED demonstrates that some contexts are constrained against large changes in mRNA base-pairing, while in others, SNVs altering base-pairs are actually enriched (**TABLE 1B).** This result reflects the fact that in some contexts *small* base-pairing changes are enriched over *no* base-pairing changes. In keeping with our main hypothesis, large changes of base-pairing are still uniformly constrained. As was the case with dMFE, we again observe that the context G>A is the exception.

Finally, **Table 1C** shows mutational contexts that exhibit significant constraint against changes to ensemble diversity as measured by dCD. We see that only a few contexts exhibit this constraint. In the two CpG-transitional contexts, the bell-shaped pattern of **Figure 4C** is faithfully reproduced, with both decreases and increases to ensemble diversity being equally harmful. However, the context G>A is enriched for changes in diversity – this context is strangely aberrant when assessed with all three metrics.

CpG transitions have constraint against de-stabilization of their mRNA structures

The data in **Table 1** show that our observed constraint for mRNA structure is greatest in the case of CpG transitions. Since these variants (and their suppression) are crucial to the story of mRNA stability, it is important to have an appreciation of their role in a biochemical context. The dinucleotide CG (usually denoted CpG to distinguish this linear sequence from the CG base-pairing of cytosine and guanine) is capable of becoming methylated and then mutating by a process called "deamination" into a TG dinucleotide; deaminations are also possible in unmethylated CpGs, but these result in a uracil that is quickly identified as a foreign base and repaired. In mammals 70-80% of CpGs are methylated, which makes a CpG transition almost 5x more common than any other mutation-type among mammals (see **Supplementary Data Table 3**) [50]. The nucleotides C and G also form foundational bonds in mRNA secondary structures. Most of the energy of an mRNA structure lies in its "stacks" of nucleotides with the average energy of a C-G pair in a stack around 65% stronger than that of any other base-pairing [51].

We find strong evidence that CpG transitions are constrained against weakening of their mRNA structures. This striking trend is largely explained (in a statistical sense) by CpG content, i.e. number of CpG dinucleotides in the vicinity (see "Proportion of variance explained by Mediator" in **Table 1**). **Figure 5** shows the populational constraint for our three main metrics in CpG-transitional contexts. Most strikingly, we find that synonymous CpG>CpA and CpG>TpG mutations both show a steady constraint against weakening of mRNA structure (high dMFE) (**Figures 5A & 5B**). Fascinatingly, both contexts exhibit a cluster of outliers in the most destructive (i.e. most de-stabilizing region), suggestive of extreme constraint borne of significant structural disruption.

The behavior of the edge metric CFEED in these contexts is also clear-cut. In **Figures 5C & 5D** we see a clear constraint against mutations with high CFEED values, and the red coloring shows that such changes are, on

average, de-stabilizing. We also observe a depletion at CFEED=0 in the CpG>TpG case; this is responsible for the bi-directional constraint reported in **Table 1**. Finally, **Figures 5E & 5F** show that the basic pattern of constraint for diversity in **Figure 4C** is reproduced and is essentially unchanged for both types of CpG transition. The coloring of **Figure 5** indicates that mutations CpG>CpA are more weakening on average than their CpG>TpG counterparts, despite being largely produced by the same biochemical mechanism (a CpG>TpG deamination on either the sense or anti-sense strand). We speculate on this disparity in the **Discussion.**

Constraint for mRNA stability in non-CpG-transitional contexts

We observe a constraint for mRNA structure in most REF>ALT contexts (as indicated by **Table 1**). We can classify the remaining contexts based on whether they are constrained against weakening or strengthening of their structures (as reported in **Table 1A**). **Supplementary Data Figure 3** shows plots of contexts where dMFE and gnomAD frequency are negatively correlated, i.e. where structure-weakening sSNVs are under constraint. Notably, all these contexts are strong>weak (or strong>strong in the case of C<>G), consistent with the principle that one purpose of such nucleotides is to maintain stability. In **Supplementary Data Figure 4** we show the contexts where dMFE and gnomAD frequency vary positively, which amounts to constraint against structure-strengthening sSNVs. Correspondingly, we note that two out of three of these contexts are weak>strong (and the third is the consistently aberrant context G>A).

Mediator variables

In **Table 1** we provide a “Mediator” variable for the connection between our RNA folding metrics and gnomAD frequencies in each mutational context. The name “Mediator” signifies that the variable explains some of the connection between the structural metric and gnomAD (details on how the Mediator and % variance explained are calculated are given in **Methods**.) These Mediators can explain large portions of the trends in **Figure 5** and **Supplementary Data Figures 3-4.** The striking trend between dMFE and gnomAD frequency in CpG-transitional contexts, for example, is largely driven by the local CpG content. CpG content is also the most powerful feature for CFEED and dCD in these contexts, with high CpG content consistently correlating with depletion. A

plausible inference is that an abundance of CpGs signifies important mRNA structure whose disruption could be harmful.

In non-CpG-transitional contexts, the Mediator almost always proves to be a nucleotide upstream or downstream of the sSNV. In the context C>A we can recover 28% of the relationship between dMFE and gnomAD frequency simply by looking at whether the C is followed by a G. The power of CpG dinucleotides in recovering our structural trends in the contexts C>A, C>G, G>C and G>T emphasizes the powerful but poorly understood role of CpGs in both mRNA stability and mammalian genomes.

Global quantification of mRNA constraint

Our analysis shows that variants predicted to disrupt mRNA secondary structures are constrained in the population. However, the complexity of mRNA structure means focusing on one single metric will surely lead to loss of information. To overcome this potential limitation of our RNA folding metrics, we set out to devise a more comprehensive method for predicting possible pathogenicity due to mRNA structure. Our strategy is to consider the *additional* statistical power bestowed by mRNA structure. In each context from **TABLE 1** we use RNA-sequence features (such as nearby bases and transcript position) to construct two separate models to estimate the probability that a sSNV will appear in gnomAD: an "active" model which incorporates our mRNA-structural metrics (P_s), and a null model which only uses sequence features (P_n). These models give us two separate estimates for the quantity P(MAF >0). Then we define the Structural Predictivity Index or **SPI** to be the log-quotient of the two probabilities:

$$\mathrm{SPI} = \log_{10}\left(\frac{P_s}{P_n}\right)$$

The metric SPI thus measures the predictive power bestowed by mRNA-structural variables. When it varies from 0, mRNA structural metrics yield new insight about a SNV's potential to have a functional role in mRNA secondary structure.

For both CpG-transitional contexts, CpG>CpA and CpG>TpG, those sSNVs with low SPI values are clearly under significant levels of constraint (**FIGURE 6**). The power of SPI in each of the 12 other sequence contexts (given by its area under the curve in predicting whether gnomAD nonzero frequency is >0) is supplied in

SUPPLEMENTARY DATA TABLE 4, and we provide plots of SPI vs. P(MAF>0) in every context in **SUPPLEMENTARY DATA FIGURE 5**. The behavior of SPI in non-CpG-transitional contexts proves to be less regular; every context shows a clear pattern, but this may amount to either enrichment or depletion (or both) as SPI moves in either direction. Given the strong dependence on REF-ALT context, the use of SPI as a deleteriousness score in non-CpG contexts may need further evaluation.

Clinical Examples of Structural Pathogenicity

The literature reveals only a few examples of synonymous sSNVs unequivocally shown to be pathogenic through their effects on mRNA structure. These sSNVs, with accompanying values of our three ViennaRNA metrics and SPI, are listed in **TABLE 2.** This set of known pathogenic sSNVs show a clear enrichment for our structural metrics, with each exhibiting a value of |dMFE|, CFEED, |dCD| or |SPI| that is in at least the 80th percentile in its context. For example, one pathogenic sSNV in NKX2-5, linked to congenital heart disease, has a dCD score in the 90th percentile [39]. It should be noted that none of these clinical sSNVs qualifies as a truly exceptional outlier for any of our ViennaRNA metrics or SPI; all have scores below the 95th percentile for |dMFE|, CFEED, |dCD| or |SPI| (see **DISCUSSION** for suggested score cutoff values).

DISCUSSION

We developed novel software to enable efficient generation of billions of RNA folding metrics for any species. This software allowed us to calculate RNA folding metrics for every base in the human transcriptome (approximately 0.5 billion SNVs). The RNA stability scores generated by this approach enable global assessment of synonymous variants and their potential role in human health and disease. We focused our analysis on the approximately 18 million synonymous variants found in the transcriptome, avoiding those sSNVs that could impact canonical splice sites and confound our analysis. Our study revealed that there is significant selection against sSNVs predicted to disrupt the given transcript’s local mRNA secondary structure, supporting our hypothesis that RNA structure itself plays a critical role in human health and disease.

Multiple arguments support a true causal relationship behind RNA stability and the observed correlation with constraint in the human population. First, we tested our hypothesis using three qualitatively distinct measures of structural disruption: change in stability (dMFE), change in base-pairing (CFEED) and change in ensemble diversity (dCD). All three metrics showed that SNVs which alter mRNA structure are constrained in human populations.

Second, our study revealed some patterns which can be elegantly explained in terms of mRNA structure. We showed that strong>weak mutations such as C>A are only depleted when they weaken mRNA structure, while weak>strong mutations are only depleted when they strengthen it. We also found that sSNVs with extreme dMFE and CFEED values are constrained even beyond the general trends (**FIGURE 5**), suggesting that this severe disruption is more-than-linearly unviable. Furthermore, **FIGURE 4B** highlights a pattern in CFEED values that alternates between high and low on successive values (CFEED can only take on even values because the destruction/creation of a base pair always requires two edits): the sSNVs with CFEED values that were multiples of 4 (4,8,12...) were shown to be enriched over those that were only multiples of 2 (2,6,10...). Such CFEED values are required if the total number of base-pairs is to be conserved, supporting that the constraint is needed to maintain overall base-pairing.

Third, the structural constraint we observe is not just restricted Watson-Crick base pairs, but also in nucleotides where wobble base pairing occurs. Wobble base-pairing takes place between two nucleotides such as guanine-uracil (G-U), that are not canonical Watson-Crick base pairs, but have comparable thermodynamic stabilities. We observed bi-directional constraint for dMFE in the context T>C, viewable in **SUPPLEMENTARY DATA FIGURE 4**. We conjecture the dual constraint in this context might be due to guanine's unique ability to wobble base-pair. Thus, the dual constraint from mutations T>C could be related to the transformation of T=G wobble base-pairs into stronger C=G Watson-Crick base pairs.

Finally, our Structural Predictivity Index (**SPI**) was created specifically to control for all confounding factors. The fact that SPI exhibits significant depletion in gnomAD further supports the hypothesis that mRNA structure is driving the constraint we observe, and that it is unlikely to be an artifact of any confounding factor. Furthermore, the Mediator variables given in **TABLE 1** are not "confounders" in the sense that they render our main

trends spurious, since they describe nucleic acid features around the SNV, such as the presence of a leading A or the local level of CpG dinucleotides, which are precisely the features that determine mRNA structure in the first place. Thus, it seems reasonable to regard these variables as mediators, rather than confounders, and award some of the causal priority to mRNA structure itself.

Successful identification of structurally disruptive sSNVs in known pathogenic synonymous variants

Over the last decade numerous studies have demonstrated that synonymous variants play essential molecular roles in regulating both mRNA structure and processing, including regulation of protein expression, folding and function [reviewed in 9, 52, 53]. However, the potential for pathogenic synonymous variants that impact RNA folding in human genetic disease remains largely unknown and this class of genetic variation is widely ignored in the practice of clinical variant interpretation. Current American College of Medical Genetics (**ACMG**) guidelines for the assessment of clinically relevant genetic variants focus primarily on missense, nonsense or canonical splice variants and suggest that synonymous "silent" variants should be classified as likely benign if the nucleotide position is not conserved and they are not implicated by splicing assessment tools [7].

The variant assessment community has numerous computational tools to systematically assess pathogenicity of nsSNVs. These algorithms are primarily based upon the high conservation of protein sequences, and as such are not equipped to assess pathogenicity in synonymous variants, which are under different constraints [54]. In the absence of functional tools that would aid in the simultaneous assessment of both nsSNVs and sSNVs in a given patient's genome, we are almost certainly missing novel disease etiologies that have their molecular underpinnings in pathological alterations to mRNA structure.

One of the primary goals of this present study was to address this critical need by creating metrics to enable systematic assessment of all sSNVs in a given subject's genome. While our structural metrics and SPI are not the first attempt to quantify pathogenicity due to mRNA-structural distortion, current methods are limited in their application for genome-wide variant assessment. For example, the RNAsnp Web Server predicts the change in optimal mRNA structure and base-pairing probabilities due to a SNV [55], and the command line tool remuRNA calculates the relative entropy between the mutant and wildtype mRNA structural ensembles [56]. However, while

these tools predict disruptions to mRNA structure, they do not attempt to predict pathogenicity and must be executed manually on each variant of interest.

Both RNAsnp and remuRNA were recently utilized to create a database of synonymous mutations in cancer (SynMICdb), using data from COSMIC across 88 tumor types [46]. For constitutional genetic disease, a related resource is the database of Deleterious Synonymous (dbDSM), which manually curates sSNVs reported to be pathogenic in the literature and in databases like ClinVar [57]. These resources represent an important step towards evaluating sSNVs in disease. However, outside of those synonymous variants known to impact splicing, relatively few sSNVs have well supported evidence of their pathogenicity. As such, to evaluate our metrics, we focused on a set of seven sSNVs that we believe the authors unequivocally demonstrated to be pathogenic through their effects on mRNA structure (**TABLE 2**). This dataset included one variant in OPTC associated with glaucoma [37], two variants in NKX2-5 associated with congenital heart defects [39], one variant in DRD2 associated with post-traumatic stress disorder [35], two variants in COMT associated with pain sensitivity [36], and one variant in F2 (prothrombin) associated with thrombosis [58].

All seven sSNVs demonstrated definite enrichment for our structural metrics, by stability, edge distance, diversity or SPI, with values in the 80th to 95th percentile range. For example, the synonymous variant in *F2* (NM_000506.5:c.1824C>T;p.Arg608=) had a CFEED value in the 94th percentile, indicating that the variant introduced a high number of base-pair changes in the *F2* mRNA. Moreover, the negative dMFE and dCD values we report for this variant indicate that it is a stabilizing change. This fits with the observations of Pruner *et al.*, as they demonstrated the variant increased *F2* mRNA levels, carriers of the variant had increased concentrations of F2 in plasma, and the frequency of the variant was significantly higher in patients with venous thromboembolism and cerebrovascular insult.

Notably, none of these clinically relevant sSNVs qualifies as a truly exceptional outlier for any of our ViennaRNA metrics or SPI with all percentiles being below 95. It is plausible that such extreme outliers are not biologically tenable, making them less likely to appear in the human population. Another possibility is that these sSNVs occupy important regulatory positions, and that a sSNV deleterious to mRNA secondary structure may

exhibit pathogenicity when it distorts structure *in a key region* of the transcript. At any rate, the moderateness of our structural metrics in putative SNVs indicate that an 80th-percentile cutoff for pathogenicity would be reasonable.

Molecular mechanisms underlying constraint of sSNVs

Synonymous variants that impact mRNA secondary structure could confer pathogenicity in numerous ways. Less stable RNA molecules may be degraded more quickly resulting in lower protein levels [19, 21, 23]. As local RNA structure is essential for the translation process, a more stable mRNA may not be able to initiate translation, also resulting in lower protein levels [16, 17, 26, 27]. Additionally, numerous studies argue that by making the mRNA structure too difficult, or too easy, for the ribosome to process, synonymous codons can act to promote or frustrate proper protein folding [30].

sSNVs also play roles in other molecular processes that could impact our observations. While the stability of an mRNA transcript can determine how quickly it is translated [15, 25, 26], protein synthesis is also regulated by both the abundance [59] and recruitment of tRNAs through synonymous codon utilization (codon bias) [60-62]. Changes in the bicodon bias have the potential to lead to pathogenicity of synonymous mutations in human disease [12, 29]. However, we mitigated some of this bias by including the tRNA Adaptivity Index (a measure of tRNA abundance) in our null model for SPI.

Finally, it is important to consider the essential role of synonymous codons in RNA splicing. While we took care to exclude sSNVs impacting the canonical splice sites from our constraint analysis, exonic variants beyond the canonical splice site can disrupt splice enhancers [63], or they may also activate cryptic splice sites, leading to loss of coding sequence [64]. Given the diversity of molecular roles that synonymous codons have, it will be important for future studies to create scores that would allow assessment of sSNV pathogenicity through any these possible mechanisms.

POTENTIAL IMPLICATIONS

We have shown that sSNVs which disrupt mRNA structure are significantly constrained in the human population, thereby supporting a growing understanding that previously assumed "silent" polymorphisms actually

play important roles in regulation of gene expression and protein function. We have demonstrated that this connection is rich, complex, and biologically intuitive. Given that there are multiple mechanisms by which sSNVs influence biological function, we are almost certainly missing undiscovered disease etiologies when these variants are ignored.

In addition to providing the community with a dataset of ten ViennaRNA structural metrics for every known variant, our Structural Predictivity Index is the first metric of its kind to enable global assessment of sSNVs in human genetic studies. We hope that these metrics will be utilized to accurately assess and prioritize an underrepresented class of genetic variation that may be playing significant and as yet to be realized role in human health and disease.

METHODS

RNA structure prediction process

Global assessment of sSNVs is truly a big data problem as it requires generation and evaluation of several raw values for each of hundreds of millions of positions within the genome. To address this challenge and successfully predict the mRNA-structural effects of every possible sSNV, we developed novel software built upon the Apache Spark framework (**FIGURE 2**). Apache Spark is a distributed, open source compute engine that drastically reduces the bottleneck of disk I/O by processing its data in memory whenever possible [65]. This leads to a 100x increase in speed and allows for more flexible software design than can be achieved in the traditional Hadoop MapReduce paradigm. Spark is well suited to address many of the challenges faced in analyzing big genomics data in a highly scalable manner and adoption is growing steadily, with applications such as SparkSeq [66] for general processing, SparkBWA [67] for alignment and VariantSpark for variant clustering [68]. By developing a solution within this framework, we eliminate significant computational hurdles standing in the way of large-scale analysis of sSNVs.

We used the NCBI RefSeq database (Release 81, GRCh38) as the source for all known human coding transcript sequences. At each position within a given transcript, four 101-base sequence windows were built, differing only in their central nucleotide, which was set to the reference nucleotide or one of the three possible

alternate bases. Using Apache Spark in the Amazon Web Services (**AWS**) Elastic Map Reduce (**EMR**) service, we developed a massively parallel implementation of the ViennaRNA Package to analyze the four possible sequences. ViennaRNA is a secondary structure prediction package that has been extensively utilized and continuously developed for nearly twenty-five years, and uses the standard partition-function paradigm of RNA structural prediction [69].

Our Spark implementation of Vienna enabled us to examine changes in mRNA folding that result from any given polymorphism, and thereby obtain ten metrics which quantified the SNV's effect on mRNA secondary structure (see **SUPPLEMENTARY DATA TABLE 1**). First, we utilized RNAfold to obtain predicted free energies for both mutant and wildtype sequences, which we compared directly to obtain four metrics describing the sSNV's effect on mRNA stability (dMFE, dCFE, dEFE and dMEAFE). Next, we fed the predicted structures from RNAfold into the ViennaRNA programs RNApdist and RNAdistance to obtain 6 additional metrics quantifying the change in base-pairing (CFEED, MFEED, EFEED, MEAD) and ensemble diversity (dCD, dEND) due to each SNV. (See the documentation of [14] for detailed descriptions of these concepts). We performed this procedure for all 469 million possible SNVs in 45,800 transcripts. After building our fasta files, we were able to run the whole computation in less than 24 hours using 51 c4.8xlarge AWS EMR computing nodes.

Classification of variants

A common difficulty in variant classification is that a SNV may have different effects in different transcripts. To address this challenge, we annotated every SNV using the program snpEff [70], whose source code was modified to allow record-by-record calling via Spark. This snpEff analysis produced annotations of predicted biotype, e.g. missense, synonymous, canonical splice site, etc. To validate these snpEff predictions we also manually predicted the biotype of each SNV using start and stop codon information from RefSeq (ftp://ftp.ncbi.nih.gov/refseq/H_sapiens/RefSeqGene/refseqgene.*.genomic.gbff.gz). The small number of sSNVs where our predicted biotype disagreed with snpEff's were discarded.

After computing variant types, we assigned each SNV a classification based on the most deleterious role it played in any transcript. In decreasing order of deleteriousness, these roles were: start loss, stop gain, start gain,

stop loss, missense, synonymous, 5 prime UTR, 3 prime UTR. Each SNV identified as “synonymous” by this scheme was assigned a “home transcript,” chosen based on proximity to the start codon, then on maximal transcript coding sequence length, and then arbitrarily.

This left us with a raw set of 22.9 million synonymous variants. After filtering out variants implicated in splicing or lacking annotations needed in future steps, we obtained a core dataset of 17.9 million sSNVs. (See **Figure 2** for a summary of our computational pipeline and **Supplementary Data Table 5** for a record of the number of SNVs filtered at each stage).

Merging of sSNV GRCh38 transcript coordinates with gnomAD GRCh37 coordinates

To measure constraint operating on a sSNV we used population frequencies obtained from the gnomAD database. Since this resource only existed for the GRCh37 reference build, we lifted our entire dataset from GRCh38 to GRCh37. The lifting procedure was carried out using the Picard Tools program liftOver, which was executed using a custom Spark wrapper. Since the great majority (approximately 90%) of sSNVs were marked with gnomAD frequency 0, it was important to identify sSNVs marked zero purely through a lack of coverage. To achieve this, we flagged and removed all sSNVs where fewer than 70% of samples had at least 20X coverage.

Further variant annotations and data partitioning

We estimated the local nucleotide content around each sSNV by dividing each transcript into windows of 40 bases and in each window computed the proportion of A’s, C’s, G’s, T’s, CpG’s and AT’s in the surrounding three windows; these annotations were used in constructing SPI. Finally, we joined multiple additional annotations (including conservation metrics such as PhyloP) from the dbNSFP dataset [71]. Again, this heavy task was greatly facilitated by our Spark framework.

We carried out most of the analysis separately on subsets of data defined by a common mRNA reference and alternate allele, e.g those sSNVs of form C>A. The reference and alternate alleles exert such a huge influence on gnomAD frequency that the best solution seemed to be to control for them explicitly. The number of sSNVs in each context and the proportion appearing in gnomAD are given in **Supplementary Table 3**.

Identification of significant contexts

Table 1, which describes the correlation between our structural metrics and gnomAD frequency in each REF>ALT context, is an abbreviated version of the more complete description given in **Supplementary Table 3**. In each context we ran linear and quadratic regressions between our structural metric and the value P(MAF>0) at each value of the metric (weighted by the number of sSNVs for which the metric attained that value). The normalized slope was computed by dividing the slope of the regression line by the average P(MAF>0) in the context and then multiplying by the range covered by the metric in its central 90% of sSNVs. The “Constrained Against” field simply states whether the normalized slope is positive or negative.

Mediator variables

Mediator variables (so called because they explain some of the connection between our mRNA structural metrics and gnomAD frequency) are given in **Table 1**. They were chosen to be the sequence feature that explained the greatest portion of the connection between a structural metric (e.g. dMFE) and the proportion of nucleotides with MAF>0 in a context. Possible Mediator variables we considered were local nucleotide content and the specific nucleotides up/downstream of the sSNV.

To compute the proportion of correlation between a structural metric (e.g. dMFE) and MAF that is explained by a sequence feature such as CpG content in a particular REF-ALT context, we first built a simple logistic regression model to estimate the quantity $\mathbf{P}(\mathrm{MAF} > 0 \mid \mathrm{CpG\ content})$. We then plug this estimate into the expression

$$\mathrm{V}_{\mathrm{CpG\ content}} = \sum_{x} \mathbf{n_x} * (\mathbf{E}(\ \mathbf{P}(\mathrm{MAF} > 0\ |\mathrm{CpG\ content}) \mid \mathrm{dMFE} = \mathrm{x}) - \mathbf{P}(\mathrm{MAF} > 0 \mid \mathrm{dMFE} = \mathrm{x}))^2$$

where the sum is over all values of dMFE and $\mathbf{n_x}$ is number of sSNVs in the context with dMFE= x. Comparing this quantity to the null variance

$$\mathrm{V}_{\mathrm{null}} = \sum_{x} \mathbf{n_x} * (\ \mathbf{P}(\mathrm{MAF} > 0) - \mathbf{P}(\mathrm{MAF} > 0 \mid \mathrm{dMFE} = \mathrm{x}))^2$$

allows us to compute the proportion of the variation explained by CpG content:

$$R^2_{\text{CpG content}} = 1 - \frac{V_{\text{CpG content}}}{V_{\text{null}}}$$

The “Mediator” for a given structural metric in a given context is chosen as the variable with the highest R^2. Finally the correlation between the Mediator and the event that MAF>0 was checked, and the Mediator given a sign (+/-) so that it correlated positively with MAF>0-.

Construction of SPI

To construct our final SPI scores we built two separate models over each of our 14 contexts to predict the event MAF > 0. The "null" model used all natural features - the nine nucleotides in the SNV's home and adjacent codons, the proportion of A/C/G/T/CpG/AT's in the surrounding 120 nucleotides, the sSNV's position in its transcript and the transcript's length, and the tAI (tRNA Adapation Index obtained from a supplement of [72] from https://ars.els-cdn.com/content/image/1-s2.0-S0092867410003193-mmc2.xls) of the wildtype and mutant codons. The second, “active” model used all these features plus our 10 ViennaRNA metrics. Both sets of variables were then used to predict MAF > 0 using a weighted general linear model as implemented in the LogisticRegression module of the python scikit-learn package [73]. We then defined the SPI score for a sSNV to be the base-10 logarithm of the active model's predicted P(MAF>0) divided by the null model's predicted P(MAF>0). Context wise plots for SPI are given in the **Supplementary Data Figure 5**.

We tried three different model-styles for computing the raw predictions that comprise SPI – general logistic as implemented in python’s sklearn LogisticRegression module, random forest as implemented in sklearn’s RandomForestClassifier, and gradient-boosted trees as implemented in the extreme gradient boosting python package XGBoost [74]. Performance of each SPI “flavor” is given in **Supplementary Data Table 4**. We eventually settled on the general logistic model, as it out-performs the gradient-boosted tree model and does not over-fit as the random forest model does.

Availability of Source Code and Requirements

Project name: rna-stability

Project home page: https://github.com/nch-igm/rna-stability

Operating system: Linux

Programming language: Scala

Other requirements: Apache Spark 2.4+

License: FreeBSD

Restrictions to use by non-academics: Our software pipeline and RNA-stability scores are free for non-commercial use by academic, government, and non-profit/not-for-profit institutions. A commercial version of the software and scores can be licensed through the Office of Technology Commercialization at Nationwide Children's Hospital. For more information, please contact: tech.commercialization@nationwidechildrens.org; Tel: (614) 355-2818.

Availability of Supporting Data and Materials

The software we developed and structural scores are available on GitHub: https://github.com/nch-igm/rna-stability and are available via the *GigaScience* database GigaDB [75].

Additional Files

A single supplementary data file (RNA_stability_supplementary_data.pdf) is available at *GigaScience* online and contains the following tables and figures:

Supplementary Data Table 1 - Vienna RNA Metrics

Supplementary Data Table 2 - Constraint Across Sequence Contexts

Supplementary Data Table 3 - sSNV Contexts Across the Human Transcriptome

Supplementary Data Table 4 - Modelling Structural Constraint with SPI Score

Supplementary Data Table 5 - Data Pre-Processing Steps

Supplementary Data Figure 1 - Distribution of Structural Metrics

Supplementary Data Figure 2 - Calculation of Edit Distance

Supplementary Data Figure 3 - Structural Metrics in Contexts Constrained Against Destabilization

Supplementary Data Figure 4 - Structural Metrics in Contexts Constrained Against Over-Stabilization

Supplementary Data Figure 5 – Sequence Context and SPI

DECLARATIONS

Abbreviations

CFEED: Centroid Free Energy Edit Distance; dCD: delta Centroid Distance; dMFE: delta Minimum Free Energy; gnomAD: Genome Aggregation Database; MAF: minor allele frequency; mRNA: messenger RNA; nsSNVs: non-synonymous single-nucleotide variants; SNP: single nucleotide variant; SNV: single nucleotide variant; SPI: Structural Predictivity Index; sSNVs: synonymous single-nucleotide variants

Competing interests

The authors declare no competing interests.

Funding

We thank the Nationwide Children's Foundation and The Abigail Wexner Research Institute at Nationwide Children's Hospital for generously supporting this body of work. James L. Li was supported by the Pelotonia Fellowship for Undergraduate Research through The Ohio State University Comprehensive Cancer Society. These funding bodies had no role in the design of the study, no role in the collection, analysis, and interpretation of data and no role in writing the manuscript.

Authors' contributions

J.B.S.G., J.L.L and P.W. developed methodology, performed data analysis and results interpretation. G.E.L. developed AWS Spark ViennaRNA pipeline and developed variant annotation tools. G.E.L. generated folding metrics. J.B.S.G. developed Structural Predictivity Index (SPI). D.M.G., H.C.K., B.J.K, and J.R.F assisted with data

analysis, interpretation of results and development of variant annotation tools. J.B.S.G, G.E.L and P.W. prepared figures. All authors contributed to the preparation and editing of the final manuscript.

Acknowledgements

This team works in the Steve and Cindy Rasmussen Institute for Genomic Medicine at Nationwide Children's Hospital. The Institute is generously supported by the Nationwide Foundation Pediatric Innovation Fund.

REFERENCES

1. Wright CF, FitzPatrick DR and Firth HV. Paediatric genomics: diagnosing rare disease in children. Nat Rev Genet. 2018;19 5:253-68. doi:10.1038/nrg.2017.116.
2. Yang Y, Muzny DM, Reid JG, Bainbridge MN, Willis A, Ward PA, et al. Clinical whole-exome sequencing for the diagnosis of mendelian disorders. N Engl J Med. 2013;369 16:1502-11. doi:10.1056/NEJMoa1306555.
3. Yang Y, Muzny DM, Xia F, Niu Z, Person R, Ding Y, et al. Molecular findings among patients referred for clinical whole-exome sequencing. JAMA. 2014;312 18:1870-9. doi:10.1001/jama.2014.14601.
4. Ellingford JM, Barton S, Bhaskar S, Williams SG, Sergouniotis PI, O'Sullivan J, et al. Whole Genome Sequencing Increases Molecular Diagnostic Yield Compared with Current Diagnostic Testing for Inherited Retinal Disease. Ophthalmology. 2016;123 5:1143-50. doi:10.1016/j.ophtha.2016.01.009.
5. Hegde M, Santani A, Mao R, Ferreira-Gonzalez A, Weck KE and Voelkerding KV. Development and Validation of Clinical Whole-Exome and Whole-Genome Sequencing for Detection of Germline Variants in Inherited Disease. Arch Pathol Lab Med. 2017;141 6:798-805. doi:10.5858/arpa.2016-0622-RA.
6. Worthey EA. Analysis and Annotation of Whole-Genome or Whole-Exome Sequencing Derived Variants for Clinical Diagnosis. Curr Protoc Hum Genet. 2017;95:9 24 1-9 8. doi:10.1002/cphg.49.
7. Richards S, Aziz N, Bale S, Bick D, Das S, Gastier-Foster J, et al. Standards and guidelines for the interpretation of sequence variants: a joint consensus recommendation of the American College of Medical

Genetics and Genomics and the Association for Molecular Pathology. Genet Med. 2015;17 5:405-24. doi:10.1038/gim.2015.30.

8. Alfares A, Aloraini T, Subaie LA, Alissa A, Qudsi AA, Alahmad A, et al. Whole-genome sequencing offers additional but limited clinical utility compared with reanalysis of whole-exome sequencing. Genet Med. 2018;20 11:1328-33. doi:10.1038/gim.2018.41.

9. Fahraeus R, Marin M and Olivares-Illana V. Whisper mutations: cryptic messages within the genetic code. Oncogene. 2016;35 29:3753-9. doi:10.1038/onc.2015.454.

10. Lee M, Roos P, Sharma N, Atalar M, Evans TA, Pellicore MJ, et al. Systematic Computational Identification of Variants That Activate Exonic and Intronic Cryptic Splice Sites. Am J Hum Genet. 2017;100 5:751-65. doi:10.1016/j.ajhg.2017.04.001.

11. Ramanouskaya TV and Grinev VV. The determinants of alternative RNA splicing in human cells. Mol Genet Genomics. 2017;292 6:1175-95. doi:10.1007/s00438-017-1350-0.

12. Hanson G and Coller J. Codon optimality, bias and usage in translation and mRNA decay. Nat Rev Mol Cell Biol. 2018;19 1:20-30. doi:10.1038/nrm.2017.91.

13. Silverman SK. A forced march across an RNA folding landscape. Chem Biol. 2008;15 3:211-3. doi:10.1016/j.chembiol.2008.02.014.

14. Lorenz R, Bernhart SH, Honer Zu Siederdissen C, Tafer H, Flamm C, Stadler PF, et al. ViennaRNA Package 2.0. Algorithms Mol Biol. 2011;6:26. doi:10.1186/1748-7188-6-26.

15. Seffens W and Digby D. mRNAs have greater negative folding free energies than shuffled or codon choice randomized sequences. Nucleic Acids Res. 1999;27 7:1578-84. doi:10.1093/nar/27.7.1578.

16. Katz L and Burge CB. Widespread selection for local RNA secondary structure in coding regions of bacterial genes. Genome Res. 2003;13 9:2042-51. doi:10.1101/gr.1257503.

17. Chamary JV and Hurst LD. Evidence for selection on synonymous mutations affecting stability of mRNA secondary structure in mammals. Genome Biol. 2005;6 9:R75. doi:10.1186/gb-2005-6-9-r75.

18. Gu W, Zhou T and Wilke CO. A universal trend of reduced mRNA stability near the translation-initiation site in prokaryotes and eukaryotes. PLoS Comput Biol. 2010;6 2:e1000664. doi:10.1371/journal.pcbi.1000664.

19. Duan J and Antezana MA. Mammalian mutation pressure, synonymous codon choice, and mRNA degradation. J Mol Evol. 2003;57 6:694-701. doi:10.1007/s00239-003-2519-1.

20. Wan Y, Qu K, Ouyang Z, Kertesz M, Li J, Tibshirani R, et al. Genome-wide measurement of RNA folding energies. Mol Cell. 2012;48 2:169-81. doi:10.1016/j.molcel.2012.08.008.

21. Lazrak A, Fu L, Bali V, Bartoszewski R, Rab A, Havasi V, et al. The silent codon change I507-ATC->ATT contributes to the severity of the DeltaF508 CFTR channel dysfunction. FASEB J. 2013;27 11:4630-45. doi:10.1096/fj.13-227330.

22. Hunt RC, Simhadri VL, Iandoli M, Sauna ZE and Kimchi-Sarfaty C. Exposing synonymous mutations. Trends Genet. 2014;30 7:308-21. doi:10.1016/j.tig.2014.04.006.

23. Shah K, Cheng Y, Hahn B, Bridges R, Bradbury NA and Mueller DM. Synonymous codon usage affects the expression of wild type and F508del CFTR. J Mol Biol. 2015;427 6 Pt B:1464-79. doi:10.1016/j.jmb.2015.02.003.

24. Bevilacqua PC, Ritchey LE, Su Z and Assmann SM. Genome-Wide Analysis of RNA Secondary Structure. Annu Rev Genet. 2016;50:235-66. doi:10.1146/annurev-genet-120215-035034.

25. Yang JR, Chen X and Zhang J. Codon-by-codon modulation of translational speed and accuracy via mRNA folding. PLoS Biol. 2014;12 7:e1001910. doi:10.1371/journal.pbio.1001910.

26. Presnyak V, Alhusaini N, Chen YH, Martin S, Morris N, Kline N, et al. Codon optimality is a major determinant of mRNA stability. Cell. 2015;160 6:1111-24. doi:10.1016/j.cell.2015.02.029.

27. Bazzini AA, Del Viso F, Moreno-Mateos MA, Johnstone TG, Vejnar CE, Qin Y, et al. Codon identity regulates mRNA stability and translation efficiency during the maternal-to-zygotic transition. EMBO J. 2016;35 19:2087-103. doi:10.15252/embj.201694699.

28. Plotkin JB and Kudla G. Synonymous but not the same: the causes and consequences of codon bias. Nat Rev Genet. 2011;12 1:32-42. doi:10.1038/nrg2899.

29. McCarthy C, Carrea A and Diambra L. Bicodon bias can determine the role of synonymous SNPs in human diseases. BMC Genomics. 2017;18 1:227. doi:10.1186/s12864-017-3609-6.

30. Walsh IM, Bowman MA, Soto Santarriaga IF, Rodriguez A and Clark PL. Synonymous codon substitutions perturb cotranslational protein folding in vivo and impair cell fitness. Proc Natl Acad Sci U S A. 2020;117 7:3528-34. doi:10.1073/pnas.1907126117.

31. Fernandez M, Kumagai Y, Standley DM, Sarai A, Mizuguchi K and Ahmad S. Prediction of dinucleotide-specific RNA-binding sites in proteins. BMC Bioinformatics. 2011;12 Suppl 13:S5. doi:10.1186/1471-2105-12-S13-S5.

32. Brummer A and Hausser J. MicroRNA binding sites in the coding region of mRNAs: extending the repertoire of post-transcriptional gene regulation. Bioessays. 2014;36 6:617-26. doi:10.1002/bies.201300104.

33. Savisaar R and Hurst LD. Both Maintenance and Avoidance of RNA-Binding Protein Interactions Constrain Coding Sequence Evolution. Mol Biol Evol. 2017;34 5:1110-26. doi:10.1093/molbev/msx061.

34. Dominguez D, Freese P, Alexis MS, Su A, Hochman M, Palden T, et al. Sequence, Structure, and Context Preferences of Human RNA Binding Proteins. Mol Cell. 2018;70 5:854-67 e9. doi:10.1016/j.molcel.2018.05.001.

35. Duan J, Wainwright MS, Comeron JM, Saitou N, Sanders AR, Gelernter J, et al. Synonymous mutations in the human dopamine receptor D2 (DRD2) affect mRNA stability and synthesis of the receptor. Hum Mol Genet. 2003;12 3:205-16. doi:10.1093/hmg/ddg055.

36. Nackley AG, Shabalina SA, Tchivileva IE, Satterfield K, Korchynskyi O, Makarov SS, et al. Human catechol-O-methyltransferase haplotypes modulate protein expression by altering mRNA secondary structure. Science. 2006;314 5807:1930-3. doi:10.1126/science.1131262.

37. Acharya M, Mookherjee S, Bhattacharjee A, Thakur SK, Bandyopadhyay AK, Sen A, et al. Evaluation of the OPTC gene in primary open angle glaucoma: functional significance of a silent change. BMC Mol Biol. 2007;8:21. doi:10.1186/1471-2199-8-21.

38. Bartoszewski RA, Jablonsky M, Bartoszewska S, Stevenson L, Dai Q, Kappes J, et al. A synonymous single nucleotide polymorphism in DeltaF508 CFTR alters the secondary structure of the mRNA and the expression of the mutant protein. J Biol Chem. 2010;285 37:28741-8. doi:10.1074/jbc.M110.154575.

39. Reamon-Buettner SM, Sattlegger E, Ciribilli Y, Inga A, Wessel A and Borlak J. Transcriptional defect of an inherited NKX2-5 haplotype comprising a SNP, a nonsynonymous and a synonymous mutation, associated with human congenital heart disease. PLoS One. 2013;8 12:e83295. doi:10.1371/journal.pone.0083295.

40. Simhadri VL, Hamasaki-Katagiri N, Lin BC, Hunt R, Jha S, Tseng SC, et al. Single synonymous mutation in factor IX alters protein properties and underlies haemophilia B. J Med Genet. 2017;54 5:338-45. doi:10.1136/jmedgenet-2016-104072.

41. Hamasaki-Katagiri N, Lin BC, Simon J, Hunt RC, Schiller T, Russek-Cohen E, et al. The importance of mRNA structure in determining the pathogenicity of synonymous and non-synonymous mutations in haemophilia. Haemophilia. 2017;23 1:e8-e17. doi:10.1111/hae.13107.

42. Gotea V, Gartner JJ, Qutob N, Elnitski L and Samuels Y. The functional relevance of somatic synonymous mutations in melanoma and other cancers. Pigment Cell Melanoma Res. 2015;28 6:673-84. doi:10.1111/pcmr.12413.

43. Supek F, Minana B, Valcarcel J, Gabaldon T and Lehner B. Synonymous mutations frequently act as driver mutations in human cancers. Cell. 2014;156 6:1324-35. doi:10.1016/j.cell.2014.01.051.

44. Zhang D and Xia J. Somatic synonymous mutations in regulatory elements contribute to the genetic aetiology of melanoma. BMC Med Genomics. 2020;13 Suppl 5:43. doi:10.1186/s12920-020-0685-2.

45. Pecce V, Sponziello M, Damante G, Rosignolo F, Durante C, Lamartina L, et al. A synonymous RET substitution enhances the oncogenic effect of an in-cis missense mutation by increasing constitutive splicing efficiency. PLoS Genet. 2018;14 10:e1007678. doi:10.1371/journal.pgen.1007678.

46. Sharma Y, Miladi M, Dukare S, Boulay K, Caudron-Herger M, Gross M, et al. A pan-cancer analysis of synonymous mutations. Nat Commun. 2019;10 1:2569. doi:10.1038/s41467-019-10489-2.

47. Lek M, Karczewski KJ, Minikel EV, Samocha KE, Banks E, Fennell T, et al. Analysis of protein-coding genetic variation in 60,706 humans. Nature. 2016;536 7616:285-91. doi:10.1038/nature19057.

48. Gronau I, Arbiza L, Mohammed J and Siepel A. Inference of natural selection from interspersed genomic elements based on polymorphism and divergence. Mol Biol Evol. 2013;30 5:1159-71. doi:10.1093/molbev/mst019.

49. Huang YF, Gulko B and Siepel A. Fast, scalable prediction of deleterious noncoding variants from functional and population genomic data. Nat Genet. 2017;49 4:618-24. doi:10.1038/ng.3810.

50. Li E and Zhang Y. DNA methylation in mammals. Cold Spring Harb Perspect Biol. 2014;6 5:a019133. doi:10.1101/cshperspect.a019133.

51. Turner DH and Mathews DH. NNDB: the nearest neighbor parameter database for predicting stability of nucleic acid secondary structure. Nucleic Acids Res. 2010;38 Database issue:D280-2. doi:10.1093/nar/gkp892.

52. Sauna ZE and Kimchi-Sarfaty C. Understanding the contribution of synonymous mutations to human disease. Nat Rev Genet. 2011;12 10:683-91. doi:10.1038/nrg3051.

53. Shabalina SA, Spiridonov NA and Kashina A. Sounds of silence: synonymous nucleotides as a key to biological regulation and complexity. Nucleic Acids Res. 2013;41 4:2073-94. doi:10.1093/nar/gks1205.

54. Gelfman S, Wang Q, McSweeney KM, Ren Z, La Carpia F, Halvorsen M, et al. Annotating pathogenic non-coding variants in genic regions. Nat Commun. 2017;8 1:236. doi:10.1038/s41467-017-00141-2.

55. Sabarinathan R, Tafer H, Seemann SE, Hofacker IL, Stadler PF and Gorodkin J. The RNAsnp web server: predicting SNP effects on local RNA secondary structure. Nucleic Acids Res. 2013;41 Web Server issue:W475-9. doi:10.1093/nar/gkt291.

56. Salari R, Kimchi-Sarfaty C, Gottesman MM and Przytycka TM. Sensitive measurement of single-nucleotide polymorphism-induced changes of RNA conformation: application to disease studies. Nucleic Acids Res. 2013;41 1:44-53. doi:10.1093/nar/gks1009.

57. Wen P, Xiao P and Xia J. dbDSM: a manually curated database for deleterious synonymous mutations. Bioinformatics. 2016;32 12:1914-6. doi:10.1093/bioinformatics/btw086.

58. Pruner I, Farm M, Tomic B, Gvozdenov M, Kovac M, Miljic P, et al. The Silence Speaks, but We Do Not Listen: Synonymous c.1824C>T Gene Variant in the Last Exon of the Prothrombin Gene as a New Prothrombotic Risk Factor. Clin Chem. 2020;66 2:379-89. doi:10.1093/clinchem/hvz015.

59. Dong H, Nilsson L and Kurland CG. Co-variation of tRNA abundance and codon usage in Escherichia coli at different growth rates. J Mol Biol. 1996;260 5:649-63. doi:10.1006/jmbi.1996.0428.

60. Sabi R and Tuller T. Modelling the efficiency of codon-tRNA interactions based on codon usage bias. DNA Res. 2014;21 5:511-26. doi:10.1093/dnares/dsu017.

61. Quax TE, Claassens NJ, Soll D and van der Oost J. Codon Bias as a Means to Fine-Tune Gene Expression. Mol Cell. 2015;59 2:149-61. doi:10.1016/j.molcel.2015.05.035.

62. Rocha EP. Codon usage bias from tRNA's point of view: redundancy, specialization, and efficient decoding for translation optimization. Genome Res. 2004;14 11:2279-86. doi:10.1101/gr.2896904.

63. Soukarieh O, Gaildrat P, Hamieh M, Drouet A, Baert-Desurmont S, Frebourg T, et al. Exonic Splicing Mutations Are More Prevalent than Currently Estimated and Can Be Predicted by Using In Silico Tools. PLoS Genet. 2016;12 1:e1005756. doi:10.1371/journal.pgen.1005756.

64. Molinski SV, Gonska T, Huan LJ, Baskin B, Janahi IA, Ray PN, et al. Genetic, cell biological, and clinical interrogation of the CFTR mutation c.3700 A>G (p.Ile1234Val) informs strategies for future medical intervention. Genet Med. 2014;16 8:625-32. doi:10.1038/gim.2014.4.

65. Zaharia M, Chowdhury M, Das T, Dave A, Ma J, McCauley M, et al. Resilient distributed datasets: a fault-tolerant abstraction for in-memory cluster computing. *Proceedings of the 9th USENIX conference on Networked Systems Design and Implementation*. San Jose, CA: USENIX Association, 2012, p. 2-.

66. Wiewiorka MS, Messina A, Pacholewska A, Maffioletti S, Gawrysiak P and Okoniewski MJ. SparkSeq: fast, scalable and cloud-ready tool for the interactive genomic data analysis with nucleotide precision. Bioinformatics. 2014;30 18:2652-3. doi:10.1093/bioinformatics/btu343.

67. Abuin JM, Pichel JC, Pena TF and Amigo J. SparkBWA: Speeding Up the Alignment of High-Throughput DNA Sequencing Data. PLoS One. 2016;11 5:e0155461. doi:10.1371/journal.pone.0155461.

68. O'Brien AR, Saunders NF, Guo Y, Buske FA, Scott RJ and Bauer DC. VariantSpark: population scale clustering of genotype information. BMC Genomics. 2015;16:1052. doi:10.1186/s12864-015-2269-7.

69. McCaskill JS. The equilibrium partition function and base pair binding probabilities for RNA secondary structure. Biopolymers. 1990;29 6-7:1105-19. doi:10.1002/bip.360290621.

70. Cingolani P, Platts A, Wang le L, Coon M, Nguyen T, Wang L, et al. A program for annotating and predicting the effects of single nucleotide polymorphisms, SnpEff: SNPs in the genome of Drosophila melanogaster strain w1118; iso-2; iso-3. Fly (Austin). 2012;6 2:80-92. doi:10.4161/fly.19695.

71. Liu X, Wu C, Li C and Boerwinkle E. dbNSFP v3.0: A One-Stop Database of Functional Predictions and Annotations for Human Nonsynonymous and Splice-Site SNVs. Hum Mutat. 2016;37 3:235-41. doi:10.1002/humu.22932.

72. Tuller T, Carmi A, Vestsigian K, Navon S, Dorfan Y, Zaborske J, et al. An evolutionarily conserved mechanism for controlling the efficiency of protein translation. Cell. 2010;141 2:344-54. doi:10.1016/j.cell.2010.03.031.

73. Pedregosa F, Varoquaux G, Gramfort A, Michel V, Thirion B, Grisel O, et al. Scikit-learn: Machine Learning in Python. Journal of Machine Learning Research. 2011;12:2825-30.

74. Chen TQ and Guestrin C. XGBoost: A Scalable Tree Boosting System. Kdd'16: Proceedings of the 22nd Acm Sigkdd International Conference on Knowledge Discovery and Data Mining. 2016:785-94. doi:10.1145/2939672.2939785.

75. Gaither JBS, Lammi GE, Li JL, Gordon DM, Kuck HC, Kelly BJ, et al. Supporting data for "Synonymous Variants that Disrupt mRNA Structure are Significantly Constrained in the Human Population". GigaScience Database. 2020.

TABLE 1. Structural metrics correlate with gnomAD frequency in most REF>ALT contexts

Context	Constrained against	R^2	p-value	Mediator	Prop. of variance explained by Mediator
**(A). dMFE – Structural Constraint**					
**CpG>CpA**	Weakening	0.690	1.49e-72	-CpG content	0.785
**CpG>TpG**	Weakening	0.439	5.02e-42	-CpG content	0.727
**G>T**	Weakening	0.165	1.13e-27	+leading C	0.317
**C>G**	Weakening	0.139	1.35e-27	+trailing G	0.141
**C>T**	Weakening	0.125	9.61e-21	- leading G	0.159
**C>A**	Weakening	0.099	2.9e-19	+trailing G	0.283
**G>A**	Strengthening	0.034	1.47e-06	+leading G	0.221
**A>G**	Strengthening	0.029	7.69e-06	+trailing T	0.354
**T>C**	Strengthening	0.018	2.17e-04	+leading A	0.351
**G>C**	Weakening	0.018	0.000169	+leading C	0.182
**(B). CFEED – Base-pairing constraint**					
**CpG>CpA**	Changes	0.655	2.28e-17	- CpG content	0.822
**CpG>TpG**	Both	0.37*	2.37e-8*	+CpG content	0.604
**G>A**	Retention	0.338	4.18e-08	-trailing A	0.557
**T>C**	Changes	0.275	8.2e-07	+leading A	0.642
**C>T**	Changes	0.244	6.13e-06	+C content	0.382
**C>A**	Retention	0.217	1.96e-05	+trailing A	0.335
**(C). dCD – Diversity Constraint**					
**CpG>CpA**	Changes	0.637	1.42e-14	-CpG content	0.825
**G>A**	Maintenance	0.401	2.08e-8	- trailing A	0.645
**CpG>TpG**	Changes	0.236	3.89e-5	- CpG content	0.438

Correlation between structural metrics (**A**) dMFE, (**B**) CFEED and integer-rounded (**C**) dCD on the one hand, and the quantity P(MAF>0) on the other, over all sSNVs in a given context. The R^2 and p-values are obtained from a weighted least-squares linear regression, with the p-value corresponding to the linear coefficient; a quadratic regression was also performed, but only the p-value was retained as denoted by "*". Only context-metric pairs with p-value < 0.005 are included. "Normalized slope" was obtained by dividing slope of regression line by average P(MAF>0) in the context and then multiplying by range covered by metric in its central 90% of sSNVs. "Mediator" is raw sequence variable that explains largest proportion of structural trend *in this context*, with sign adjusted to correlate negatively with gnomAD frequency. "Mediator R^2" gives proportion of variance explained by the Mediator (see *Mediator variables* in **RESULTS** for details).

TABLE 2. Known sSNVs clinically implicated for structural pathogenicity are successfully predicted to be pathogenic by our structural metrics

Gene	Condition	SNP (GRCh37)	Context	|dMFE|	CFEED	|dCD|	|SPI|
**COMT**	Pain sensitivity	rs4633<br>NC_000022.10:g.19950235C>T<br>NM_000754.3:c.186C>T<br>NP_000745.1:p.His62=	CpG>TpG	0.5<br>(35.9)	**66<br>(88.9)**	3.80<br>(58.6)	0.0043<br>(31.5)
**COMT**	Pain sensitivity	rs4818<br>NC_000022.10:g.19951207C>G<br>NM_000754.3:c.408C>G<br>NP_000745.1:p.Leu136=	C>G	**3.0<br>(82.4)**	38<br>(60.3)	6.83<br>(72.2)	0.0027<br>(10.4)
**DRD2**	Schizophrenia, substance abuse	rs6277<br>NC_000011.9:g.113283459G>A<br>NM_000795.4:c.957C>T<br>NP_000786.1:p.Pro319=	CpG>TpG	1.0<br>(52.4)	**60<br>(86.4)**	**9.42<br>(87.4)**	0.0010<br>(65.7)
**F2**	Thrombosis	rs3136532<br>NC_000011.9:g.46760913C>T<br>NM_000506.5:c.1824C>T<br>NP_000497.1:p.Arg608=	C>T	1.7<br>(72.4)	**72<br>(94.2)**	4.50<br>(69.0)	0.0158<br>(63.2)
**NKX2-5**	Congenital heart disease	rs72554028<br>NC_000005.9:g.172660004C>T<br>NM_004387.4:c.543G>A<br>NP_004378.1:p.Gln181=	G>A	**3.5<br>(89.1)**	4<br>(30.0)	0.24<br>(10.6)	0.0098<br>(52.9)
**NKX2-5**	Congenital heart disease	rs2277923<br>NC_000005.9:g.172662024T>C<br>NM_004387.3:c.63A>G<br>NP_004378.1:p.Glu21=	A>G	0.0<br>(22.2)	20<br>(57.8)	**8.85<br>(89.9)**	0.0153<br>(47.3)
**OPTC**	Primary open angle glaucoma	rs559635109<br>NC_000001.10:g.203467924C>T<br>NM_014359.3:c.486C>T<br>NP_055174.1:p.Phe162=	C>T	0.0<br>(26.6)	32<br>(69.8)	2.53<br>(52.5)	**0.0261<br>(87.2)**

dbSNP RS number and standardized SNP annotations are provided, along with the genes official symbol and disease the sSNV has been associated with. The absolute value of dMFE, CFEED, dCD and SPI are provided, along with the percentile value of that score, computed over each context, in parentheses.

Figure Legends

Figure 1. A synonymous variant introduces a marked change in local minimum free energy of the mRNA secondary structures in the *DRD2* gene. Using a known synonymous variant of pharmacogenomic significance in the dopamine receptor, DRD2 (NM_000795.4:c.957C>T (p.Pro319=)), this figure demonstrates how the 101-bp window used in our analysis captures the variant's impact on RNA secondary structure. Wildtype (**A**) and mutant (**B** and **C**) sequences (RefSeq transcript NM_000795.4, coding positions 907-1008) are identical except for a synonymous C->T mutation at position 51 (major "C" allele is indicated by the black arrow, minor "T" allele is indicated by the red arrow). **(A)** Wildtype optimal and centroid structures (which coincide) demonstrate a relatively stable secondary structure with a minimum free energy of -12.5 kcal/mol. In the ensemble of possible structures arising from the sSNV a position 51, there is a significant reduction in stability of the molecule in terms of both the (**B)** mutant optimal structure (-11.5 kcal/mol) and (**C)** mutant centroid structure (-5.1 kcal/mol). The synonymous variant results in a less stable mRNA molecule which laboratory studies demonstrate reduces the half-life of the transcript, ultimately reducing protein expression of the dopamine receptor, DRD2. Nucleotides are colored according to the type of structure that they are in: Green: Stems (canonical helices); Red: Multiloops (junctions); Yellow: Internal Loops; Blue: Hairpin loops; Orange: 5' and 3' unpaired region.

Figure 2. Graphical depiction of computational workflow used to generate ViennaRNA folding metrics for the entire transcriptome. The entire analysis workflow was parallelized using Apache Spark and the Amazon Elastic Map Reduce (EMR) service, generating 5 billion ViennaRNA metrics over the course of 2 days. Using a custom pipeline developed for the process that was executed across 47 Amazon Elastic Cloud Compute (EC2) spot instances, input data was retrieved from an Amazon Simple Storage Solution (S3) bucket and processed through the pipeline consisting of 8 steps. We first obtained the 101-base sequence centered around a SNV in a transcript and generated three alternate sequences (with the ALT rather than the REF at position 51) (step 1). We next applied ViennaRNA modules to sequence to obtain structural metrics (step 2). Results were then mapped to chromosomal coordinates (step 3) and annotated with SnpEff to identify splice variants (step 4), lifted to the hg19

build (step 5), annotated with gnomAD population frequencies (step 6) and coverage information (step 7), and finally annotated with metrics from dbNSFP (step 8). Final dataset was written to Amazon S3 in Parquet columnar file format for further analysis and interpretation.

FIGURE 3. Exonic SNVs predicted to impact mRNA structure are constrained in the human population. Population frequency of SNVs was plotted against predicted impact on mRNA structure. Circles show proportion of SNVs with nonzero gnomAD exonic frequency at each value of the RNA stability metric dMFE. The bell-shaped pattern of constraint was observed across all classes of SNVs, with constraint appearing to be greatest in sSNVs (green), followed by SNVs in the 5 prime UTR (orange), then SNVs in the 3 prime UTR (blue), and finally nsSNVs (red). Values of dMFE with fewer than 1000 (synonymous), 200 (UTRs) or 2000 (missense) positive-MAF sSNVs are excluded. Axis limits are restricted to show main pattern more clearly, resulting in removal of a few high-P(MAF>0) synonymous outliers. Only SNVs with high exonic coverage are represented (see **METHODS** for details.)

FIGURE 4. Synonymous variants predicted to impact mRNA structure are constrained in the human population. Population frequency of sSNVs were plotted against the predicted impact on mRNA structure. Synonymous variants that disrupt structure tend to be absent from the gnomAD database, while those with limited impact on structure appear at least once in the gnomAD database. **(A)** Proportion of sSNVs with nonzero gnomAD frequency at each value of the RNA stability metric dMFE. Points with fewer than 2000 positive-MAF sSNVs excluded. Color represents average CFEED value, to highlight the relationship between minimum free energy and edit distance. **(B)** Analogous plot for metric CFEED measuring edge differences between mutant/wildtype centroid structures. Color represents |dMFE|, measuring absolute change in stability. **(C)** Analogous plot for diversity-metric dCD measuring change in structural ensemble diversity due to sSNV. Color is by dMFE measuring change in stability.

FIGURE 5. Synonymous CpG transitions are markedly constrained against destabilization of their mRNA structures. Population frequency of sSNV vs. effect on mRNA structure in synonymous CpG transitions was examined. Proportion of synonymous CpG transitions with nonzero MAF at each value of dMFE were determined for (**A**) CpG>CpA and (**B**) CpG>TpG synonymous mutations. dMFE values with fewer than 75 nonzero-MAF sSNVs are excluded. Color gives average CFEED in each context, ranging from 15 (blue) to 50 (red). Similarly, proportion of synonymous CpG transitions with nonzero MAF at each value of CFEED were determined for (**C**) CpG>CpA sSNVs and (**D**) CpG>TpG sSNVs. Color represents average dMFE and ranges from -0.8 (blue) to 1.85 (red). CFEED values with fewer than 75 nonzero-MAF sSNVs are excluded). Finally, proportion of synonymous CpG transitions with nonzero MAF at each value of dCD (after rounding to nearest integer) were determined for (**E**) CpG>CpA and (**F**) CpG>TpG sSNVs sSNVS. Color represents average dMFE and ranges from -3 (blue) to 4 (red). Rounded dCD values with fewer than 75 nonzero-MAF sSNVs are excluded.

FIGURE 6. SPI score correlates with constraint in synonymous CpG transitions. Variants in the contexts **(A)** CpG>CpA and **(B)** CpG>TpG are divided by SPI score into 20 equal bins and the value P(MAF>0) plotted against the mean of each bin. We also colored by the mean dMFE over each bin. In both contexts the constraint is highest towards negative SPI, i.e. sSNVs for which structural information decreases the predicted probability that MAF > 0.

Figure 1 Click here to download Figure Figure_1.eps

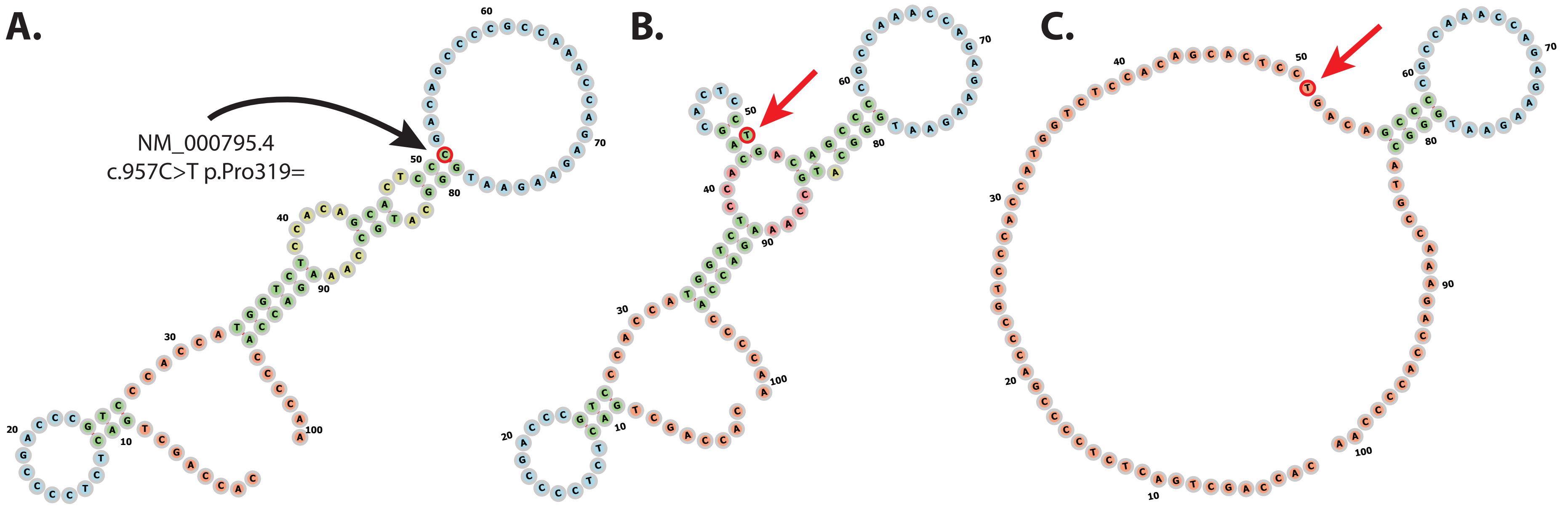

Figure 2

Click here to download Figure Figure_2.eps

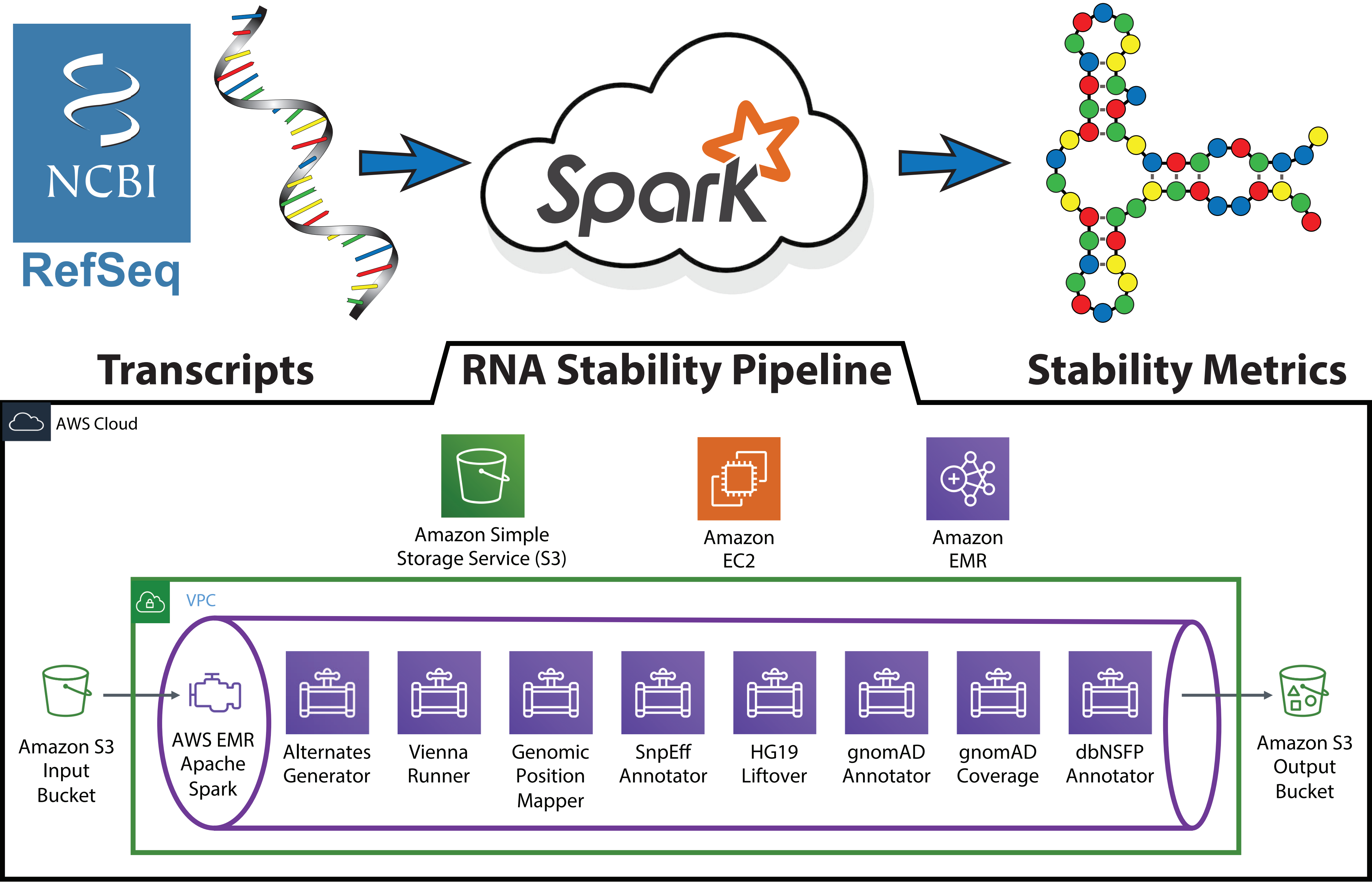

Figure 3

Click here to download Figure Figure_3.eps

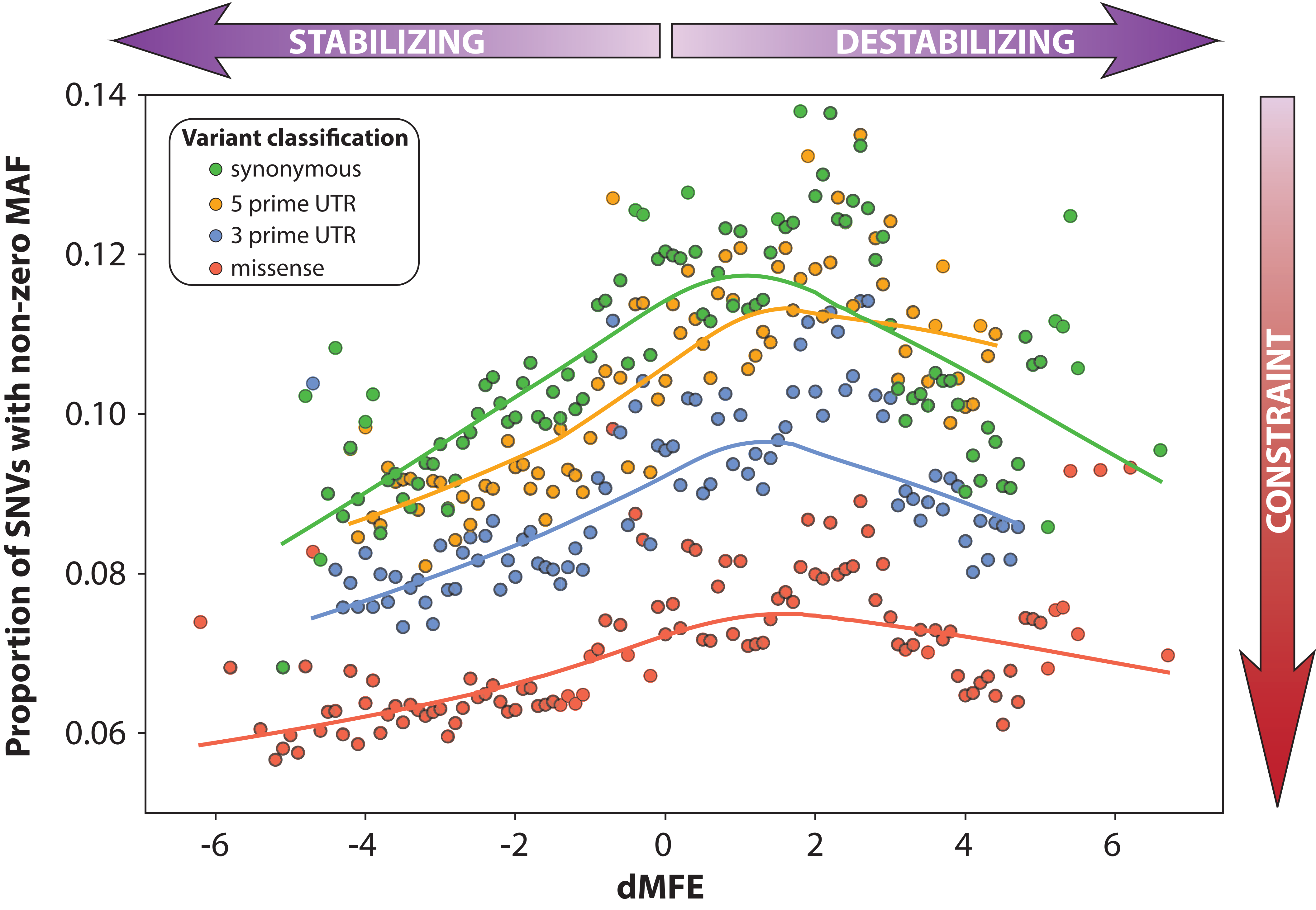

Figure 4

Click here to download Figure Figure_4.eps

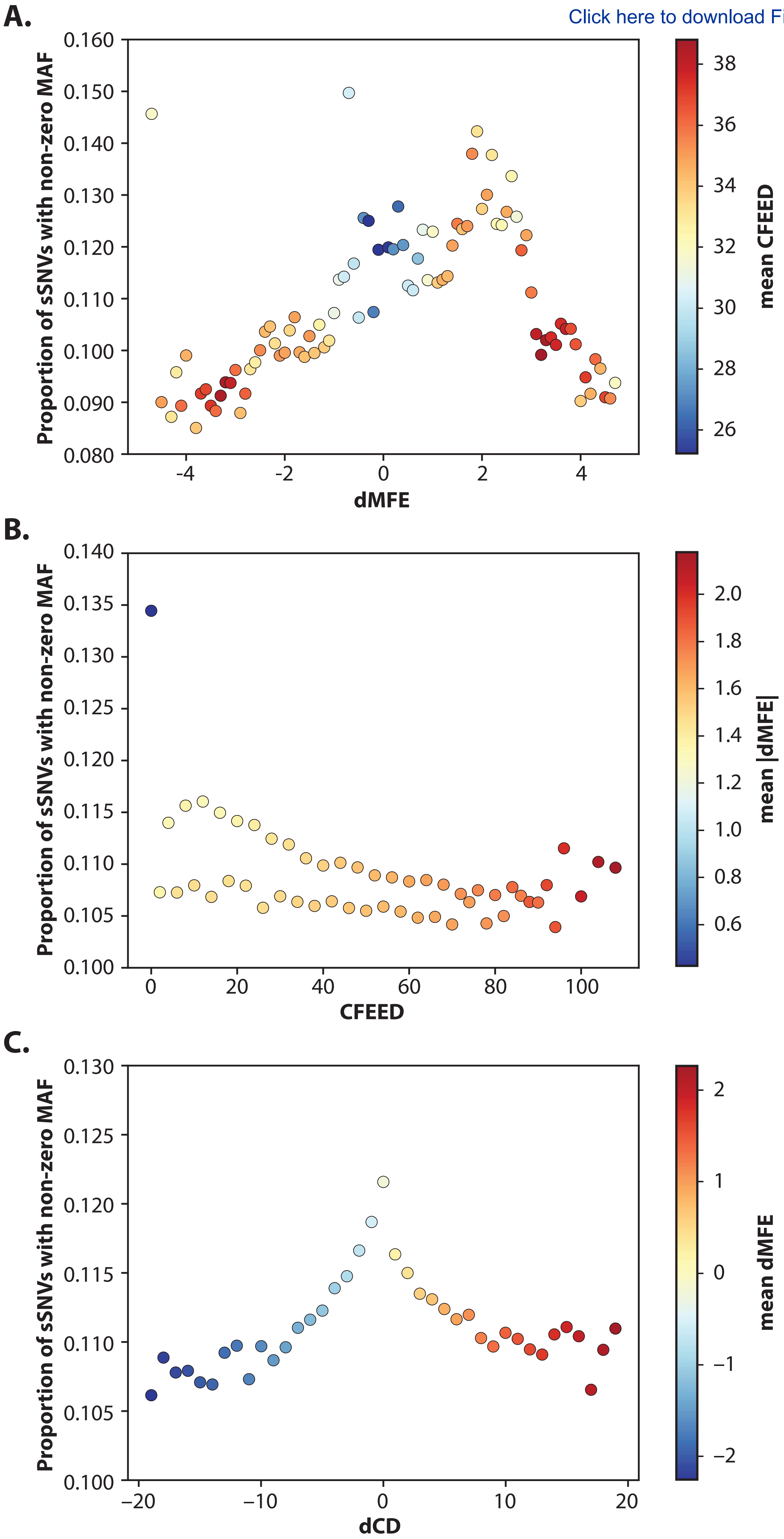

Figure 5

Click here to download Figure Figure_5.eps

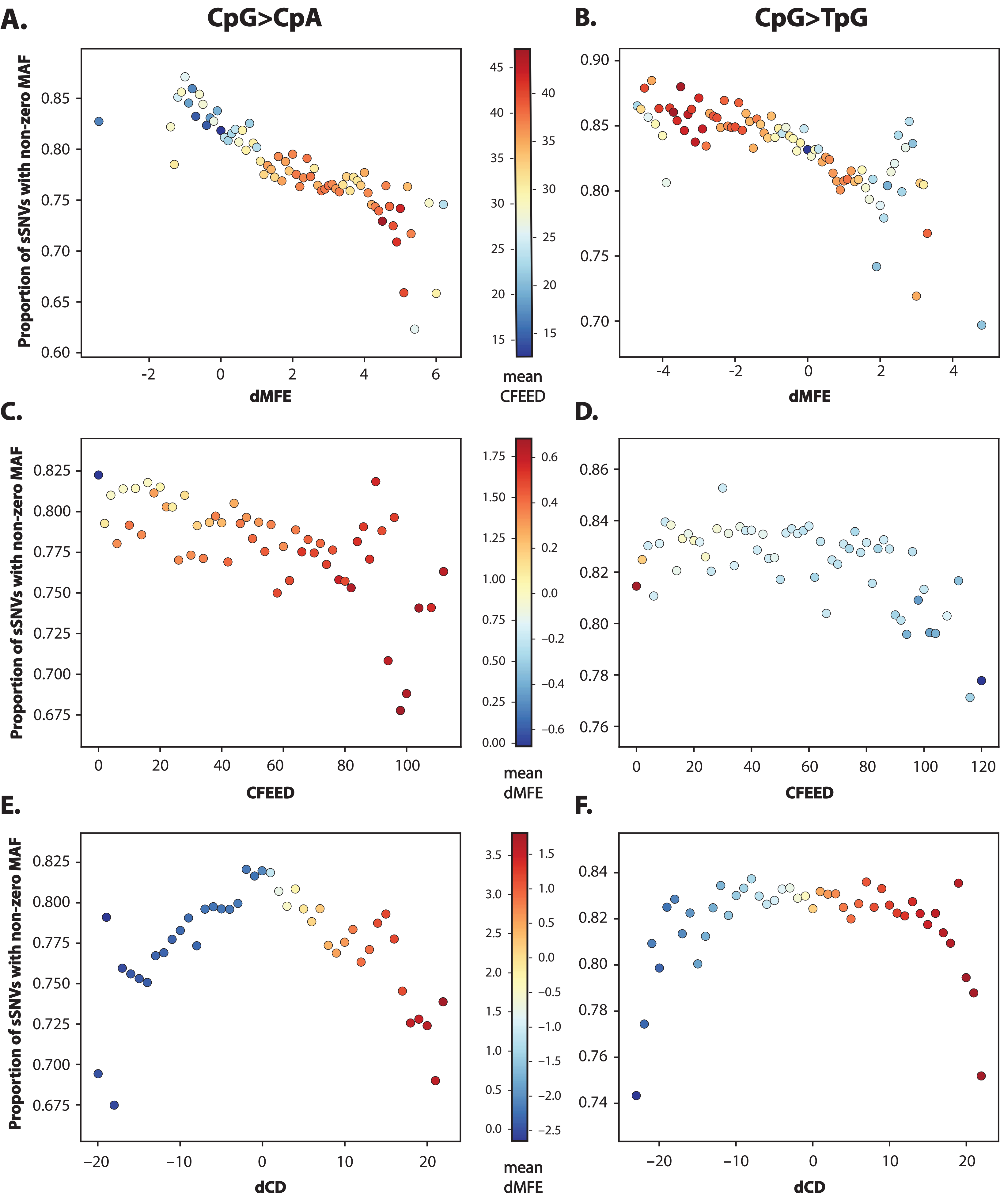

Figure 6

Click here to download Figure Figure_6.eps

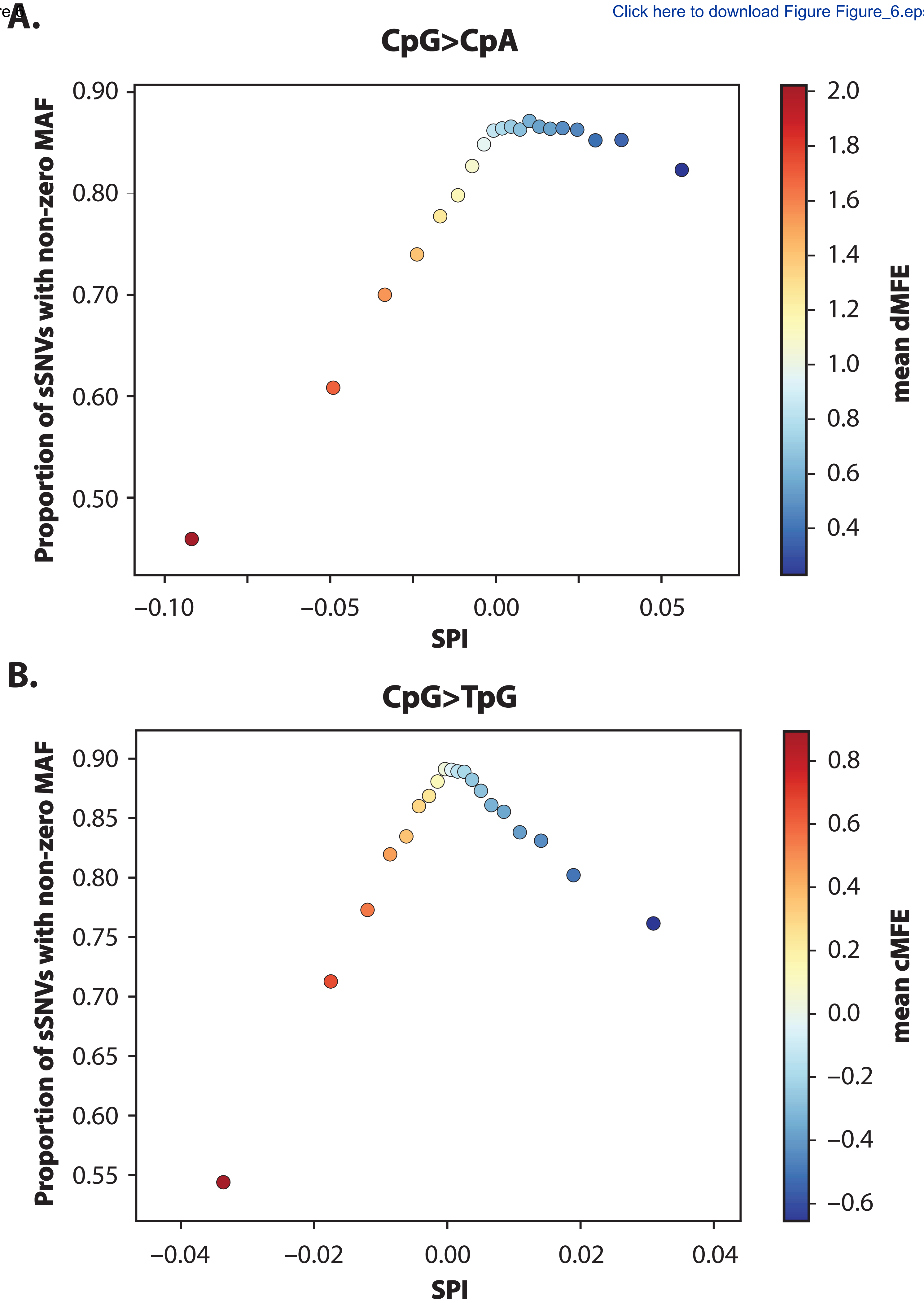

Click here to access/download
Supplementary Material
RNA_stability_supplementary_data.pdf
