# Supplementary material for: Synonymous variants that disrupt messenger RNA structure are significantly constrained in the human population
Source: Gigascience. 2021 Apr 5;10(4):giab023. doi: 10.1093/gigascience/giab023 (PMC8023685; doi:10.1093/gigascience/giab023)
Supplement: giab023_GIGA-D-20-00178_Revision_1 [file giab023_giga-d-20-00178_revision_1.pdf]

GigaScience

Synonymous Variants that Disrupt mRNA Structure are Significantly Constrained in the Human Population

--Manuscript Draft--

**Manuscript Number:**	GIGA-D-20-00178R1
**Full Title:**	Synonymous Variants that Disrupt mRNA Structure are Significantly Constrained in the Human Population
**Article Type:**	Research
**Funding Information:**	
**Abstract:**	Background: The role of synonymous single nucleotide variants in human health and disease is poorly understood, yet there is a growing body of evidence to suggest that this class of "silent" genetic variation plays multiple regulatory roles in both transcription and translation. One mechanism by which synonymous codons direct and modulate the translational process is through alteration of the elaborate structure formed by single-stranded mRNA molecules. While tools to computationally predict the impact of non-synonymous variants on protein structure are plentiful, analogous tools to systematically assess how synonymous variants might disrupt mRNA structure are lacking.<br>Results: To address this need, we developed novel software using a parallel processing framework for large-scale generation of secondary RNA structures and folding statistics for the transcriptome of any species. Focusing our analysis on the human transcriptome, we calculated 5 billion RNA folding statistics for 469 million single nucleotide variants in 45,800 transcripts. By considering the impact of all possible synonymous variants globally, we discover that synonymous variants predicted to disrupt mRNA structure have significantly lower rates of incidence in the human population.<br>Conclusions: These findings support the hypothesis that synonymous variants may play a role in genetic disorders due to their effects on mRNA structure. Given that the community lacks tools to evaluate the potential pathogenic impact of synonymous variants, we provide RNA stability, edge distance and diversity metrics for every nucleotide in the human transcriptome and introduce a "Structural Predictivity Index" (SPI) to quantify structural constraint operating on any synonymous variant. Because no single RNA-folding metric can capture the diversity of mechanisms by which a variant could alter secondary mRNA structure, we generated a SUmmarized RNA Folding (SURF) metric to provide a single measurement to predict the impact of secondary structure altering variants in human genetic studies.
**Corresponding Author:**	Peter White, Ph.D.<br>Nationwide Children's Hospital<br>Columbus, OH UNITED STATES
**Corresponding Author Secondary Information:**	
**Corresponding Author's Institution:**	Nationwide Children's Hospital
**Corresponding Author's Secondary Institution:**	
**First Author:**	Jeffrey B.S. Gaither, Ph.D.
**First Author Secondary Information:**	
**Order of Authors:**	Jeffrey B.S. Gaither, Ph.D.
	Grant E. Lammi
	James L. Li
	David M. Gordon
	Harkness C. Kuck
	Benjamin J. Kelly
	James R. Fitch

Powered by Editorial Manager® and ProduXion Manager® from Aries Systems Corporation

	Peter White, Ph.D.
**Order of Authors Secondary Information:**	
**Response to Reviewers:**	We thank the reviewers for their consideration and insightful feedback on our manuscript, “Synonymous Variants that Disrupt mRNA Structure are Significantly Constrained in the Human Population.” The critiques were very helpful, and in addressing them we believe the manuscript is substantially improved over our original submission. In summary, we made the following major revisions to the manuscript:<br><br>•Added survey of existing synonymous variant scores.<br>•Expanded discussion of conservation for RNA structure.<br>•Expanded description of link between RNA structure, gene expression and codon optimality.<br>•Converted our whole set of SNVs from GRCh37 to GRCh38 and added data from an additional 76,000 healthy individuals (gnomAD version 3), giving us both WGS and WES population data and augmenting core dataset from 18 million to 21 million sSNVs.<br>•Updated Figure 3-6 with new GRCh38 and gnomAD data. As population frequency data is improved by direct mapping to GRCh38, we dropped the extra “Liftover” step from Fig. 2.<br>•Added predicted binding-status to SPI so that it works consistently across all contexts and re-scaled as to PHRED values for easy interpretation.<br>•To simplify useability, we added a new Summarized RNA-Folding (SURF) score which takes maximum PHRED-score from any of our 11 RNA metrics. We added Figure 7 showing connection to population constraint.<br>•Updated software license to remove any commercial restrictions, and registered application with bio.tools and SciCrunch.org.<br><br>Please also find attached a red-line version of the manuscript that highlights all changes from the original submission. Additionally, we provide detailed responses to each reviewer’s critiques, below.<br><br>-- RESPONSE TO REVIEWER #1 --<br><br>1.The introduction misses a large volume of previous work on classification of synonymous variants. Please read Zishuo et al. Front. Genet., 10: 914 (2019) for some of those missing articles. For example, there were "four sSNV-specific effect predictors: SilVA (Silent Variant Analyzer) (Buske et al., 2013), regSNPs-splicing (Zhang et al., 2017), DDIG-SN (Detecting Disease-causing Genetic SynoNymous variants) (Livingstone et al., 2017), and IDSV (Identification of Deleterious Synonymous Variants) (Shi et al., 2019)."<br><br>•We thank the reviewer for bringing these tools to our attention. We had originally omitted these tools as the focus of our manuscript is on synonymous variants that impact mRNA structure, and tools like SiLVA focus on splicing. However, we agree that when discussing synonymous variants, it is appropriate to include background on all tools for sSNVs. As such we have added a paragraph to that surveys these tools and also describe a novel one (SynTool) (BACKGROUND, P5)<br><br>2.The authors also seem to be unaware of a previous demonstration of the relations between minor allele frequency of single nucleotide variants and base-pairing probabilities at the mutation site and between MAF and RNA solvent accessibility at the mutation site (Yang et al. RNA 23:14-22, 2016).<br><br>•We thank the reviewer – we were indeed not aware of this paper. We address Yang’s tool RNAsnap in the context of our own work, describing its focus on protein-bound tertiary structures as well as its finding of a correlation between RNA structure and population frequencies. (BACKGROUND, P4)<br><br>3.Figure 3: it would be interesting to examine the relation between MAF and dMFE/CFEED/dCD (and other structural properties) as the literature above.<br><br>•We agree that a study of the relation of our structural metrics to log(MAF) (as was performed in Yang et al., 2016) would be most interesting. We have added

Powered by Editorial Manager® and ProduXion Manager® from Aries Systems Corporation

	SUPPLEMENTARY FIGURE 7 showing the correlation of log(MAF) against the metrics dMFE, CFEED and dCD (BACKGROUND, P4).<br>•NOTE: to build these plots, we had to decide on a value of log(0). (This was not a problem for Yang et al., since 1000 Genomes contains only SNVs with positive MAFs). We adopted two separate approaches: setting log(0)=-6 (a reasonable value, since the gnomAD cohort has around 100,000 individuals, meaning the lowest possible log(MAF) is -5), or throwing out all SNVs with MAF=0. If we take the first approach, we essentially recover FIGURE 3. As might be expected, if we take the second approach (resulting in the loss of about 80% of our data) the pattern is lost. This seems to justify our decision to use P(MAF>0) as our primary y-statistic.<br><br>4.Figure 4: can you explain why peaked for dMFE at 2?<br><br>•This peak reflects the dominant contribution from CpG transitions, which have the highest gnomAD frequencies and an average dMFE of around 2. We added a note mentioning this important detail. (ANALYSIS, P7).<br><br>5.There are much more than a few examples of pathogenic SNVs shown in Table 2. In fact, there are a few thousands in HGMD (Stenson, et al. Human Genetics, 136(6), 665-677(2017)). A few datasets are available online to download. Authors should examine more examples for those neutral (from 1000 genomes) and pathogenic SNVs.<br><br>•The reviewer is quite correct that there are thousands of known pathogenic SNVs in HGMD. However, our goal was to present sSNVs that caused disease through their disruption of mRNA structure, and the HGMD does not consider RNA structure as an etiology, only including synonymous variants if they occur in splicing or regulatory regions. (Stenson et al., 2017, PMID 28349240, Table 1). The lack of mRNA structurally altering synonymous variants in databases such as ClinVar and HGMD was actually one of the motivating factors for our study, and it is one that the synonymous-variant community must grapple with. To lend credibility to this claim, we note that a 2019 Nature Communications article on synonymous mutations in cancer asserts that “no changes in RNA secondary structures of cancer genes have been proven so far.” (Nat Commun 10, 2569 (2019) https://doi.org/10.1038/s41467-019-10489-2).<br>•For the purposes of evaluating our metrics against known sSNVs that disrupt mRNA structure the best resource we could find was the dbDSM (the Database of Deleterious Synonymous Mutations) which only had 17 sSNVs tagged specifically as disease-causal via their effect on mRNA structure. After checking the original publications of these 17 sSNVs, we found only 6 where we felt that true causality for RNA structure had been demonstrated (several other publications speculated a relationship but did not perform the necessary functional work). Through extensive literature searching we found an additional three sSNVs variants that clearly demonstrate a pathogenic disease etiology through alteration in mRNA structure and have added them to TABLE 2. For the 9 known sSNVs clinically implicated for mRNA structural pathogenicity, all of them are successfully predicted to be pathogenic by our structural metrics. (TABLE 2, P40).<br>•With regard to the addition of neutral SNVs in Table 2 – we understand the reviewer’s wish to have neutral sSNVs represented in the table. However, given that the vast majority of our sSNVs are predicted to be structurally neutral by our metrics, this would require adding a large sample of neutral sSNVs to give a good representation. We did however modify our core dataset to include the more recently released gnomAD 3 data, which included the addition of WGS data for over 76,000 healthy subjects and updated all figures accordingly (see DATA DESCRIPTION, P7). We also have uploaded the complete dataset to GigaDB (see AVAILABILITY OF SUPPORTING DATA AND MATERIALS, P25).<br><br>6."However, the potential for pathogenic synonymous variants that impact RNA folding in human genetic disease remains largely unknown". This statement is incorrect as such an effect was demonstrated before. I would like to see the discussion changed.<br><br>•The reviewer is correct – it is now scientific dogma that such variants exist. We modified the discussion, changing “remains largely unknown” to “is not universally appreciated.” (DISCUSSION, P15)

Powered by Editorial Manager® and ProduXion Manager® from Aries Systems Corporation

7."In the absence of functional tools that would aid in the simultaneous assessment of both nsSNVs and sSNVs". This statement is incorrect as these tools do exist. I would like to see the discussion changed.

•We agree with the reviewer's recommendation. We changed "In the absence of functional tools" to "Given the scarcity of RNA-structure-specific tools". (DISCUSSION, P15)

-- RESPONSE TO REVIEWER #2 --

1.This discussion of the relationship between mRNA secondary structure/thermodynamic stability and mRNA intracellular stability/propensity to degradation is very vague. The authors failed to cite some key publications in the field (e.g. Ding et al., In vivo genome-wide profiling of RNA secondary structure reveals novel regulatory features. Nature, 2014; Mauger et al, mRNA structure regulates protein expression through changes in functional half-life, PNAS, 2019, see also ref. therein).

•We thank the reviewer for raising this valid criticism and have greatly expanded the BACKGROUND (P3) to further explain that "RNA stability" refers to the RNA's physical structure, which derives primarily from base-pair stacks, rather than to its functional lifetime or its ability to avoid degradation. We emphasize however that stable RNAs do, in fact, have longer lifetimes, express more protein and suffer later degradation, and hypothesize this is the preeminent reason why stability is selected for in the first place. In the DISCUSSION (Molecular mechanisms underlying constraint of sSNVs, P17-18) we elaborate that stable RNA structures are thought to regulate ribosomal speed, possibly with the goal of preventing collisions, which are known to trigger degradation pathways. We discuss Ding et al., 2014 and Mauger et al., 2019 as well as several other publications cited by them, including:
oPresnyak V et al. Codon optimality is a major determinant of mRNA stability. Cell. 2015 Mar 12;160(6):1111-24. doi: 10.1016/j.cell.2015.02.029. PMID: 25768907
oWan Y et al. Landscape and variation of RNA secondary structure across the human transcriptome. Nature. 2014 Jan 30;505(7485):706-9. doi: 10.1038/nature12946. PMID: 24476892
oMustoe AM et al. Pervasive Regulatory Functions of mRNA Structure Revealed by High-Resolution SHAPE Probing. Cell. 2018 Mar 22;173(1):181-195.e18. doi: 10.1016/j.cell.2018.02.034. Epub 2018 Mar 15. PMID: 2955126
oKertesz M. Genome-wide measurement of RNA secondary structure in yeast. Nature. 2010 Sep 2;467(7311):103-7. doi: 10.1038/nature09322. PMID: 20811459

2.The interplay between local and global mRNA secondary structure and mRNA intracellular stability as well as overall protein expression and folding/activity has not been fully understood yet. While it has been suggested that nucleotides that stabilize mRNA secondary structure within the coding regions do enable high protein expression, the effect of such variants in the 5'UTRs is usually quite the opposite, as the increase in 5'UTR stability usually decreases efficiency of translation initiation. The authors should carefully discuss these different scenarios in the context of their observations.

•We thank the reviewer for this relevant point, which we agree should be included. We have added a couple sentences explaining that, in contrast to the CDS, stability in 5' UTRs is associated with decreased expression due to the interference of structure with translation initiation. (BACKGROUND – P3-4)
•Interestingly, in most organisms this effect extends into the coding region – the first 30 or so bases have reduced stability (Gu et al., doi:10.1371/journal.pcbi.1000664) However, warm-blooded animals are partially an exception to this rule – it holds only in the most G+C-rich genes, which may be attributable to the CpG islands that generally co-locate with mammal and bird gene promoters. We also describe this extension of this effect into the coding region, and why mammals might be an exception. DISCUSSION (Molecular mechanisms underlying constraint of sSNVs, P17)

3.Notably, because in the human genome optimal codons tend to be GC rich, the structure of the genetic code inherently and generally disallows coding sequences with

Powered by Editorial Manager® and ProduXion Manager® from Aries Systems Corporation

both highly optimal codons and low structure/thermodynamic stability. The authors should also investigate the limits and the impact of codon usage/codon optimality on the secondary structure. How many pathogenic sSNVs would lead to a change of an optimal codon to a non-optimal codon?

•We thank the reviewer and agree that we did not give due attention to codon optimality. We have added SUPPLEMENTARY FIGURE 8, which shows no depletion or enrichment of mutations that increase/decrease optimality, suggesting it is not a major factor in our findings. We also note that in most REF>ALT contexts the change in optimality (increase or decrease) is largely the same over all codon classes. Thus, our decomposition of all mutations into 14 contexts has largely accounted for differences in codon optimality. However, it is of note that we do observe more constraint in mutations G+C > A+T, that is, the optimal to sub-optimal direction. We certainly do not rule out the effects of codon optimality, which is why we included the tRNA Adaptation Index (tAI) in our list of null features for SPI. (BACKGROUND, P4, DISCUSSION, P17-18)

-- RESPONSE TO EXTERNAL EDITORIAL ADVISOR --

Major:

1.It would be useful to know if the mediator variables, in particular the ones that are up-stream or downstream nucleotides have a simple, mechanistic explanation in terms of the energy model RNA folding. Since folding energies in paired regions depend not just on the base pair but on the stacking interaction with the adjacent base pairs, there may be a strong correlation of effects with adjacent nucleotides that could simply be read of the pairing tables in the Turner energy model. The authors should check whether this already provides an explanation. Similarly, CpGs in RNA have a correlation with base pairing since the stacking of GC with CG pairs has the largest stabilizing energy contribution, whence secondary structure have a propensity for CpG pairing with CpG.

•We thank the reviewer for this interesting point, upon which we have added a paragraph in the DISCUSSION. Interestingly, the sign of the Mediator indicates that stacks are enriched for mutations, not constrained. We hypothesize that this observation could be due to stable structures having a greater tolerance for de-stabilization than unstable structures. Relatedly, we observe the Mediator usually has the same S/W type as the reference (e.g. a trailing T in the A>G context). The co-occurrence of A/Ts suggests that unstable regions are in little danger of over-stabilization. (DISCUSSION, P14-15)

Minor:

1.This study is not the first to identify large-scale selection pressures on RNA structures throughout in a genome/transcriptome-wide setting. While to my knowledge the approach here is novel, prior work on this topic should be to be referenced and the conclusions need to be discussed at least briefly also in this context. See e.g. Seemann et al doi: 10.1101/gr.208652.116 and the references therein. A paper that explicitly considers structural constraints in mRNAs is, albeit for fruitfly https://www.sciencedirect.com/science/article/pii/S0300908411002811?via%3Dihub

•We thank the reviewer for bringing these works to our attention and agree that our description of related studies was inadequate. In addition to the two references suggested we have added descriptions of RNAsnap and SNPfold, two tools that assess disruptions of RNA structure, the former using population frequencies like our own work. We also cite the secondary-structure prediction tools used in Seemann (CMFinder) and the reviewer’s other reference Findeiss (RNAz) (BACKGROUND, P4).

2.Consider using the more common notation capital-Delta for differences in MFE etc.

•We agree with the reviewer’s suggestion and have changed structural metric name “dMFE” to “ΔMFE” throughout, similarly for the metrics dEFE, dCFE, dMEAFE, dCD and dEND.

Powered by Editorial Manager® and ProduXion Manager® from Aries Systems Corporation

	3.the terminology CFEED (Centroid Free Energy Edit Distance) is awkward, why the "free energy" in the name? Usually these quantities are referred to as base pair distances since they just count the caradinality of the set of base pairs in which two structures differ. Or are you using a different edit distance?<br><br>•Changed Also changed CFEED to CED due to the phrase “free energy” not being relevant. Analogously, EFEED has been changed to EED.<br><br>4.Also the definition of the metrics that are used in the main text should be given there instead of being relegated to the supplement.<br><br>•We have recast so that the three metrics are defined in the same sentences they are introduced. (DATA DESCRIPTION, P6)<br><br>5.how were positions closer than 50 nt to the ends of transcripts handled?<br><br>•We certainly should have mentioned this! For such positions we just take the leading/trailing 101 bases as our 101-base window. Added explanation. (METHODS, P19)<br><br>6.spell out acronyms at first usage<br><br>•This has been fixed throughout.<br><br>7.-avoid * as multiplication symbol, use standard mathematical notation<br><br>•This has been fixed throughout the text and figures.
Additional Information:	
Question	Response
Are you submitting this manuscript to a special series or article collection?	No
**Experimental design and statistics**<br><br>Full details of the experimental design and statistical methods used should be given in the Methods section, as detailed in our Minimum Standards Reporting Checklist. Information essential to interpreting the data presented should be made available in the figure legends.<br><br>Have you included all the information requested in your manuscript?	Yes
**Resources**<br><br>A description of all resources used, including antibodies, cell lines, animals and software tools, with enough information to allow them to be uniquely identified, should be included in the Methods section. Authors are strongly	Yes

encouraged to cite Research Resource Identifiers (RRIDs) for antibodies, model organisms and tools, where possible.<br><br>Have you included the information requested as detailed in our Minimum Standards Reporting Checklist?	
**Availability of data and materials**<br><br>All datasets and code on which the conclusions of the paper rely must be either included in your submission or deposited in publicly available repositories (where available and ethically appropriate), referencing such data using a unique identifier in the references and in the “Availability of Data and Materials” section of your manuscript.<br><br>Have you have met the above requirement as detailed in our Minimum Standards Reporting Checklist?	Yes

Manuscript (doc) Click here to access/download;Manuscript;RNA_stability_manuscript_GS.doc

Synonymous Variants that Disrupt mRNA Structure are Significantly Constrained in the Human Population

Jeffrey B.S. Gaither[1], Grant E. Lammi[1], James L. Li[1], David M. Gordon[1], Harkness C. Kuck[1], Benjamin J. Kelly[1], James R. Fitch[1] and Peter White[1,2,*]

[1] Computational Genomics Group, The Institute for Genomic Medicine, Nationwide Children's Hospital, Columbus, Ohio, USA

[2] Department of Pediatrics, College of Medicine, The Ohio State University, Columbus, Ohio, USA

* To whom correspondence should be addressed. Tel: +1 (614) 355-2671; Fax: +1 (614) 355-6833; Email: peter.white@nationwidechildrens.org

Mailing address:

Prof. Peter White, PhD
The Institute for Genomic Medicine
Nationwide Children's Hospital
575 Children's Crossroad
Columbus, OH 43215. USA

Running title: RNA Structural Constraint and Synonymous Variants

Abstract

Background: The role of synonymous single nucleotide variants in human health and disease is poorly understood, yet there is a growing body of evidence to suggest that this class of "silent" genetic variation plays multiple regulatory roles in both transcription and translation. One mechanism by which synonymous codons direct and modulate the translational process is through alteration of the elaborate structure formed by single-stranded mRNA molecules. While tools to computationally predict the impact of non-synonymous variants on protein structure are plentiful, analogous tools to systematically assess how synonymous variants might disrupt mRNA structure are lacking.

Results: To address this need, we developed novel software using a parallel processing framework for large-scale generation of secondary RNA structures and folding statistics for the transcriptome of any species. Focusing our analysis on the human transcriptome, we calculated 5 billion RNA folding statistics for 469 million single nucleotide variants in 45,800 transcripts. By considering the impact of all possible synonymous variants globally, we discover that synonymous variants predicted to disrupt mRNA structure have significantly lower rates of incidence in the human population.

Conclusions: These findings support the hypothesis that synonymous variants may play a role in genetic disorders due to their effects on mRNA structure. Given that the community lacks tools to evaluate the potential pathogenic impact of synonymous variants, we provide RNA stability, edge distance and diversity metrics for every nucleotide in the human transcriptome and introduce a "Structural Predictivity Index" (SPI) to quantify structural constraint operating on any synonymous variant. Because no single RNA-folding metric can capture the diversity of mechanisms by which a variant could alter secondary mRNA structure, we generated a SUmmarized RNA Folding (SURF) metric to provide a single measurement to predict the impact of secondary structure altering variants in human genetic studies.

Keywords: synonymous variant, RNA structure, mRNA stability, genetic disease, Apache Spark, genomics

BACKGROUND

Accurate molecular genetic diagnosis of a rare disease is essential for patient care [1], yet today's best molecular tests and analysis strategies leave 60-75% of patients undiagnosed [2-6]. Current clinical practice for sequence variant interpretation focuses primarily on missense, nonsense or canonical splice variants [7], with numerous computational methods for prediction of the impact of non-synonymous single-nucleotide variants (**nsSNVs**) on protein function [8]. By contrast, we have limited knowledge in regard to the role that synonymous variants (**sSNVs**) may have in health and disease. These variants modify the codon in a transcript but leave the protein unchanged, and for years were erroneously considered to be "silent." However, the past two decades have seen a growing understanding that synonymous codons serve vital regulatory functions [9-12].

One of the principal levers by which synonymous codons direct the translational process is through mRNA structure. Unlike DNA, a messenger RNA (**mRNA**) molecule is single-stranded and therefore capable of forming complex configurations largely by base-pairing with itself, yielding the *secondary structure*, which further folds through covalent attractions to form the *tertiary structure* (**FIGURE 1**) [13]. The secondary structure has proven to be essential for understanding the regulatory functions of RNAs, and sophisticated methods exist to predict the ensemble of possible structures a given mRNA strand can adopt [14]. An important physical property of an RNA structure is its stability, which is defined as the extent to which an RNA molecule retains its structural integrity. RNA stability is largely a function of G+C content of the molecule in question, although most of the energy comes from the stacking-energy of the G=C pairs rather than the pairs individually [15].

Studies first published in 1999 indicated that stable mRNA secondary structures are selected for in key genomic regions across all kingdoms of life [16-19]. Stable RNA has a longer functional half-life, being more resistant to degradation or base-catalyzed hydrolysis, and stronger coding structures can endure more rounds of translation, ultimately resulting in more protein [17, 20-25]. Repeated translation de-stabilizes an RNA, weakening the brakes on ribosomal translational speed and producing collisions that trigger decay pathways [26-31]. There are however cases where *weak* structure is more desirable, most notably in the 5′ untranslated regions (**UTR**) and around the start codon, to make it easier to commence translation [17, 32-37]. Diminished stability in stress-response genes may also permit a more dynamic response of the cell to stress [33]. The stability of an mRNA transcript can

also determine the speed of translation [16, 18, 19, 29, 38, 39] and vitally facilitate or prevent miRNAs and RNA-binding proteins from attaching to specific structural motifs [40-44]. Studies have also strongly linked mRNA structure to protein conformation and function, with synonymous codons acting as a subliminal code for the protein folding process [12, 29, 45-50]. Given all these mechanisms, when synonymous variants are ignored, we are almost certainly missing novel plausible explanations for genetic disease.

The growing understanding of the importance of RNA structure has inspired a rich literature of *in silico* secondary structure prediction methods. One culminating study looked at predicted structures across the genomes of 17 vertebrates and found 516,000 structurally conserved elements across species, with the most conserved structures lying in coding regions [51]. An analogous work focusing on 23 drosophilids and four other insect species found 345,000 structurally conserved elements [52], and recently a study on the whole Tree of Life found comparable conservation [53]. As we have employed in this present work, all three of these previous studies utilized the ViennaRNA package [14] (or tools built to utilize it, such as CMFinder [54] and RNAz [55]). In an alternative approach, one group trained a machine-learning algorithm called RNAsnap on both single and multiple-aligned sequences to predict solvent accessibility in protein-bound RNA tertiary structures [56]. Anticipating our study, the authors found decreased minor allele frequencies (**MAF**) in the 1000 Genomes database [57] at structurally significant positions (**Supplementary Figure 7** shows that the pattern of constraint observed with p(MAF)>0 is maintained when using the log(MAF) statistic used by Yang *et al.* [56]). In a study similar to ours but more limited in scope, the authors compared wildtype and mutated predicted structures to identify "RiboSNitches" or structurally disruptive SNVs in 5′ UTRs [58, 59]. However, the authors were limited by the computational cost of computing folding statistics for every SNV of interest.

Despite the widespread scientific interest in mRNA structure, its role in human health and disease remains poorly comprehended and relatively few pathogenic synonymous variants impacting mRNA folding have been described [20, 21, 23, 25]. A structure-altering sSNV in the dopamine receptor DRD2 inhibited protein synthesis and accelerated mRNA degradation [60]. A sSNV in the *COMT* gene, implicated in cognitive impairment and pain sensitivity, was shown *in vitro* to constrain enzymatic activity and protein expression [61]. A sSNV in the *OPTC* gene of a glaucoma patient resulted in decreased protein expression *in vivo* [62]. In cystic fibrosis patients, a sSNV

in *CFTR* was linked to decreased expression [63], and an mRNA-secondary-structure-altering silent codon change contributed to *CFTR* dysfunction by altering the dynamics of translation leading to protein misfolding [22, 24]. Two sSNVs in *NKX2-5*, identified in patients with congenital heart disease, decreased the mRNA's transactivation potential [64]. In hemophilia B, a sSNV in the factor IX gene impacted the transcript's secondary structure and reduced extracellular protein levels [65], and both synonymous and nonsynonymous variants were shown more likely be deleterious when occurring in a stable regions of *F8* and *DMD* mRNAs [66]. Our understanding of the role of synonymous variants in cancer is rapidly expanding, with recent studies demonstrating that they may act as drivers of the disease [67-69], altering the function of oncogenes such as RET [70] and KRAS [71].

While there are numerous methods to predict the impact of amino acid altering and regulatory variation, relatively few approaches have been developed to identify functional sSNVs. Of the five synonymous variant metrics we found in the literature, only two utilize RNA-folding statistics – SiLVA [72] and DDIG-SN [73] – and in each case the authors emphasize the structural features make almost no difference to the model. These scores primarily excel at identifying splicing defects, and the same is true for other synonymous scores such as IDSV [74], regSNPs-splicing [75] and Syntool [76]. There are, in contrast, tools that measure disruptions of RNA folding, albeit not exclusively in synonymous variants – the three most prominent are the webservers RNAsnp [77], SNPfold [59] and MutaRNA [78]. These three webservers perform largely the same task, comparing predicted wildtype and mutated structures and returning the change in base-pairing probabilities and or/ visualizations of the structures themselves. However, the three tools are limited to the assessment of a single variant, requiring an on-the-fly calculation for every SNV under consideration, making them unsuitable for scoring sSNVs in the 4-6 million variants typically identified from genome sequencing of a single individual. To the best of our knowledge there are no precalculated transcriptome-wide scores well-equipped to model sSNVs that specifically alter RNA structure.

Given the established importance of RNA structure, we hypothesize that there may be many more as yet to be identified sSNVs that can provoke genetic disorders through their disruption of RNA structural elements. As such, the aims of this study were the creation of RNA-structural metrics for every possible single nucleotide variant (**SNV**) and to evaluate whether structure-disrupting sSNVs are constrained in the human population. Through

developing methods to predict whether a SNV is “structurally pathogenic,” we hope to drive the discovery of novel genetic etiologies in both monogenic genetic disorders and more complex human disease.

DATA DESCRIPTION

Raw Dataset

To obtain all human mRNA transcripts we downloaded the NCBI RefSeq Release 81 from an online repository (ftp://ftp.ncbi.nlm.nih.gov/refseq/H_sapiens/mRNA_Prot/). Transcript sequences corresponded to human reference genome build GRCh38.

Massively parallel generation of RNA stability metrics

To assess the impact of synonymous mutations on mRNA structure, we carried out a genome-wide computation in which folding statistics were calculated for every possible variant in the human transcriptome (RefSeq Release 81, GRCh38). For each position in all transcripts, we built a 101-base window centered around the reference and three alternate sequences with the alternate allele substituted at the 51st position. We applied the ViennaRNA software package to the wildtype and mutated sequences to obtain 10 folding metrics quantifying the structural disruption caused by all three possible SNVs at the position (see **SUPPLEMENTARY DATA TABLE 1** for metric details). Computing this dataset of structural predictions for nearly half a billion SNVs was truly a “big data” computational task. We relied heavily on the parallelizability of the Apache Spark framework and custom wrappers which adapted the ViennaRNA software package to run within the Hadoop framework (**FIGURE 2**). Details of the calculation and subsequent assignment of variants into classes are given in **METHODS**.

Of the 10 mRNA-structural metrics output by our Vienna implementation, we adopted three as central to our analysis: delta Minimum Free Energy (**ΔMFE**), Centroid Edge Distance (**CED**), and delta Centroid Distance (**ΔCD**). The metric ΔMFE measures the change in mRNA free energy or “stability” caused by the sSNV, while CED gives the number of base pairs that vary between the mutant and wildtype centroid structures. The metric ΔCD measures the sSNV’s effect on the diversity of the mRNA’s structural ensemble, which is the collection of various

structures that a given sequence can exhibit. Distributions of these metrics, along with the other 7 mRNA-structural metrics output by our RNA structure pipeline are presented in **SUPPLEMENTARY DATA FIGURE 1**.

To test whether certain sSNVs are under constraint due to their effect on mRNA structure, we utilized population frequencies from the Genome Aggregation Database (gnomAD) containing aggregate genome and exome sequencing data from a total of 201,904 unrelated human individuals (gnomAD v2.1 data set contains data from 125,748 exomes mapped to the GRCh37/hg19 reference sequence and lifted over the GRCh38; the gnomAD v3.1 data set contains 76,156 whole genomes (and no exomes), all mapped to the GRCh38 reference sequence) [79]. Our expectation was that SNVs with disruptive structural properties would be found less frequently in human populations. We defined a variant to be constrained if it was absent from both gnomAD v2.1 and v3.1 datasets and un-constrained if it had a MAF > 0 in either set, a strategy similar to that employed by other groups [80, 81].

ANALYSIS

Global constraint to maintain stability

Our study reveals a striking connection between a given SNV's impact on mRNA structure and its frequency in the gnomAD database. This central finding is summarized in **FIGURE 3**, which depicts the proportion of SNVs with gnomAD MAF>0 at every value of our stability-metric ΔMFE. All four variant classifications – synonymous, 5′-UTR, 3′-UTR and missense – show a bi-directional constraint to maintain the wild-type mRNA structure. When the SNV either weakens the mRNA structure (high ΔMFE) or strengthens it (low ΔMFE) the SNV is depleted in the population roughly in proportion to the level of disruption. While this pattern of constraint was observed across all four variant classes, **FIGURE 3** indicates that it is strongest for synonymous variants.

FIGURE 4 summarizes constraint in the synonymous case, showing the relationship of our three main structural metrics with gnomAD frequency. **FIGURE 4A** recapitulates the pattern of green circles in **FIGURE 3**, revealing that disrupting mRNA stability decreases the chance of a synonymous SNV's appearing in human mRNA transcripts. The global peak at ΔMFE=2 reflects the dominant contribution of CpG transitions, which tend to be de-stabilizing – see **DISCUSSION**: *CpG transitions have constraint against de-stabilization of their mRNA structures.* The effect of removing or creating new base-pairings, quantified by the metric CED, is shown in **FIGURE 4B** (see

SUPPLEMENTARY DATA FIGURE 2 for an illustration of how CED is calculated). This figure validates our basic hypothesis that structurally disruptive sSNVs should appear less frequently in the population. We see that sSNVs which leave the centroid structure unchanged (i.e. CED=0) are roughly 15% more common than those sSNVs predicted to alter it, and SNVs with large CED values are constrained in proportion. Our third metric ΔCD measures change in the diversity of the mRNA ensemble (that is, the collection of all the structures formed by millions of *in vivo* mRNAs) and is shown in **FIGURE 4C**. This figure illustrates that changes in diversity – either towards more or less – are also constrained in gnomAD. The symmetry in depletion between over- and under-diversifying sSNVs is surprisingly regular. Analogous plots for the remaining seven structural metrics can be viewed in **SUPPLEMENTARY DATA FIGURE 3**.

The color-coding in **FIGURE 4** illuminates the relationship between the three structural metrics. Changes in stability are correlated with changes in base-pairing and vice-versa, as demonstrated by the red values at the extremes of each distribution. **FIGURE 4C** depicts a clear relationship between diversity and stability, with those sSNVs that diversify the ensemble (high ΔCD) also tending to weaken it (red). This diversity-instability relationship is intuitive, as a destabilizing mutation "frees up" portions of the mRNA to assume new shapes.

Variation of constraint with REF>ALT context

We next set out to determine if the constraint demonstrated in **FIGURE 4** holds uniformly for all synonymous nucleotides or whether it varies in different REF>ALT contexts. We would expect the latter as the bases C and G form much stronger structural bonds than do A and T. To probe this question we divide our sSNVs into 14 classes (**TABLE 1):** 12 classes based on their reference and alternate mRNA alleles (e.g. A>C, C>G, T>C, etc.) and 2 additional classes based on potential loss of methylated cytosine (CpG>TpG or CpG>CpA, the latter of which results from a deamination on an antisense strand). For consistency and clarity, we treat thymine as an mRNA base, even though it is actually replaced by uracil in mRNA. Then within each REF>ALT context we reconstruct the three plots of **FIGURE 4** and also perform weighted linear (or quadratic, for ΔCD) regressions between the three different stability metrics and the probability that the gnomAD minor allele frequency is > 0 (see **METHODS** for details and **SUPPLEMENTARY DATA TABLE 2** for full regression statistics).

We observe that constraint for mRNA structure is highly dependent on mutational context (**Table 1**). Some REF>ALT contexts show constraint in one direction only (e.g. against weakening of their structures), while other contexts show no significant constraint at all. The metric ΔMFE, which measures changes to mRNA energy or stability, shows a striking context-dependence (**Table 1A).** All significant REF>ALT changes are constrained *unidirectionally,* with one direction showing a depletion in population frequencies, while the other shows an enrichment (the direction of constraint is obtained by a weighted linear regression; see **Methods** for details). In line with our understanding of structural biochemistry of RNA folding, mutations from "strong" REFs (C and G, so called because they form strong Watson-Crick bonds) to "weak" ALTs A and T are constrained against high values of ΔMFE. i.e. against the weakening of structure. Conversely, mutations from "weak" to "strong" nucleotides are constrained against the strengthening of structure (low ΔMFE). The exception to this rule is the context G>A (see *Constraint for mRNA stability in non-CpG-transitional contexts*).

Evaluation of the base-pair metric CED demonstrates that some contexts are constrained against large changes in mRNA base-pairing, while in others, SNVs altering base-pairs are actually enriched (**Table 1B).** This result reflects the fact that in some contexts *small* base-pairing changes are enriched over *no* base-pairing changes. In keeping with our main hypothesis, large changes of base-pairing are still uniformly constrained. As was the case with ΔMFE, we again observe that the context G>A is the exception.

Finally, **Table 1C** shows mutational contexts that exhibit significant constraint against changes to ensemble diversity as measured by ΔCD. We see that only a few contexts exhibit this constraint. In the two CpG-transitional contexts, the bell-shaped pattern of **Figure 4C** is faithfully reproduced, with both decreases and increases to ensemble diversity being equally harmful. However, the context G>A is enriched for changes in diversity – this context is strangely aberrant when assessed with all three metrics.

CpG transitions have constraint against de-stabilization of their mRNA structures

The data in **Table 1** show that our observed constraint for mRNA structure is greatest in the case of CpG transitions. Since these variants (and their suppression) are crucial to the story of mRNA stability, it is important to have an appreciation of their role in a biochemical context. The dinucleotide CG (usually denoted CpG to distinguish

this linear sequence from the CG base-pairing of cytosine and guanine) is capable of becoming methylated and then mutating by a process called “deamination” into a TG dinucleotide; deaminations are also possible in unmethylated CpGs, but these result in a uracil that is quickly identified as a foreign base and repaired. In mammals 70-80% of CpGs are methylated, which makes a CpG transition about 4x more common than any other mutation-type among mammals (see **SUPPLEMENTARY DATA TABLE 3**) [82]. The nucleotides C and G also form foundational bonds in mRNA secondary structures. Most of the energy of an mRNA structure lies in its “stacks” of nucleotides with the average energy of a C-G pair in a stack around 65% stronger than that of any other base-pairing [83].

We find strong evidence that CpG transitions are constrained against weakening of their mRNA structures. This striking trend is largely explained (in a statistical sense) by CpG content, i.e. number of CpG dinucleotides in the vicinity (see “Proportion of variance explained by Mediator” in **TABLE 1**). **FIGURE 5** shows the populational constraint for our three main metrics in CpG-transitional contexts. Most strikingly, we find that synonymous CpG>CpA and CpG>TpG mutations both show a steady constraint against weakening of mRNA structure (high ΔMFE) (**FIGURES 5A & 5B**). Fascinatingly, both contexts exhibit a cluster of outliers in the most destructive (i.e. most de-stabilizing region), suggestive of extreme constraint borne of significant structural disruption.

The behavior of the edge metric CED in these contexts is also clear-cut. In **FIGURES 5C & 5D** we see a clear constraint against mutations with high CED values, and the red coloring shows that such changes are, on average, de-stabilizing. We also observe a depletion at CED=0 in the CpG>TpG case; this is responsible for the bi-directional constraint reported in **TABLE 1**. Finally, **FIGURES 5E & 5F** show that the basic pattern of constraint for diversity in **FIGURE 4C** is reproduced and is essentially unchanged for both types of CpG transition. The coloring of **FIGURE 5** indicates that mutations CpG>CpA are more weakening on average than their CpG>TpG counterparts, despite being largely produced by the same biochemical mechanism (a CpG>TpG deamination on either the sense or anti-sense strand). We speculate on this disparity in the **DISCUSSION.**

Constraint for mRNA stability in non-CpG-transitional contexts

We observe a constraint for mRNA structure in most REF>ALT contexts (as indicated by **TABLE 1**). We can classify the remaining contexts based on whether they are constrained against weakening or strengthening of

their structures (as reported in **Table 1A**). **Supplementary Data Figure 4** shows plots of contexts where ΔMFE and gnomAD frequency are negatively correlated, i.e. where structure-weakening sSNVs are under constraint. Notably, all these contexts are strong>weak (or strong>strong in the case of C<>G), consistent with the principle that one purpose of such nucleotides is to maintain stability. In **Supplementary Data Figure 5** we show the contexts where ΔMFE and gnomAD frequency vary positively, which amounts to constraint against structure-strengthening sSNVs. Correspondingly, we note that two out of three of these contexts are weak>strong (and the third is the consistently aberrant context G>A).

Mediator variables

In **Table 1** we provide a "Mediator" variable for the connection between our RNA folding metrics and gnomAD frequencies in each mutational context. The name "Mediator" signifies that the variable explains some of the connection between the structural metric and gnomAD (details on how the Mediator and % variance explained are calculated are given in **Methods**.) These Mediators can explain large portions of the trends in **Figure 5** and **Supplementary Data Figures 4-5.** The striking trend between ΔMFE and gnomAD frequency in CpG-transitional contexts, for example, is largely driven by the local CpG content. CpG content is also the most powerful feature for CED and ΔCD in these contexts, with high CpG content consistently correlating with depletion. A plausible inference is that an abundance of CpGs signifies important mRNA structure whose disruption could be harmful.

In non-CpG-transitional contexts, the Mediator almost always proves to be a nucleotide upstream or downstream of the sSNV. In the context C>A we can recover 28% of the relationship between ΔMFE and gnomAD frequency simply by looking at whether the C is followed by a G. The power of CpG dinucleotides in recovering our structural trends emphasizes the effect of these dinucleotides on mRNA structure.

Global quantification of mRNA constraint

Our analysis shows that variants predicted to disrupt mRNA secondary structures are constrained in the population. However, the complexity of mRNA structure means focusing on one single metric will surely lead to

loss of information. To overcome this potential limitation of our RNA folding metrics, we set out to devise a more comprehensive method for predicting possible pathogenicity due to mRNA structure. Our strategy is to consider the *additional* statistical power bestowed by mRNA structure. In each context from **TABLE 1** we use RNA-sequence features (such as nearby bases and transcript position) to construct two separate models to estimate the probability that a sSNV will appear in gnomAD: an "active" model which incorporates our mRNA-structural metrics (P_s), and a null model which only uses sequence features (P_n). These models give us two separate estimates for the quantity P(MAF >0). Then we define the Structural Predictivity Index or **SPI** to be the log-quotient of the two probabilities:

$$\mathrm{SPI} = \log_{10}\left(\frac{P_s}{P_n}\right)$$

The metric SPI thus measures the predictive power bestowed by mRNA-structural variables. When it varies from 0, mRNA structural metrics yield new insight about a SNV's potential to have a functional role in mRNA secondary structure. The variation of gnomAD MAF with respect to SPI can be seen in **FIGURE 6**. We observe uniform constraint in SPI, validating the structural score P_s: when P_s is relatively low, SNVs are depleted; when it is relatively high, SNVs are enriched. This global relationship between SPI and constraint is also evident across all 14 sequence contexts (**SUPPLEMENTARY DATA FIGURE 6**). We show the power of SPI in each sequence context (given by its area under the curve in predicting whether gnomAD nonzero frequency is >0) in **SUPPLEMENTARY DATA TABLE 4**.

Finally, to simplify use of our RNA stability data set we calculated **SU**mmarized **R**NA **F**olding (**SURF**) metrics. Each of the ten RNA folding metrics and SPI, scores were percentile ranked and Phred-scaled ($-10 \times \log_{10}(\text{Percentile Rank})$), such that the larger the Phred-scaled value the greater the precited change in RNA structure. For each SNV in our dataset, the maximum Phred score across either all 11 metrics (***SURF***), across the four stability metrics (***SURF Stability***), across the four edge distance metrics (***SURF Edit Distance***), or across the two diversity metrics (***SURF Diversity***). Across all 4 summarized metrics, a clear correlation between global constraint and increasing score can be observed (**FIGURE 7)**.

Clinical Examples of Structural Pathogenicity

The literature reveals only a few examples of synonymous sSNVs unequivocally shown to be pathogenic through their effects on mRNA structure. These sSNVs, with accompanying values of our three ViennaRNA metrics, SPI and SURF, are listed in **TABLE 2.** This set of known pathogenic sSNVs show a clear enrichment for our structural metrics, with each exhibiting a value of ΔMFE, CED, ΔCD or SPI that is in the third quartile of distribution for the given score. All nine SNVs had a damaging SURF score, ranging from 9.5 to 18.6 (the 89th to 99th percentile). For example, one pathogenic sSNV in NKX2-5 (rs2277923), linked to congenital heart disease, has a SURF score in the 90th percentile [64]. It should be noted that none of these clinical sSNVs qualifies as a truly exceptional outlier for any of our ViennaRNA metrics or SPI; while all have SURF scores above the 89th percentile, none exceed the 99th percentile (see **DISCUSSION** for suggested score cutoff values).

DISCUSSION

We developed novel software to enable efficient generation of billions of RNA folding metrics for any species. This software allowed us to calculate RNA folding metrics for every base in the human transcriptome (approximately 0.5 billion SNVs). The RNA stability scores generated by this approach enable global assessment of synonymous variants and their potential role in human health and disease. We focused our analysis on the approximately 21 million synonymous variants found in the transcriptome, avoiding those sSNVs that could impact canonical splice sites and confound our analysis. Our study revealed that there is significant selection against sSNVs predicted to disrupt the given transcript's local mRNA secondary structure, supporting our hypothesis that RNA structure itself plays a critical role in human health and disease.

Multiple arguments support a true causal relationship behind RNA stability and the observed correlation with constraint in the human population. First, we tested our hypothesis using three qualitatively distinct measures of structural disruption: change in stability (ΔMFE), change in base-pairing (CED) and change in ensemble diversity (ΔCD). All three metrics showed that SNVs which alter mRNA structure are constrained in human populations.

Second, our study revealed some patterns which can be elegantly explained in terms of mRNA structure. We showed that strong>weak mutations such as C>A are only depleted when they weaken mRNA structure, while weak>strong mutations are only depleted when they strengthen it. We also found that sSNVs with extreme ΔMFE

and CED values are constrained even beyond the general trends (**FIGURE 5**), suggesting that this severe disruption is more-than-linearly unviable. Furthermore, **FIGURE 4B** highlights a pattern in CED values that alternates between high and low on successive values (CED can only take on even values because the destruction/creation of a base pair always requires two edits): the sSNVs with CED values that were multiples of 4 (4,8,12...) were shown to be enriched over those that were only multiples of 2 (2,6,10...). Such CED values are required if the total number of base-pairs is to be conserved, supporting that the constraint is needed to maintain overall base-pairing.

Third, the structural constraint we observe is not just restricted Watson-Crick base pairs, but also in nucleotides where wobble base pairing occurs. Wobble base-pairing takes place between two nucleotides such as guanine-uracil (G-U), that are not canonical Watson-Crick base pairs, but have comparable thermodynamic stabilities. We observed bi-directional constraint for ΔMFE in the context T>C, viewable in **SUPPLEMENTARY DATA FIGURE 5**. We conjecture the dual constraint in this context might be due to guanine's unique ability to wobble base-pair. Thus, the dual constraint from mutations T>C could be related to the transformation of T=G wobble base-pairs into stronger C=G Watson-Crick base pairs.

Finally, our Structural Predictivity Index (**SPI**), created specifically to control for all confounding factors, demonstrates a clear relationship between mRNA structure and constraint. When structural metrics decrease the model score, the gnomAD MAF is lower, whereas when structural metrics increase the model score, gnomAD MAF is higher (**FIGURE 6)**. This strongly suggests that our trends are direct and causal. This "proof-of-non-spuriousness" justifies our decision to regard sequence variables that contribute to mRNA structure – such as adjacent nucleotides and GC/CpG content – as Mediators (**TABLE 1**).

That many of the Mediators are adjacent nucleotides – leading C", "trailing G" and so on – suggests the reference and Mediator are set next to one another in a stable "stack," such stacks being the principal feature of mRNA structures. Our data shows that these stacks are more likely to be enriched for mutations, not depleted; suggesting that a strong structure has more tolerance to be de-stabilized, whereas a weak structure cannot. The trend operates in the other direction too, with weak features like "leading A" and "trailing T" featuring mainly in W (A or T) > S (G or C) contexts – as if the less existing structure, the less the danger of being over-stabilized. Relatedly, several of the Mediators simply create a CpG – in view of the hyper-mutability and structural sturdiness of CpG

dinucleotides, it seems inevitable that they should explain some of our trends. However, CpGs do not explain the appearance of Mediator As and Ts in the W>S contexts, nor do they account for the bi-directional constraint we observe in ΔMFE and ΔCD in **FIGURES 3 AND 4.** Regardless, in view of the deep connections linking CpG status and all the other Mediators to both mutability and RNA structure, an ensemble approach such as SPI is perhaps the best way of isolating the structural contribution of any given SNV.

Successful identification of structurally disruptive sSNVs in known pathogenic synonymous variants

Over the last decade numerous studies have demonstrated that synonymous variants play essential molecular roles in regulating both mRNA structure and processing, including regulation of protein expression, folding and function [reviewed in 9, 84, 85]. However, the potential for pathogenic synonymous variants that impact RNA folding in human genetic disease is not universally appreciated and this class of genetic variation is widely ignored in the practice of clinical variant interpretation. Current American College of Medical Genetics (**ACMG**) guidelines for the assessment of clinically relevant genetic variants focus primarily on missense, nonsense or canonical splice variants and suggest that synonymous "silent" variants should be classified as likely benign if the nucleotide position is not conserved and they are not implicated by splicing assessment tools [7].

The variant assessment community has numerous computational tools to systematically assess pathogenicity of amino acid altering nsSNVs. These algorithms are primarily based upon the high conservation of protein sequences, and as such are not equipped to assess pathogenicity in synonymous variants, which are under different constraints [86]. Given the scarcity of RNA-structure-specific tools that would aid in the simultaneous assessment of both nsSNVs and functional sSNVs in a given patient's genome, we are almost certainly missing novel disease etiologies that have their molecular underpinnings in pathological alterations to mRNA structure.

One of the primary goals of this present study was to address this critical need by creating metrics to enable systematic assessment of all sSNVs in a given subject's genome. While our structural metrics and SPI are not the first attempt to quantify pathogenicity due to mRNA-structural distortion, current methods are limited in their application for genome-wide variant assessment. For example, the RNAsnp Web Server predicts the change in optimal mRNA structure and base-pairing probabilities due to a SNV [77], and the command line tool remuRNA

calculates the relative entropy between the mutant and wildtype mRNA structural ensembles [87]. However, while these tools predict disruptions to mRNA structure, they do not attempt to predict pathogenicity and must be executed manually on each variant of interest.

Both RNAsnp and remuRNA were recently utilized to create a database of synonymous mutations in cancer (SynMICdb), using data from COSMIC across 88 tumor types [71]. For constitutional genetic disease, a related resource is the database of Deleterious Synonymous (dbDSM), which manually curates sSNVs reported to be pathogenic in the literature and in databases like ClinVar [88]. These resources represent an important step towards evaluating sSNVs in disease. However, outside of those synonymous variants known to impact splicing, relatively few sSNVs have well supported evidence of their pathogenicity. As such, to evaluate our metrics, we focused on a set of nine sSNVs that we believe the authors unequivocally demonstrated to be pathogenic through their effects on mRNA structure (**TABLE 2**). This dataset included one variant in OPTC associated with glaucoma [62], two variants in NKX2-5 associated with congenital heart defects [64], one variant in DRD2 associated with post-traumatic stress disorder [60], two variants in COMT associated with pain sensitivity [61], one variant in F2 (prothrombin) associated with thrombosis [89], and two variants linked to cancer in KRAS [71] and TP53 [90].

All nines sSNVs demonstrated definite enrichment for our structural metrics, by stability, edge distance, diversity or SPI, with the summary metric, SURF, having values in the 90th percentile range for all nine sSNVs. For example, the synonymous variant in *F2* (NM_000506.4:c.1824C>T;p.Arg608=) had a SURF score in the 97th percentile (driven by a high MFEED value), indicating that the variant introduced a high number of base-pair changes in the *F2* mRNA. Moreover, the negative ΔMFE and ΔCD values we report for this variant indicate that it results in a more stable mRNA with reduced diversity in the structural ensemble. This fits with the observations of Pruner *et al.*, as they demonstrated the variant increased *F2* mRNA levels, carriers of the variant had increased concentrations of F2 in plasma, and the frequency of the variant was significantly higher in patients with venous thromboembolism and cerebrovascular insult.

Notably, none of these clinically relevant sSNVs qualifies as a truly exceptional outlier for any of our ViennaRNA metrics or SPI with all percentiles being below 99. It is plausible that such extreme outliers are not biologically tenable, making them less likely to appear in the human population. Another possibility is that these

sSNVs occupy important regulatory positions, and that a sSNV deleterious to mRNA secondary structure may exhibit pathogenicity when it distorts structure *in a key region* of the transcript. At any rate, the moderateness of our structural metrics in putative SNVs indicate that a 70th-percentile cutoff (Phred value >= 5) for pathogenicity would be reasonable.

Molecular mechanisms underlying constraint of sSNVs

Synonymous variants that impact mRNA secondary structure could confer pathogenicity in numerous ways. Foremost of these mechanisms is that an unstable RNA has a shorter functional half-life and so produces less overall protein [20, 22, 24]. RNA structure modulates the movement of the ribosome along the mRNA molecule, dictating the length of pauses in ribosomal elongation and translocation, both critical for appropriate protein folding and ensuring a safe distance is maintained between adjacent ribosomes [31]. Stronger structures may snap quickly back together after translation, reducing the possible time-window for ribosomal collisions [27], while weaker secondary structures may disappear between ribosomes operating close to one another [91], demonstrating how precisely ribosomal positioning can be regulated through the folding of RNA. Ribosomal collisions essentially end the RNA's life, activating the no-go decay (NGD) pathway, and are also known to cause frame-shifts [26, 30, 91, 92]. In support of all these hypotheses, we note that the majority of our observed constraint is to preserve stability.

Another potential consequence of RNA misfolding is that a more stable mRNA may not be able to initiate translation, also resulting in lower protein levels [16, 18, 29, 39]. Nearly all species exhibit a reduction in mRNA stability near the start codon, however, for mammals and birds this trend in mainly seen in GC rich genes [17]. Some studies suggest that by making the mRNA structure too difficult, or too easy, for the ribosome to process, synonymous codons can act to promote or frustrate proper protein folding [49]. RNA stability limits the growth-rate of the peptide chain and thereby provides time for the core of the protein to establish itself [93, 94]. These findings emphasize the centrality of mRNA structure in regulation of ribosomal speed.

sSNVs also play roles in other processes that could impact our observations. While the stability of an mRNA transcript can determine how quickly it is translated [19, 29, 38], protein synthesis is regulated by both the abundance [95] and recruitment of tRNAs through synonymous codon utilization (codon bias) [96-98]. However,

there are two reasons we expect codon optimality to be a secondary factor in our study. First, we do not observe a depletion in mutations from optimal to sub-optimal codons (see **SUPPLEMENTARY FIGURE 8**). Second, the optimal reference codons tend to be those ending in G or C, so our REF>ALT contexts should largely account for changes in codon optimality. This assumption is consistent with an earlier study that clearly separated the two factors' contributions to gene expression [27]. Yet it is worth remarking that optimal to sub-optimal mutations (i.e. G/C to A/T) do show sharper constraint throughout our work. Regardless, to give proper weight to tRNA we include the tRNA Adaptivity Index (**tAI**), a measure of codon optimality, in our null model for SPI [99]. Our understanding of the role of bicodon bias in human disease is limited, yet pairing of consecutive codons is another mechanisms by which the translational process is regulated [12, 47].

Finally, it is important to consider the essential role of synonymous codons in RNA splicing. While we took care to exclude sSNVs impacting the canonical splice sites from our constraint analysis, exonic variants beyond the canonical splice site can disrupt splice enhancers [100], or they may also activate cryptic splice sites, leading to loss of coding sequence [101]. Given the diversity of molecular roles that synonymous codons have, it will be important for future studies to create scores that would allow assessment of sSNV pathogenicity through any these possible mechanisms.

POTENTIAL IMPLICATIONS

We have shown that sSNVs which disrupt mRNA structure are significantly constrained in the human population, thereby supporting a growing understanding that previously assumed "silent" polymorphisms actually play important roles in regulation of gene expression and protein function. We have demonstrated that this connection is rich, complex, and biologically intuitive. Given that there are multiple mechanisms by which sSNVs influence biological function, we are almost certainly missing undiscovered disease etiologies when these variants are ignored.

In addition to providing the community with a dataset of ten ViennaRNA structural metrics for every known variant, our Structural Predictivity Index (SPI) represents a comprehensive method for predicting possible pathogenicity due specifically to changes in mRNA secondary structure. Because no single metric is capable of

capturing all aspects by which a variant can alter structure, our summary metric SURF provides a single measurement to predict the impact of mRNA-structural variables in human genetic studies. We hope that these metrics will be utilized to accurately assess and prioritize an underrepresented class of genetic variation that may be playing significant and as yet to be realized role in human health and disease.

METHODS

RNA structure prediction process

Global assessment of sSNVs is truly a big data problem as it requires generation and evaluation of several raw values for each of hundreds of millions of positions within the genome. To address this challenge and successfully predict the mRNA-structural effects of every possible sSNV, we developed novel software built upon the Apache Spark framework (**FIGURE 2**). Apache Spark is a distributed, open source compute engine that drastically reduces the bottleneck of disk I/O by processing its data in memory whenever possible [102]. This leads to a 100x increase in speed and allows for more flexible software design than can be achieved in the traditional Hadoop MapReduce paradigm. Spark is well suited to address many of the challenges faced in analyzing big genomics data in a highly scalable manner and adoption is growing steadily, with applications such as SparkSeq [103] for general processing, SparkBWA [104] for alignment and VariantSpark for variant clustering [105]. By developing a solution within this framework, we eliminate significant computational hurdles standing in the way of large-scale analysis of sSNVs.

We used the NCBI RefSeq database (Release 81, GRCh38) as the source for all known human coding transcript sequences. At each position within a given transcript, four 101-base sequence windows were built, differing only in their central nucleotide, which was set to the reference nucleotide or one of the three possible alternate bases. If the nucleotide lay within 50 bases of the transcript boundary, the window was simply taken to be the leading/trailing 101 nucleotides of the transcript. Using Apache Spark in the Amazon Web Services (**AWS**) Elastic Map Reduce (**EMR**) service, we developed a massively parallel implementation of the ViennaRNA Package to analyze the four possible sequences. ViennaRNA is a secondary structure prediction package that has been

extensively utilized and continuously developed for nearly twenty-five years, and uses the standard partition-function paradigm of RNA structural prediction [106].

Our Spark implementation of Vienna enabled us to examine changes in mRNA folding that result from any given polymorphism, and thereby obtain ten metrics which quantified the SNV's effect on mRNA secondary structure (see **Supplementary Data Table 1**). First, we utilized RNAfold to obtain predicted free energies for both mutant and wildtype sequences, which we compared directly to obtain four metrics describing the sSNV's effect on mRNA stability (ΔMFE, ΔCFE, ΔEFE and ΔMEAFE). Next, we fed the predicted structures from RNAfold into the ViennaRNA programs RNApdist and RNAdistance to obtain 6 additional metrics quantifying the change in base-pairing (CED, MFEED, EED, MEAD) and ensemble diversity (ΔCD, ΔEND) due to each SNV. (See the documentation of [14] for detailed descriptions of these concepts). We performed this procedure for all 470 million possible SNVs in 45,800 transcripts. After building our fasta files, we were able to run the whole computation in less than 24 hours using 51 c4.8xlarge AWS EMR computing nodes.

Classification of variants

A common difficulty in variant classification is that a SNV may have different effects in different transcripts. To address this challenge, we annotated every SNV using the program snpEff [107], whose source code was modified to allow record-by-record calling via Spark. This snpEff analysis produced multiple annotations including the effect and location of the variant, e.g. missense, synonymous, canonical splice site, etc. To validate these snpEff predictions we also manually predicted the coding effect each SNV using start and stop codon information from RefSeq (ftp://ftp.ncbi.nih.gov/refseq/H_sapiens/RefSeqGene/refseqgene.*.genomic.gbff.gz). The small number of sSNVs where our predicted biotype disagreed with snpEff's were discarded. After computing variant effect and location, we assigned each SNV a classification based on the most deleterious role it played in any transcript. In decreasing order of deleteriousness, these roles were: start loss, stop gain, start gain, stop loss, missense, synonymous, 5 prime UTR, 3 prime UTR.

Having completed the annotation process we had a total of 470,606,772 SNVs in all known transcripts. As exonic locations can share the same genomic coordinates for multiple transcripts, we next collapsed the data to

184,810,596 unique chromosome positions, assigning each variant assigned a canonical transcript. Canonical transcripts were selected by (1) representation in the MANE database (v0.9); or if the given gene was not in MANE, we chose either (2) the transcript with the longest CDS or when CDS length was the same across multiple transcripts for a given gene (3) the longest transcript. After filtering out variants implicated in splicing or lacking annotations needed in future steps, we obtained a dataset of 22.9 million synonymous variants, 70 million missense variants, 73 million variants in the 3′ UTR, and 13 million variants in the 5′ UTR. See **FIGURE 2** for a summary of our computational pipeline and **SUPPLEMENTARY DATA TABLE 5** for a record of the number of SNVs filtered at each stage.

Determination of population minor allele frequencies

To measure constraint operating on a SNV we used population frequencies obtained from the gnomAD database. We combined both the exome variant calls from release v2.1.1 (originally mapped to GRCh37 and lifted over to GRCh38 coordinates by the gnomAD group) and genome sequencing variants calls from v3.1 (mapped and called using GRCh38). Quality filtering was applied using gnomAD recommendations, removing approximately 1 million SNVs that failed random forest filtering (thresholds of 0.055 for gnomAD 2.1.1 exome data) and removing approximately 3,000 SNVs with an inbreeding coefficient < 0.3. Approximately 22,000 were filtered out with a MAF >= 0.5 (indicative of sites where the reference allele represented a minor allele in the population). Finally, since the majority (approximately 90%) of SNVs have a gnomAD frequency 0, it was important to identify SNVs marked zero purely through a lack of coverage. To achieve this, we flagged and removed all sSNVs where fewer than 70% of samples had at least 20X coverage. Approximately 7.6 million SNVs failed these quality and coverage metrics, leaving a core dataset of 21.4 million synonymous variants, 68 million missense variants, 69 million variants in the 3′ UTR, and 12 million variants in the 5′ UTR (**SUPPLEMENTARY DATA TABLE 5**). When combining the gnomAD data from WGS and WES sets, we used only those SNVs that passed all our filters in both sets. A SNV with MAF>0 in only one of the sets was considered to have MAF>0 in the joint set.

Further variant annotations and data partitioning

We estimated the local nucleotide content around each sSNV by dividing each transcript into windows of 40 bases and in each window calculated the proportion of A's, C's, G's, T's, CpG's and AT's in the surrounding three windows; these annotations were used in constructing SPI and identifying Mediator variables. Finally, we joined multiple additional annotations (including conservation metrics such as PhyloP) from the dbNSFP dataset [108]. Again, this heavy task was greatly facilitated by our Spark framework.

We carried out most of the analysis separately on subsets of data defined by a common mRNA reference and alternate allele, e.g those sSNVs of form C>A. The reference and alternate alleles exert such a huge influence on gnomAD frequency that the best solution seemed to be to control for them explicitly. The number of sSNVs in each context and the proportion appearing in gnomAD are given in **SUPPLEMENTARY TABLE 3**.

Identification of significant contexts

TABLE 1, which describes the correlation between our structural metrics and gnomAD frequency in each REF>ALT context, is an abbreviated version of the more complete description given in **SUPPLEMENTARY TABLE 2**. In each context we ran linear and quadratic regressions between our structural metric and the value P(MAF>0) at each value of the metric, weighted by the number of sSNVs for which the metric attained that value. An asterisk (*) denotes that quadratic R^2 and p-values are reported instead of linear; this was done if quadratic pseudo-R^2 exceeded the linear by a factor of at least five. The normalized slope was computed by dividing the slope of the regression line by the average P(MAF>0) in the context and then multiplying by the range covered by the metric in its central 90% of sSNVs. The "Constrained Against" field simply states whether the normalized slope (or the quadratic coefficient, in quadratic cases) is positive or negative.

Mediator variables

Mediator variables (so called because they explain some of the connection between our mRNA structural metrics and gnomAD frequency) are given in **TABLE 1**. They were chosen to be the sequence feature that explained the greatest portion of the connection between a structural metric (e.g. ΔMFE) and the proportion of nucleotides

with MAF>0 in a context. Possible Mediator variables we considered were local nucleotide content and the specific nucleotides up/downstream of the sSNV.

To compute the proportion of correlation between a structural metric (e.g. ΔMFE) and MAF that is explained by a sequence feature such as CpG content in a particular REF-ALT context, we first built a simple logistic regression model to estimate the quantity $\mathbf{P}(\mathrm{MAF} > 0 \mid \mathrm{CpG\ content})$. We then plug the resulting estimate $\mathbf{P}_{\mathbf{estimated}}(\mathrm{MAF} > 0 \mid \mathrm{CpG\ content})$ into the expression

$$V_{\mathrm{CpG\ content}} = \sum_{x} \mathbf{n_x} \times \left(\mathbf{E}\left(\mathbf{P}_{\mathbf{estimated}}(\mathrm{MAF} > 0 \mid \mathrm{CpG\ content}) \mid \Delta\mathrm{MFE} = \mathrm{x}\right) - \mathbf{P}(\mathrm{MAF} > 0 \mid \Delta\mathrm{MFE} = \mathrm{x})\right)^2$$

where the sum is over all values of ΔMFE and $\mathbf{n_x}$ is number of sSNVs in the context with ΔMFE= x. Comparing this quantity $V_{\mathrm{CpG\ content}}$ to the null variance

$$V_{\mathrm{null}} = \sum_{x} \mathbf{n_x} \times \left(\mathbf{P}(\mathrm{MAF} > 0) - \mathbf{P}(\mathrm{MAF} > 0 \mid \Delta\mathrm{MFE} = \mathrm{x})\right)^2$$

allows us to compute the proportion of the variation explained by CpG content:

$$R^2_{\mathrm{CpG\ content}} = 1 - \frac{V_{\mathrm{CpG\ content}}}{V_{\mathrm{null}}}$$

The “Mediator” for a given structural metric in a given context is chosen as the variable with the highest R^2. Finally, the correlation between the Mediator and the event that MAF>0 was checked, and the Mediator given a sign (+/-) so that it correlated positively with MAF>0-.

Construction of SPI

To construct SPI scores we built two separate models over each of our 14 contexts to predict the event MAF > 0. The “null” model used multiple natural features - the nine nucleotides in the SNV's home and adjacent codons, the proportion of A/C/G/T/CpG/AT's in the surrounding 120 nucleotides, the sSNV's position in its codon, its transcript and the transcript's length, and the tAI (tRNA Adapation Index obtained from a supplement of [109] from https://ars.els-cdn.com/content/image/1-s2.0-S0092867410003193-mmc2.xls) of the wildtype and mutant codons. The second, “active” model used all these features plus our 10 ViennaRNA metrics and the binding statuses of the reference and alternate bases in the predicted MFE structures generated by Vienna.

Both sets of variables were then used to predict whether MAF > 0 using a weighted general linear model as implemented in the LogisticRegression module of the python scikit-learn package [110]. We then defined the SPI score for a sSNV to be the base-10 logarithm of the active model's predicted P(MAF>0) divided by the null model's predicted P(MAF>0). Context-wise plots for SPI are given in the **SUPPLEMENTARY DATA FIGURE 6**.

We trained our SPIs using a five-fold cross-validation in each SNV context, with the final assigned prediction being the average of all five predicted probabilities for a variant. When training SPI we used six separate schemes for partitioning the gnomAD data: WGS only, WES only, their union but throw away SNVs present in only one dataset; the union but count such SNVs as having MAF>0; and analogously for intersections. Then in each SNV context we use the SPI score that yields the highest AUC. We also tried three different model-styles for computing the raw predictions that comprise SPI – general logistic as implemented in python's sklearn LogisticRegression module, random forest as implemented in sklearn's RandomForestClassifier and gradient-boosted trees as implemented in the extreme gradient boosting python package XGBoost [111]. Performance of each SPI "flavor" is given in **SUPPLEMENTARY DATA TABLE 4**. We settled on the general logistic model, due to its simplicity, and also due to the generally poor performance of the two tree-based models. SPI scores were Z-score normalized (subtracted the mean and divided by the s.d.) and percentile ranked within each context. Finally, these context-specific percentile rankings were converted to a Phred-scaled score ($-10 \times log_{10}(1 - \text{SPI Context Percentile})$) prior to building **FIGURE 6.**

Construction of SURF

To construct our final **SU**mmarized **R**NA **F**olding (**SURF**) metrics (**FIGURE 7)**, each of the ten RNA folding metrics were percentile ranked and Phred-scaled, such that the larger the Phred-scaled value the greater the precited change in RNA structure. For scores measuring a delta in the given metric, negative stability and diversity values were ranked separately from positive values, using the formula $-10 \times log_{10}(1 - \text{Percentile Rank})$. For edge distance, positive stability and positive diversity metrics results were Phred-scaled using the formula $-10 \times log_{10}(1 - \text{Percentile Rank})$. Finally, any Phred score >50 (i.e. a metric in the 99.999th percentile or above) was set to a value of 50, resulting in all Phred-scaled scores ranging from 0 to 50. For each SNV in our dataset, maximum Phred score

was determined across the four stability metrics (ΔMFE, ΔCFE, ΔMEAFE and ΔEFE) to generate the SURF Stability score, across the four edge distance metrics (CED, MFEED, EED and MEAED) to generate SURF Edit Distance score, or across the two diversity metrics (ΔCD and ΔEND) to generate the SURF Diversity score. Finally, the single summary metric, SURF, was generated by choosing the maximum Phred score across any of the 10 RNA stability metrics and SPI.

AVAILABILITY OF SOURCE CODE AND REQUIREMENTS

Project name: rna-stability

Project home page: https://github.com/nch-igm/rna-stability

Operating system: Linux

Programming language: Scala

Other requirements: Apache Spark 2.4+

License: FreeBSD

AVAILABILITY OF SUPPORTING DATA AND MATERIALS

The software we developed and structural scores are available on GitHub: https://github.com/nch-igm/rna-stability and are available via the *GigaScience* database GigaDB [112]. The software is registered with bio.tools (https://bio.tools/rna-stability) and in the ScriCrunh.org database under resource ID SCR_019259 [113].

ADDITIONAL FILES

A single supplementary data file (RNA_stability_supplementary_data.pdf) is available at *GigaScience* online and contains the following tables and figures:

Supplementary Data Table 1 - Vienna RNA Metrics

Supplementary Data Table 2 - Constraint Across Sequence Contexts

Supplementary Data Table 3 - sSNV Contexts Across the Human Transcriptome

Supplementary Data Table 4 - Modelling Structural Constraint with SPI Score

Supplementary Data Table 5 - Data Pre-Processing Steps

Supplementary Data Figure 1 - Distribution of Structural Metrics

Supplementary Data Figure 2 - Calculation of Edit Distance

Supplementary Data Figure 3 - Structural Metrics over All Synonymous SNVs

Supplementary Data Figure 4 - Structural Metrics in Contexts Constrained Against Destabilization

Supplementary Data Figure 5 - Structural Metrics in Contexts Constrained Against Over-Stabilization

Supplementary Data Figure 6 - Sequence Context and SPI

Supplementary Data Figure 7 - Structural Metrics vs. log(MAF)

Supplementary Data Figure 8 - Change in Codon Optimality vs. Mutation Rate

DECLARATIONS

Abbreviations

CED: Centroid Edit Distance; ΔCD: delta Centroid Distance; ΔMFE: delta Minimum Free Energy; gnomAD: Genome Aggregation Database; MAF: minor allele frequency; mRNA: messenger RNA; nsSNVs: non-synonymous single-nucleotide variants; SNP: single nucleotide variant; SNV: single nucleotide variant; SPI: Structural Predictivity Index; sSNVs: synonymous single-nucleotide variants

Competing interests

The authors declare no competing interests.

Funding

We thank the Nationwide Children's Foundation and The Abigail Wexner Research Institute at Nationwide Children's Hospital for generously supporting this body of work. James L. Li was supported by the Pelotonia Fellowship for Undergraduate Research through The Ohio State University Comprehensive Cancer Society. These funding bodies had no role in the design of the study, no role in the collection, analysis, and interpretation of data and no role in writing the manuscript.

Authors' contributions

J.B.S.G., J.L.L and P.W. developed methodology, performed data analysis and results interpretation. G.E.L. developed AWS Spark ViennaRNA pipeline and developed variant annotation tools. G.E.L. generated folding metrics. J.B.S.G. developed Structural Predictivity Index (SPI). D.M.G., H.C.K., B.J.K, and J.R.F assisted with data analysis, interpretation of results and development of variant annotation tools. J.B.S.G, G.E.L and P.W. prepared figures. All authors contributed to the preparation and editing of the final manuscript.

Acknowledgements

This team works in the Steve and Cindy Rasmussen Institute for Genomic Medicine at Nationwide Children's Hospital. The Institute is generously supported by the Nationwide Foundation Pediatric Innovation Fund.

REFERENCES

1. Wright CF, FitzPatrick DR and Firth HV. Paediatric genomics: diagnosing rare disease in children. Nat Rev Genet. 2018;19 5:253-68. doi:10.1038/nrg.2017.116.
2. Yang Y, Muzny DM, Reid JG, Bainbridge MN, Willis A, Ward PA, et al. Clinical whole-exome sequencing for the diagnosis of mendelian disorders. N Engl J Med. 2013;369 16:1502-11. doi:10.1056/NEJMoa1306555.
3. Yang Y, Muzny DM, Xia F, Niu Z, Person R, Ding Y, et al. Molecular findings among patients referred for clinical whole-exome sequencing. JAMA. 2014;312 18:1870-9. doi:10.1001/jama.2014.14601.
4. Ellingford JM, Barton S, Bhaskar S, Williams SG, Sergouniotis PI, O'Sullivan J, et al. Whole Genome Sequencing Increases Molecular Diagnostic Yield Compared with Current Diagnostic Testing for Inherited Retinal Disease. Ophthalmology. 2016;123 5:1143-50. doi:10.1016/j.ophtha.2016.01.009.

5. Hegde M, Santani A, Mao R, Ferreira-Gonzalez A, Weck KE and Voelkerding KV. Development and Validation of Clinical Whole-Exome and Whole-Genome Sequencing for Detection of Germline Variants in Inherited Disease. Arch Pathol Lab Med. 2017;141 6:798-805. doi:10.5858/arpa.2016-0622-RA.
6. Worthey EA. Analysis and Annotation of Whole-Genome or Whole-Exome Sequencing Derived Variants for Clinical Diagnosis. Curr Protoc Hum Genet. 2017;95:9 24 1-9 8. doi:10.1002/cphg.49.
7. Richards S, Aziz N, Bale S, Bick D, Das S, Gastier-Foster J, et al. Standards and guidelines for the interpretation of sequence variants: a joint consensus recommendation of the American College of Medical Genetics and Genomics and the Association for Molecular Pathology. Genet Med. 2015;17 5:405-24. doi:10.1038/gim.2015.30.
8. Alfares A, Aloraini T, Subaie LA, Alissa A, Qudsi AA, Alahmad A, et al. Whole-genome sequencing offers additional but limited clinical utility compared with reanalysis of whole-exome sequencing. Genet Med. 2018;20 11:1328-33. doi:10.1038/gim.2018.41.
9. Fahraeus R, Marin M and Olivares-Illana V. Whisper mutations: cryptic messages within the genetic code. Oncogene. 2016;35 29:3753-9. doi:10.1038/onc.2015.454.
10. Lee M, Roos P, Sharma N, Atalar M, Evans TA, Pellicore MJ, et al. Systematic Computational Identification of Variants That Activate Exonic and Intronic Cryptic Splice Sites. Am J Hum Genet. 2017;100 5:751-65. doi:10.1016/j.ajhg.2017.04.001.
11. Ramanouskaya TV and Grinev VV. The determinants of alternative RNA splicing in human cells. Mol Genet Genomics. 2017;292 6:1175-95. doi:10.1007/s00438-017-1350-0.
12. Hanson G and Coller J. Codon optimality, bias and usage in translation and mRNA decay. Nat Rev Mol Cell Biol. 2018;19 1:20-30. doi:10.1038/nrm.2017.91.
13. Silverman SK. A forced march across an RNA folding landscape. Chem Biol. 2008;15 3:211-3. doi:10.1016/j.chembiol.2008.02.014.
14. Lorenz R, Bernhart SH, Honer Zu Siederdissen C, Tafer H, Flamm C, Stadler PF, et al. ViennaRNA Package 2.0. Algorithms Mol Biol. 2011;6:26. doi:10.1186/1748-7188-6-26.

15. Yakovchuk P, Protozanova E and Frank-Kamenetskii MD. Base-stacking and base-pairing contributions into thermal stability of the DNA double helix. Nucleic Acids Res. 2006;34 2:564-74. doi:10.1093/nar/gkj454.

16. Chamary JV and Hurst LD. Evidence for selection on synonymous mutations affecting stability of mRNA secondary structure in mammals. Genome Biol. 2005;6 9:R75. doi:10.1186/gb-2005-6-9-r75.

17. Gu W, Zhou T and Wilke CO. A universal trend of reduced mRNA stability near the translation-initiation site in prokaryotes and eukaryotes. PLoS Comput Biol. 2010;6 2:e1000664. doi:10.1371/journal.pcbi.1000664.

18. Katz L and Burge CB. Widespread selection for local RNA secondary structure in coding regions of bacterial genes. Genome Res. 2003;13 9:2042-51. doi:10.1101/gr.1257503.

19. Seffens W and Digby D. mRNAs have greater negative folding free energies than shuffled or codon choice randomized sequences. Nucleic Acids Res. 1999;27 7:1578-84. doi:10.1093/nar/27.7.1578.

20. Duan J and Antezana MA. Mammalian mutation pressure, synonymous codon choice, and mRNA degradation. J Mol Evol. 2003;57 6:694-701. doi:10.1007/s00239-003-2519-1.

21. Wan Y, Qu K, Ouyang Z, Kertesz M, Li J, Tibshirani R, et al. Genome-wide measurement of RNA folding energies. Mol Cell. 2012;48 2:169-81. doi:10.1016/j.molcel.2012.08.008.

22. Lazrak A, Fu L, Bali V, Bartoszewski R, Rab A, Havasi V, et al. The silent codon change I507-ATC->ATT contributes to the severity of the DeltaF508 CFTR channel dysfunction. FASEB J. 2013;27 11:4630-45. doi:10.1096/fj.13-227330.

23. Hunt RC, Simhadri VL, Iandoli M, Sauna ZE and Kimchi-Sarfaty C. Exposing synonymous mutations. Trends Genet. 2014;30 7:308-21. doi:10.1016/j.tig.2014.04.006.

24. Shah K, Cheng Y, Hahn B, Bridges R, Bradbury NA and Mueller DM. Synonymous codon usage affects the expression of wild type and F508del CFTR. J Mol Biol. 2015;427 6 Pt B:1464-79. doi:10.1016/j.jmb.2015.02.003.

25. Bevilacqua PC, Ritchey LE, Su Z and Assmann SM. Genome-Wide Analysis of RNA Secondary Structure. Annu Rev Genet. 2016;50:235-66. doi:10.1146/annurev-genet-120215-035034.

26. D'Orazio KN, Wu CC, Sinha N, Loll-Krippleber R, Brown GW and Green R. The endonuclease Cue2 cleaves mRNAs at stalled ribosomes during No Go Decay. Elife. 2019;8 doi:10.7554/eLife.49117.

27. Mauger DM, Cabral BJ, Presnyak V, Su SV, Reid DW, Goodman B, et al. mRNA structure regulates protein expression through changes in functional half-life. Proc Natl Acad Sci U S A. 2019;116 48:24075-83. doi:10.1073/pnas.1908052116.

28. Mustoe AM, Busan S, Rice GM, Hajdin CE, Peterson BK, Ruda VM, et al. Pervasive Regulatory Functions of mRNA Structure Revealed by High-Resolution SHAPE Probing. Cell. 2018;173 1:181-95 e18. doi:10.1016/j.cell.2018.02.034.

29. Presnyak V, Alhusaini N, Chen YH, Martin S, Morris N, Kline N, et al. Codon optimality is a major determinant of mRNA stability. Cell. 2015;160 6:1111-24. doi:10.1016/j.cell.2015.02.029.

30. Simms CL, Yan LL and Zaher HS. Ribosome Collision Is Critical for Quality Control during No-Go Decay. Mol Cell. 2017;68 2:361-73 e5. doi:10.1016/j.molcel.2017.08.019.

31. Wen JD, Lancaster L, Hodges C, Zeri AC, Yoshimura SH, Noller HF, et al. Following translation by single ribosomes one codon at a time. Nature. 2008;452 7187:598-603. doi:10.1038/nature06716.

32. Babendure JR, Babendure JL, Ding JH and Tsien RY. Control of mammalian translation by mRNA structure near caps. RNA. 2006;12 5:851-61. doi:10.1261/rna.2309906.

33. Ding Y, Tang Y, Kwok CK, Zhang Y, Bevilacqua PC and Assmann SM. In vivo genome-wide profiling of RNA secondary structure reveals novel regulatory features. Nature. 2014;505 7485:696-700. doi:10.1038/nature12756.

34. Dvir S, Velten L, Sharon E, Zeevi D, Carey LB, Weinberger A, et al. Deciphering the rules by which 5'-UTR sequences affect protein expression in yeast. Proc Natl Acad Sci U S A. 2013;110 30:E2792-801. doi:10.1073/pnas.1222534110.

35. Keller TE, Mis SD, Jia KE and Wilke CO. Reduced mRNA secondary-structure stability near the start codon indicates functional genes in prokaryotes. Genome Biol Evol. 2012;4 2:80-8. doi:10.1093/gbe/evr129.

36. Kertesz M, Wan Y, Mazor E, Rinn JL, Nutter RC, Chang HY, et al. Genome-wide measurement of RNA secondary structure in yeast. Nature. 2010;467 7311:103-7. doi:10.1038/nature09322.

37. Zhou T and Wilke CO. Reduced stability of mRNA secondary structure near the translation-initiation site in dsDNA viruses. BMC Evol Biol. 2011;11:59. doi:10.1186/1471-2148-11-59.

38. Yang JR, Chen X and Zhang J. Codon-by-codon modulation of translational speed and accuracy via mRNA folding. PLoS Biol. 2014;12 7:e1001910. doi:10.1371/journal.pbio.1001910.

39. Bazzini AA, Del Viso F, Moreno-Mateos MA, Johnstone TG, Vejnar CE, Qin Y, et al. Codon identity regulates mRNA stability and translation efficiency during the maternal-to-zygotic transition. EMBO J. 2016;35 19:2087-103. doi:10.15252/embj.201694699.

40. Fernandez M, Kumagai Y, Standley DM, Sarai A, Mizuguchi K and Ahmad S. Prediction of dinucleotide-specific RNA-binding sites in proteins. BMC Bioinformatics. 2011;12 Suppl 13:S5. doi:10.1186/1471-2105-12-S13-S5.

41. Brummer A and Hausser J. MicroRNA binding sites in the coding region of mRNAs: extending the repertoire of post-transcriptional gene regulation. Bioessays. 2014;36 6:617-26. doi:10.1002/bies.201300104.

42. Savisaar R and Hurst LD. Both Maintenance and Avoidance of RNA-Binding Protein Interactions Constrain Coding Sequence Evolution. Mol Biol Evol. 2017;34 5:1110-26. doi:10.1093/molbev/msx061.

43. Dominguez D, Freese P, Alexis MS, Su A, Hochman M, Palden T, et al. Sequence, Structure, and Context Preferences of Human RNA Binding Proteins. Mol Cell. 2018;70 5:854-67 e9. doi:10.1016/j.molcel.2018.05.001.

44. Wan Y, Qu K, Zhang QC, Flynn RA, Manor O, Ouyang Z, et al. Landscape and variation of RNA secondary structure across the human transcriptome. Nature. 2014;505 7485:706-9. doi:10.1038/nature12946.

45. Fung KL, Pan J, Ohnuma S, Lund PE, Pixley JN, Kimchi-Sarfaty C, et al. MDR1 synonymous polymorphisms alter transporter specificity and protein stability in a stable epithelial monolayer. Cancer Res. 2014;74 2:598-608. doi:10.1158/0008-5472.CAN-13-2064.

46. Guisez Y, Robbens J, Remaut E and Fiers W. Folding of the MS2 coat protein in Escherichia coli is modulated by translational pauses resulting from mRNA secondary structure and codon usage: a hypothesis. J Theor Biol. 1993;162 2:243-52. doi:10.1006/jtbi.1993.1085.

47. McCarthy C, Carrea A and Diambra L. Bicodon bias can determine the role of synonymous SNPs in human diseases. BMC Genomics. 2017;18 1:227. doi:10.1186/s12864-017-3609-6.

48. Plotkin JB and Kudla G. Synonymous but not the same: the causes and consequences of codon bias. Nat Rev Genet. 2011;12 1:32-42. doi:10.1038/nrg2899.

49. Walsh IM, Bowman MA, Soto Santarriaga IF, Rodriguez A and Clark PL. Synonymous codon substitutions perturb cotranslational protein folding in vivo and impair cell fitness. Proc Natl Acad Sci U S A. 2020;117 7:3528-34. doi:10.1073/pnas.1907126117.

50. Yang JR. Does mRNA structure contain genetic information for regulating co-translational protein folding? Zool Res. 2017;38 1:36-43. doi:10.13918/j.issn.2095-8137.2017.004.

51. Seemann SE, Mirza AH, Hansen C, Bang-Berthelsen CH, Garde C, Christensen-Dalsgaard M, et al. The identification and functional annotation of RNA structures conserved in vertebrates. Genome Res. 2017;27 8:1371-83. doi:10.1101/gr.208652.116.

52. Kirsch R, Seemann SE, Ruzzo WL, Cohen SM, Stadler PF and Gorodkin J. Identification and characterization of novel conserved RNA structures in Drosophila. BMC Genomics. 2018;19 1:899. doi:10.1186/s12864-018-5234-4.

53. Peeri M and Tuller T. High-resolution modeling of the selection on local mRNA folding strength in coding sequences across the tree of life. Genome Biol. 2020;21 1:63. doi:10.1186/s13059-020-01971-y.

54. Yao Z, Weinberg Z and Ruzzo WL. CMfinder--a covariance model based RNA motif finding algorithm. Bioinformatics. 2006;22 4:445-52. doi:10.1093/bioinformatics/btk008.

55. Gruber AR, Findeiss S, Washietl S, Hofacker IL and Stadler PF. RNAz 2.0: improved noncoding RNA detection. Pac Symp Biocomput. 2010:69-79.

56. Yang Y, Li X, Zhao H, Zhan J, Wang J and Zhou Y. Genome-scale characterization of RNA tertiary structures and their functional impact by RNA solvent accessibility prediction. RNA. 2017;23 1:14-22. doi:10.1261/rna.057364.116.

57. Genomes Project C, Auton A, Brooks LD, Durbin RM, Garrison EP, Kang HM, et al. A global reference for human genetic variation. Nature. 2015;526 7571:68-74. doi:10.1038/nature15393.

58. Garst AD, Edwards AL and Batey RT. Riboswitches: structures and mechanisms. Cold Spring Harb Perspect Biol. 2011;3 6 doi:10.1101/cshperspect.a003533.

59. Halvorsen M, Martin JS, Broadaway S and Laederach A. Disease-associated mutations that alter the RNA structural ensemble. PLoS Genet. 2010;6 8:e1001074. doi:10.1371/journal.pgen.1001074.

60. Duan J, Wainwright MS, Comeron JM, Saitou N, Sanders AR, Gelernter J, et al. Synonymous mutations in the human dopamine receptor D2 (DRD2) affect mRNA stability and synthesis of the receptor. Hum Mol Genet. 2003;12 3:205-16. doi:10.1093/hmg/ddg055.

61. Nackley AG, Shabalina SA, Tchivileva IE, Satterfield K, Korchynskyi O, Makarov SS, et al. Human catechol-O-methyltransferase haplotypes modulate protein expression by altering mRNA secondary structure. Science. 2006;314 5807:1930-3. doi:10.1126/science.1131262.

62. Acharya M, Mookherjee S, Bhattacharjee A, Thakur SK, Bandyopadhyay AK, Sen A, et al. Evaluation of the OPTC gene in primary open angle glaucoma: functional significance of a silent change. BMC Mol Biol. 2007;8:21. doi:10.1186/1471-2199-8-21.

63. Bartoszewski RA, Jablonsky M, Bartoszewska S, Stevenson L, Dai Q, Kappes J, et al. A synonymous single nucleotide polymorphism in DeltaF508 CFTR alters the secondary structure of the mRNA and the expression of the mutant protein. J Biol Chem. 2010;285 37:28741-8. doi:10.1074/jbc.M110.154575.

64. Reamon-Buettner SM, Sattlegger E, Ciribilli Y, Inga A, Wessel A and Borlak J. Transcriptional defect of an inherited NKX2-5 haplotype comprising a SNP, a nonsynonymous and a synonymous mutation, associated with human congenital heart disease. PLoS One. 2013;8 12:e83295. doi:10.1371/journal.pone.0083295.

65. Simhadri VL, Hamasaki-Katagiri N, Lin BC, Hunt R, Jha S, Tseng SC, et al. Single synonymous mutation in factor IX alters protein properties and underlies haemophilia B. J Med Genet. 2017;54 5:338-45. doi:10.1136/jmedgenet-2016-104072.

66. Hamasaki-Katagiri N, Lin BC, Simon J, Hunt RC, Schiller T, Russek-Cohen E, et al. The importance of mRNA structure in determining the pathogenicity of synonymous and non-synonymous mutations in haemophilia. Haemophilia. 2017;23 1:e8-e17. doi:10.1111/hae.13107.

67. Gotea V, Gartner JJ, Qutob N, Elnitski L and Samuels Y. The functional relevance of somatic synonymous mutations in melanoma and other cancers. Pigment Cell Melanoma Res. 2015;28 6:673-84. doi:10.1111/pcmr.12413.

68. Supek F, Minana B, Valcarcel J, Gabaldon T and Lehner B. Synonymous mutations frequently act as driver mutations in human cancers. Cell. 2014;156 6:1324-35. doi:10.1016/j.cell.2014.01.051.

69. Zhang D and Xia J. Somatic synonymous mutations in regulatory elements contribute to the genetic aetiology of melanoma. BMC Med Genomics. 2020;13 Suppl 5:43. doi:10.1186/s12920-020-0685-2.

70. Pecce V, Sponziello M, Damante G, Rosignolo F, Durante C, Lamartina L, et al. A synonymous RET substitution enhances the oncogenic effect of an in-cis missense mutation by increasing constitutive splicing efficiency. PLoS Genet. 2018;14 10:e1007678. doi:10.1371/journal.pgen.1007678.

71. Sharma Y, Miladi M, Dukare S, Boulay K, Caudron-Herger M, Gross M, et al. A pan-cancer analysis of synonymous mutations. Nat Commun. 2019;10 1:2569. doi:10.1038/s41467-019-10489-2.

72. Buske OJ, Manickaraj A, Mital S, Ray PN and Brudno M. Identification of deleterious synonymous variants in human genomes. Bioinformatics. 2013;29 15:1843-50. doi:10.1093/bioinformatics/btt308.

73. Livingstone M, Folkman L, Yang Y, Zhang P, Mort M, Cooper DN, et al. Investigating DNA-, RNA-, and protein-based features as a means to discriminate pathogenic synonymous variants. Hum Mutat. 2017;38 10:1336-47. doi:10.1002/humu.23283.

74. Shi F, Yao Y, Bin Y, Zheng CH and Xia J. Computational identification of deleterious synonymous variants in human genomes using a feature-based approach. BMC Med Genomics. 2019;12 Suppl 1:12. doi:10.1186/s12920-018-0455-6.

75. Zhang X, Li M, Lin H, Rao X, Feng W, Yang Y, et al. regSNPs-splicing: a tool for prioritizing synonymous single-nucleotide substitution. Hum Genet. 2017;136 9:1279-89. doi:10.1007/s00439-017-1783-x.

76. Zhang T, Wu Y, Lan Z, Shi Q, Yang Y and Guo J. Syntool: A Novel Region-Based Intolerance Score to Single Nucleotide Substitution for Synonymous Mutations Predictions Based on 123,136 Individuals. Biomed Res Int. 2017;2017:5096208. doi:10.1155/2017/5096208.

77. Sabarinathan R, Tafer H, Seemann SE, Hofacker IL, Stadler PF and Gorodkin J. The RNAsnp web server: predicting SNP effects on local RNA secondary structure. Nucleic Acids Res. 2013;41 Web Server issue:W475-9. doi:10.1093/nar/gkt291.

78. Miladi M, Raden M, Diederichs S and Backofen R. MutaRNA: analysis and visualization of mutation-induced changes in RNA structure. Nucleic Acids Res. 2020;48 W1:W287-W91. doi:10.1093/nar/gkaa331.

79. Lek M, Karczewski KJ, Minikel EV, Samocha KE, Banks E, Fennell T, et al. Analysis of protein-coding genetic variation in 60,706 humans. Nature. 2016;536 7616:285-91. doi:10.1038/nature19057.

80. Gronau I, Arbiza L, Mohammed J and Siepel A. Inference of natural selection from interspersed genomic elements based on polymorphism and divergence. Mol Biol Evol. 2013;30 5:1159-71. doi:10.1093/molbev/mst019.

81. Huang YF, Gulko B and Siepel A. Fast, scalable prediction of deleterious noncoding variants from functional and population genomic data. Nat Genet. 2017;49 4:618-24. doi:10.1038/ng.3810.

82. Li E and Zhang Y. DNA methylation in mammals. Cold Spring Harb Perspect Biol. 2014;6 5:a019133. doi:10.1101/cshperspect.a019133.

83. Turner DH and Mathews DH. NNDB: the nearest neighbor parameter database for predicting stability of nucleic acid secondary structure. Nucleic Acids Res. 2010;38 Database issue:D280-2. doi:10.1093/nar/gkp892.

84. Sauna ZE and Kimchi-Sarfaty C. Understanding the contribution of synonymous mutations to human disease. Nat Rev Genet. 2011;12 10:683-91. doi:10.1038/nrg3051.

85. Shabalina SA, Spiridonov NA and Kashina A. Sounds of silence: synonymous nucleotides as a key to biological regulation and complexity. Nucleic Acids Res. 2013;41 4:2073-94. doi:10.1093/nar/gks1205.

86. Gelfman S, Wang Q, McSweeney KM, Ren Z, La Carpia F, Halvorsen M, et al. Annotating pathogenic non-coding variants in genic regions. Nat Commun. 2017;8 1:236. doi:10.1038/s41467-017-00141-2.

87. Salari R, Kimchi-Sarfaty C, Gottesman MM and Przytycka TM. Sensitive measurement of single-nucleotide polymorphism-induced changes of RNA conformation: application to disease studies. Nucleic Acids Res. 2013;41 1:44-53. doi:10.1093/nar/gks1009.

88. Wen P, Xiao P and Xia J. dbDSM: a manually curated database for deleterious synonymous mutations. Bioinformatics. 2016;32 12:1914-6. doi:10.1093/bioinformatics/btw086.

89. Pruner I, Farm M, Tomic B, Gvozdenov M, Kovac M, Miljic P, et al. The Silence Speaks, but We Do Not Listen: Synonymous c.1824C>T Gene Variant in the Last Exon of the Prothrombin Gene as a New Prothrombotic Risk Factor. Clin Chem. 2020;66 2:379-89. doi:10.1093/clinchem/hvz015.

90. Karakostis K, Vadivel Gnanasundram S, Lopez I, Thermou A, Wang L, Nylander K, et al. A single synonymous mutation determines the phosphorylation and stability of the nascent protein. J Mol Cell Biol. 2019;11 3:187-99. doi:10.1093/jmcb/mjy049.

91. Mao Y, Liu H, Liu Y and Tao S. Deciphering the rules by which dynamics of mRNA secondary structure affect translation efficiency in Saccharomyces cerevisiae. Nucleic Acids Res. 2014;42 8:4813-22. doi:10.1093/nar/gku159.

92. Simms CL, Yan LL, Qiu JK and Zaher HS. Ribosome Collisions Result in +1 Frameshifting in the Absence of No-Go Decay. Cell Rep. 2019;28 7:1679-89 e4. doi:10.1016/j.celrep.2019.07.046.

93. Faure G, Ogurtsov AY, Shabalina SA and Koonin EV. Role of mRNA structure in the control of protein folding. Nucleic Acids Res. 2016;44 22:10898-911. doi:10.1093/nar/gkw671.

94. Faure G, Ogurtsov AY, Shabalina SA and Koonin EV. Adaptation of mRNA structure to control protein folding. RNA Biol. 2017;14 12:1649-54. doi:10.1080/15476286.2017.1349047.

95. Dong H, Nilsson L and Kurland CG. Co-variation of tRNA abundance and codon usage in Escherichia coli at different growth rates. J Mol Biol. 1996;260 5:649-63. doi:10.1006/jmbi.1996.0428.

96. Sabi R and Tuller T. Modelling the efficiency of codon-tRNA interactions based on codon usage bias. DNA Res. 2014;21 5:511-26. doi:10.1093/dnares/dsu017.

97. Quax TE, Claassens NJ, Soll D and van der Oost J. Codon Bias as a Means to Fine-Tune Gene Expression. Mol Cell. 2015;59 2:149-61. doi:10.1016/j.molcel.2015.05.035.

98. Rocha EP. Codon usage bias from tRNA's point of view: redundancy, specialization, and efficient decoding for translation optimization. Genome Res. 2004;14 11:2279-86. doi:10.1101/gr.2896904.

99. dos Reis M, Savva R and Wernisch L. Solving the riddle of codon usage preferences: a test for translational selection. Nucleic Acids Res. 2004;32 17:5036-44. doi:10.1093/nar/gkh834.

100. Soukarieh O, Gaildrat P, Hamieh M, Drouet A, Baert-Desurmont S, Frebourg T, et al. Exonic Splicing Mutations Are More Prevalent than Currently Estimated and Can Be Predicted by Using In Silico Tools. PLoS Genet. 2016;12 1:e1005756. doi:10.1371/journal.pgen.1005756.

101. Molinski SV, Gonska T, Huan LJ, Baskin B, Janahi IA, Ray PN, et al. Genetic, cell biological, and clinical interrogation of the CFTR mutation c.3700 A>G (p.Ile1234Val) informs strategies for future medical intervention. Genet Med. 2014;16 8:625-32. doi:10.1038/gim.2014.4.

102. Zaharia M, Chowdhury M, Das T, Dave A, Ma J, McCauley M, et al. Resilient distributed datasets: a fault-tolerant abstraction for in-memory cluster computing. Proceedings of the 9th USENIX conference on Networked Systems Design and Implementation. San Jose, CA: USENIX Association, 2012, p. 2-.

103. Wiewiorka MS, Messina A, Pacholewska A, Maffioletti S, Gawrysiak P and Okoniewski MJ. SparkSeq: fast, scalable and cloud-ready tool for the interactive genomic data analysis with nucleotide precision. Bioinformatics. 2014;30 18:2652-3. doi:10.1093/bioinformatics/btu343.

104. Abuin JM, Pichel JC, Pena TF and Amigo J. SparkBWA: Speeding Up the Alignment of High-Throughput DNA Sequencing Data. PLoS One. 2016;11 5:e0155461. doi:10.1371/journal.pone.0155461.

105. O'Brien AR, Saunders NF, Guo Y, Buske FA, Scott RJ and Bauer DC. VariantSpark: population scale clustering of genotype information. BMC Genomics. 2015;16:1052. doi:10.1186/s12864-015-2269-7.

106. McCaskill JS. The equilibrium partition function and base pair binding probabilities for RNA secondary structure. Biopolymers. 1990;29 6-7:1105-19. doi:10.1002/bip.360290621.

107. Cingolani P, Platts A, Wang le L, Coon M, Nguyen T, Wang L, et al. A program for annotating and predicting the effects of single nucleotide polymorphisms, SnpEff: SNPs in the genome of Drosophila melanogaster strain w1118; iso-2; iso-3. Fly (Austin). 2012;6 2:80-92. doi:10.4161/fly.19695.

108. Liu X, Wu C, Li C and Boerwinkle E. dbNSFP v3.0: A One-Stop Database of Functional Predictions and Annotations for Human Nonsynonymous and Splice-Site SNVs. Hum Mutat. 2016;37 3:235-41. doi:10.1002/humu.22932.

109. Tuller T, Carmi A, Vestsigian K, Navon S, Dorfan Y, Zaborske J, et al. An evolutionarily conserved mechanism for controlling the efficiency of protein translation. Cell. 2010;141 2:344-54. doi:10.1016/j.cell.2010.03.031.

110. Pedregosa F, Varoquaux G, Gramfort A, Michel V, Thirion B, Grisel O, et al. Scikit-learn: Machine Learning in Python. Journal of Machine Learning Research. 2011;12:2825-30.

111. Chen TQ and Guestrin C. XGBoost: A Scalable Tree Boosting System. Kdd'16: Proceedings of the 22nd Acm Sigkdd International Conference on Knowledge Discovery and Data Mining. 2016:785-94. doi:10.1145/2939672.2939785.

112. Gaither JBS, Lammi GE, Li JL, Gordon DM, Kuck HC, Kelly BJ, et al. Supporting data for "Synonymous Variants that Disrupt mRNA Structure are Significantly Constrained in the Human Population". GigaScience Database. 2020.

113 rna-stability, RRID:SCR_019259

Table 1.

Context	Constrained against	R^2	p-value	Mediator	Prop. of variance explained by Mediator
**(A). ΔMFE – Structural Stability Constraint**					
CpG>CpA	Weaker structure	0.683	5.23e-69	-CpG content	0.769
CpG>TpG	Weaker structure	0.482	2.43e-45	-CpG content	0.746
C>G	Weaker structure	0.154	1.72e-29	+trailing G	0.156
G>T	Weaker structure	0.136	4.02e-22	+leading C	0.317
C>T	Weaker structure	0.125	1.67e-20	-leading G	0.134
T>C	Both	0.117*	5.97e-18*	+leading A	0.343
C>A	Weaker structure	0.0867	1.73e-16	+trailing G	0.332
G>A	Stronger structure	0.0354	1.64e-06	-trailing A	0.241
A>G	Stronger structure	0.0301	1.11e-05	+trailing T	0.335
G>C	Weaker structure	0.0183	0.000286	+leading C	0.227
A>C	Stronger structure	0.0112	0.00296	+leading C	0.0636
**(B). CED – Base-pairing constraint**					
CpG>CpA	Base-pair alteration	0.606	5.56e-15	-CpG content	0.787
C>A	Base-pair retention	0.396	2.51e-09	+CodonBase2=G	0.506
G>A	Base-pair retention	0.352	3.2e-08	-trailing A	0.607
CpG>TpG	Both	0.388*	3.79e-08*	-CpG content	0.563
T>C	Base-pair alteration	0.24	8.33e-06	+CodonBase2=A	0.666
C>T	Base-pair alteration	0.196	9.8e-05	+C content	0.414
G>C	Base-pair alteration	0.161	0.000444	+leading C	0.386
**(C). ΔCD – Diversity Constraint**					
CpG>CpA	Diversity changes	0.65	1.57e-14	-CpG content	0.849
G>A	Diversity maintenance	0.482	1.07e-09	-trailing A	0.621
CpG>TpG	Diversity changes	0.336	3.81e-06	+A content	0.418
C>A	Diversity maintenance	0.278	6.71e-06	+trailing G	0.443

Table 1. Structural metrics correlate with gnomAD frequency in most REF>ALT contexts. Correlation between structural metrics (**A**) ΔMFE, (**B**) CED and integer-rounded (**C**) ΔCD on the one hand, and the quantity P(MAF>0) on the other, over all sSNVs in a given context. The R^2 and p-values are obtained from a weighted least-squares linear regression, with the p-value corresponding to the linear coefficient; a quadratic regression was also performed, but only the p-value was retained as denoted by "*". Only context-metric pairs with p-value < 0.005 are included. "Normalized slope" was obtained by dividing slope of regression line by average P(MAF>0) in the context and then multiplying by range covered by metric in its central 90% of sSNVs. "Mediator" is raw sequence variable that explains largest proportion of structural trend *in this context*, with sign adjusted to correlate negatively with gnomAD frequency. "Mediator R^2" gives proportion of variance explained by the Mediator (see *Mediator variables* in **Results** for details).

TABLE 2.

Gene	Condition	SNP (GRCh38)	Context	SURF	SPI	ΔMFE	CED	ΔCD
**COMT**	Pain Sensitivity	rs4633<br>NC_000022.11:g.19962712C>T<br>NM_000754.3:c.186C>T<br>NP_000745.1:p.His62=	CpG>TpG	**9.7<br>CED**	3.8<br>[0.28]<br>58%	2.9<br>[-0.5]<br>31%	**9.7<br>[66]<br>89%**	4.6<br>[-4.0]<br>19%
**COMT**	Pain sensitivity	rs4818<br>NC_000022.11:g.19963684C>G<br>NM_000754.3:c.408C>G<br>NP_000745.1:p.Leu136=	C>G	**10.1<br>ΔMFE**	4.0<br>[-0.16]<br>60%	**10.1<br>[-3.0]<br>6%**	**5<br>[38]<br>68%**	**7.4<br>[-7.0]<br>10%**
**DRD2**	Schizophrenia, substance abuse	rs6277<br>NC_000011.10:g.113412737G>A<br>NM_000795.4:c.957C>T<br>NP_000786.1:p.Pro319=	CpG>TpG	**18.6<br>ΔMEAFE**	3.1<br>[0.35]<br>51%	4<br>[1.0]<br>75 %	**8.4<br>[60]<br>86%**	**9.8<br>[9.5]<br>94%**
**F2**	Thrombosis	rs72554028<br>NC_000011.10:g.46739363C>T<br>NM_000506.4:c.1824C>T<br>NP_000497.1:p.Arg608=	C>T	**15.9<br>MFEED**	1.4<br>[0.61]<br>28%	**5.9<br>[-1.7]<br>15%**	**11.2<br>[72]<br>92%**	**5.1<br>[-4.5]<br>17%**
**KRAS**	Cancer	NA<br>NC_000012.12:g.25245355T>G<br>NM_033360.3:c.30A>C<br>NP_203524.1:p.Gly10=	A>C	**10.2<br>EED**	2.8<br>[0.11]<br>48%	0.5<br>[0]<br>49%	5.2<br>[40]<br>70%	3.3<br>2.5<br>74%
**NKX2-5**	Congenital heart disease	rs72554028<br>NC_000005.10:g.173233001C>T<br>NM_004387.4:c.543G>A<br>NP_004378.1:p.Gln181	G>A	**12.6<br>ΔEFE**	4<br>[-0.18]<br>60%	**11.5<br>[3.5]<br>96%**	1.1<br>[4]<br>22%	0.2<br>[0.0]<br>50%
**NKX2-5**	Congenital heart disease	rs2277923<br>NC_000005.10:g.173235021T>C<br>NM_004387.3:c.63A>G<br>NP_004378.1:p.Glu21=	A>G	**9.8<br>ΔEND**	1.9<br>[0.41]<br>36%	0.5<br>[0]<br>49%	2.9<br>[20]<br>49%	**9.3<br>[9.0]<br>94%**
**OPTC**	Primary open angle glaucoma	rs559635109<br>NC_000001.11:g.203498796C>T<br>NM_014359.3:c.486C>T<br>NP_055174.1:p.Phe162=	C>T	**9.5<br>ΔCFE**	**5.7<br>[-0.57]<br>73%**	0.5<br>[0]<br>49%	4.3<br>[32]<br>63%	3.3<br>[2.5]<br>74%
**TP53**	Cancer	rs748527030<br>NC_000017.11:7676528:T:C<br>NM_000546.5:c.66A>G<br>NP_000537.3:p.Leu22=	A>G	**13.1<br>ΔCD**	0.1<br>[1.95]<br>2%	2.2<br>[-0.2]<br>36%	2.9<br>[20]<br>49%	**13.1<br>[12.5]<br>97%**

TABLE 2. Known sSNVs clinically implicated for structural pathogenicity are successfully predicted to be pathogenic by our structural metrics. dbSNP RS number and standardized SNV annotations are provided, along with the gene's official symbol and disease the sSNV has been associated with. SURF scores are shown, along with the metric that produced that score (i.e., for the first sSNV in the table, the highest Phred-scaled value across all 11 metrics was of 9.7, observed with the CED metric). Phred scores for SPI, ΔMFE, CED and ΔCD are also provided (top value), along with the metrics raw value ([middle value]) and percentile value (bottom value). For all scores, the greater the Phred-scaled value the greater the predicted change to the RNA structure. Any score greater than 5 (our suggested minimum cutoff, representing the 3rd quartile for the metric) is highlighted in bold.

FIGURE LEGENDS

FIGURE 1. A synonymous variant introduces a marked change in local minimum free energy of the mRNA secondary structures in the *DRD2* gene. Using a known synonymous variant of pharmacogenomic significance in the dopamine receptor, DRD2 (NM_000795.4:c.957C>T (p.Pro319=)), this figure demonstrates how the 101-bp window used in our analysis captures the variant's impact on RNA secondary structure. Wildtype (**A**) and mutant (**B** and **C**) sequences (RefSeq transcript NM_000795.4, coding positions 907-1008) are identical except for a synonymous C->T mutation at position 51 (major "C" allele is indicated by the black arrow, minor "T" allele is indicated by the red arrow). **(A)** Wildtype optimal and centroid structures (which coincide) demonstrate a relatively stable secondary structure with a minimum free energy of -12.5 kcal/mol. In the ensemble of possible structures arising from the sSNV a position 51, there is a significant reduction in stability of the molecule in terms of both the (**B)** mutant optimal structure (-11.5 kcal/mol) and **(C)** mutant centroid structure (-5.1 kcal/mol). The synonymous variant results in a less stable mRNA molecule which laboratory studies demonstrate reduces the half-life of the transcript, ultimately reducing protein expression of the dopamine receptor, DRD2. Nucleotides are colored according to the type of structure that they are in: Green: Stems (canonical helices); Red: Multiloops (junctions); Yellow: Internal Loops; Blue: Hairpin loops; Orange: 5' and 3' unpaired region.

FIGURE 2. Graphical depiction of computational workflow used to generate ViennaRNA folding metrics for the entire transcriptome. The entire analysis workflow was parallelized using Apache Spark and the Amazon Elastic Map Reduce (EMR) service, generating 5 billion ViennaRNA metrics over the course of 2 days. Using a custom pipeline developed for the process that was executed across 47 Amazon Elastic Cloud Compute (EC2) spot instances, input data was retrieved from an Amazon Simple Storage Solution (S3) bucket and processed through the pipeline consisting of 8 steps. We first obtained the 101-base sequence centered around a SNV in a transcript and generated three alternate sequences (with the ALT rather than the REF at position 51) (step 1). We next applied ViennaRNA modules to sequence to obtain structural metrics (step 2). Results were then mapped to chromosomal coordinates (step 3) and annotated with SnpEff to identify splice variants (step 4), annotated with gnomAD population frequencies (step 5) and coverage information (step 6), and finally annotated with metrics from

dbNSFP (step 7). Final dataset was written to Amazon S3 in Parquet columnar file format for further analysis and interpretation.

FIGURE 3. Exonic SNVs predicted to impact mRNA structure are constrained in the human population. Population frequency of SNVs was plotted against predicted impact on mRNA structure. Circles show proportion of SNVs with nonzero gnomAD exonic frequency at each value of the RNA stability metric ΔMFE. The bell-shaped pattern of constraint was observed across all classes of SNVs, with constraint appearing to be greatest in sSNVs (red), followed by SNVs in the 5-prime UTR (orange), then SNVs in the 3-prime UTR (blue), and finally nsSNVs (green). Values of ΔMFE with fewer than 2000 (synonymous), 200 (UTRs) or 5000 (missense) positive-MAF sSNVs are excluded. Only SNVs passing all filters for both WGS and WES data are represented (see **METHODS** for details).

FIGURE 4. Synonymous variants predicted to impact mRNA structure are constrained in the human population. Population frequency of sSNVs was plotted against the predicted impact on mRNA structure. Synonymous variants that disrupt structure tend to be absent from the gnomAD database, while those with limited impact on structure appear at least once in the gnomAD database. **(A)** Proportion of sSNVs with nonzero gnomAD frequency at each value of the RNA stability metric ΔMFE. Color represents average CED value, to highlight the relationship between minimum free energy and edit distance. **(B)** Analogous plot for metric CED measuring edge differences between mutant/wildtype centroid structures. Color represents |ΔMFE|, measuring absolute change in stability. **(C)** Analogous plot for diversity-metric ΔCD measuring change in structural ensemble diversity due to sSNV. Color is by ΔMFE measuring change in stability. Metric values with fewer than 2,500 (ΔMFE), 7,500 (CED) or 3,500 (ΔCD) positive-MAF sSNVs excluded.

FIGURE 5. Synonymous CpG transitions are markedly constrained against destabilization of their mRNA structures. Population frequency of sSNV vs. effect on mRNA structure in synonymous CpG transitions was examined. Proportion of synonymous CpG transitions with nonzero MAF at each value of ΔMFE were

determined for (**A**) CpG>CpA and (**B**) CpG>TpG synonymous mutations. ΔMFE values with fewer than 75 nonzero-MAF sSNVs are excluded. Color gives average CED in each context, ranging from 15 (blue) to 50 (red). Similarly, proportion of synonymous CpG transitions with nonzero MAF at each value of CED were determined for (**C**) CpG>CpA sSNVs and (**D**) CpG>TpG sSNVs. Color represents average ΔMFE and ranges from -0.8 (blue) to 1.85 (red). CED values with fewer than 40 (CpG>CpA) or 75 (CpG>TpG) nonzero-MAF sSNVs are excluded). Finally, proportion of synonymous CpG transitions with nonzero MAF at each value of ΔCD (after rounding to nearest integer) were determined for (**E**) CpG>CpA and (**F**) CpG>TpG sSNVs sSNVS. Color represents average ΔMFE and ranges from -3 (blue) to 4 (red). Rounded ΔCD values with fewer than 250 (CpG>CpA) or 20 (CpG>TpG) nonzero-MAF sSNVs are excluded.

FIGURE 6. SPI score correlates with constraint in synonymous variants. Variants are grouped by Phred-scaled SPI integer values into 33 bins, with the number of sSNVs per bin ranging from ~1,000,000 (large circles) to ~5,000 SNVs (small circles). The corresponding value of P(MAF>0) was plotted against the Phred-scaled SPI score of each bin (red circles) and fitted with a smoothed loess curve (red line). A clear correlation between global constraint and increasing score can be observed, with all scores >= 5 (our suggested minimum cutoff, dashed arrow) demonstrating constraint in P(MAF>0) below that of the average seen in sSNVs globally (grey line). To assess the power of this correlation as compared to random chance, SPI scores were randomly shuffled and the MAF distribution of the shuffled SPI scores plotted (grey circles). Across all Phred-scaled SPI bins, the P(MAF>0) for the shuffled data remains at or close to the expected global average of 13.8%, calculated for all 17 million sSNVs that had sufficient coverage in gnomAD to determine MAF. This clearly demonstrates that sSNVs high Phred-scaled SPI scores are constrained (red arrow), while those with a low score demonstrate greater plasticity (green arrow), with an increased probability that their MAF is greater than 0. Shaded area represents 90th percentile confident intervals for both SPI (red) and shuffled SPI (grey).

FIGURE 7. SUmmarized RNA Folding (SURF) metrics correlate with constraint in synonymous variants. SPI and each of the ten RNA folding metrics were percentile ranked and Phred-scaled (-10 × log10(rank)),

such that the larger the Phred-scaled value the greater the predicted change in RNA structure. For each SNV in our dataset, the maximum Phred score was determined across (**A**) all 11 metrics – ***SURF***, (**B**) the four stability metrics (ΔMFE, ΔCFE, ΔMEAFE and ΔEFE) – ***SURF Stability***, (**C**) the four edge distance metrics (MFEED, CED, MEAED and EED) – ***SURF Edit Distance***, or (**D**) – the two diversity metrics (ΔCD and ΔEND) – ***SURF Diversity***. For each plot, variants are grouped by integer values into 36 bins (ranging from 0 to 40, i.e the 99.99th percentile). The corresponding value of P(MAF>0) was plotted against the SURF metric for each bin (red circles) and fitted with a smoothed loess curve (red line). Shaded area represents 90th percentile confident intervals for the given summary metric. Dashed red line indicates the average P(MAF>0) value of 13.8% seen in sSNVs globally. The dashed arrow indicates our suggested minimum cutoff of 5 for any given metric. Across all 4 summarized metrics, a clear correlation between global constraint and increasing score can be observed.

PDF of main figures and legends
Click here to access/download;Figure;RNA_stability_figures_GS.pdf

Figure 1

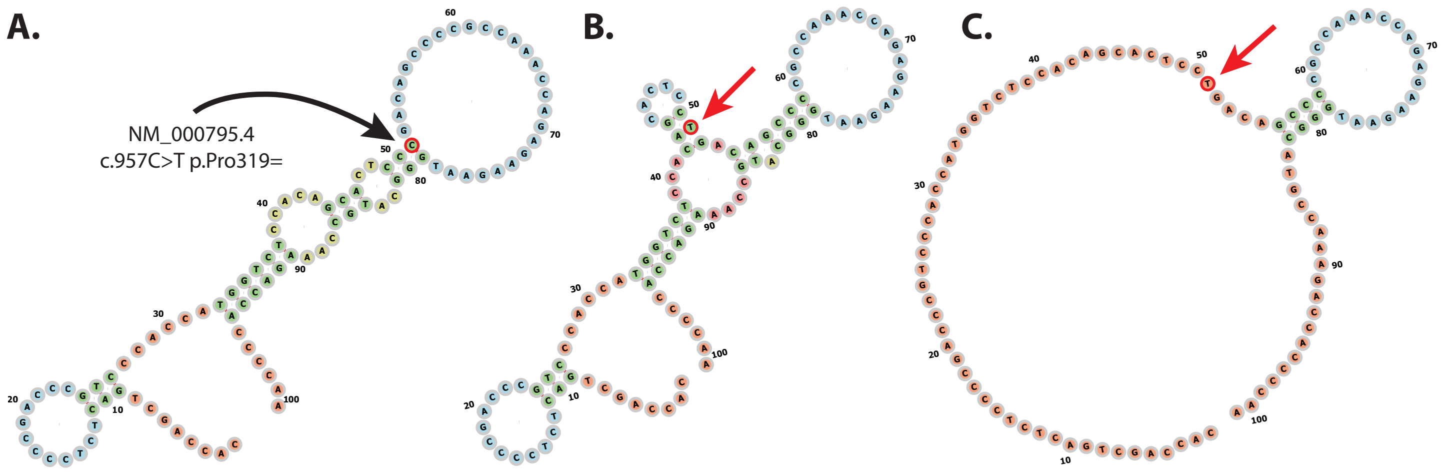

FIGURE 1. A synonymous variant introduces a marked change in local minimum free energy of the mRNA secondary structures in the DRD2 gene. Using a known synonymous variant of pharmacogenomic significance in the dopamine receptor, DRD2 (NM_000795.4:c.957C>T (p.Pro319=)), this figure demonstrates how the 101-bp window used in our analysis captures the variant's impact on RNA secondary structure. Wildtype (**A**) and mutant (**B** and **C**) sequences (RefSeq transcript NM_000795.4, coding positions 907-1008) are identical except for a synonymous C->T mutation at position 51 (major "C" allele is indicated by the black arrow, minor "T" allele is indicated by the red arrow). (**A**) Wildtype optimal and centroid structures (which coincide) demonstrate a relatively stable secondary structure with a minimum free energy of -12.5 kcal/mol. In the ensemble of possible structures arising from the sSNV a position 51, there is a significant reduction in stability of the molecule in terms of both the (**B**) mutant optimal structure (-11.5 kcal/mol) and (**C**) mutant centroid structure (-5.1 kcal/mol). The synonymous variant results in a less stable mRNA molecule which laboratory studies demonstrate reduces the half-life of the transcript, ultimately reducing protein expression of the dopamine receptor, DRD2. Nucleotides are colored according to the type of structure that they are in: Green: Stems (canonical helices); Red: Multiloops (junctions); Yellow: Internal Loops; Blue: Hairpin loops; Orange: 5' and 3' unpaired region.

Figure 2

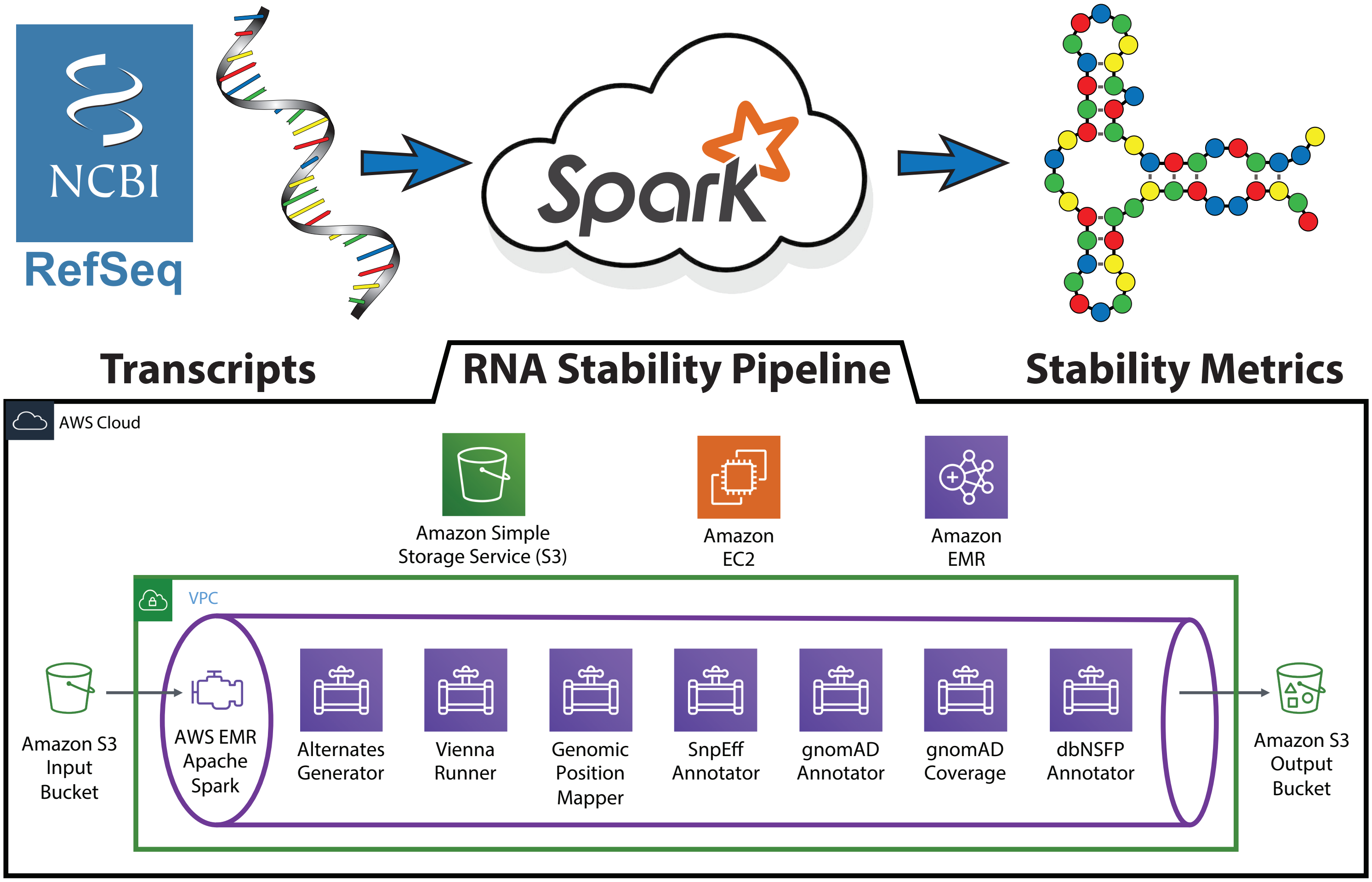

FIGURE 2. Graphical depiction of computational workflow used to generate ViennaRNA folding metrics for the entire transcriptome. The entire analysis workflow was parallelized using Apache Spark and the Amazon Elastic Map Reduce (EMR) service, generating 5 billion ViennaRNA metrics over the course of 2 days. Using a custom pipeline developed for the process that was executed across 47 Amazon Elastic Cloud Compute (EC2) spot instances, input data was retrieved from an Amazon Simple Storage Solution (S3) bucket and processed through the pipeline consisting of 8 steps. We first obtained the 101-base sequence centered around a SNV in a transcript and generated three alternate sequences (with the ALT rather than the REF at position 51) (step 1). We next applied ViennaRNA modules to sequence to obtain structural metrics (step 2). Results were then mapped to chromosomal coordinates (step 3) and annotated with SnpEff to identify splice variants (step 4), annotated with gnomAD population frequencies (step 5) and coverage information (step 6), and finally annotated with metrics from dbNSFP (step 7). Final dataset was written to Amazon S3 in Parquet columnar file format for further analysis and interpretation.

FIGURE 3

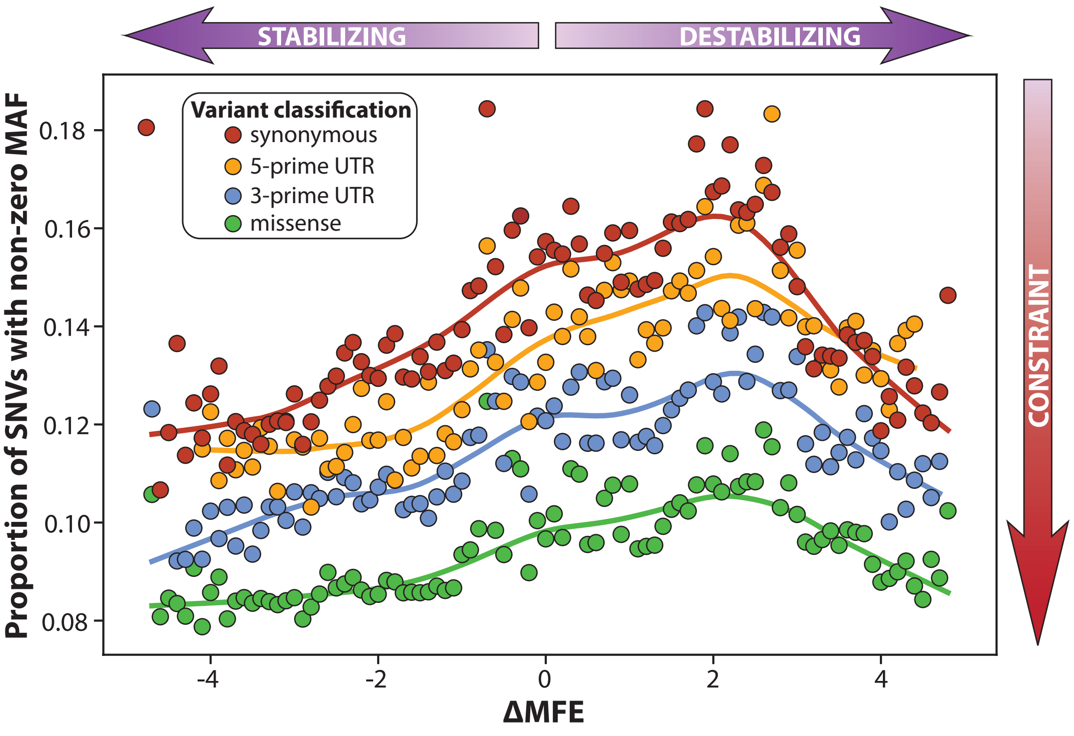

FIGURE 3. Exonic SNVs predicted to impact mRNA structure are constrained in the human population. Population frequency of SNVs was plotted against predicted impact on mRNA structure. Circles show proportion of SNVs with nonzero gnomAD exonic frequency at each value of the RNA stability metric ΔMFE. The bell-shaped pattern of constraint was observed across all classes of SNVs, with constraint appearing to be greatest in sSNVs (red), followed by SNVs in the 5-prime UTR (orange), then SNVs in the 3-prime UTR (blue), and finally nsSNVs (green). Values of dMFE with fewer than 2000 (synonymous), 200 (UTRs) or 5000 (missense) positive-MAF sSNVs are excluded. Only SNVs passing all filters for both WGS and WES data are represented (see **METHODS** for details).

FIGURE 4

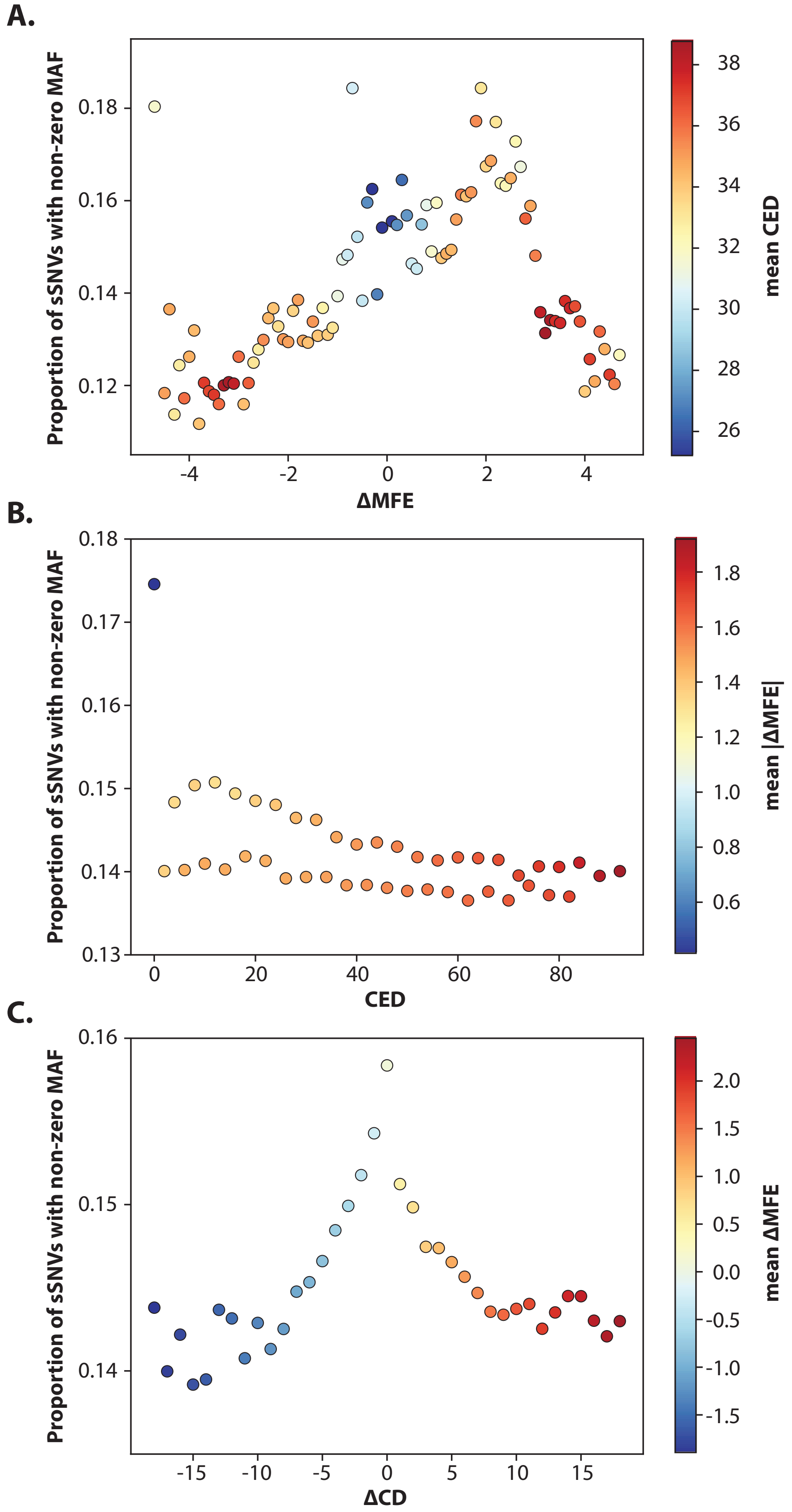

FIGURE 4. Synonymous variants predicted to impact mRNA structure are constrained in the human population. Population frequency of sSNVs was plotted against the predicted impact on mRNA structure. Synonymous variants that disrupt structure tend to be absent from the gnomAD database, while those with limited impact on structure appear at least once in the gnomAD database. (**A**) Proportion of sSNVs with nonzero gnomAD frequency at each value of the RNA stability metric ΔMFE. Color represents average CED value, to highlight the relationship between minimum free energy and edit distance. (**B**) Analogous plot for metric CED measuring edge differences between mutant/wildtype centroid structures. Color represents |ΔMFE|, measuring absolute change in stability. (**C**) Analogous plot for diversity-metric ΔCD measuring change in structural ensemble diversity due to sSNV. Color is by ΔMFE measuring change in stability. Metric values with fewer than 2,500 (ΔMFE), 7,500 (CED) or 3,500 (ΔCD) positive-MAF sSNVs excluded.

FIGURE 5

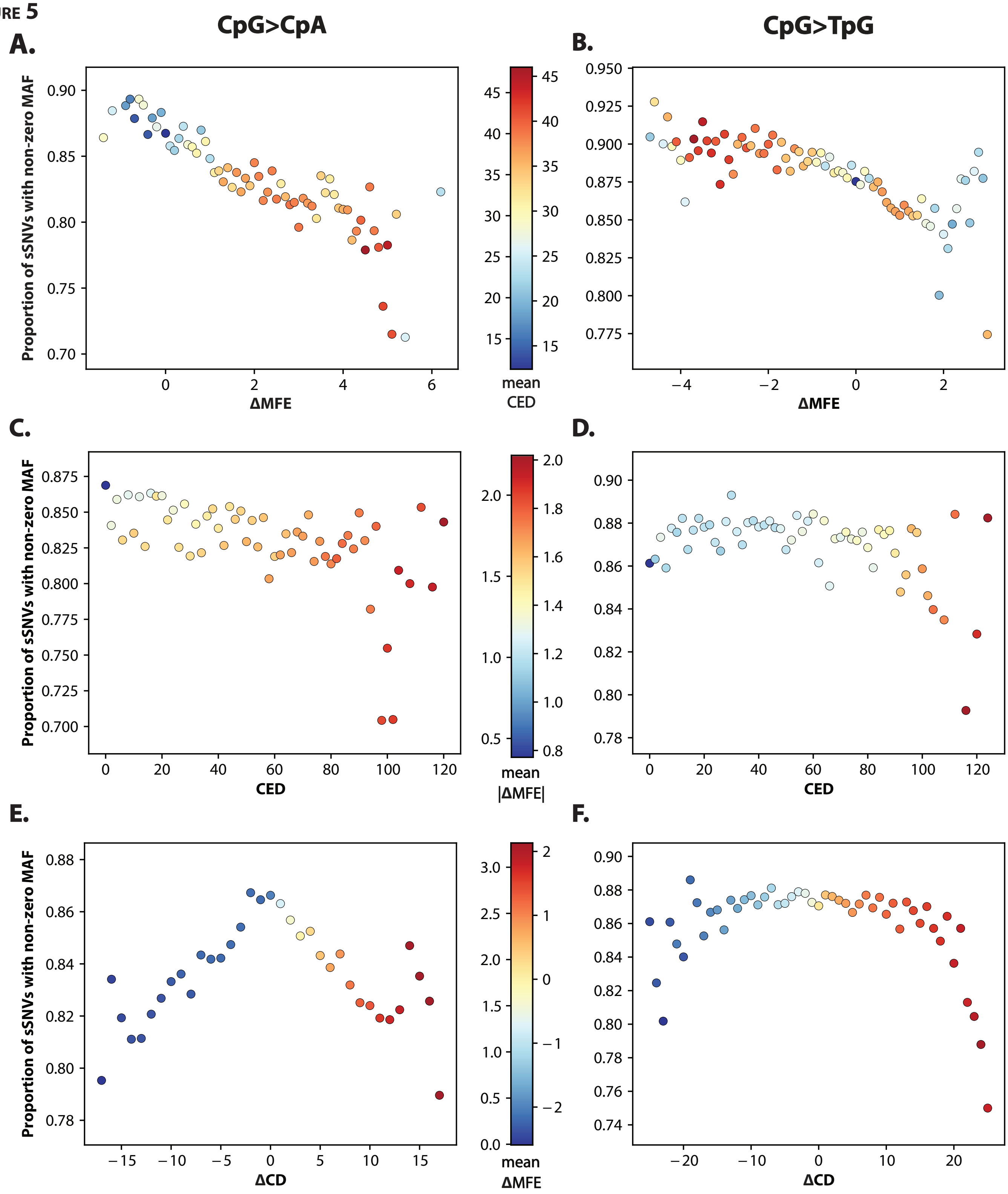

FIGURE 5. Synonymous CpG transitions are markedly constrained against destabilization of their mRNA structures. Population frequency of sSNV vs. effect on mRNA structure in synonymous CpG transitions was examined. Proportion of synonymous CpG transitions with nonzero MAF at each value of ΔMFE were determined for (**A**) CpG>CpA and (**B**) CpG> TpG synonymous mutations. ΔMFE values with fewer than 75 nonzero-MAF sSNVs are excluded. Color gives average CED in each context, ranging from 15 (blue) to 50 (red). Similarly, proportion of synonymous CpG transitions with nonzero MAF at each value of CED were determined for (**C**) CpG>CpA sSNVs and (**D**) CpG>TpG sSNVs. Color represents average ΔMFE and ranges from -0.8 (blue) to 1.85 (red). CED values with fewer than 40 (CpG>CpA) or 75 (CpG>TpG) nonzero-MAF sSNVs are excluded. Finally, proportion of synonymous CpG transitions with nonzero MAF at each value of ΔCD (after rounding to nearest integer) were determined for (**E**) CpG>CpA and (**F**) CpG>TpG sSNVs sSNVS. Color represents average ΔMFE and ranges from -3 (blue) to 4 (red). Rounded ΔCD values with fewer than 250 (CpG>CpA) or 20 (CpG>TpG) nonzero-MAF sSNVs are excluded.

Figure 6

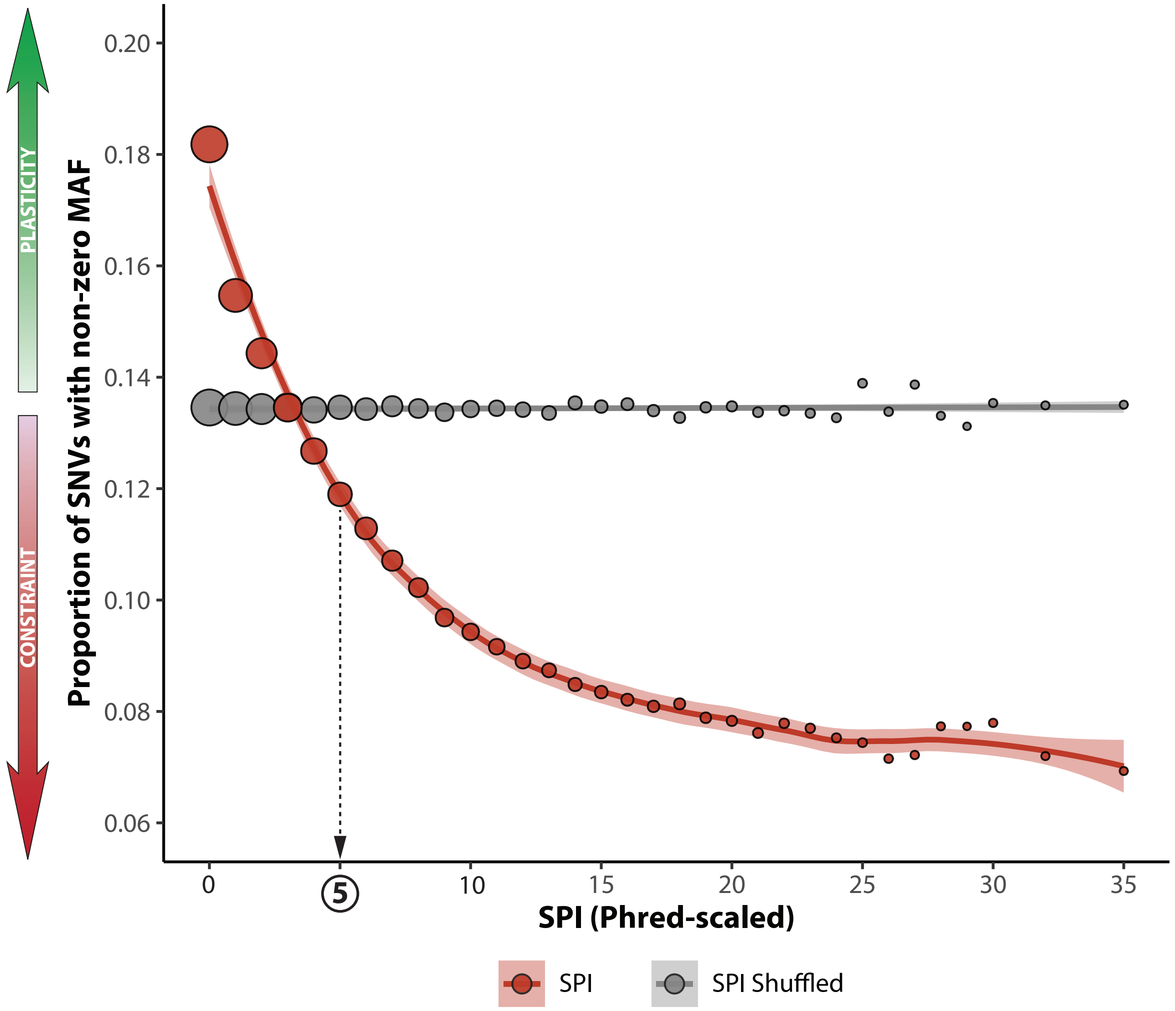

FIGURE 6. SPI score correlates with constraint in synonymous variants. Variants are grouped by Phred-scaled SPI integer values into 33 bins, with the number of sSNVs per bin ranging from ~1,000,000 (large circles) to ~5,000 SNVs (small circles). The corresponding value of P(MAF>0) was plotted against the Phred-scaled SPI score of each bin (red circles) and fitted with a smoothed loess curve (red line). A clear correlation between global constraint and increasing score can be observed, with all scores >= 5 (our suggested minimum cutoff, dashed arrow) demonstrating constraint in P(MAF>0) below that of the average seen in sSNVs globally (grey line). To assess the power of this correlation as compared to random chance, SPI scores were randomly shuffled and the MAF distribution of the shuffled SPI scores plotted (grey circles). Across all Phred-scaled SPI bins, the P(MAF>0) for the shuffled data remains at or close to the expected global average of 13.8%, calculated for all 17 million sSNVs that had sufficient coverage in gnomAD to determine MAF. This clearly demonstrates that sSNVs high Phred-scaled SPI scores are constrained (red arrow), while those with a low score demonstrate greater plasticity (green arrow), with an increased probability that their MAF is greater than 0. Shaded area represents 90th percentile confident intervals for both SPI (red) and shuffled SPI (grey).

Figure 7

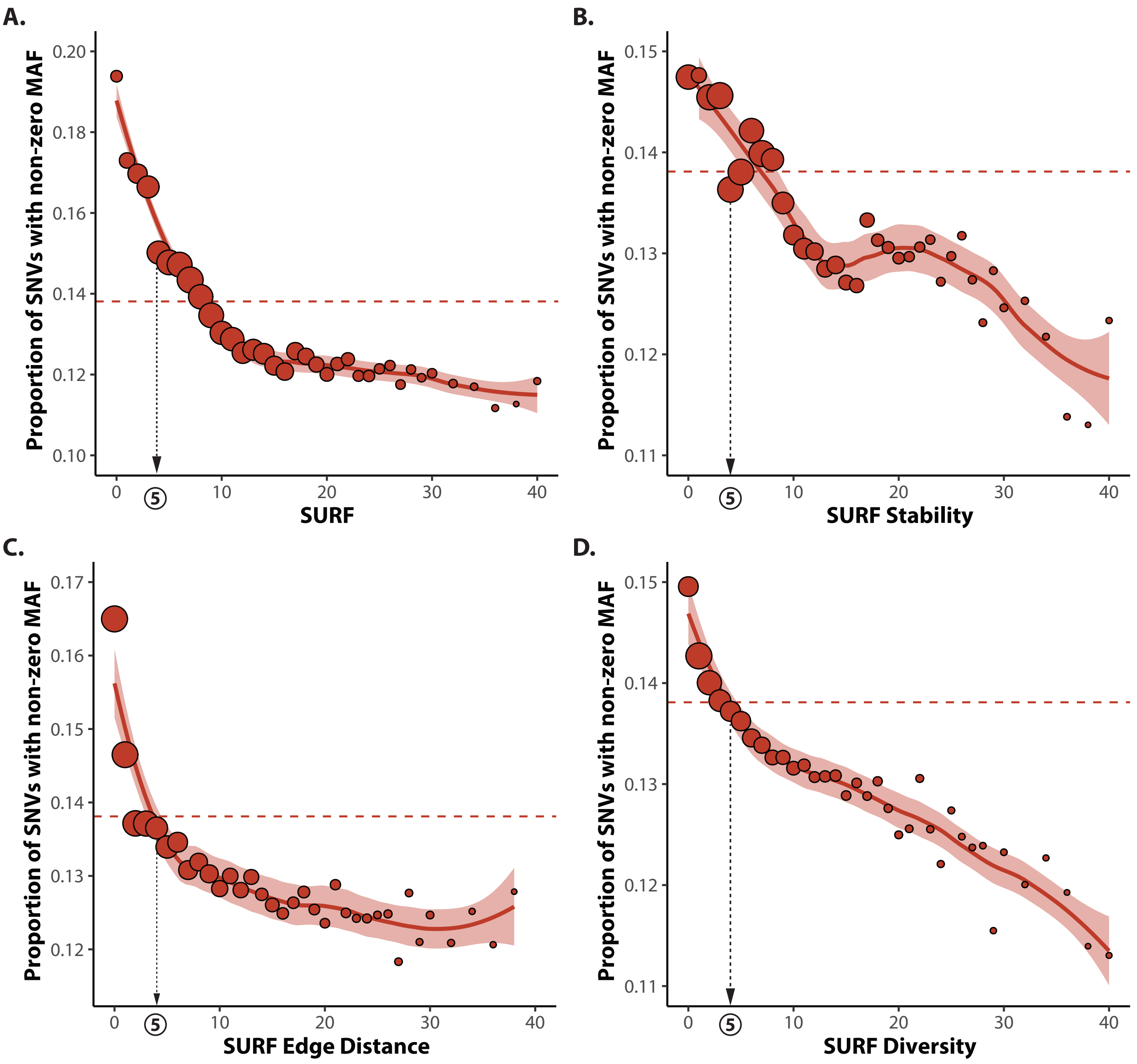

FIGURE 7. SUmmarized RNA Folding (SURF) metrics correlate with constraint in synonymous variants. SPI and each of the ten RNA folding metrics were percentile ranked and Phred-scaled (-10 × log10(rank)), such that the larger the Phred-scaled value the greater the precited change in RNA structure. For each SNV in our dataset, the maximum Phred score was determined across (**A**) all 11 metrics – ***SURF***, (**B**) the four stability metrics (ΔMFE, ΔCFE, ΔMEAFE and Δ EFE) – ***SURF Stability***, (**C**) the four edge distance metrics (MFEED, CED, MEAED and EED) – ***SURF Edit Distance***, or (**D**) – the two diversity metrics (ΔCD and ΔEND) – ***SURF Diversity***. For each plot, variants are grouped by integer values into 36 bins (ranging from 0 to 40, i.e the 99.99th percentile). The corresponding value of P(MAF>0) was plotted against the SURF metric for each bin (red circles) and fitted with a smoothed loess curve (red line). Shaded area represents 90th percentile confident intervals for the given summary metric. Dashed red line indicates the average P(MAF>0) value of 13.8% seen in sSNVs globally. The dashed arrow indicates our suggested minimum cutoff of 5 for any given metric. Across all 4 summarized metrics, a clear correlation between global constraint and increasing score can be observed.

Figure 2 (eps)

Click here to access/download;Figure;Figure_2.eps

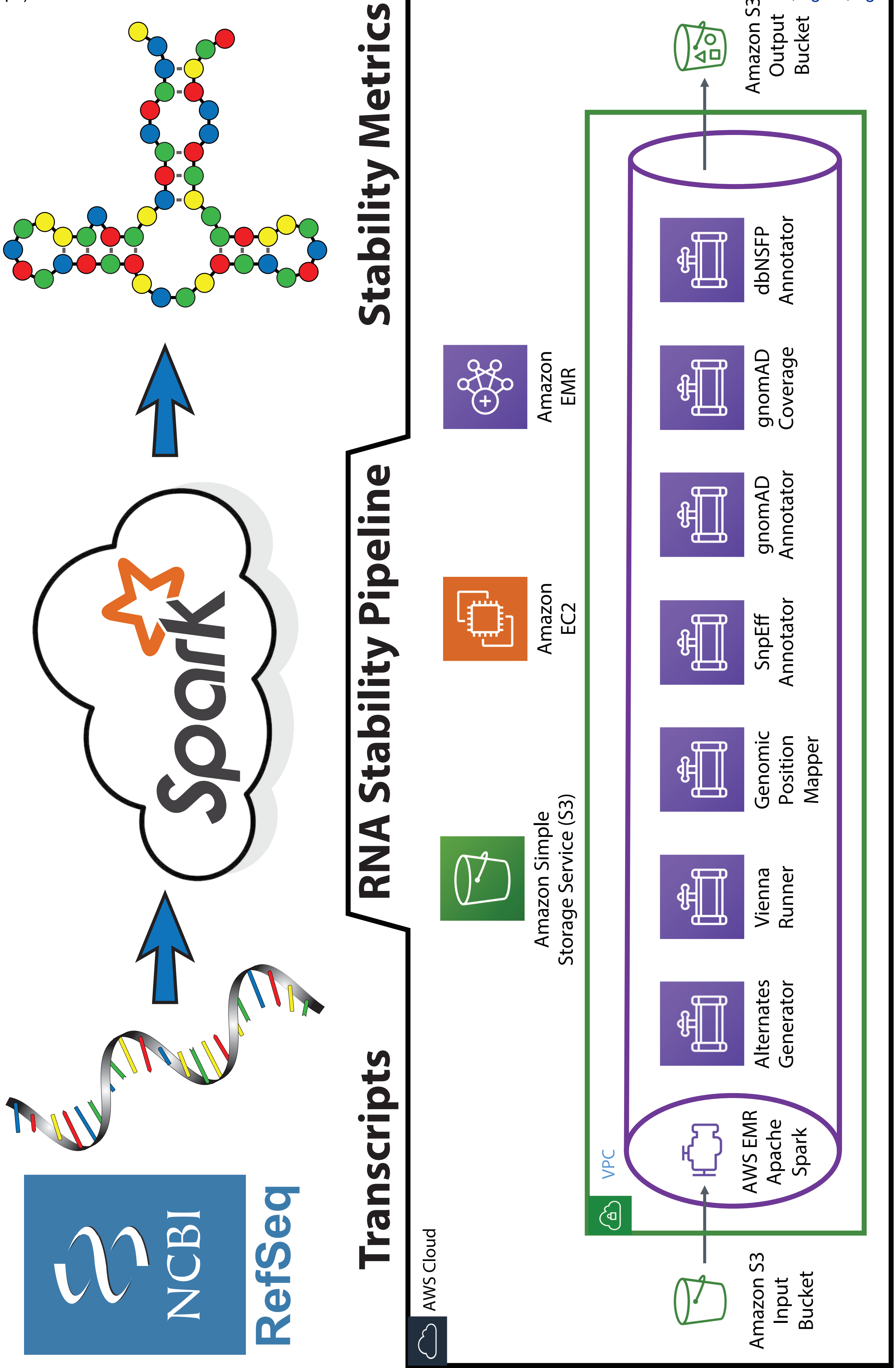

Figure 3 (eps)

Click here to access/download;Figure;Figure_3.eps

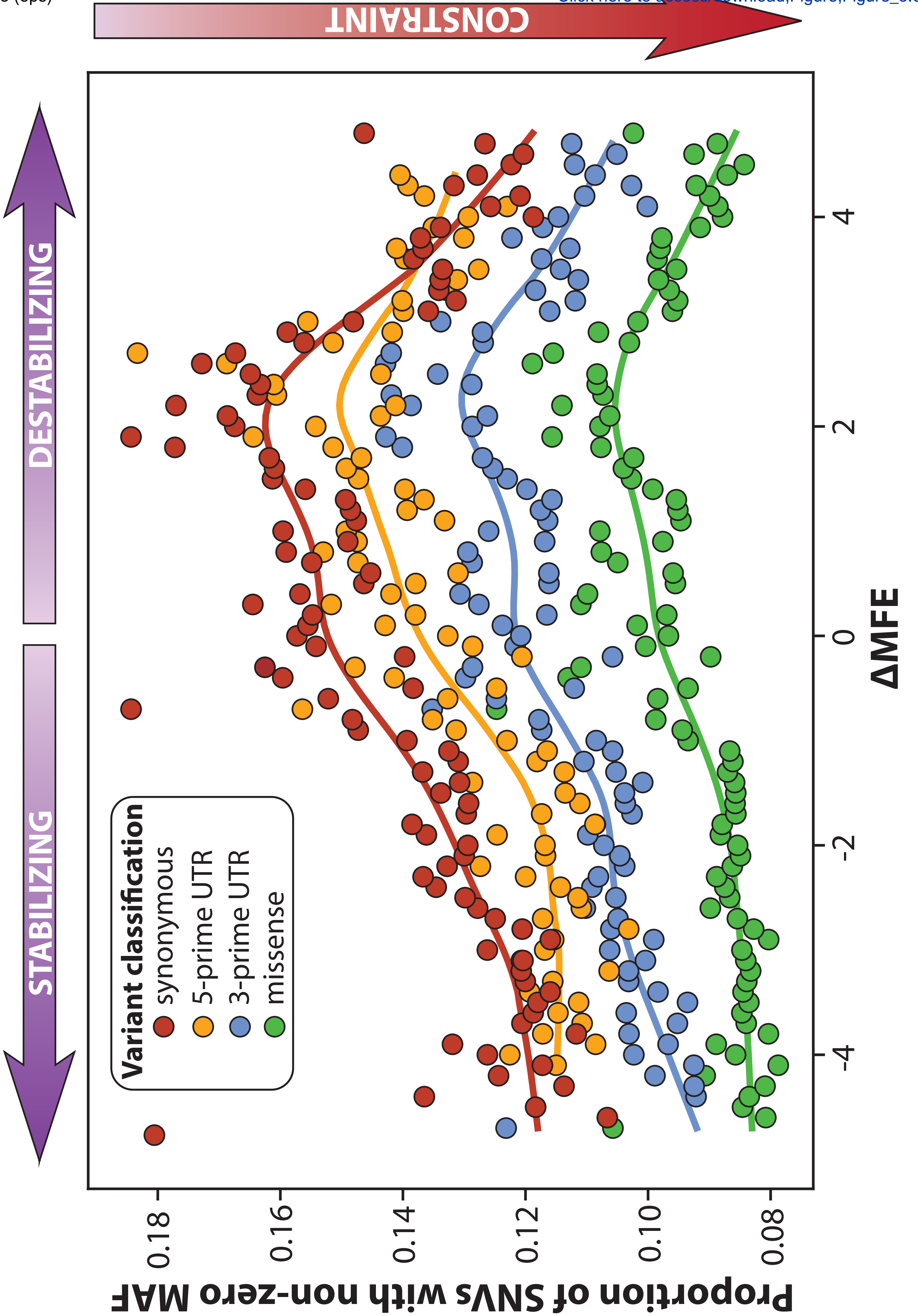

Figure 4 (eps)

Click here to access/download;Figure;Figure_4.eps

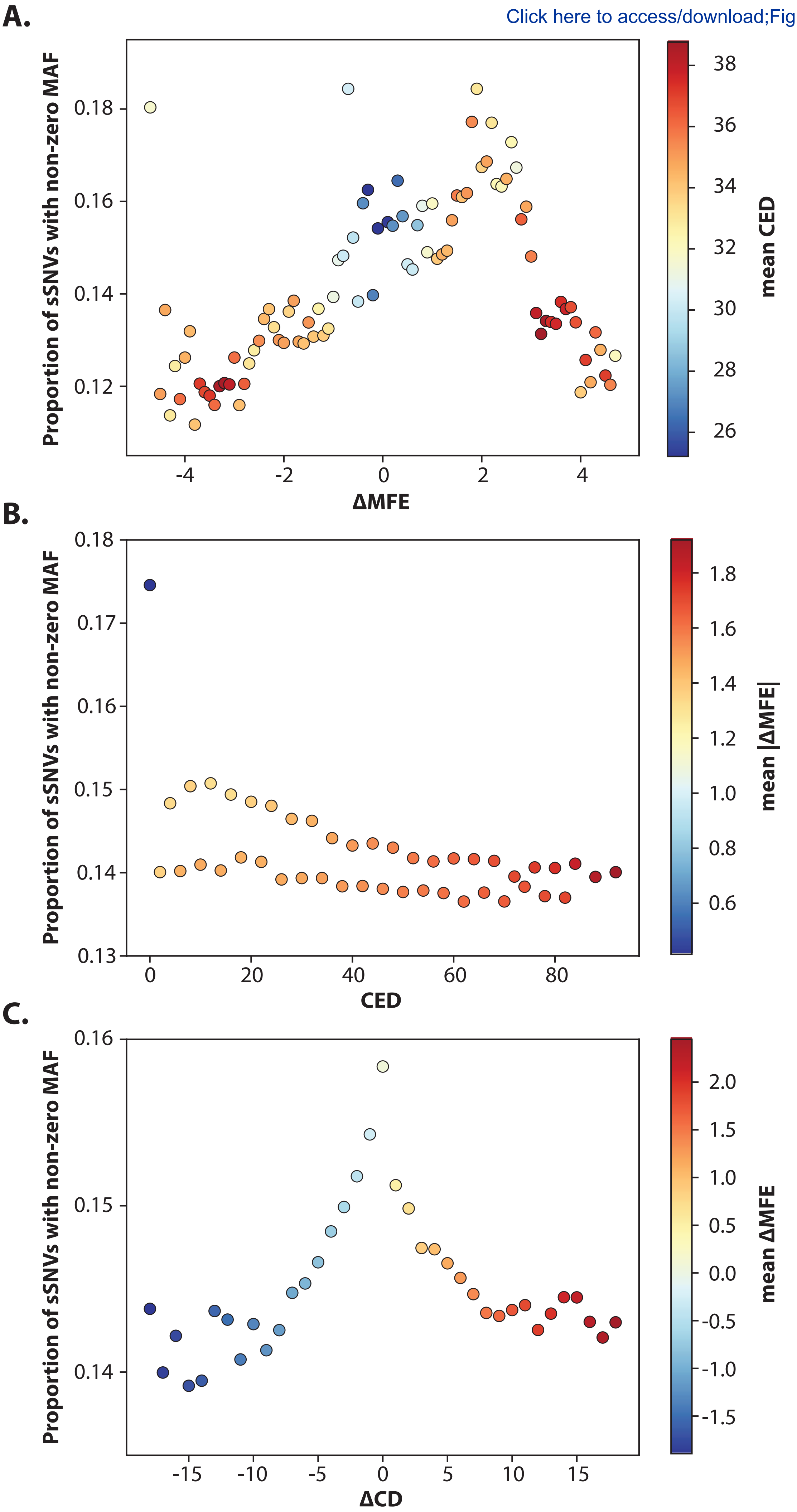

Figure 5 (eps) Click here to access/download;Figure;Figure_5.eps

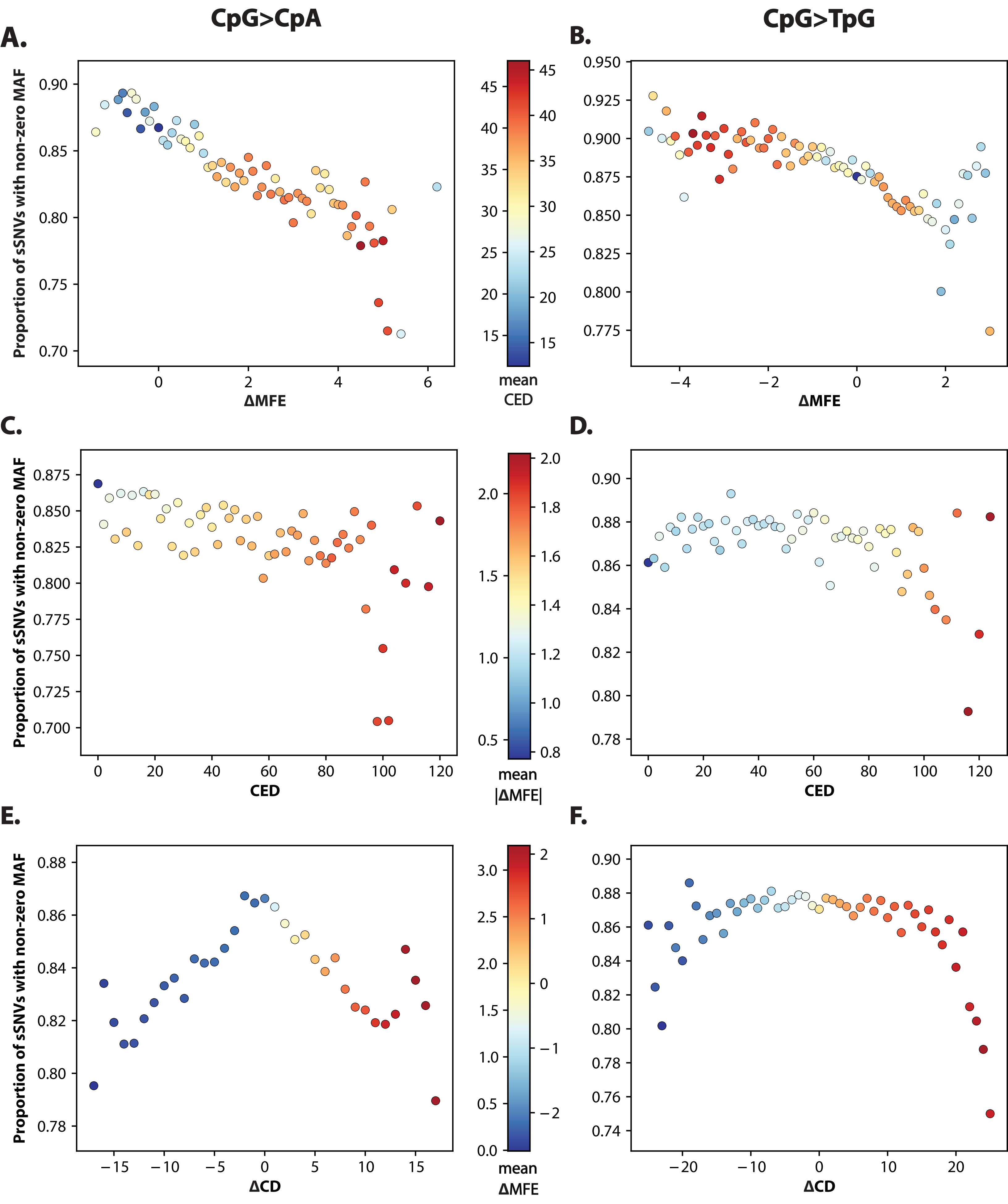

Figure 6 (eps)

Click here to access/download;Figure;Figure_6.eps

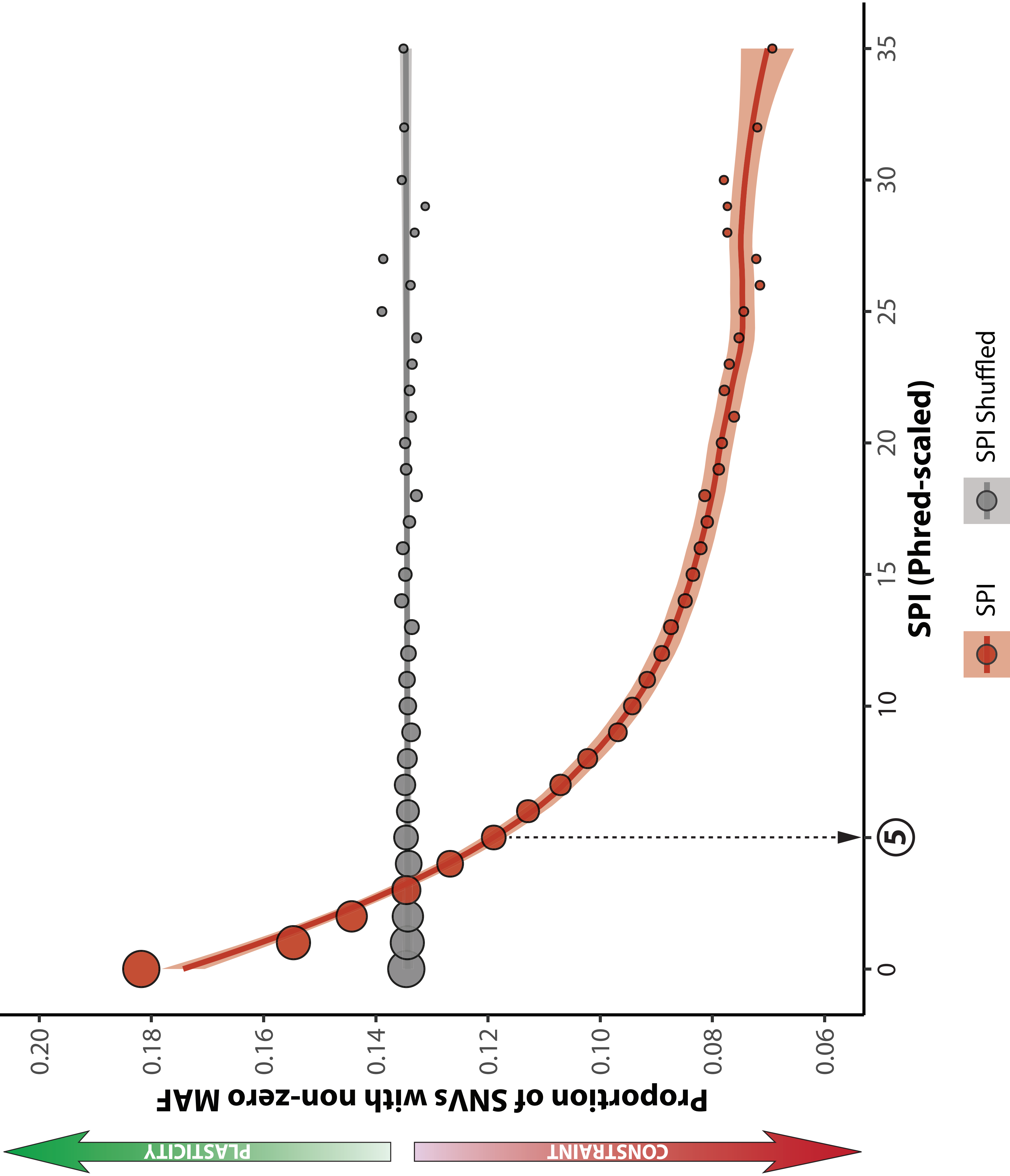

Figure 7 (eps)

Click here to access/download;Figure;Figure_7.eps

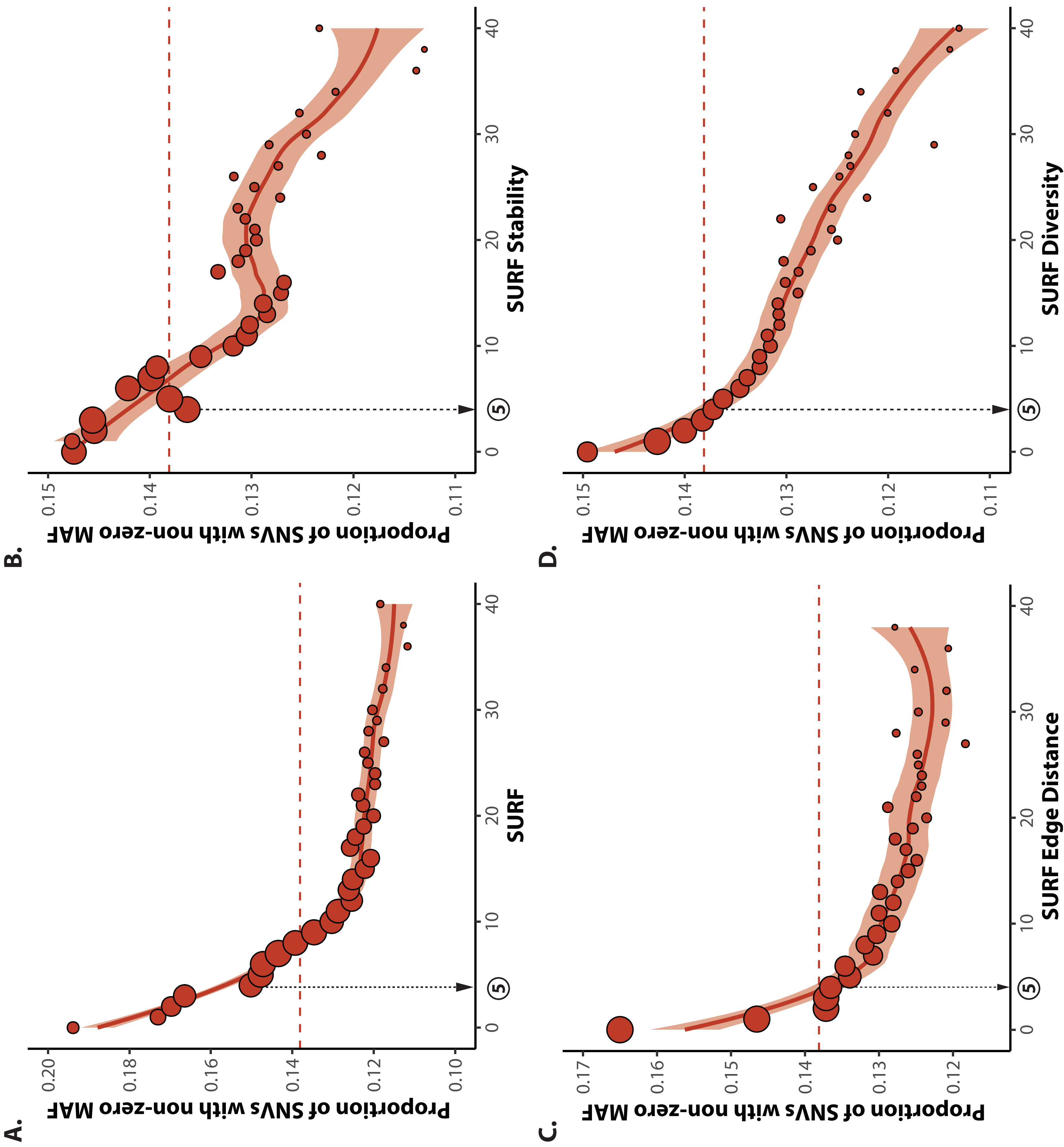

Supplementary Material (pdf)

Click here to access/download
Supplementary Material
RNA_stability_supplement_GS.pdf

Click here to access/download
Supplementary Material
RNA_stability_Review_Response_and_Redline.pdf
