# Supplementary material for: Synonymous variants that disrupt messenger RNA structure are significantly constrained in the human population
Source: Gigascience. 2021 Apr 5;10(4):giab023. doi: 10.1093/gigascience/giab023 (PMC8023685; doi:10.1093/gigascience/giab023)
Supplement: giab023_Supplemental_File [file giab023_supplemental_file.pdf]

-- SUPPLEMENTARY DATA --

SYNONYMOUS VARIANTS THAT DISRUPT mRNA STRUCTURE ARE SIGNIFICANTLY CONSTRAINED IN THE HUMAN POPULATION

Jeffrey B.S. Gaither[1], Grant E. Lammi[1], James L. Li[1], David M. Gordon[1], Harkness C. Kuck[1], Benjamin J. Kelly[1], James R. Fitch[1] and Peter White[1,2, *]

[1] Computational Genomics Group, The Institute for Genomic Medicine, Nationwide Children's Hospital, Columbus, Ohio, USA

[2] Department of Pediatrics, College of Medicine, The Ohio State University, Columbus, Ohio, USA

* To whom correspondence should be addressed. Tel: +1 (614) 355-2671; Fax: +1 (614) 355-6833; Email: peter.white@nationwidechildrens.org

Mailing address:

Prof. Peter White, PhD

The Institute for Genomic Medicine

Nationwide Children's Hospital

575 Children's Crossroad

Columbus, OH 43215. USA

TABLE OF CONTENTS

Supplementary Data Table 1 - Vienna RNA Metrics 3
Supplementary Data Table 2 - Constraint Across Sequence Contexts 4
Supplementary Data Table 3 - Ssnv Contexts Across the Human Transcriptome 6
Supplementary Data Table 4 - Modelling Structural Constraint with SPI Score 7
Supplementary Data Table 5 - Data Pre-Processing Steps 8
Supplementary Data Figure 1 - Distribution of Structural Metrics 9
Supplementary Data Figure 2 - Calculation of Edit Distance 11
Supplementary Data Figure 3 - Structural Metrics Over All Synonymous SNVs 12
Supplementary Data Figure 4 - Structural Metrics in Contexts Constrained Against Destabilization..... 14
Supplementary Data Figure 5 - Structural Metrics in Contexts Constrained Against Over-Stabilization 15
Supplementary Data Figure 6 - Sequence Context and SPI 16
Supplementary Data Figure 7 - Structural Metrics vs. log(MAF) 18
Supplementary Data Figure 8 - Change in Codon Optimality vs. Mutation Rate 19

SUPPLEMENTARY DATA TABLE 1 - Vienna RNA Metrics

Vienna metric	Vienna metric abbreviation	Description
	Stability Metrics	
**ΔMFE**	Delta Minimum Free Energy	Change in stability of optimal mRNA structure
**ΔCFE**	Delta Centroid Free Energy	Change in stability of centroid mRNA structure
**ΔMEAFE**	Delta Maximum Expected Accuracy Free Energy	Change in stability of maximum expected accuracy mRNA structure
**ΔEFE**	Delta Ensemble Free Energy	Expected change in stability over all structures
	Edge distance metrics	
**MFEED**	Minimum Free Energy Edit Distance	Edge-changes in optimal structure
**CED**	Centroid Edit Distance	Edge-changes in centroid structure
**MEAED**	Maximum Expected Accuracy Edit Distance	Edge-changes in maximum expected accuracy structure
**EED**	Ensemble Edit Distance	Expected edge-changes over all structures
	Diversity metrics	
**ΔCD**	Delta Centroid Distance	Change in expected distance from centroid structure
**ΔEND**	Delta Ensemble Diversity	Change in expected distance between two structures

Supplementary Data Table 1. Description of the ten Vienna RNA metrics calculated through the Spark RNA stability pipeline. These metrics are divided into three classes: stability, edge distance and diversity.

SUPPLEMENTARY DATA TABLE 2 - Constraint Across Sequence Contexts

Context	Normalized slope	R^2	R^2 (quadratic)	p-value	p-value (quadratic)	Mediator	Prop. of variance explained by Mediator
**(A). ΔMFE – Structural Stability Constraint**							
CpG>CpA	-0.0877	0.683	0.683	5.23e-69	0.409	-CpG content	0.769
CpG>TpG	-0.063	0.482	0.521	2.43e-45	1.37e-06	-CpG content	0.746
C>G	-0.145	0.154	0.158	1.72e-29	0.0583	+trailing G	0.156
G>T	-0.136	0.136	0.15	4.02e-22	0.00166	+leading C	0.317
C>T	-0.0462	0.125	0.135	1.67e-20	0.00685	-leading G	0.134
T>C	0.0304	0.0172	0.117	0.000517	5.97e-18	+leading A	0.343
C>A	-0.0976	0.0867	0.145	1.73e-16	2.37e-12	+trailing G	0.332
G>A	0.0275	0.0354	0.0576	1.64e-06	0.000119	-trailing A	0.241
A>G	0.0451	0.0301	0.0348	1.11e-05	0.0816	+trailing T	0.335
G>C	-0.0497	0.0183	0.0278	0.000286	0.00841	+leading C	0.227
A>C	0.0435	0.0112	0.0118	0.00297	0.501	+leading C	0.0636
A>T	0.0427	0.00795	0.00801	0.0208	0.839	+trailing T	0.0636
T>G	0.000578	1.97e-06	0.000187	0.97	0.716	+leading C	0.00409
T>A	0.00378	4.74e-05	0.000133	0.856	0.808	+trailing C	0.00335
**(B). CED – Base-pairing constraint**							
CpG>CpA	-0.0486	0.606	0.606	5.56e-15	0.993	-CpG content	0.787
C>A	0.0576	0.396	0.416	2.51e-09	0.126	+CodonBase2=G	0.506
G>A	0.0369	0.352	0.36	3.2e-08	0.34	-trailing A	0.607
CpG>TpG	0.00556	0.0349	0.388	0.121	3.79e-08	-CpG content	0.563
T>C	-0.0371	0.24	0.405	8.33e-06	2.76e-05	+CodonBase2=A	0.666
C>T	-0.0211	0.196	0.569	9.8e-05	6.21e-11	+C content	0.414
G>C	-0.0425	0.161	0.345	0.000444	3.25e-05	+leading C	0.386
C>G	-0.0249	0.0967	0.103	0.0066	0.485	+leading C	0.0758
T>A	-0.0163	0.0193	0.0979	0.251	0.0184	+LeadingCodonBase3=A	0.0104
A>C	-0.0263	0.0522	0.168	0.0535	0.00275	+CodonBase2=C	0.164
A>G	0.00861	0.0315	0.0448	0.139	0.334	+trailing T	0.203
T>G	0.0193	0.026	0.0289	0.18	0.654	-codon tAI	0.0264
A>T	0.0177	0.0224	0.031	0.216	0.443	+codon tAI	0.0259
G>T	-0.000456	2.8e-05	0.00249	0.965	0.679	-codon tAI	0.113
**(C). ΔCD – Diversity Constraint**							
CpG>CpA	-0.00578	0.00575	0.65	0.565	1.57e-14	-CpG content	0.849
G>A	-0.00681	0.011	0.482	0.421	1.07e-09	-trailing A	0.621
CpG>TpG	-0.0048	0.0304	0.336	0.183	3.81e-06	+A content	0.418
C>A	0.0101	0.00675	0.278	0.509	6.71e-06	+trailing G	0.443
A>G	-0.00613	0.0167	0.0913	0.325	0.0348	-G content	0.0965
T>C	0.0023	0.00144	0.0434	0.768	0.11	+CodonBase2=A	0.264
A>T	-0.00715	0.00273	0.0217	0.689	0.293	+CodonBase2=T	0.0172
G>C	0.00283	0.000609	0.0172	0.845	0.311	+LeadingCodonBase3=C	0.0291
T>A	-0.0164	0.0155	0.024	0.336	0.475	-trailing A	0.00987
G>T	0.00184	0.000336	0.0118	0.887	0.407	-CodonBase2=A	0.0245
C>T	-0.00413	0.00759	0.00844	0.497	0.822	+C content	0.416
C>G	0.00656	0.00503	0.00636	0.575	0.774	+CodonBase1=A	0.0206
T>G	-0.00833	0.00471	0.0203	0.596	0.336	-trailing A	0.0732
A>C	0.00757	0.00319	0.00426	0.663	0.803	+leading C	0.0899

Supplementary Data Table 2. **Structural metrics correlate with gnomAD frequency in most REF>ALT contexts.** Regression statistics between structural metrics (**A**) ΔMFE, (**B**) CED and integer-rounded (**C**) ΔCD and P(MAF>0) over all sSNVs in context. The R^2 and p-values are obtained from a weighted least-squares linear regression, with the p-value corresponding to the linear coefficient. A quadratic regression was also performed, but only the p-value was retained. Only context-metric pairs with p-value < 0.005 are included. "Normalized slope" was obtained by dividing slope of regression line by average P(MAF>0) in the context and then multiplying by range covered by metric in its central 90% of sSNVs. "Mediator" is raw sequence variable that explains largest proportion of structural trend in this context, with sign adjusted to correlate positively with gnomAD frequency. "Mediator R^2" gives proportion of variance explained by the Mediator (see *Mediator variables* in **RESULTS** for details).

SUPPLEMENTARY DATA TABLE 3 - sSNV Contexts Across the Human Transcriptome

mRNA REF allele	mRNA ALT allele	Transition (Ti) / Transversion (Tv)	# synonymous variants	P(MAF>0)
A	G	Ti	1,575,610	0.18
A	C	Tv	1,088,457	0.054
A	T	Tv	891,778	0.042
C	T	Ti	2,454,991	0.23
C	G	Tv	1,171,097	0.097
C	A	Tv	1,479,806	0.078
G	A	Ti	1,818,884	0.215
G	T	Tv	916,003	0.091
G	C	Tv	917,277	0.082
T	C	Ti	2,087,918	0.154
T	G	Tv	883,128	0.051
T	A	Tv	1,023,223	0.035
**CpG Sequence Context**				
C	T	Ti	276,759	0.873
G	A	Ti	165,845	0.852

Supplementary Data Table 3. Synonymous variants in each sequence context. In each mRNA context we give the total number of possible synonymous variants in the human genome (subject to the filters imposed in Supplementary Data Table 2) as well as the proportion of sSNVs that appear in gnomAD (i.e. MAF >0).

SUPPLEMENTARY DATA TABLE 4 - Modelling Structural Constraint with SPI Score

Context	GLM AUC Training Dataset	GLM AUC Testing Dataset	XGB AUC Training Dataset	XGB AUC Testing Dataset	RF AUC Training Dataset	RF AUC Testing Dataset
**A>C**	0.54	0.539	0.494	0.492	0.455	0.455
**A>G**	0.615	0.615	0.504	0.503	0.421	0.421
**A>T**	0.52	0.516	0.49	0.486	0.429	0.424
**C>A**	0.596	0.595	0.518	0.516	0.452	0.452
**C>G**	0.556	0.556	0.503	0.504	0.438	0.438
**C>T**	0.556	0.556	0.502	0.502	0.479	0.48
**CpG>TpG**	0.697	0.697	0.517	0.512	0.365	0.365
**G>A**	0.546	0.545	0.501	0.501	0.465	0.465
**CpG>CpA**	0.599	0.598	0.513	0.51	0.339	0.338
**G>C**	0.606	0.605	0.503	0.499	0.409	0.408
**G>T**	0.59	0.59	0.496	0.493	0.391	0.392
**T>A**	0.55	0.548	0.489	0.486	0.463	0.461
**T>C**	0.609	0.608	0.506	0.505	0.452	0.453
**T>G**	0.548	0.547	0.489	0.488	0.477	0.474

Supplementary Data Table 4. Performance of SPI score under different model frameworks. In each context we test the power of an SPI score built under one of three different schemes (general logistic, random forest, and gradient-boosted trees) for predicting whether a sSNV has MAF>0. Metric AUC measures the area under the receiver operating characteristic curve, averaged over a 5-fold cross validation. We ultimately select the general logistic model (GLM) as overwhelmingly the most successful candidate.

Supplementary Data Table 5 - Data Pre-Processing Steps

Variant type	Total # SNVs, counted by transcript position	Total # SNVs, counted by chromosomal position	# SNVs passing gnomAD filter
**Synonymous**	51,793,895	22,050,267	**21,422,393**
**Missense**	166,989,719	70,138,465	68,186,900
**3' UTR**	172,391.085	73,294,881	69,300,248
**5' UTR**	24,618,205	12,883,930	12,101,624
**Other**	54,813,868	6,443,053	6,191,078
**Total**	470,606,772	184,810,596	177,202,243

Supplementary Data Table 5. Summary of data pre-processing steps. Vienna metrics were calculated for a total of 470,606,772 SNVs in all known transcripts. As multiple transcripts share the same exonic genomic coordinates, we first collapsed the data to 184,810,596 unique chromosome positions, assigning each variant based on the canonical transcript (i.e. represented in the MANE database or longest CDS when not in MANE). We next filtered out any variants that were flagged by gnomAD as lacking adequate gnomAD coverage or suspected to have an unreliable population frequency. Finally, variants marked as "synonymous" were extracted, giving us a core data set of 21,422,393 sSNVs.

Supplementary Data Figure 1 - Distribution of Structural Metrics

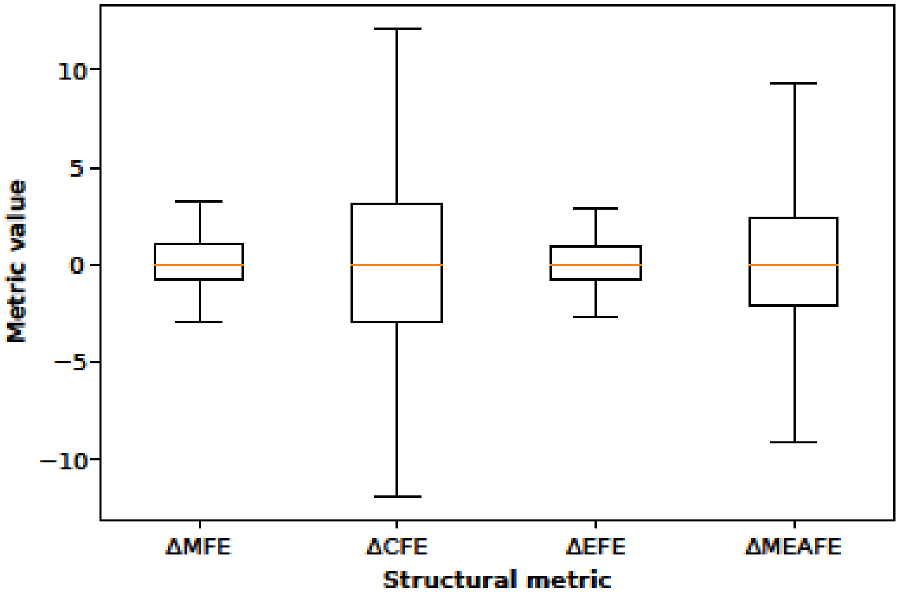

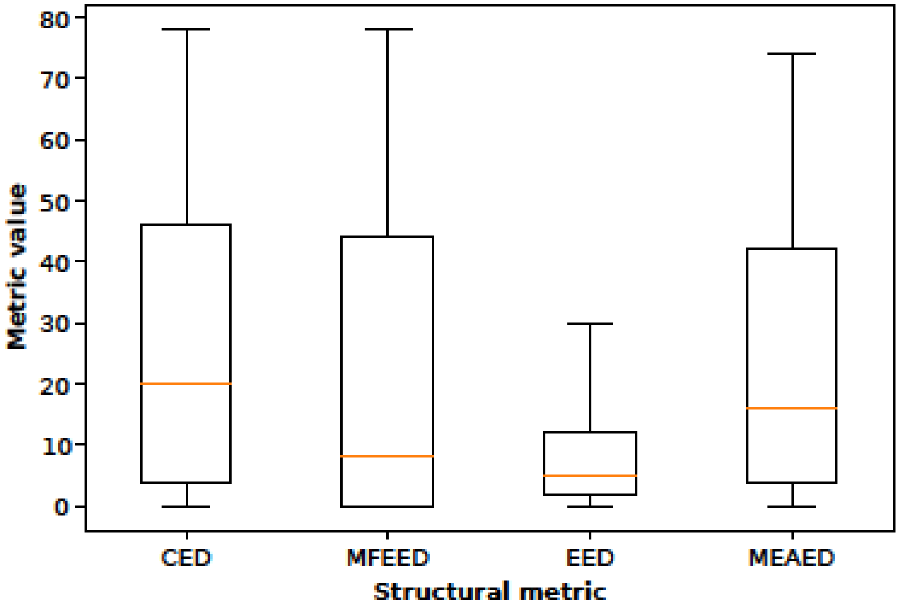

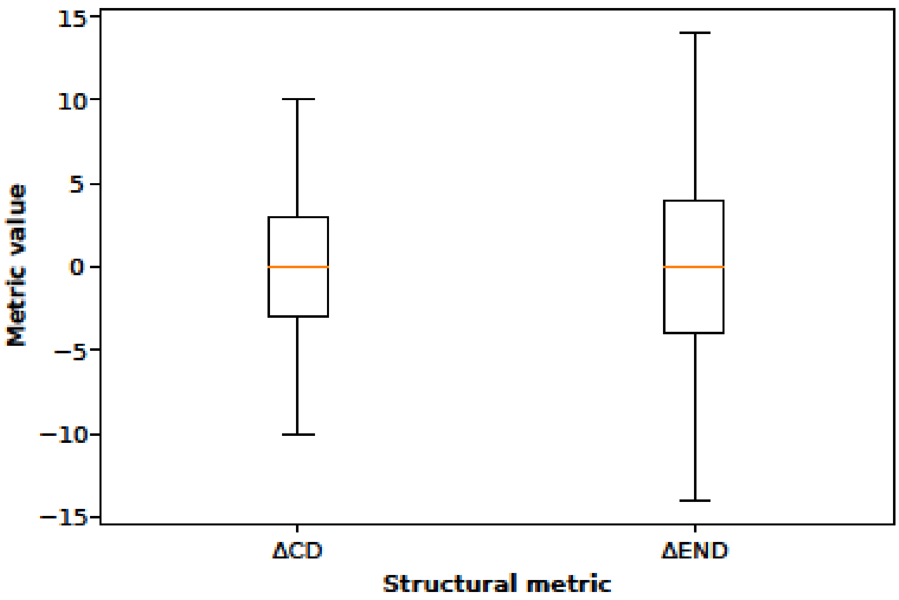

Supplementary Data Figure 1. Global distribution of structural metrics using box-and-whisker plots. We show the distribution of each metric our filtered database of possible sSNVs. Orange line shows median and box encloses central 75% of data. Whiskers enclose central 90%.

Supplementary Data Figure 2 - Calculation of Edit Distance

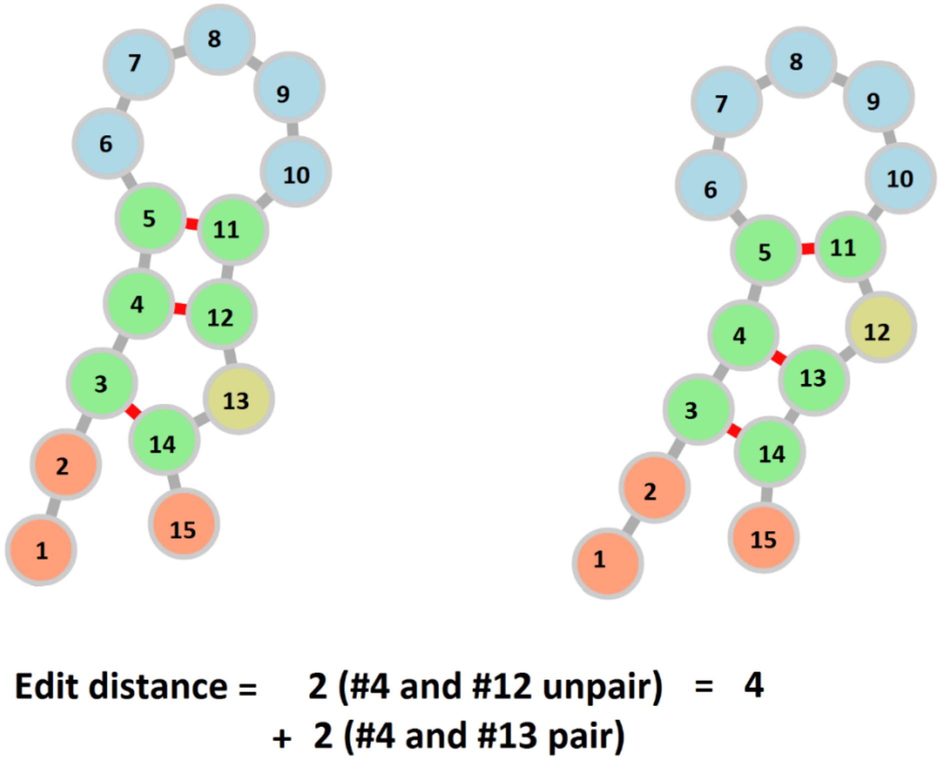

Supplementary Data Figure 2. Calculation of edit distance. The “edit distance” between two mRNA secondary structures with the same primary structure is the number of “edits” needed to transform one structure into another. Creation and removal of base pairs (the only possible changes) each count for two edits.

SUPPLEMENTARY DATA FIGURE 3 - Structural Metrics Over All Synonymous SNVs

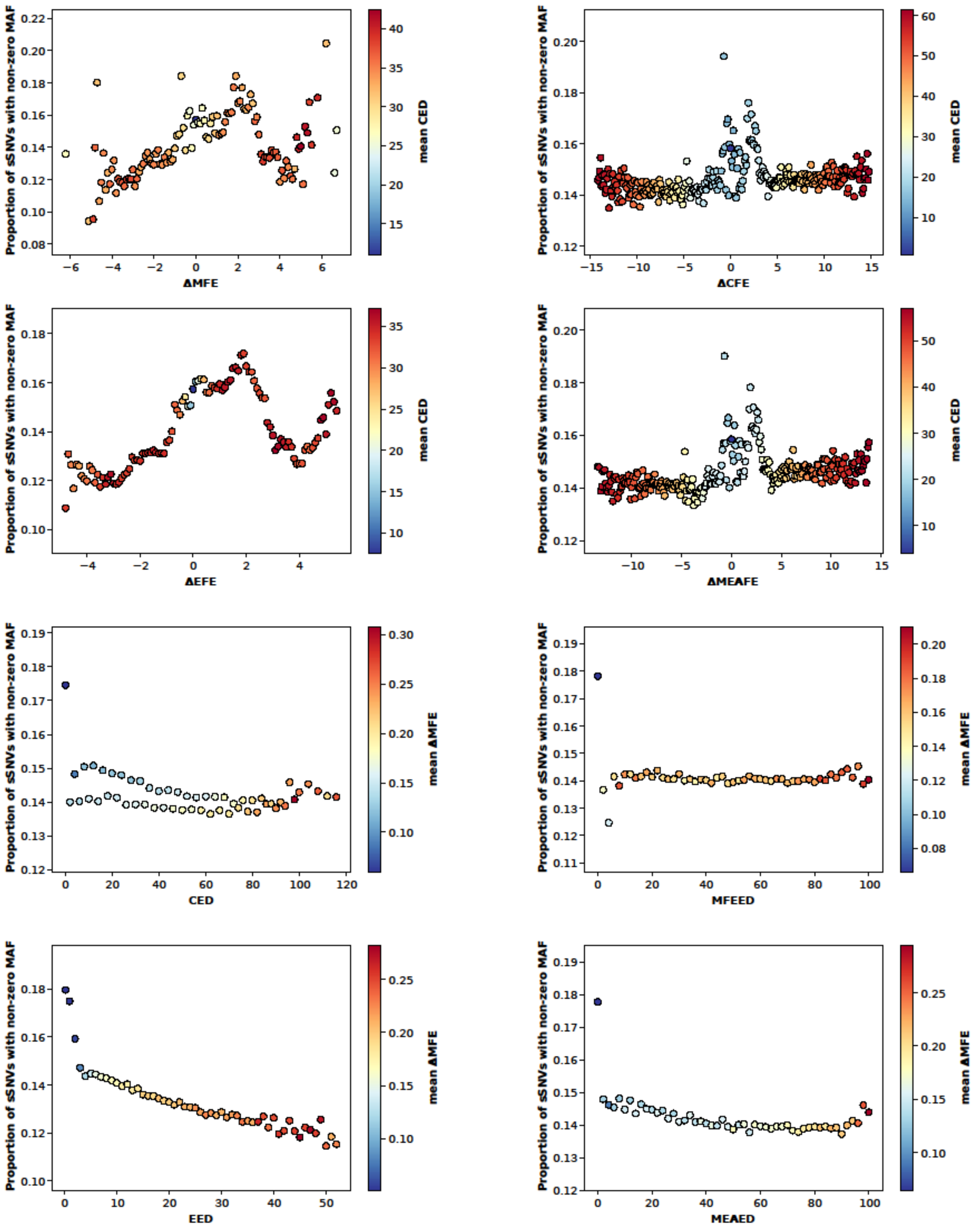

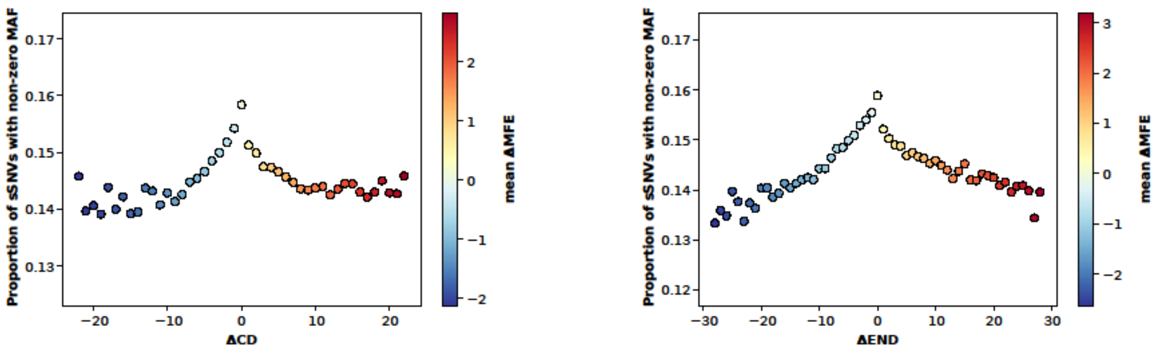

Supplementary Data Figure 3: All Vienna metrics across full set of synonymous variants. For each of our 10 Vienna metrics we plot the proportion of sSNVs with MAF>0 at each metric value. The metrics EED, ΔCD and ΔEND are rounded to the nearest integer; ΔEFE is rounded to the nearest 0.1. Metric values with fewer than 1000 sSNVs appearing in gnomAD are not shown.

SUPPLEMENTARY DATA FIGURE 4 - Structural Metrics in Contexts Constrained Against Destabilization

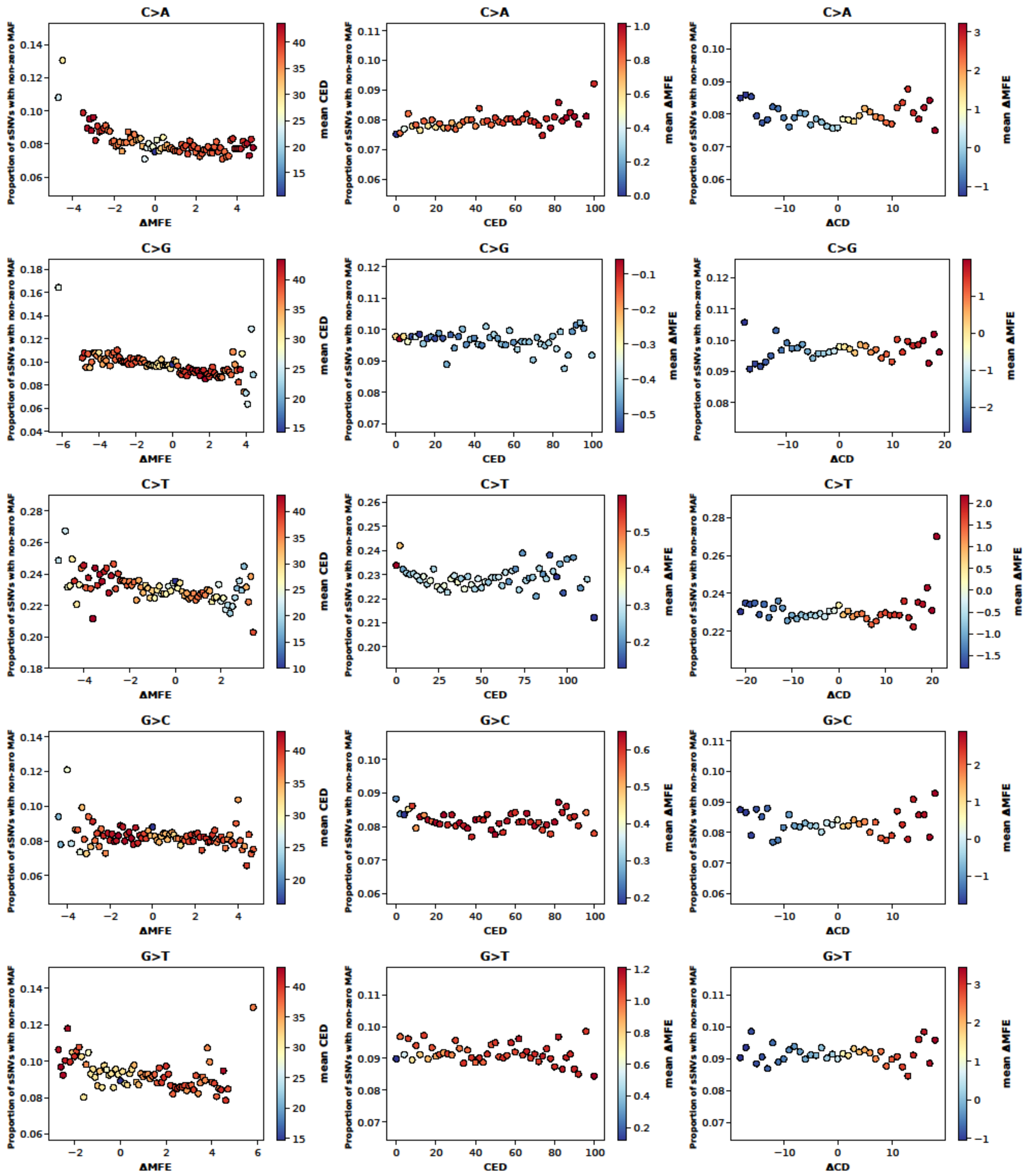

Supplementary Data Figure 4. Primary Vienna metrics in contexts constrained against destabilization. For every non-CpG-transitional context shown in Table 1A with a negative normalized slope (i.e. constraint against de-stabilization), we plot P(MAF>0) vs. our three main Vienna metrics (ΔMFE, CED, ΔCD). Values of ΔCD were rounded to the nearest integer prior to computing P(MAF>0). Metric-values with fewer than 200 sSNVs in gnomAD are not shown.

SUPPLEMENTARY DATA FIGURE 5 - Structural Metrics in Contexts Constrained Against Over-Stabilization

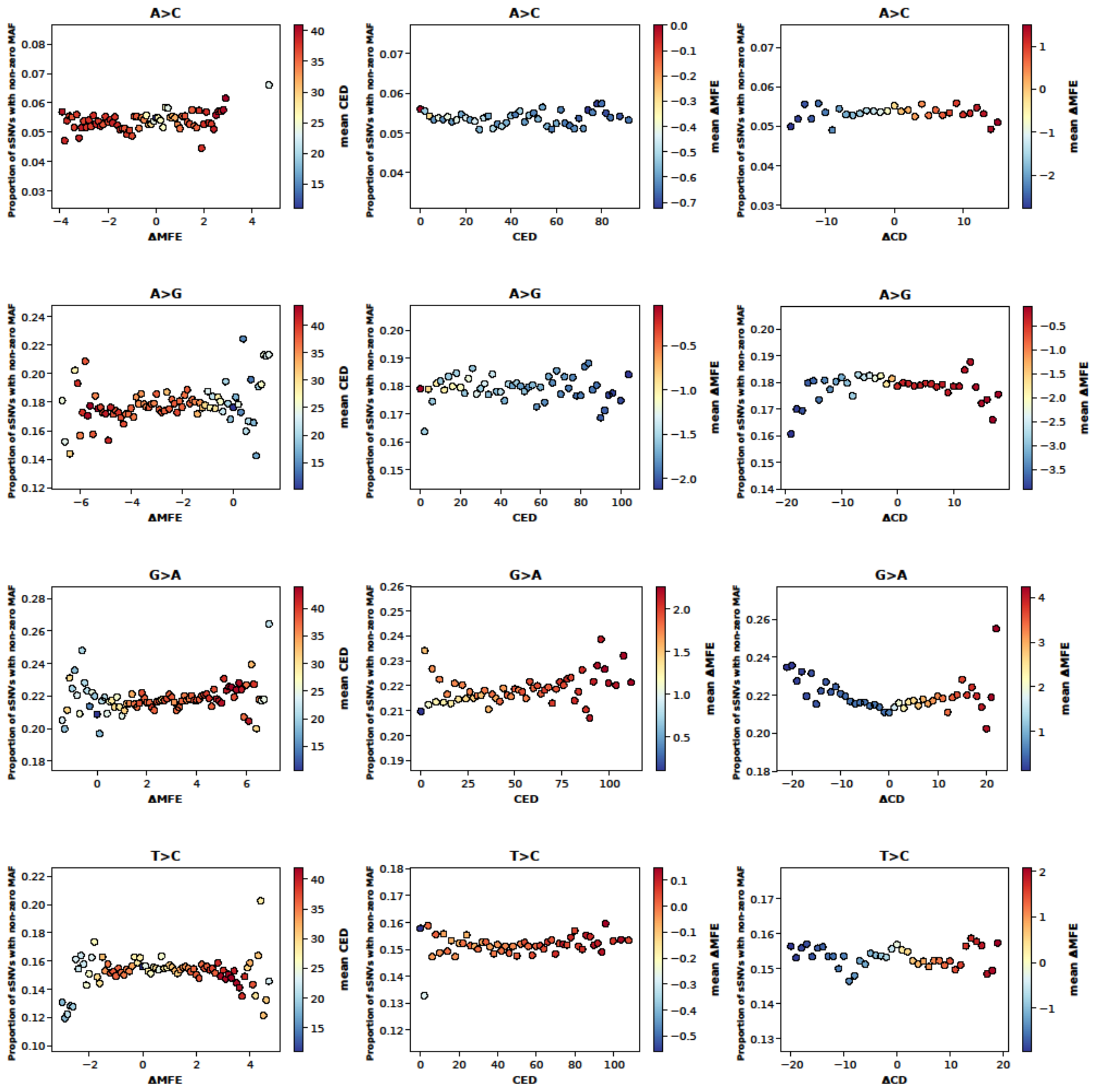

Supplementary Data Figure 5. Primary Vienna metrics in contexts constrained against over-stabilization. For every non-CpG-transitional context shown in Table 1A with a positive normalized slope (i.e. constraint against over-stabilization), we plot P(MAF>0) vs. our three main Vienna metrics (ΔMFE, CED, ΔCD). Values of ΔCD were founded to the nearest integer prior to computing P(MAF>0). Metric-values with fewer than 200 sSNVs in gnomAD are not shown.

SUPPLEMENTARY DATA FIGURE 6 - Sequence Context and SPI

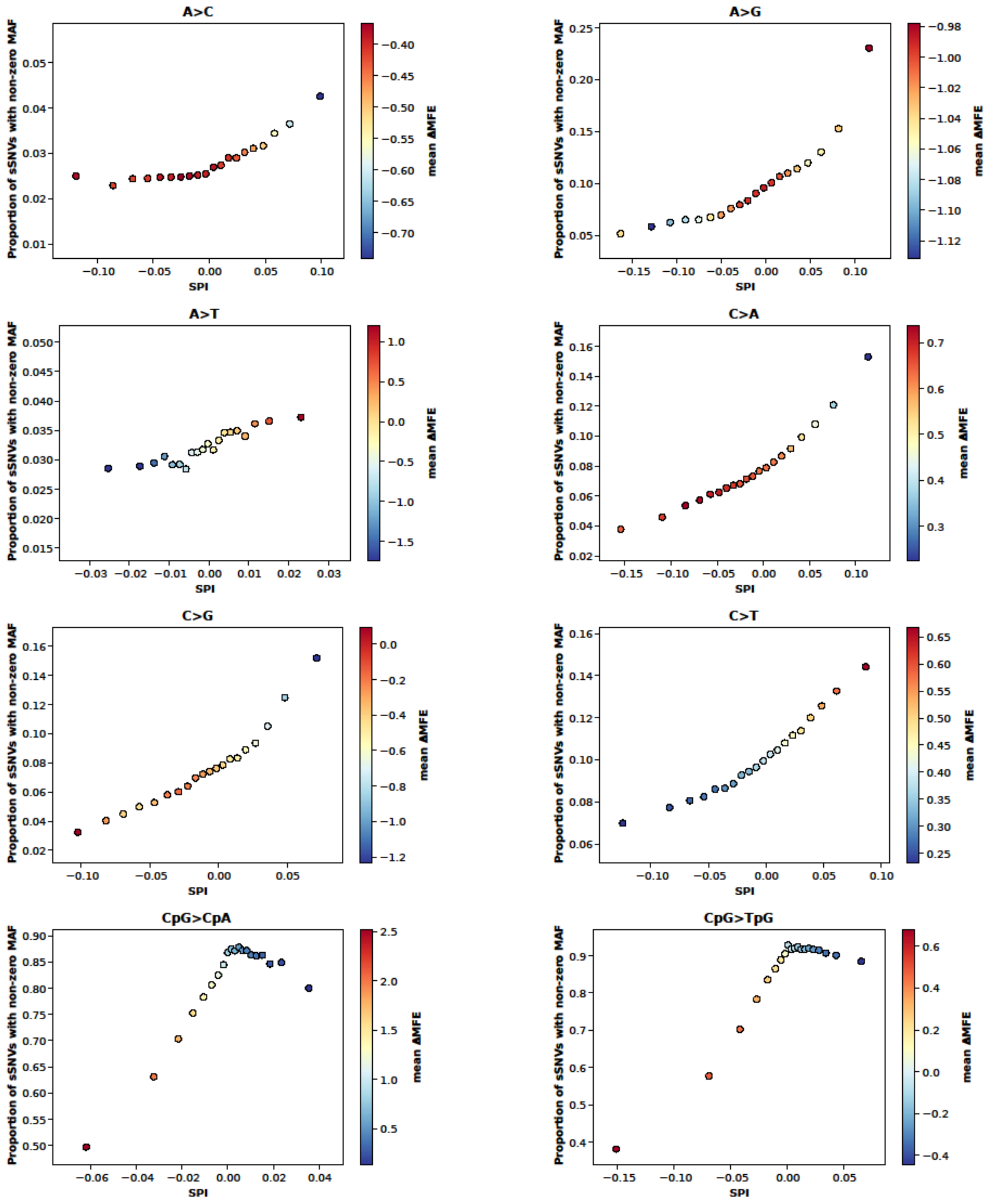

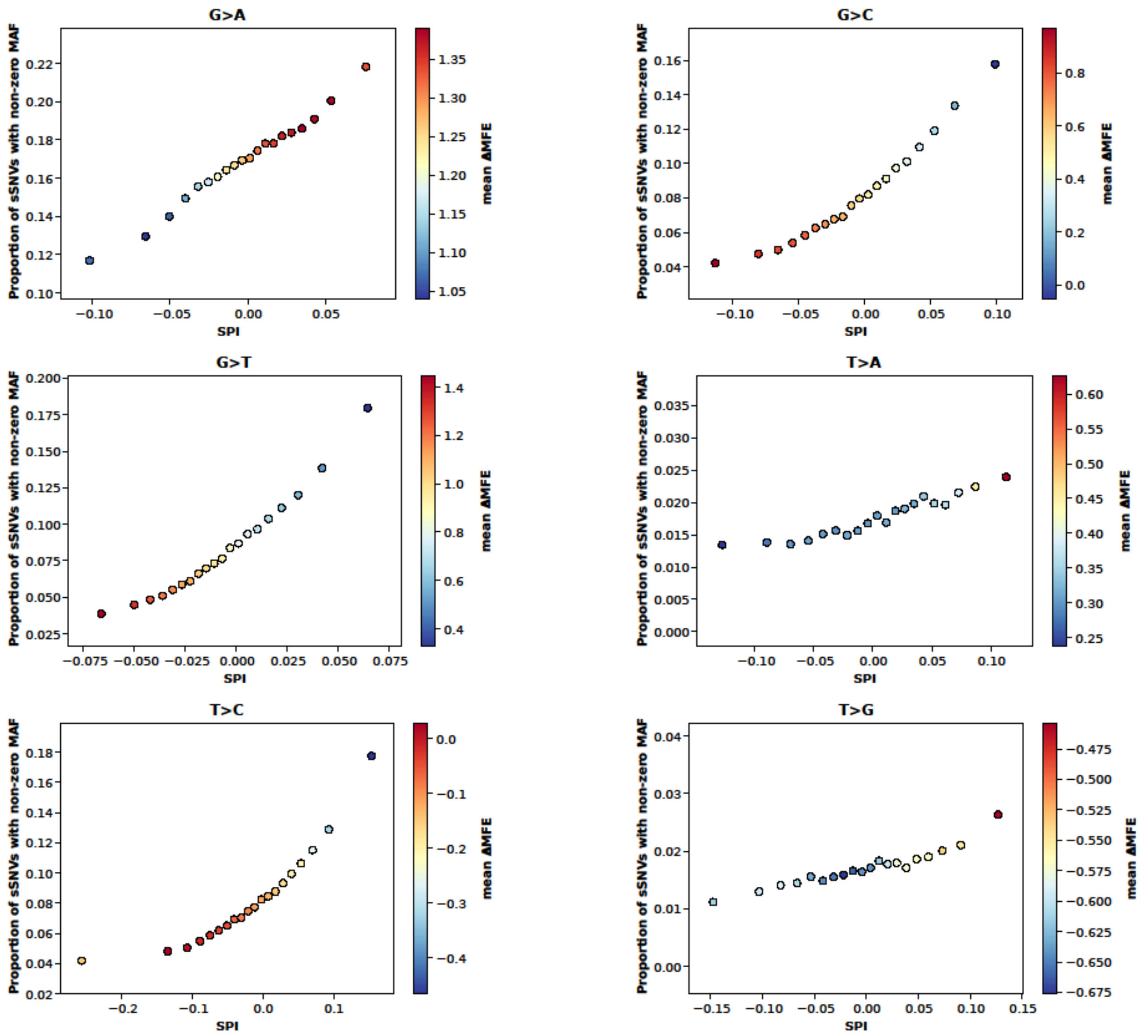

Supplementary Data Figure 6. SPI score vs. P(MAF>0). In each of our 14 mutational contexts we bin by SPI into 20 bins and then plot against P(**MAF**>0). The coloring by ΔMFE shows whether increased or decreased structure is suggested as a deleterious agent.

SUPPLEMENTARY DATA FIGURE 7 - Structural Metrics vs. log(MAF)

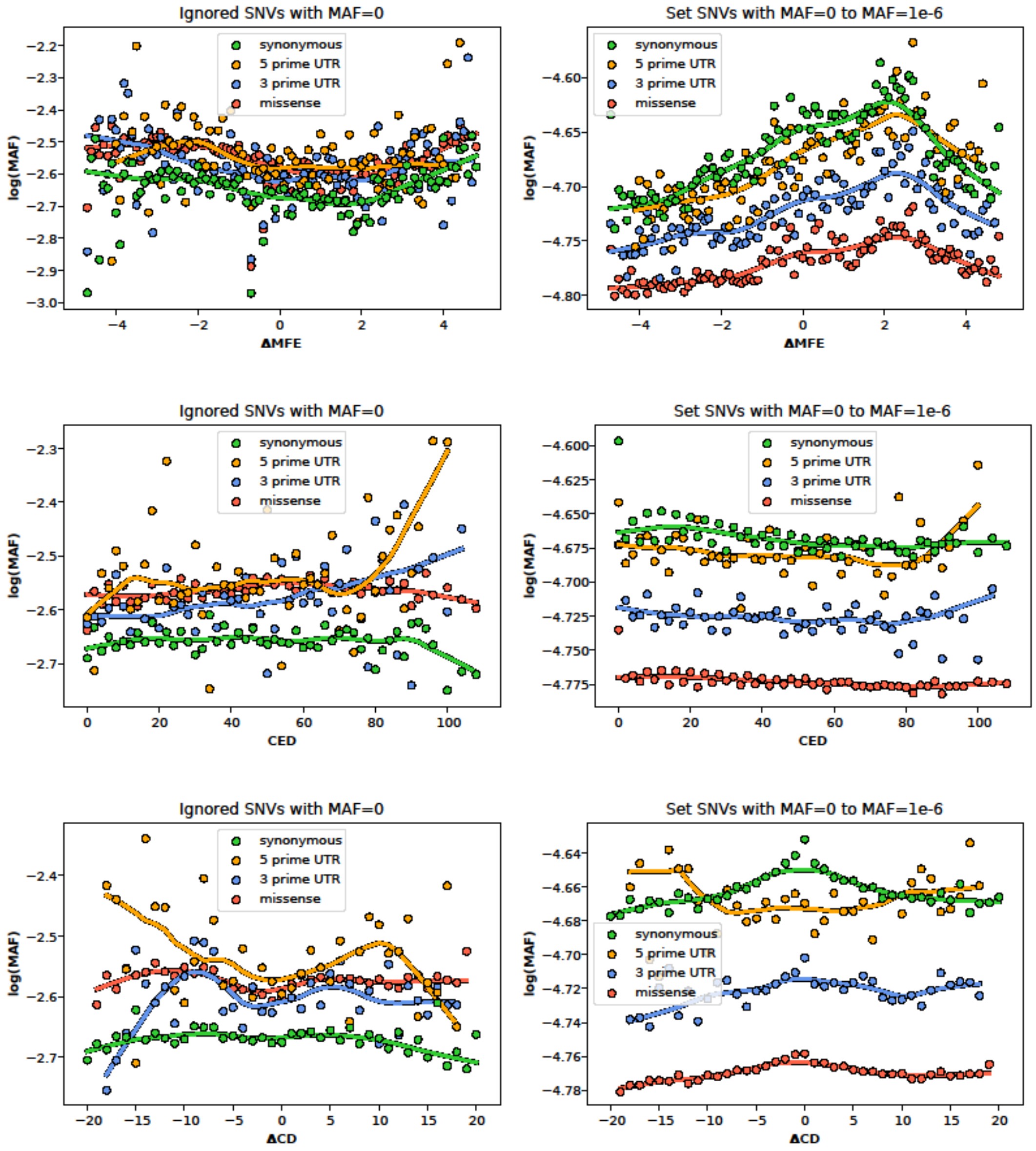

Supplementary Data Figure 7. Structural metrics vs. log(MAF). We plot the mean value of log(MAF) at each value of our three main metrics, ΔMFE, CED and ΔCD. Filtering conditions are the same as in **FIGURE 3**. In left-hand figures, SNVs with MAF=0 are omitted from this analysis; in the right-hand figures the MAF of such SNVs is set to 10^{-6}. The right-hand plots closely resemble those in **FIGURES 3** and **4**, showing that we capture the essential pattern whether we use P(MAF>0) or some variation of log(MAF) which accounts for zeroes. The left-hand plots show that variations among the positive SNV frequencies carry much less information.

SUPPLEMENTARY DATA FIGURE 8 - CHANGE IN CODON OPTIMALITY VS. MUTATION RATE

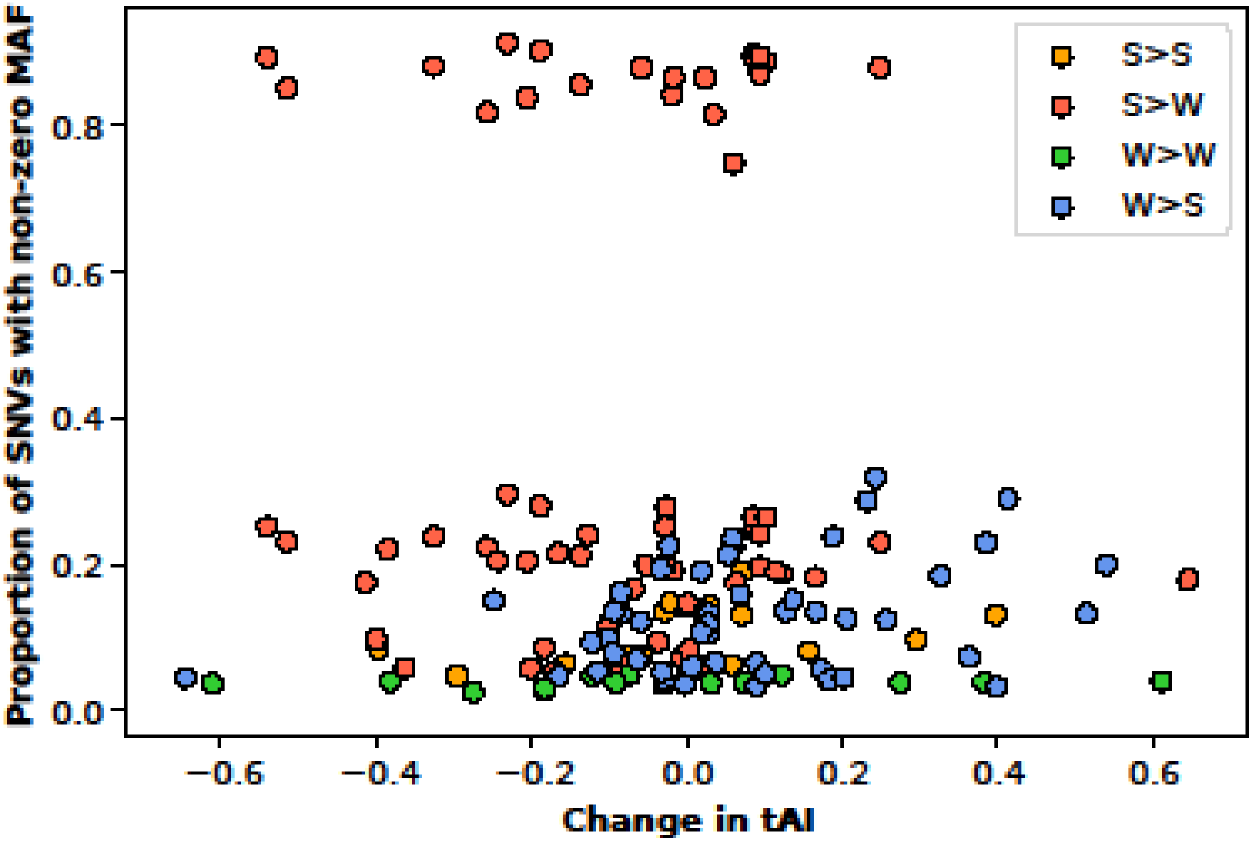

Supplementary Data Figure 8. Codon tRNA optimality vs. P(MAF>0). For 150 possible synonymous codon substitutions (also distinguishing CpG transitions in codons ending in C) we plot proportion of sSNVs with nonzero gnomAD frequency against change in tRNA Adaptation Index. The plot does not show any clear enrichment/depletion based on change in codon optimality. Legend shows weak/strong status of mutating bases. The S>W dots near the top are CpG transitions.
