# Supplementary material for: Synonymous variants that disrupt messenger RNA structure are significantly constrained in the human population
Source: Gigascience. 2021 Apr 5;10(4):giab023. doi: 10.1093/gigascience/giab023 (PMC8023685; doi:10.1093/gigascience/giab023)
Supplement: giab023_Response_to_Reviewer_Comments_Original_Submission [file giab023_response_to_reviewer_comments_original_submission.pdf]

Author's Response To Reviewer Comments

Close

We thank the reviewers for their consideration and insightful feedback on our manuscript, “Synonymous Variants that Disrupt mRNA Structure are Significantly Constrained in the Human Population.” The critiques were very helpful, and in addressing them we believe the manuscript is substantially improved over our original submission. In summary, we made the following major revisions to the manuscript:

• Added survey of existing synonymous variant scores.
• Expanded discussion of conservation for RNA structure.
• Expanded description of link between RNA structure, gene expression and codon optimality.
• Converted our whole set of SNVs from GRCh37 to GRCh38 and added data from an additional 76,000 healthy individuals (gnomAD version 3), giving us both WGS and WES population data and augmenting core dataset from 18 million to 21 million sSNVs.
• Updated Figure 3-6 with new GRCh38 and gnomAD data. As population frequency data is improved by direct mapping to GRCh38, we dropped the extra “Liftover” step from Fig. 2.
• Added predicted binding-status to SPI so that it works consistently across all contexts and re-scaled as to PHRED values for easy interpretation.
• To simplify useability, we added a new Summarized RNA-Folding (SURF) score which takes maximum PHRED-score from any of our 11 RNA metrics. We added Figure 7 showing connection to population constraint.
• Updated software license to remove any commercial restrictions, and registered application with bio.tools and SciCrunch.org.

Please also find attached a red-line version of the manuscript that highlights all changes from the original submission. Additionally, we provide detailed responses to each reviewer’s critiques, below.

-- RESPONSE TO REVIEWER #1 --

1. The introduction misses a large volume of previous work on classification of synonymous variants. Please read Zishuo et al. Front. Genet., 10: 914 (2019) for some of those missing articles. For example, there were "four sSNV-specific effect predictors: SilVA (Silent Variant Analyzer) (Buske et al., 2013), regSNPs-splicing (Zhang et al., 2017), DDIG-SN (Detecting Disease-causing Genetic SynoNymous variants) (Livingstone et al., 2017), and IDSV (Identification of Deleterious Synonymous Variants) (Shi et al., 2019)."

• We thank the reviewer for bringing these tools to our attention. We had originally omitted these tools as the focus of our manuscript is on synonymous variants that impact mRNA structure, and tools like SiLVA focus on splicing. However, we agree that when discussing synonymous variants, it is appropriate to include background on all tools for sSNVs. As such we have added a paragraph to that surveys these tools and also describe a novel one (SynTool) (BACKGROUND, P5)

2. The authors also seem to be unaware of a previous demonstration of the relations between minor allele frequency of single nucleotide variants and base-pairing probabilities at the mutation site and between MAF and RNA solvent accessibility at the mutation site (Yang et al. RNA 23:14-22, 2016).

• We thank the reviewer – we were indeed not aware of this paper. We address Yang’s tool RNAsnap in the context of our own work, describing its focus on protein-bound tertiary structures as well as its finding of a correlation between RNA structure and population frequencies. (BACKGROUND, P4)

3. Figure 3: it would be interesting to examine the relation between MAF and dMFE/CFEED/dCD (and other structural properties) as the literature above.

• We agree that a study of the relation of our structural metrics to log(MAF) (as was performed in Yang et al., 2016) would be most interesting. We have added SUPPLEMENTARY FIGURE 7 showing the correlation of log(MAF) against the metrics dMFE, CFEED and dCD (BACKGROUND, P4).

• NOTE: to build these plots, we had to decide on a value of log(0). (This was not a problem for Yang et al., since 1000 Genomes contains only SNVs with positive MAFs). We adopted two separate approaches: setting log(0)=-6 (a reasonable value, since the gnomAD cohort has around 100,000 individuals, meaning the lowest possible log(MAF) is -5), or throwing out all SNVs with MAF=0. If we take the first approach, we essentially recover FIGURE 3. As might be expected, if we take the second approach (resulting in the loss of about 80% of our data) the pattern is lost. This seems to justify our decision to use P(MAF>0) as our primary y-statistic.

4. Figure 4: can you explain why peaked for dMFE at 2?

• This peak reflects the dominant contribution from CpG transitions, which have the highest gnomAD frequencies and an average dMFE of around 2. We added a note mentioning this important detail. (ANALYSIS, P7).

5. There are much more than a few examples of pathogenic SNVs shown in Table 2. In fact, there are a few thousands in HGMD (Stenson, et al. Human Genetics, 136(6), 665-677(2017)). A few datasets are available online to download. Authors should examine more examples for those neutral (from 1000 genomes) and pathogenic SNVs.

• The reviewer is quite correct that there are thousands of known pathogenic SNVs in HGMD. However, our goal was to present sSNVs that caused disease through their disruption of mRNA structure, and the HGMD does not consider RNA structure as an etiology, only including synonymous variants if they occur in splicing or regulatory regions. (Stenson et al., 2017, PMID 28349240, Table 1). The lack of mRNA structurally altering synonymous variants in databases such as ClinVar and HGMD was actually one of the motivating factors for our study, and it is one that the synonymous-variant community must grapple with. To lend credibility to this claim, we note that a 2019 Nature Communications article on synonymous mutations in cancer asserts that “no changes in RNA secondary structures of cancer genes have been proven so far.” (Nat Commun 10, 2569 (2019) https://doi.org/10.1038/s41467-019-10489-2).
• For the purposes of evaluating our metrics against known sSNVs that disrupt mRNA structure the best resource we could find was the dbDSM (the Database of Deleterious Synonymous Mutations) which only had 17 sSNVs tagged specifically as disease-causal via their effect on mRNA structure. After checking the original publications of these 17 sSNVs, we found only 6 where we felt that true causality for RNA structure had been demonstrated (several other publications speculated a relationship but did not perform the necessary functional work). Through extensive literature searching we found an additional three sSNVs variants that clearly demonstrate a pathogenic disease etiology through alteration in mRNA structure and have added them to TABLE 2. For the 9 known sSNVs clinically implicated for mRNA structural pathogenicity, all of them are successfully predicted to be pathogenic by our structural metrics. (TABLE 2, P40).
• With regard to the addition of neutral SNVs in Table 2 – we understand the reviewer’s wish to have neutral sSNVs represented in the table. However, given that the vast majority of our sSNVs are predicted to be structurally neutral by our metrics, this would require adding a large sample of neutral sSNVs to give a good representation. We did however modify our core dataset to include the more recently released gnomAD 3 data, which included the addition of WGS data for over 76,000 healthy subjects and updated all figures accordingly (see DATA DESCRIPTION, P7). We also have uploaded the complete dataset to GigaDB (see AVAILABILITY OF SUPPORTING DATA AND MATERIALS, P25).

6. "However, the potential for pathogenic synonymous variants that impact RNA folding in human genetic disease remains largely unknown". This statement is incorrect as such an effect was demonstrated before. I would like to see the discussion changed.

• The reviewer is correct – it is now scientific dogma that such variants exist. We modified the discussion, changing “remains largely unknown” to “is not universally appreciated.” (DISCUSSION, P15)

7. "In the absence of functional tools that would aid in the simultaneous assessment of both nsSNVs and sSNVs". This statement is incorrect as these tools do exist. I would like to see the discussion changed.

• We agree with the reviewer’s recommendation. We changed “In the absence of functional tools” to “Given the scarcity of RNA-structure-specific tools”. (DISCUSSION, P15)

-- RESPONSE TO REVIEWER #2 --

1. This discussion of the relationship between mRNA secondary structure/thermodynamic stability and mRNA intracellular stability/propensity to degradation is very vague. The authors failed to cite some key publications in the field (e.g. Ding et al., In vivo genome-wide profiling of RNA secondary structure reveals novel regulatory features. Nature, 2014; Mauger et al, mRNA structure regulates protein expression through changes in functional half-life, PNAS, 2019, see also ref. therein).

• We thank the reviewer for raising this valid criticism and have greatly expanded the BACKGROUND (P3) to further explain that "RNA stability" refers to the RNA's physical structure, which derives primarily from base-pair stacks, rather than to its functional lifetime or its ability to avoid degradation. We emphasize however that stable RNAs do, in fact, have longer lifetimes, express more protein and suffer later degradation, and hypothesize this is the preeminent reason why stability is selected for in the first place. In the DISCUSSION (Molecular mechanisms underlying constraint of sSNVs, P17-18) we elaborate that stable RNA structures are thought to regulate ribosomal speed, possibly with the goal of preventing collisions, which are known to trigger degradation pathways. We discuss Ding et al., 2014 and Mauger et al., 2019 as well as several other publications cited by them, including:
o Presnyak V et al. Codon optimality is a major determinant of mRNA stability. Cell. 2015 Mar 12;160(6):1111-24. doi: 10.1016/j.cell.2015.02.029. PMID: 25768907
o Wan Y et al. Landscape and variation of RNA secondary structure across the human transcriptome. Nature. 2014 Jan 30;505(7485):706-9. doi: 10.1038/nature12946. PMID: 24476892
o Mustoe AM et al. Pervasive Regulatory Functions of mRNA Structure Revealed by High-Resolution SHAPE Probing. Cell. 2018 Mar 22;173(1):181-195.e18. doi: 10.1016/j.cell.2018.02.034. Epub 2018 Mar 15. PMID: 2955126
o Kertesz M. Genome-wide measurement of RNA secondary structure in yeast. Nature. 2010 Sep 2;467(7311):103-7. doi: 10.1038/nature09322. PMID: 20811459

2. The interplay between local and global mRNA secondary structure and mRNA intracellular stability as well as overall protein expression and folding/activity has not been fully understood yet. While it has been suggested that nucleotides that stabilize mRNA secondary structure within the coding regions do enable high protein expression, the effect of such variants in the 5'UTRs is usually quite the opposite, as the increase in 5'UTR stability usually decreases efficiency of translation initiation. The authors should carefully discuss these different scenarios in the context of their observations.

• We thank the reviewer for this relevant point, which we agree should be included. We have added a couple sentences explaining that, in contrast to the CDS, stability in 5' UTRs is associated with decreased expression due to the interference of structure with translation initiation. (BACKGROUND – P3-4)
• Interestingly, in most organisms this effect extends into the coding region – the first 30 or so bases have reduced stability (Gu et al., doi:10.1371/journal.pcbi.1000664) However, warm-blooded animals are partially an exception to this rule – it holds only in the most G+C-rich genes, which may be attributable to the CpG islands that generally co-locate with mammal and bird gene promoters. We also describe this extension of this effect into the coding region, and why mammals might be an exception. DISCUSSION (Molecular mechanisms underlying constraint of sSNVs, P17)

3. Notably, because in the human genome optimal codons tend to be GC rich, the structure of the genetic code inherently and generally disallows coding sequences with both highly optimal codons and low structure/thermodynamic stability. The authors should also investigate the limits and the impact of codon usage/codon optimality on the secondary structure. How many pathogenic sSNVs would lead to a change of an optimal codon to a non-optimal codon?

• We thank the reviewer and agree that we did not give due attention to codon optimality. We have added SUPPLEMENTARY FIGURE 8, which shows no depletion or enrichment of mutations that increase/decrease optimality, suggesting it is not a major factor in our findings. We also note that in most REF>ALT contexts the change in optimality (increase or decrease) is largely the same over all codon classes. Thus, our decomposition of all mutations into 14 contexts has largely accounted for differences in codon optimality. However, it is of note that we do observe more constraint in mutations G+C > A+T, that is, the optimal to sub-optimal direction. We certainly do not rule out the effects of codon optimality, which is why we included the tRNA Adaptation Index (tAI) in our list of null features for SPI. (BACKGROUND, P4, DISCUSSION, P17-18)

-- RESPONSE TO EXTERNAL EDITORIAL ADVISOR --

Major:

1. It would be useful to know if the mediator variables, in particular the ones that are up-stream or downstream nucleotides have a simple, mechanistic explanation in terms of the energy model RNA folding. Since folding energies in paired regions depend not just on the base pair but on the stacking interaction with the adjacent base pairs, there may be a strong correlation of effects with adjacent nucleotides that could simply be read of the pairing tables in the Turner energy model. The authors should check whether this already provides an explanation. Similarly, CpGs in RNA have a correlation with base pairing since the stacking of GC with CG pairs has the largest stabilizing energy contribution, whence secondary structure have a propensity for CpG pairing with CpG.

• We thank the reviewer for this interesting point, upon which we have added a paragraph in the DISCUSSION. Interestingly, the sign of the Mediator indicates that stacks are enriched for mutations, not constrained. We hypothesize that this observation could be due to stable structures having a greater tolerance for de-stabilization than unstable structures. Relatedly, we observe the Mediator usually has the same S/W type as the reference (e.g. a trailing T in the A>G context). The co-occurrence of A/Ts suggests that unstable regions are in little danger of over-stabilization. (DISCUSSION, P14-15)

Minor:

1. This study is not the first to identify large-scale selection pressures on RNA structures throughout in a genome/transcriptome-wide setting. While to my knowledge the approach here is novel, prior work on this topic should be to be referenced and the conclusions need to be discussed at least briefly also in this context. See e.g. Seemann et al doi: 10.1101/gr.208652.116 and the references therein. A paper that explicitly considers structural constraints in mRNAs is, albeit for fruitfly
https://www.sciencedirect.com/science/article/pii/S0300908411002811?via%3Dihub

• We thank the reviewer for bringing these works to our attention and agree that our description of related studies was inadequate. In addition to the two references suggested we have added descriptions of RNAsnap and SNPfold, two tools that assess disruptions of RNA structure, the former using population frequencies like our own work. We also cite the secondary-structure prediction tools used in Seemann (CMFinder) and the reviewer's other reference Findeiss (RNAz) (BACKGROUND, P4).

2. Consider using the more common notation capital-Delta for differences in MFE etc.

• We agree with the reviewer's suggestion and have changed structural metric name "dMFE" to "ΔMFE" throughout, similarly for the metrics dEFE, dCFE, dMEAFE, dCD and dEND.

3. the terminology CFEED (Centroid Free Energy Edit Distance) is awkward, why the "free energy" in the name? Usually these quantities are referred to as base pair distances since they just count the caradinality of the set of base pairs in which two structures differ. Or are you using a different edit distance?

• Changed Also changed CFEED to CED due to the phrase "free energy" not being relevant. Analogously, EFEED has been changed to EED.

4. Also the definition of the metrics that are used in the main text should be given there instead of being relegated to the supplement.

• We have recast so that the three metrics are defined in the same sentences they are introduced. (DATA DESCRIPTION, P6)

5. how were positions closer than 50 nt to the ends of transcripts handled?

• We certainly should have mentioned this! For such positions we just take the leading/trailing 101 bases as our 101-base window. Added explanation. (METHODS, P19)

6. spell out acronyms at first usage

• This has been fixed throughout.

7. -avoid * as multiplication symbol, use standard mathematical notation

• This has been fixed throughout the text and figures.

Close
