# Supplementary material for: Synonymous variants that disrupt messenger RNA structure are significantly constrained in the human population
Source: Gigascience. 2021 Apr 5;10(4):giab023. doi: 10.1093/gigascience/giab023 (PMC8023685; doi:10.1093/gigascience/giab023)
Supplement: giab023_Response_to_Reviewer_Comments_Revision_1 [file giab023_response_to_reviewer_comments_revision_1.pdf]

Author's Response To Reviewer Comments

Close

GigaDB submission has been completed and reference updated in the manuscript accordingly.

Close
