# Supplementary material for: Synonymous variants that disrupt messenger RNA structure are significantly constrained in the human population
Source: Gigascience. 2021 Apr 5;10(4):giab023. doi: 10.1093/gigascience/giab023 (PMC8023685; doi:10.1093/gigascience/giab023)
Supplement: giab023_Reviewer_1_Report_Original_Submission — Yaoqi Zhou -- 11/5/2020 Reviewed [file giab023_reviewer_1_report_original_submission.pdf]

Reviewer Report

Title: Synonymous Variants that Disrupt mRNA Structure are Significantly Constrained in the Human Population

Version: Original Submission **Date:** 11/5/2020

Reviewer name: Yaoqi Zhou

Reviewer Comments to Author:

GIGA-D-20-00178
Synonymous Variants that Disrupt mRNA Structure are Significantly Constrained in the Human Population
Jeffrey B.S. Gaither, Ph.D.; Grant E. Lammi; James L. Li; David M. Gordon; Harkness C. Kuck; Benjamin J. Kelly; James R. Fitch; Peter White, Ph.D.
GigaScience
Gaither et al performed a large-scale study for the effect of mRNA structures on synonymous variants in human population. They examined every possible variant in the human transcriptome and found that synonymous variants predicted to disrupt mRNA structure have significantly lower rates of incidence in the human population. This is a nice, comprehensive study. However, similar conclusions were found in previous studies. Moreover, a number of tools were developed for synonymous variants. The authors fail to discuss them and make a comparison. In addition, there are thousands of known pathogenic SNVs, not 7 as claimed by the paper.
Comments.
1) The introduction misses a large volume of previous work on classification of synonymous variants. Please read Zishuo et al. Front. Genet., 10: 914 (2019) for some of those missing articles. For example, there were "four sSNV-specific effect predictors: SilVA (Silent Variant Analyzer) (Buske et al., 2013), regSNPs-splicing (Zhang et al., 2017), DDIG-SN (Detecting Disease-causing Genetic SynoNymous variants) (Livingstone et al., 2017), and IDSV (Identification of Deleterious Synonymous Variants) (Shi et al., 2019)."
2) The authors also seem to be unaware of previously demonstrated the relations between minor allele frequency of single nucleotide variants and base-pairing probabilities at the mutation site and between MAF and RNA solvent accessibility at the mutation site (Yang et al. RNA 23:14-22, 2016).
3) Figure 3: it would be interesting to examine the relation between MAF and dMFE/CFEED/dCD (and other structural properties) as the literature above.
4) Figure 4: can you explain why peaked for dMFE at 2?
5) There are much more than a few examples of pathogenic SNVs shown in Table 2. In fact, there are a few thousands in HGMD (Stenson, et al. Human Genetics, 136(6), 665-677(2017)). A few datasets are available online to download. Authors should examine more examples for those neutral (from 1000 genomes) and pathogenic SNVs.
6) "However, the potential for pathogenic synonymous variants that impact RNA folding in human genetic disease remains largely unknown". This statement is incorrect as such effect was demonstrated

before. I would like to see changed discussions.

7) "In the absence of functional tools that would aid in the simultaneous assessment of both nsSNVs and sSNVs". This statement is incorrect as these tools do exist. I would like to see changed discussions.

Methods

Are the methods appropriate to the aims of the study, are they well described, and are necessary controls included? Choose an item.

Conclusions

Are the conclusions adequately supported by the data shown? Choose an item.

Reporting Standards

Does the manuscript adhere to the journal's guidelines on [minimum standards of reporting?]() Choose an item.

Choose an item.

Statistics

Are you able to assess all statistics in the manuscript, including the appropriateness of statistical tests used? Choose an item.

Quality of Written English

Please indicate the quality of language in the manuscript: Choose an item.

Declaration of Competing Interests

Please complete a declaration of competing interests, considering the following questions:

- Have you in the past five years received reimbursements, fees, funding, or salary from an organisation that may in any way gain or lose financially from the publication of this manuscript, either now or in the future?
- Do you hold any stocks or shares in an organisation that may in any way gain or lose financially from the publication of this manuscript, either now or in the future?
- Do you hold or are you currently applying for any patents relating to the content of the manuscript?
- Have you received reimbursements, fees, funding, or salary from an organization that holds or has applied for patents relating to the content of the manuscript?
- Do you have any other financial competing interests?
- Do you have any non-financial competing interests in relation to this paper?

If you can answer no to all of the above, write 'I declare that I have no competing interests' below. If your reply is yes to any, please give details below.

I declare that I have no competing interests.

I agree to the open peer review policy of the journal. I understand that my name will be included on my report to the authors and, if the manuscript is accepted for publication, my named report including any attachments I upload will be posted on the website along with the authors' responses. I agree for my report to be made available under an Open Access Creative Commons CC-BY license (http://creativecommons.org/licenses/by/4.0/). I understand that any comments which I do not wish to be included in my named report can be included as confidential comments to the editors, which will not be published.

Choose an item.

To further support our reviewers, we have joined with Publons, where you can gain additional credit to further highlight your hard work (see: https://publons.com/journal/530/gigascience). On publication of this paper, your review will be automatically added to Publons, you can then choose whether or not to claim your Publons credit. I understand this statement.

Yes Choose an item.
