# Supplementary material for: Synonymous variants that disrupt messenger RNA structure are significantly constrained in the human population
Source: Gigascience. 2021 Apr 5;10(4):giab023. doi: 10.1093/gigascience/giab023 (PMC8023685; doi:10.1093/gigascience/giab023)
Supplement: giab023_Reviewer_2_Report_Original_Submission — Anton A Komar, PhD -- 11/18/2020 Reviewed [file giab023_reviewer_2_report_original_submission.pdf]

Reviewer Report

Title: Synonymous Variants that Disrupt mRNA Structure are Significantly Constrained in the Human Population

Version: Original Submission **Date:** 11/18/2020

Reviewer name: Anton A Komar, PhD

Reviewer Comments to Author:

In this study, Jeffrey Gaither and co-authors developed a novel computational tool for the large-scale generation and assessment of secondary RNA structures and folding statistics in the transcriptomes of any desired species. They have used this tool to analyze RNA folding statistics of the 45,800 human transcripts and assess the impact of 469 million single nucleotide variants in these transcripts. They have found that synonymous variants predicted to disrupt mRNA structure have significantly lower rates of incidence in the human population. They have focused their analysis on the synonymous single nucleotide variants (sSNVs) found recently to be important players in human health and disease. Their analysis of the approximately 18 million synonymous variants found in the human transcriptome (with the exception of those that could impact canonical splice sites), further revealed that there is significant selection against sSNVs predicted to disrupt the given transcript's local mRNA secondary structure, supporting their hypothesis that RNA structure itself plays a critical role in human health and disease. The authors concluded that the Structural Predictivity Index (SPI) that they have developed to quantify structural constraint operating on any synonymous variant, may serve as a valuable tool to evaluate the potential pathogenic impact of synonymous variants in human population in general and individual patients in particular.

This manuscript is addressing a very interesting and important topic. I have a number of remarks though:

1. This discussion of the relationship between mRNA secondary structure/thermodynamic stability and mRNA intracellular stability/propensity to degradation is very vague. The authors failed to cite some key publications in the field (e.g. Ding et al., In vivo genome-wide profiling of RNA secondary structure reveals novel regulatory features. Nature, 2014; Mauger et al, mRNA structure regulates protein expression through changes in functional half-life, PNAS, 2019, see also ref. therein).
2. The interplay between local and global mRNA secondary structure and mRNA intracellular stability as well as overall protein expression and folding/activity has not been fully understood yet. While it has been suggested that nucleotides that stabilize mRNA secondary structure within the coding regions do enable high protein expression, the effect of such variants in the 5'UTRs is usually quite the opposite, as the increase in 5'UTR stability usually decreases efficiency of translation initiation. The authors should carefully discuss these different scenarios in the context of their observations.
3. Notably, because in the human genome optimal codons tend to be GC rich, the structure of the genetic code inherently and generally disallows coding sequences with both highly optimal codons and low structure/thermodynamic stability. The authors should also investigate the limits and the impact of codon usage/codon optimality on the secondary structure. How many pathogenic sSNVs would lead to a

change of an optimal codon to a non-optimal codon?

Methods

Are the methods appropriate to the aims of the study, are they well described, and are necessary controls included? Choose an item.

Conclusions

Are the conclusions adequately supported by the data shown? Choose an item.

Reporting Standards

Does the manuscript adhere to the journal's guidelines on minimum standards of reporting? Choose an item.

Choose an item.

Statistics

Are you able to assess all statistics in the manuscript, including the appropriateness of statistical tests used? Choose an item.

Quality of Written English

Please indicate the quality of language in the manuscript: Choose an item.

Declaration of Competing Interests

Please complete a declaration of competing interests, considering the following questions:

- Have you in the past five years received reimbursements, fees, funding, or salary from an organisation that may in any way gain or lose financially from the publication of this manuscript, either now or in the future?
- Do you hold any stocks or shares in an organisation that may in any way gain or lose financially from the publication of this manuscript, either now or in the future?
- Do you hold or are you currently applying for any patents relating to the content of the manuscript?
- Have you received reimbursements, fees, funding, or salary from an organization that holds or has applied for patents relating to the content of the manuscript?
- Do you have any other financial competing interests?
- Do you have any non-financial competing interests in relation to this paper?

If you can answer no to all of the above, write 'I declare that I have no competing interests' below. If your reply is yes to any, please give details below.

I declare that I have no competing interests

I agree to the open peer review policy of the journal. I understand that my name will be included on my report to the authors and, if the manuscript is accepted for publication, my named report including any attachments I upload will be posted on the website along with the authors' responses. I agree for my report to be made available under an Open Access Creative Commons CC-BY license (http://creativecommons.org/licenses/by/4.0/). I understand that any comments which I do not wish to be included in my named report can be included as confidential comments to the editors, which will not be published.

Choose an item.

To further support our reviewers, we have joined with Publons, where you can gain additional credit to further highlight your hard work (see: https://publons.com/journal/530/gigascience). On publication of this paper, your review will be automatically added to Publons, you can then choose whether or not to claim your Publons credit. I understand this statement.

Yes Choose an item.
